# Supplementary material for: Temperature variability projections remain uncertain after constraining them to best performing Large Ensembles of individual Climate Models
Source: Nat Commun. 2025 Dec 13;17:314. doi: 10.1038/s41467-025-67005-y (PMC12789089; doi:10.1038/s41467-025-67005-y)
Supplement: Supplementary file 1 — Supplementary Information [file 41467_2025_67005_MOESM1_ESM.pdf]

Supplementary Information to:

Temperature variability projections remain uncertain after constraining them to best performing Large Ensembles

By Laura Suarez-Gutierrez [1,2,3] & Nicola Maher [4,5]

1. Institute for Atmospheric and Climate Science, ETH Zürich, Zurich, Switzerland
2. Laboratoire des Sciences du Climat et de l'Environnement, Institut Pierre-Simon Laplace, Paris, France
3. Meteorology and Air Quality Group, Wageningen University & Research, Wageningen, the Netherlands
4. Research School of Earth Sciences, The Australian National University, Canberra, Australia
5. ARC Centre of Excellence for Weather of the 21st Century

The Supplementary Information (SI) file provides additional data and analyses supporting the findings of the main manuscript. Section 1 of this SI includes the full account of rank-frequency temperature evaluation results for all 11 models included in the main manuscript. Section 2 describes the specific timing when warming levels are reach in each model. Section 3 provides a performance assessment of the constrained and non-constrained ensembles in capturing future variability change against the best preforming model, CESM2-LE.

1. Rank-Frequency Performance Evaluation of Temperature Variability

This section encloses the full temperature variability rank-frequency evaluation framework results for all 11 single-model initial condition large ensembles (SMILEs) considered in this study globally, against GISTEMPv4 and ERSSTv5 observations over land and ocean regions respectively, both for detrended and non-detrended temperature anomalies. For the purposes of this evaluation, monthly mean temperature anomalies are relative to the period 1961–1990, and model output data are regridded to match the observational grid.

Enclosed figures in this section include:
For each model, first, maps for the rank-frequency evaluation for December, January, February (DJF; top) and June, July, August (JJA; bottom) monthly mean temperature anomalies, followed by time series and rank-histograms for DJF (first) and JJA (second) for spatially aggregated regions. Results are first shown for land regions for detrended data for all models, followed by results for non-detrended data for all models; then for ocean regions following the dame structure. Results are shown for the following 11 SMILEs, in order of appearance: ACCESS-ESM1.5, CanESM2, CanESM5, CESM-LE, CESM2-LE, CSIRO-MK3.6, GFDL-ESM2M, GFDL-SPEAR-MED, MIROC6, MPI-GE5 and MPI-GE6.

These results are separated into the following subsections:

- Detrended Land Surface Air Temperatures
- Non-Detrended Land Surface Air Temperatures
- Detrended Ocean Surface Temperatures
- Non-Detrended Ocean Surface Temperatures

Detrended Land Surface Air Temperatures

Rank-frequency variability evaluation framework for detrended 2m air temperature (TAS) anomalies over land grid cells.

Maps show grid-cell evaluation of the simulated DJF and JJA monthly mean temperature anomalies for the 11 SMILEs included against GISTEMPv4 observations globally. Gray hatching represents where observations cluster within the 75th percentile bounds of the ensemble (12.5th to 87.5th percentiles) for more than 80% of months (light grey) or for more than 95% of months (dark grey). Red and blue shading represents where observations are larger than the ensemble maximum (red) or smaller than the ensemble minimum (blue), respectively, for more than 8% of the months (light red and blue) or for more than 20% of the months (dark red and blue). Dotted areas represent ocean areas or grid cells where observations are missing and are therefore excluded from this analysis. Colored boxes demark the boundaries of each land region assessed. The percentage of assessed grid-cells that present none of these biases, unbiased grid-cells, in given at the top (white area). For each assessed region, models are considered to provide an adequate grid-cell representation when they exhibit more than 50% of unbiased grid cells in the region, fulfilling Criteria 2 of our evaluation framework.

Time series and rank frequency histograms show spatially aggregated DJF and JJA TAS for each land region for all 11 SMILEs. Time series show the ensemble maximum and minimum (color lines) and central 75th percentile ensemble spread (shading) are shown against observations (black dots).

Rank histograms represent the frequency of each place that observations would take in a list of ensemble members ordered by ascending temperature anomaly values. Rank histograms show the observations rank frequency accumulated for 3-rank bins (bars), the running mean rank frequency over a centered n/5 rank window (lines; for 1 to n-1 ranks) and the absolute frequencies of rank 0 and n (crosses), with n the number of ensemble members, for observations (color) and perfect model rank range (gray).

To illustrate how internal variability sampling may affect rank frequencies given the non-infinite record length considered, we include a comparison to the perfect-model rank range, which shows the range of rank frequencies that each ensemble member would take if it were observations (see Methods in main article for further details). If the rank exhibited by observations (colors) is within this perfect-model range (gray; allowing a maximum 10% deviation outside of this range), the rank frequency evaluation shows an adequate model performance, and any deviations from a perfectly frank rank histogram can be assumed to be within the range of deviations that could be caused by internal variability. In the case the observations rank frequency is within the perfect-model range for all rank windows, Criteria 1 of our evaluation framework is met and this is highlighted by a green star at the top right; if not, by a red cross. Percentages at the top left show the frequency of regionally averaged observations occurring above (red) or below (blue) ensemble limits, or clustering within the central 75th percentile range (gray), analogous to the grid-cell evaluation in Criteria 2.

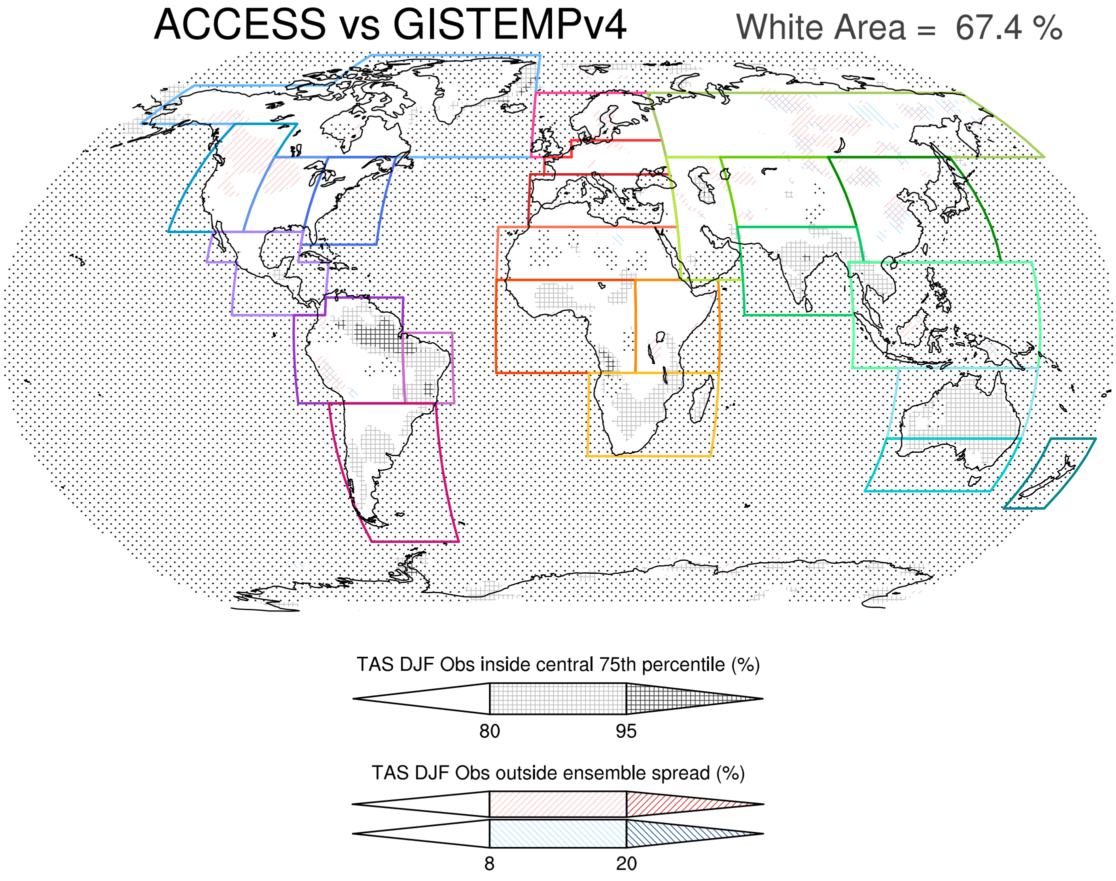
ACCESS vs GISTEMPv4
White Area = 67.4 %
TAS DJF Obs inside central 75th percentile (%)
80
95
TAS DJF Obs outside ensemble spread (%)
8
20

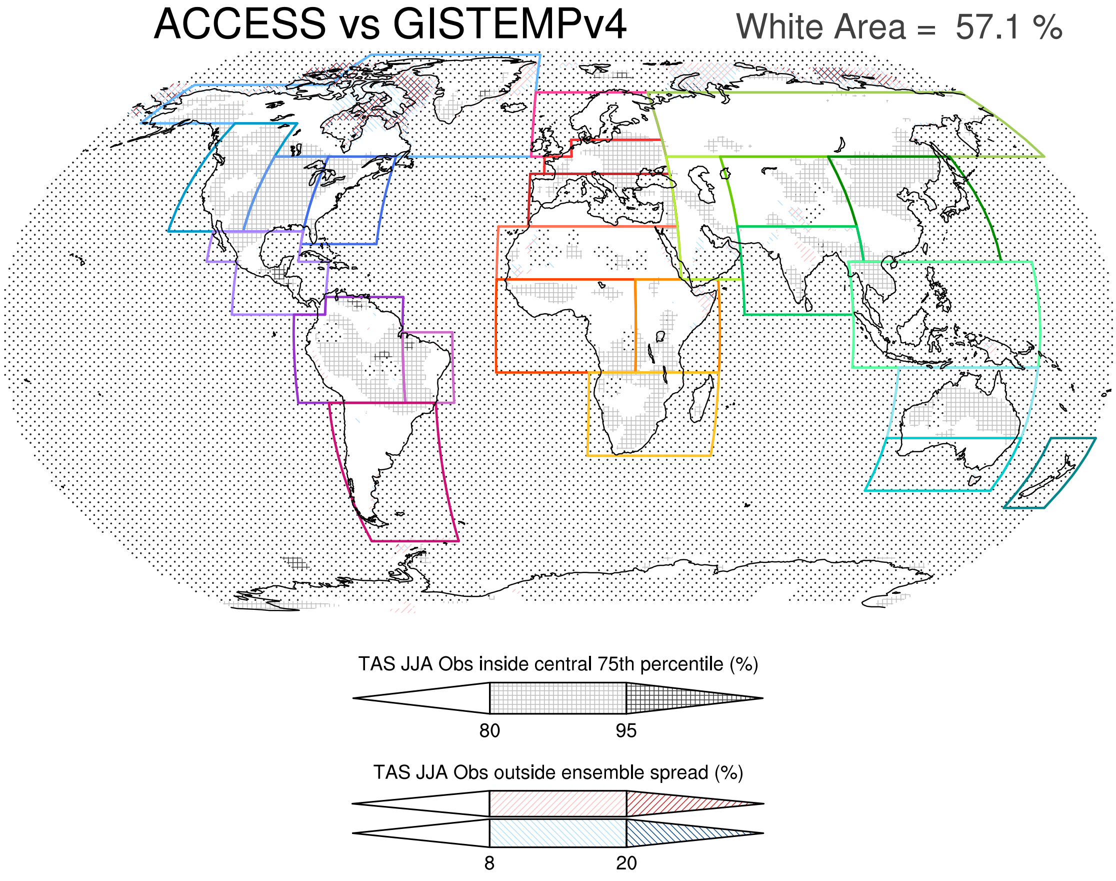
ACCESS vs GISTEMPv4
White Area = 57.1 %
TAS JJA Obs inside central 75th percentile (%)
80
95
TAS JJA Obs outside ensemble spread (%)
8
20

ACCESS vs GISTEMPv4 TAS DJF

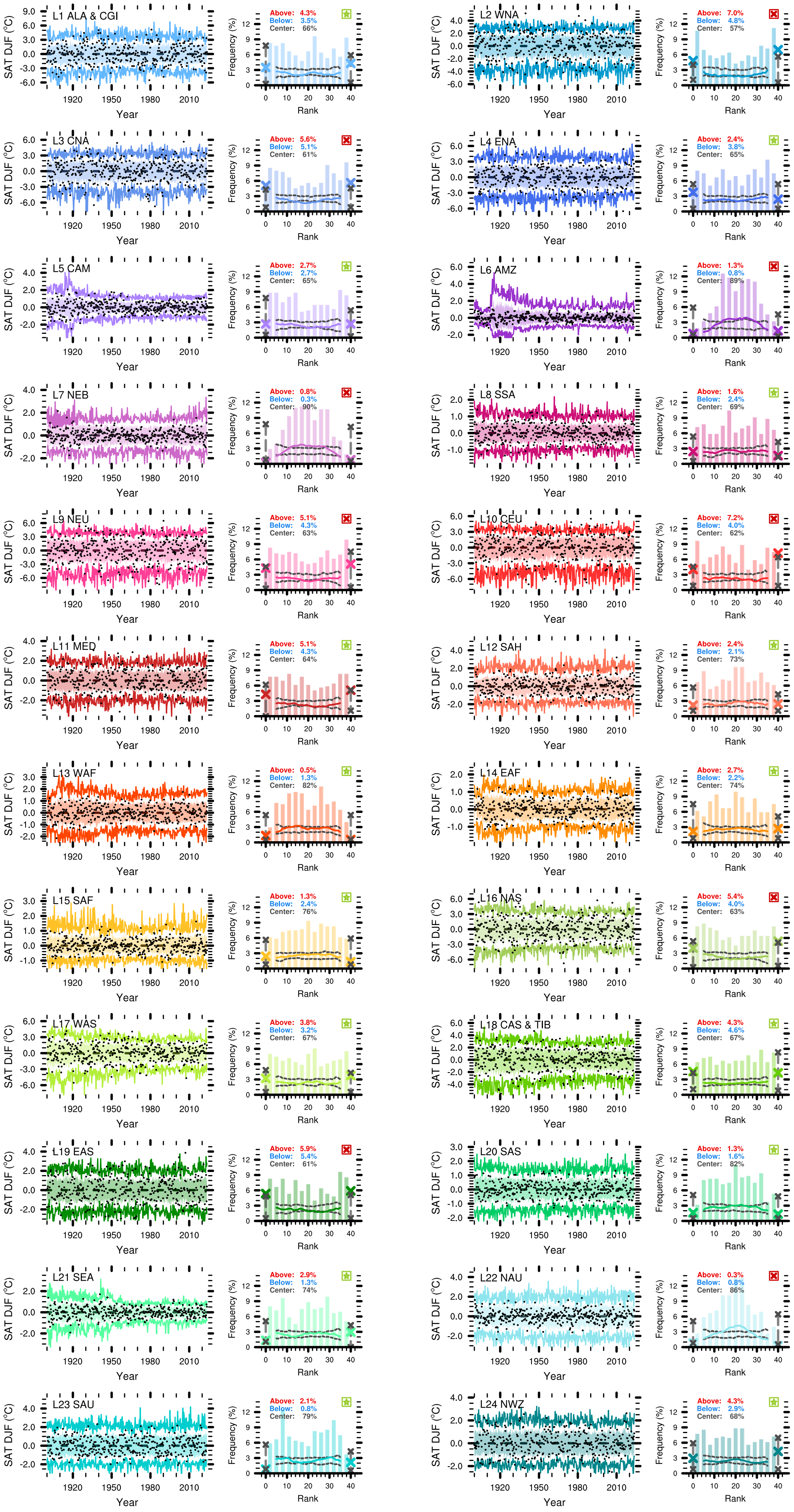

ACCESS vs GISTEMPv4 TAS JJA

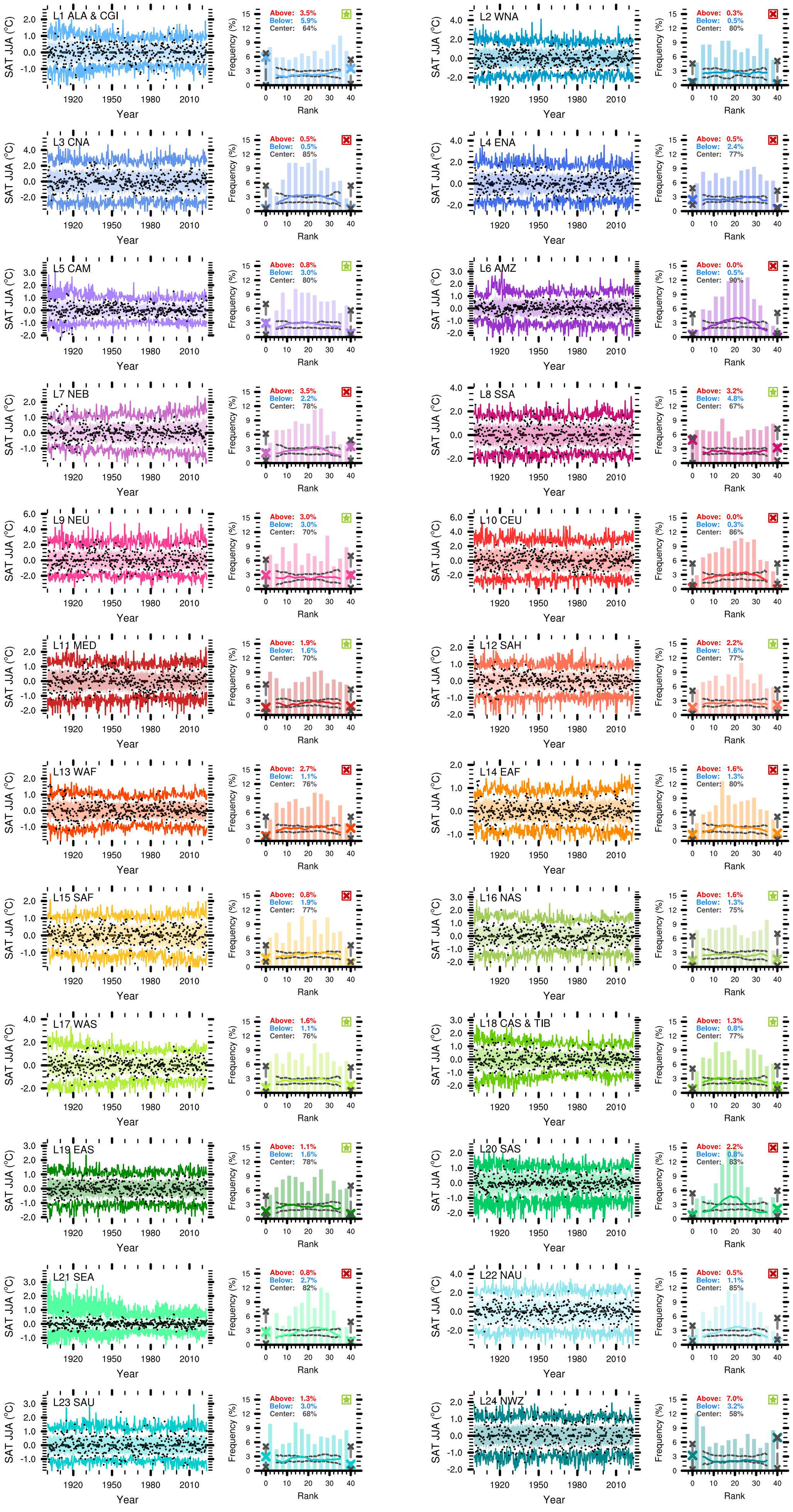

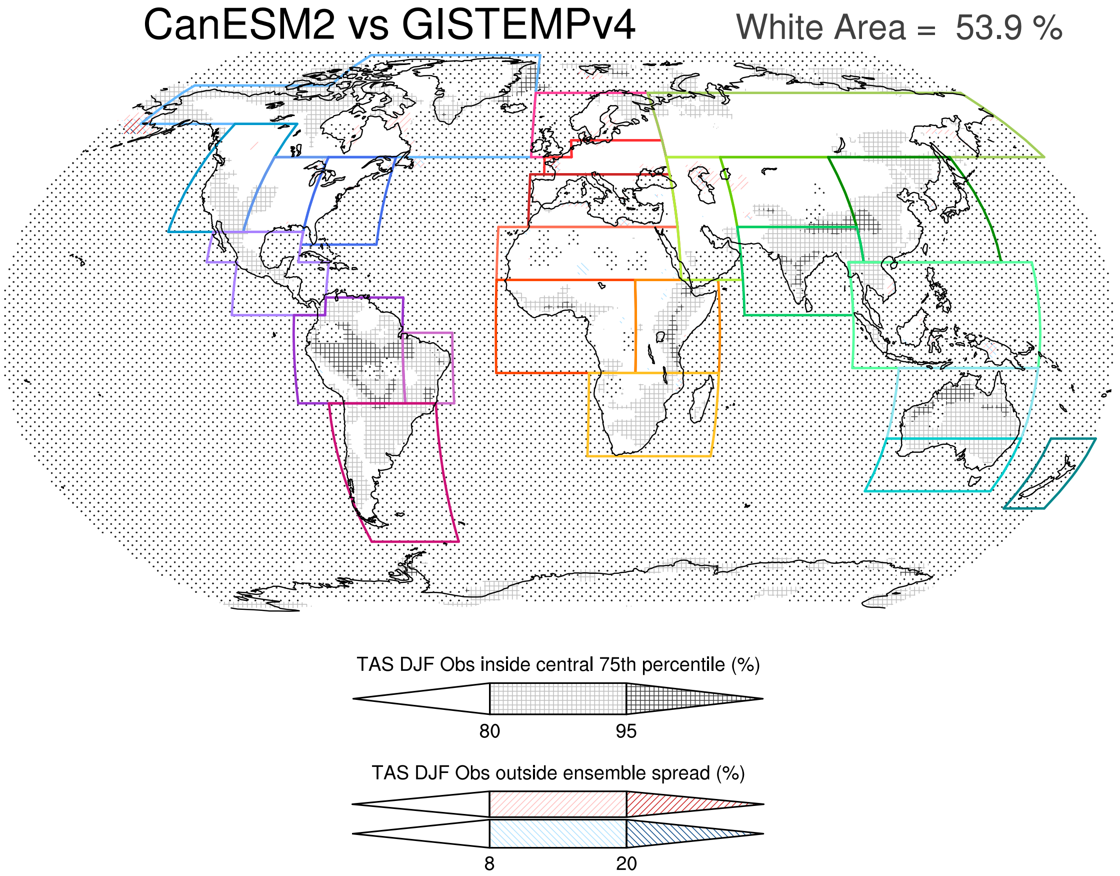
CanESM2 vs GISTEMPv4
White Area = 53.9 %
TAS DJF Obs inside central 75th percentile (%)
80
95
TAS DJF Obs outside ensemble spread (%)
8
20

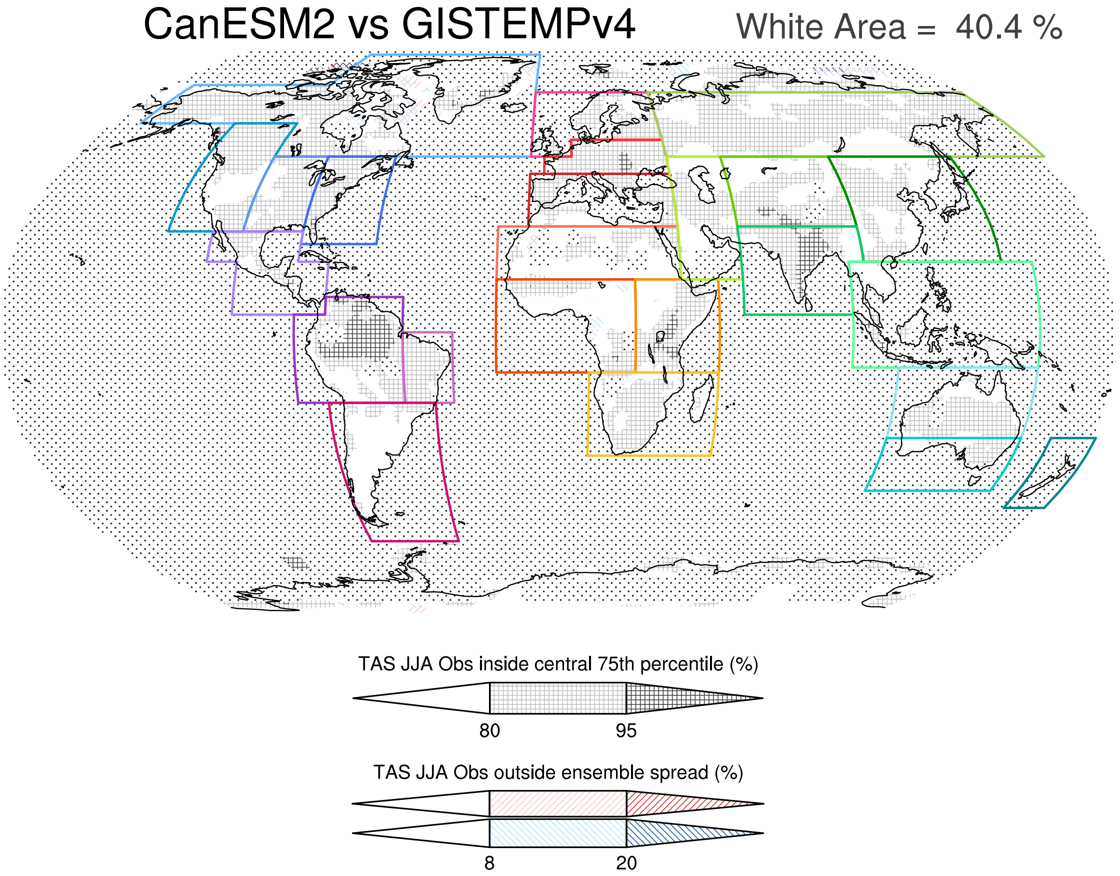
CanESM2 vs GISTEMPv4
White Area = 40.4 %
TAS JJA Obs inside central 75th percentile (%)
80
95
TAS JJA Obs outside ensemble spread (%)
8
20

CanESM2 vs GISTEMPv4 TAS DJF

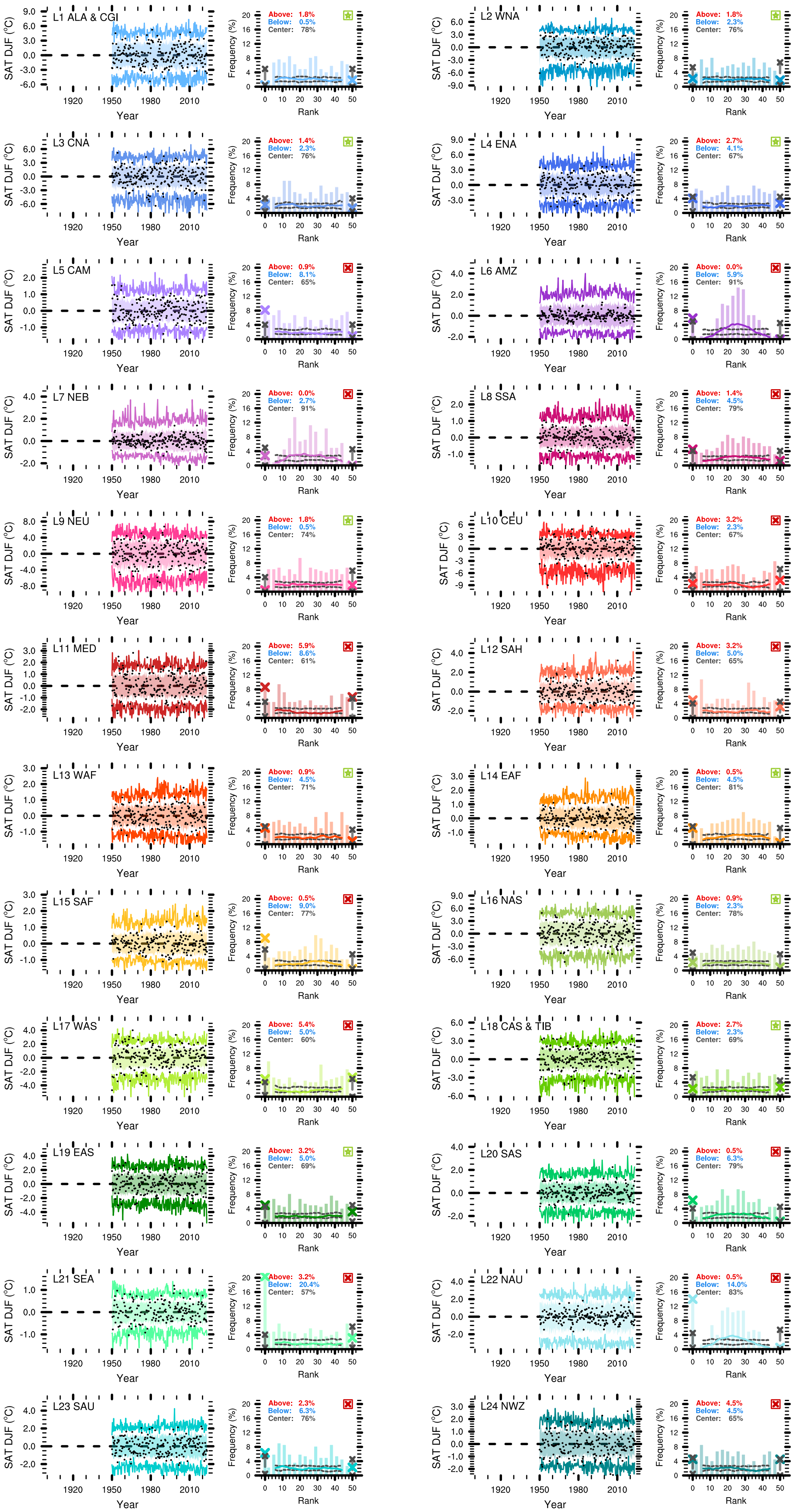

CanESM2 vs GISTEMPv4 TAS JJA

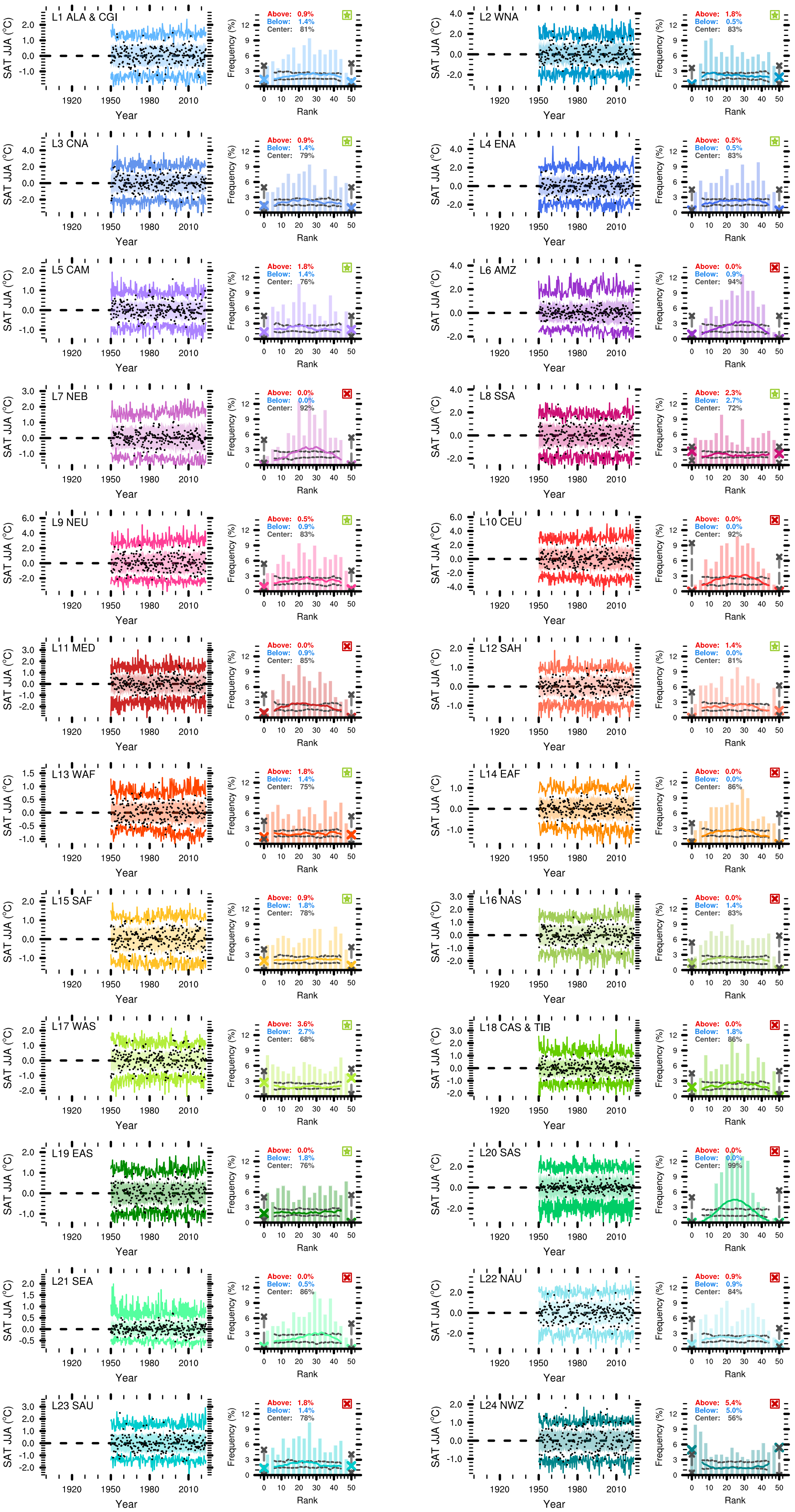

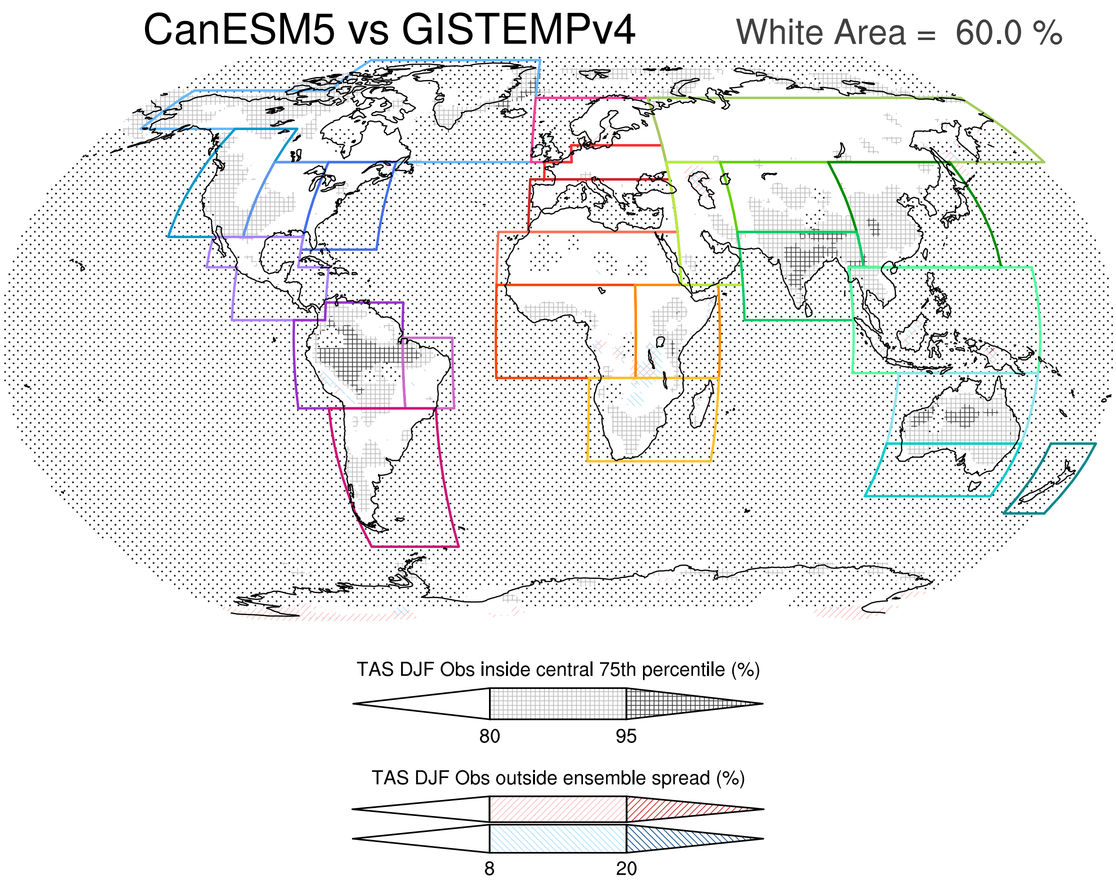
CanESM5 vs GISTEMPv4
White Area = 60.0 %
TAS DJF Obs inside central 75th percentile (%)
80
95
TAS DJF Obs outside ensemble spread (%)
8
20

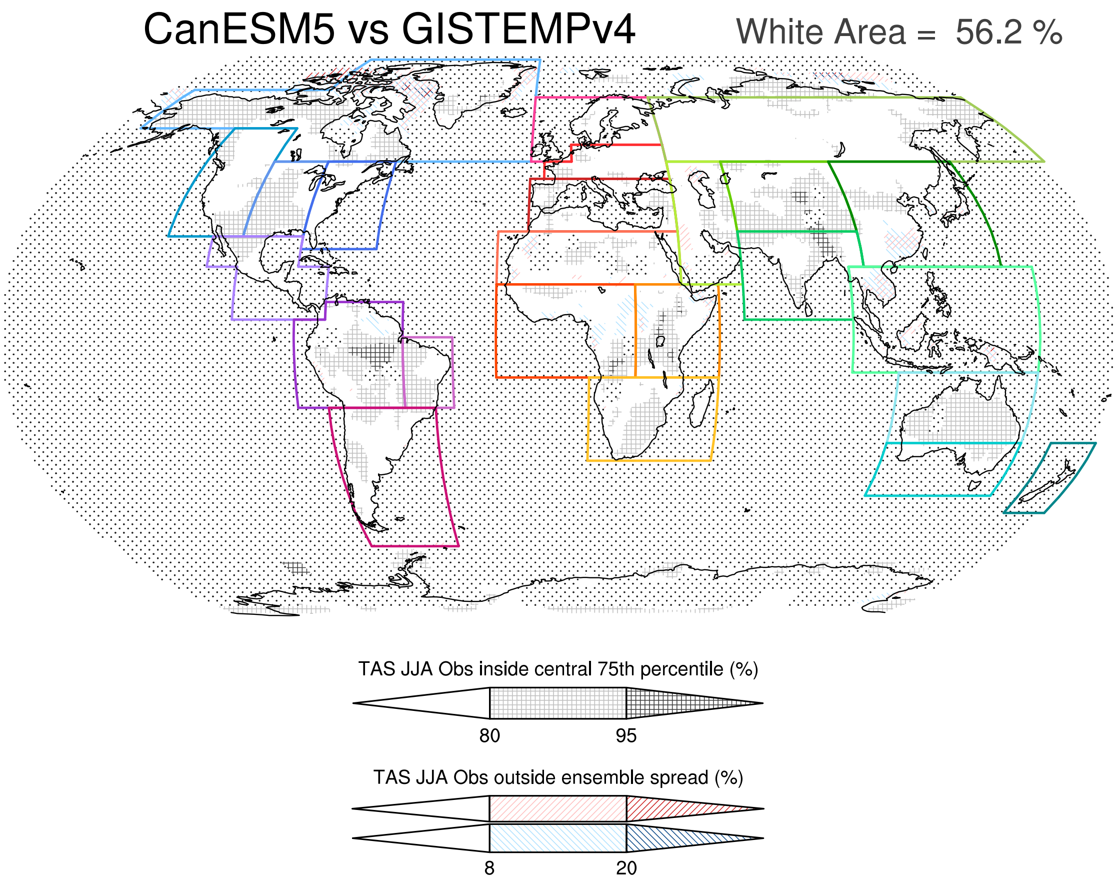
CanESM5 vs GISTEMPv4
White Area = 56.2 %
TAS JJA Obs inside central 75th percentile (%)
80
95
TAS JJA Obs outside ensemble spread (%)
8
20

CanESM5 vs GISTEMPv4 TAS DJF

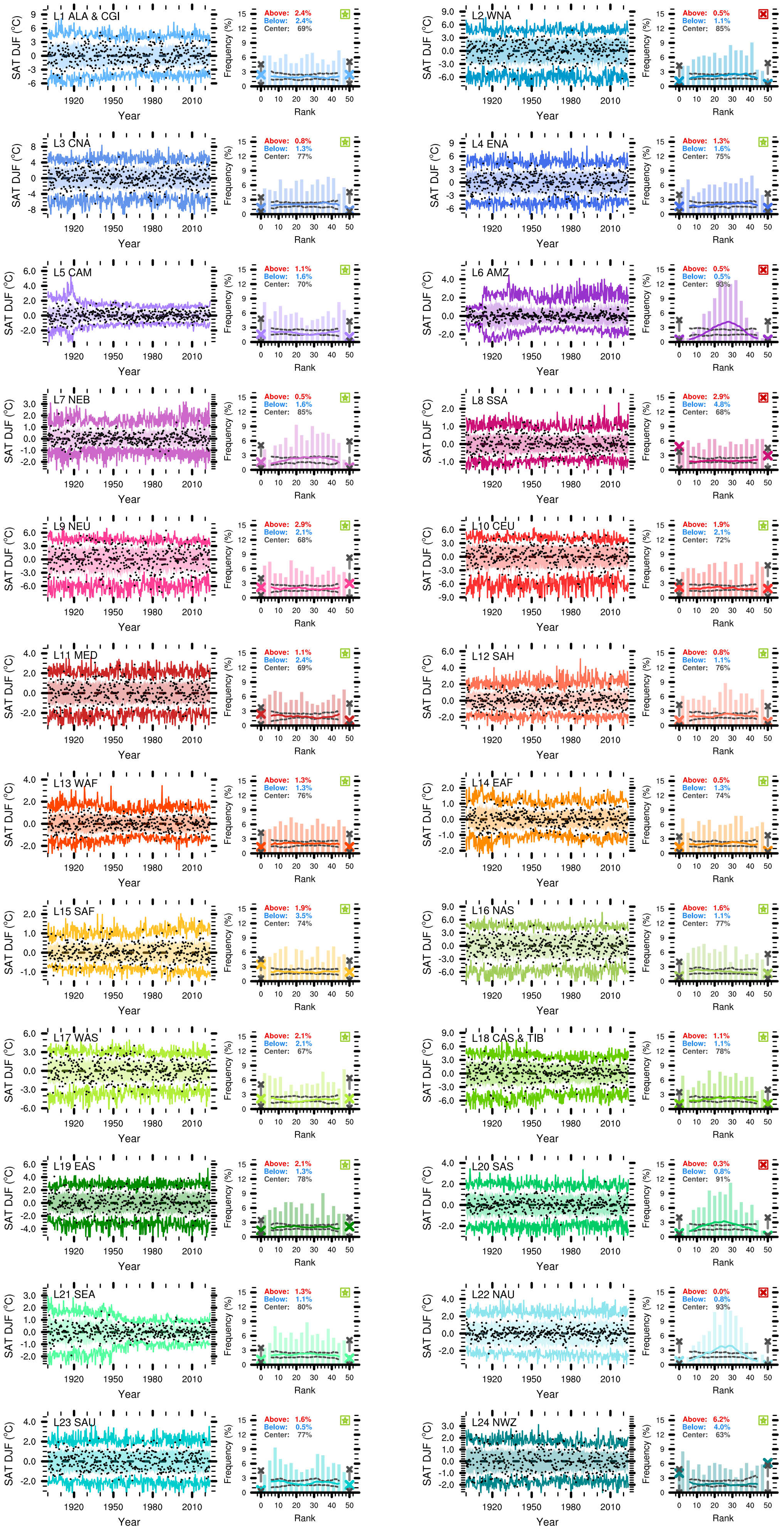

CanESM5 vs GISTEMPv4 TAS JJA

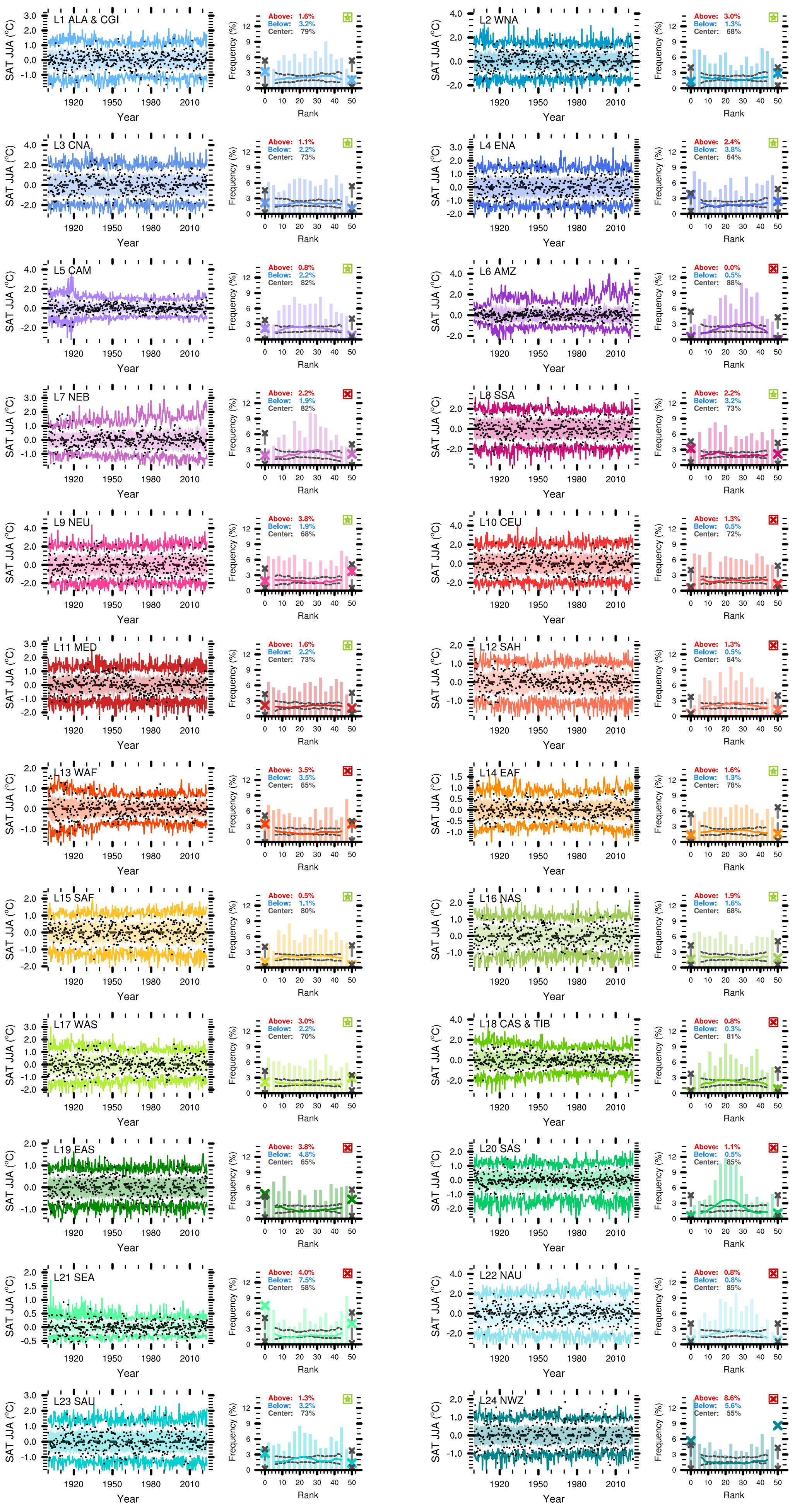

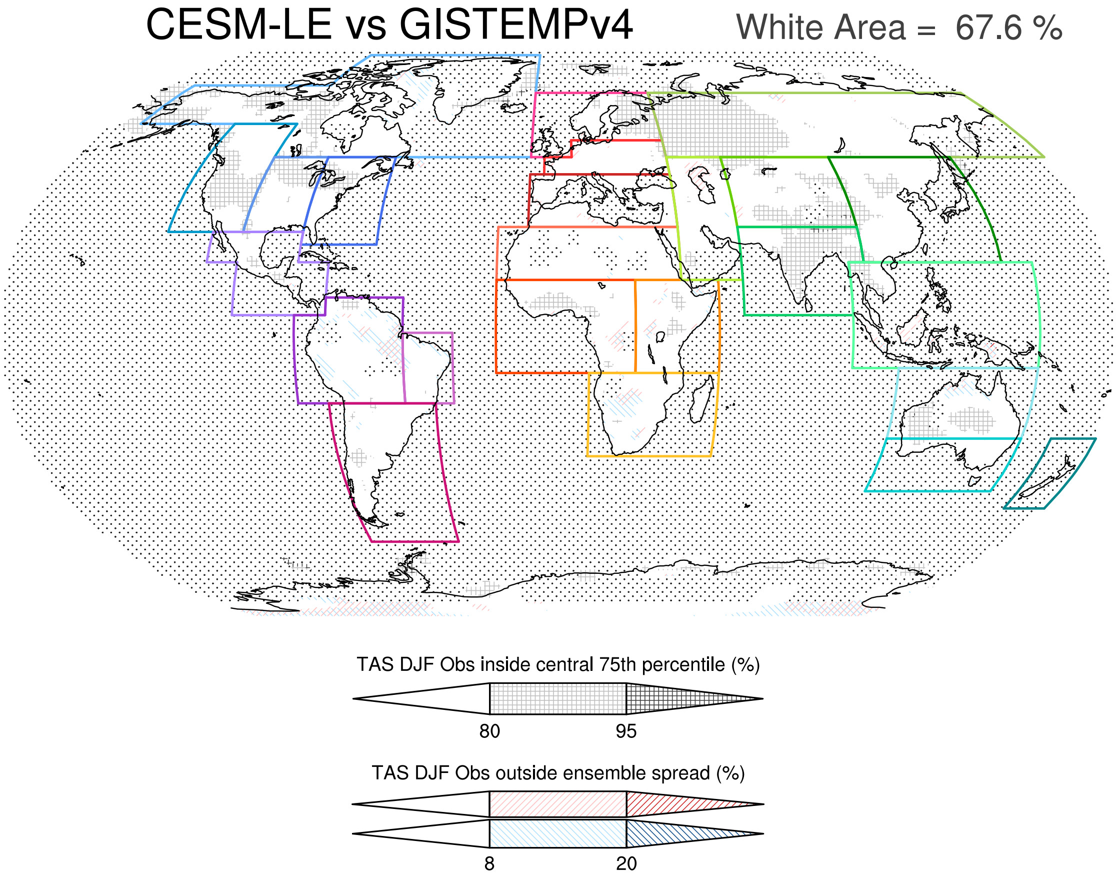

CESM-LE vs GISTEMPv4
White Area = 67.6 %
TAS DJF Obs inside central 75th percentile (%)
80
95
TAS DJF Obs outside ensemble spread (%)
8
20

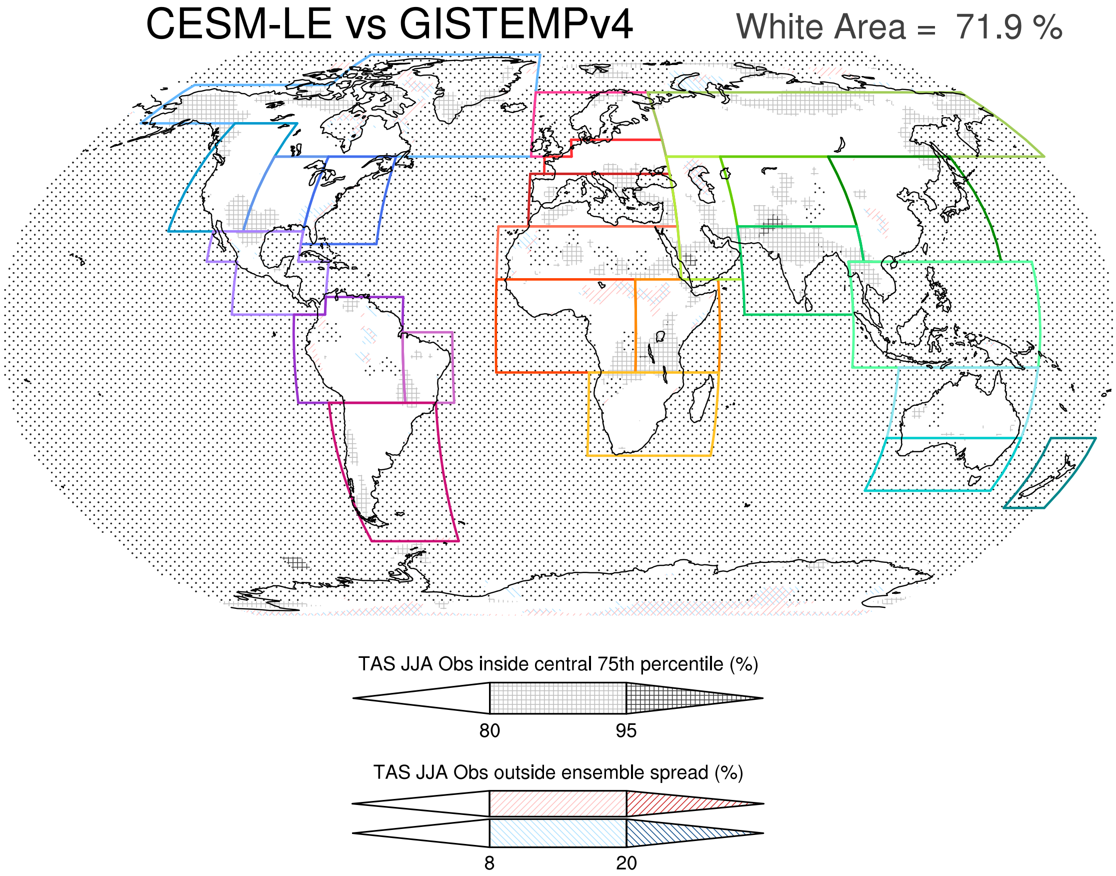

CESM-LE vs GISTEMPv4
White Area = 71.9 %
TAS JJA Obs inside central 75th percentile (%)
80
95
TAS JJA Obs outside ensemble spread (%)
8
20

CESM-LE vs GISTEMPv4 TAS DJF

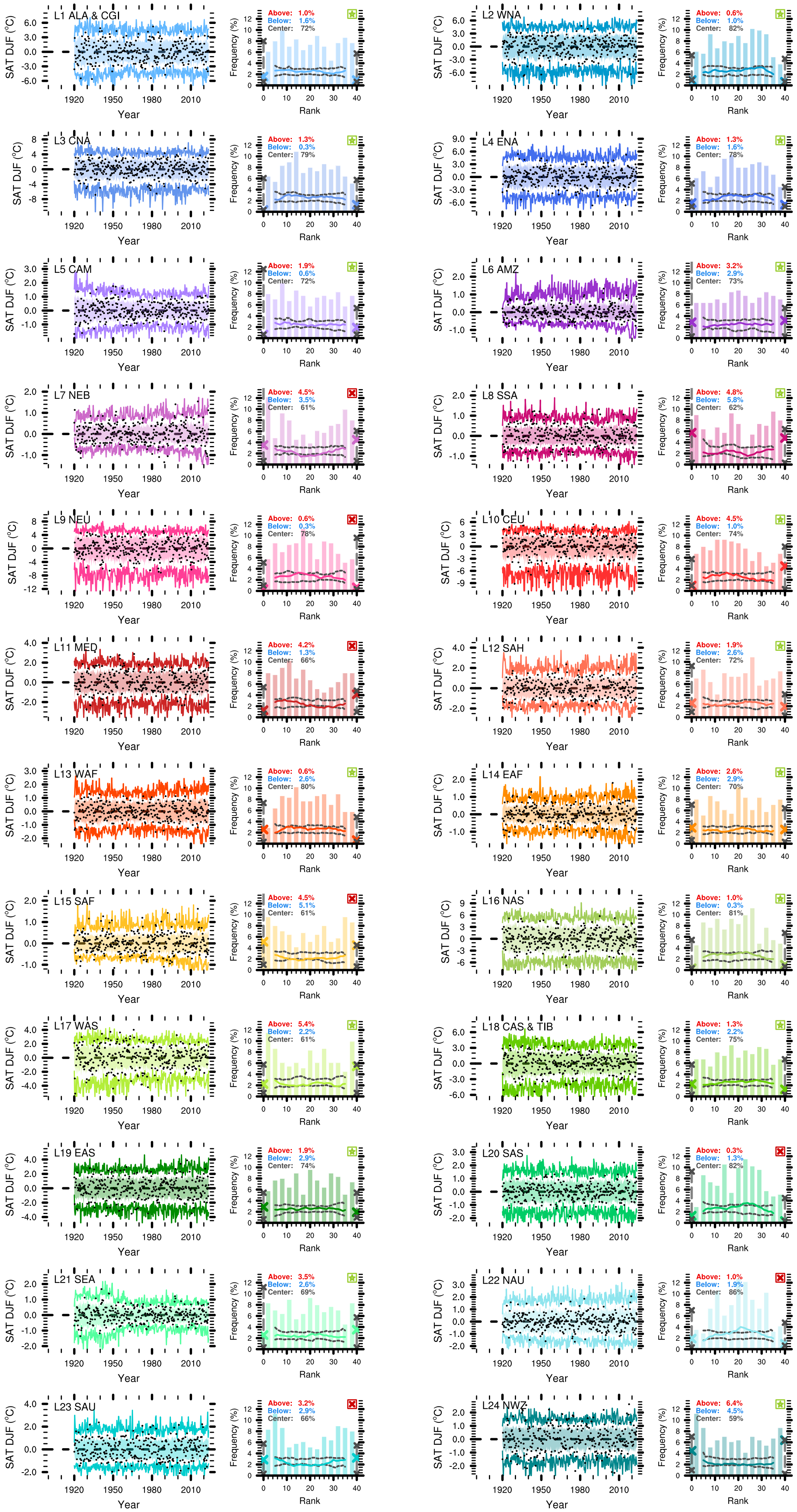

CESM-LE vs GISTEMPv4 TAS JJA

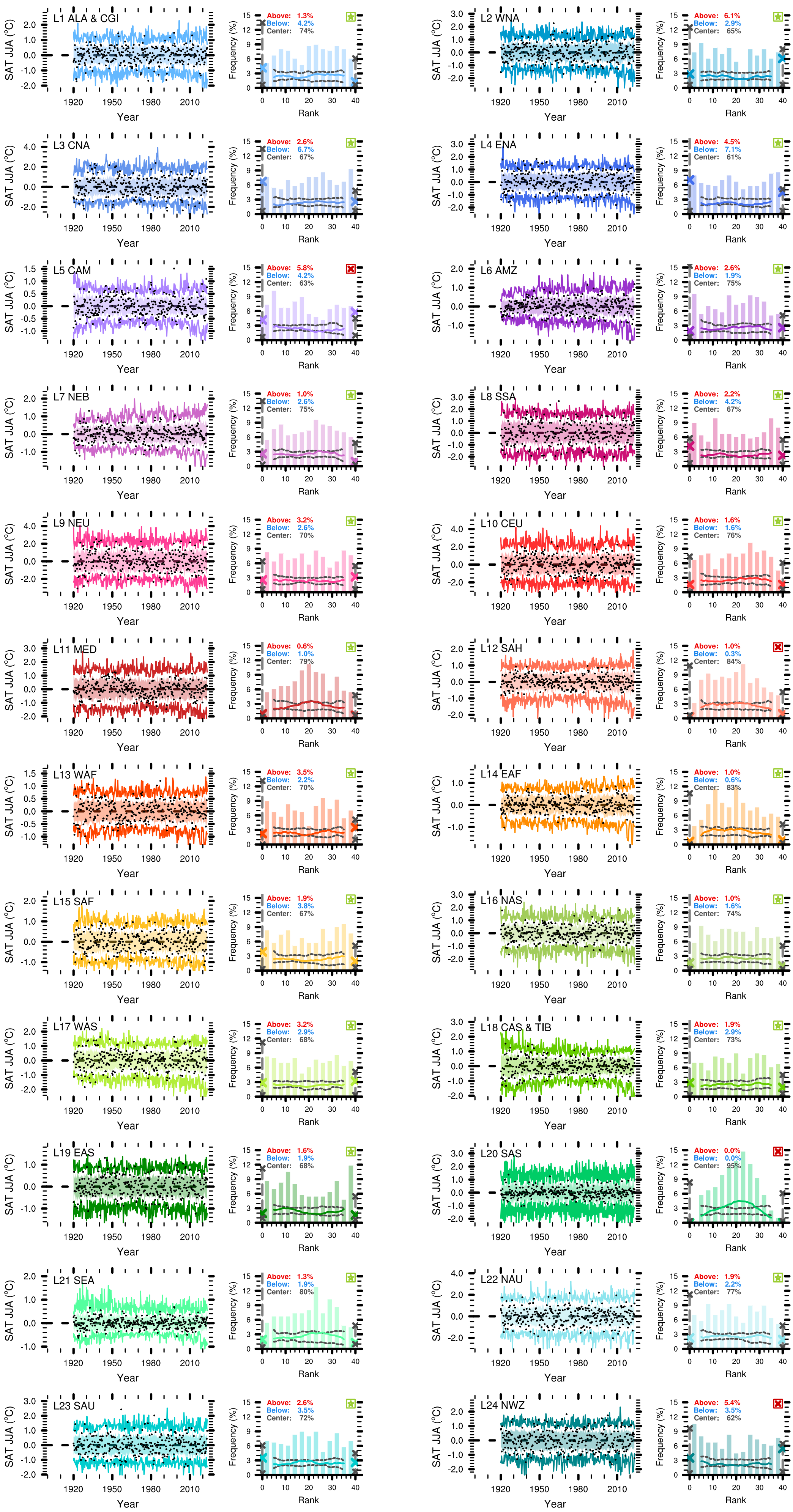

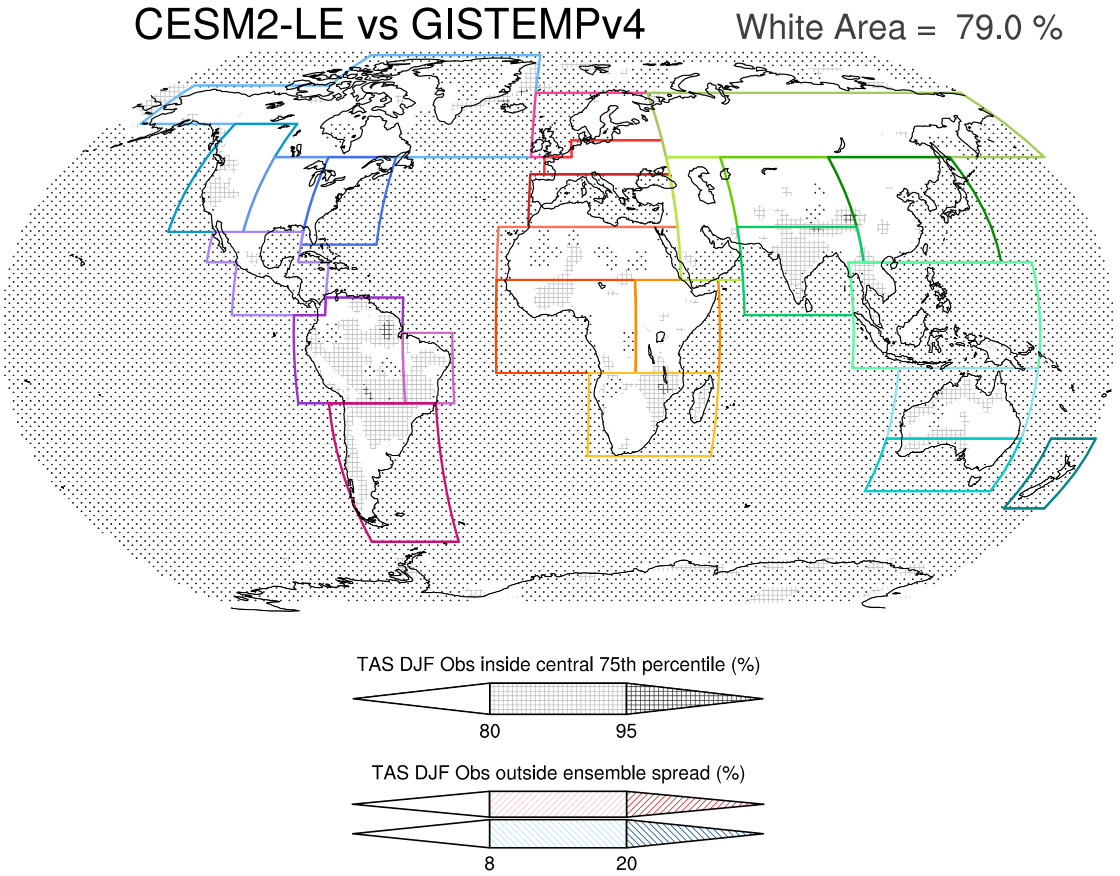
CESM2-LE vs GISTEMPv4
White Area = 79.0 %
TAS DJF Obs inside central 75th percentile (%)
80
95
TAS DJF Obs outside ensemble spread (%)
8
20

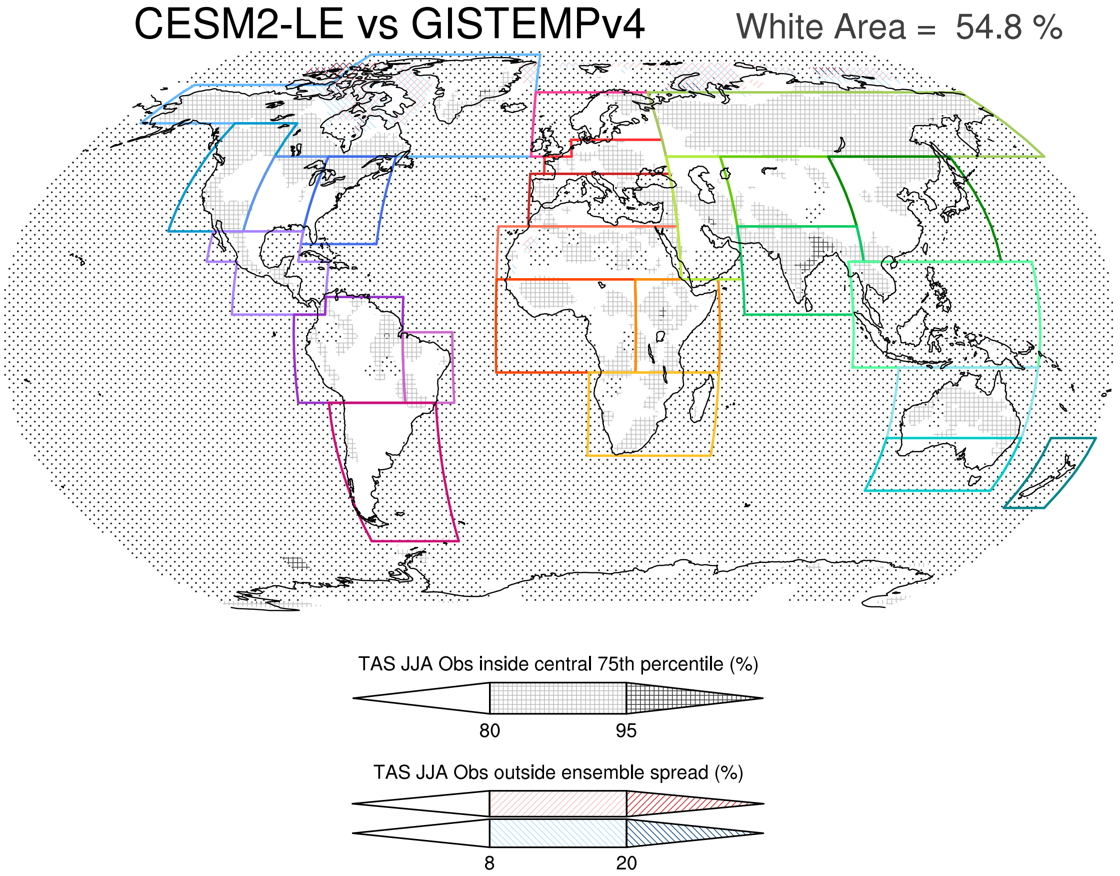
CESM2-LE vs GISTEMPv4
White Area = 54.8 %
TAS JJA Obs inside central 75th percentile (%)
80
95
TAS JJA Obs outside ensemble spread (%)
8
20

CESM2-LE vs GISTEMPv4 TAS DJF

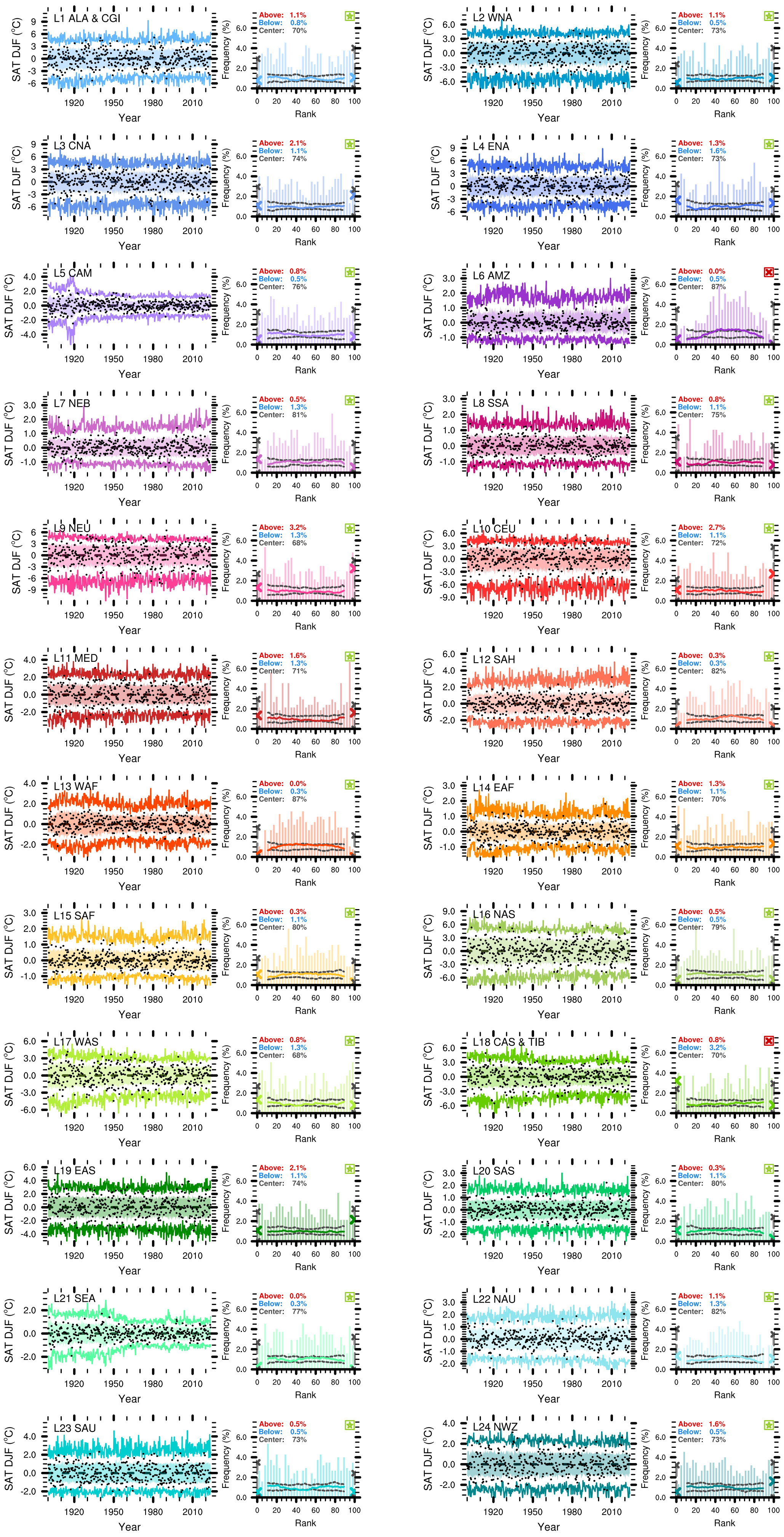

CESM2-LE vs GISTEMPv4 TAS JJA

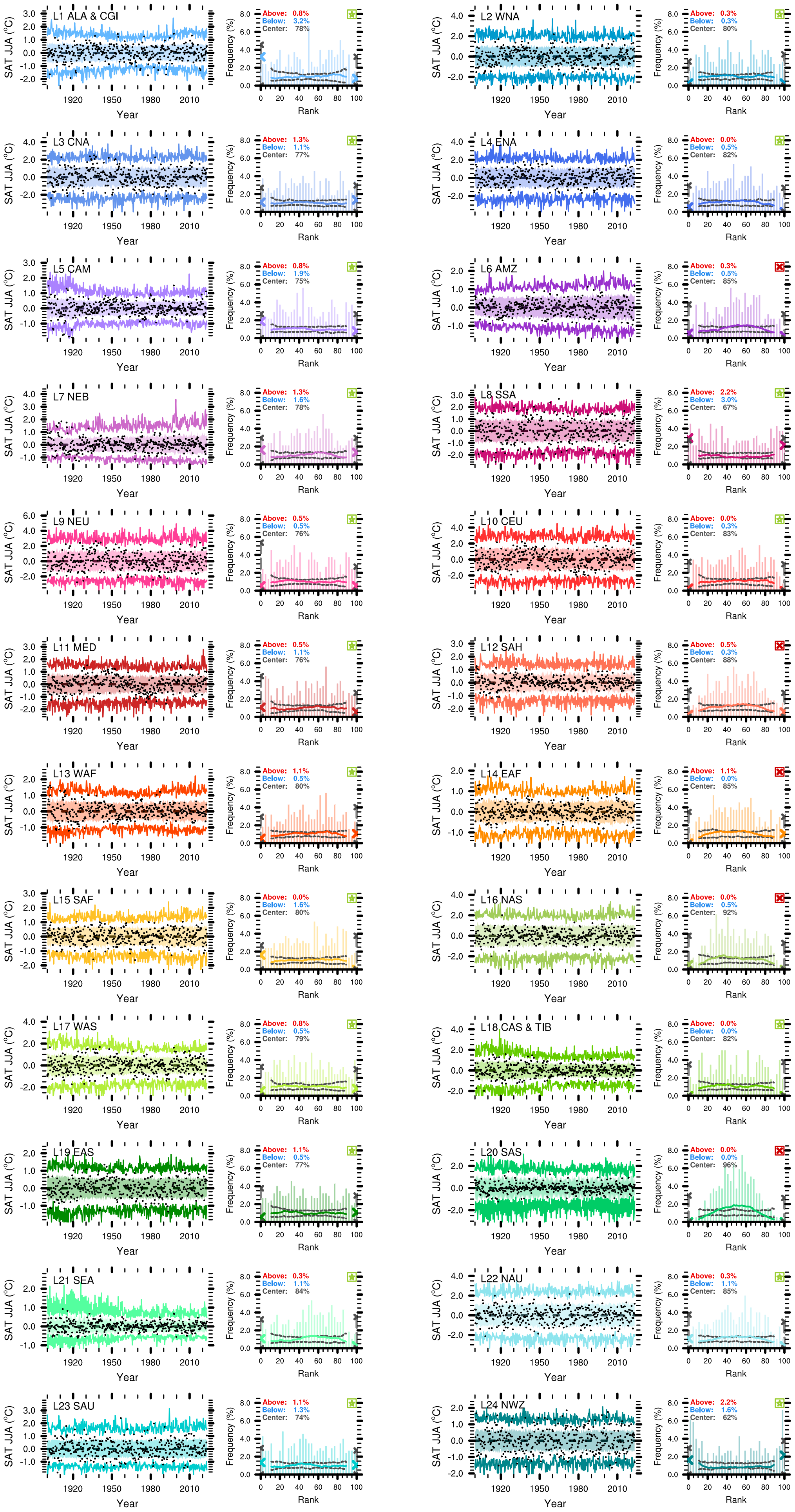

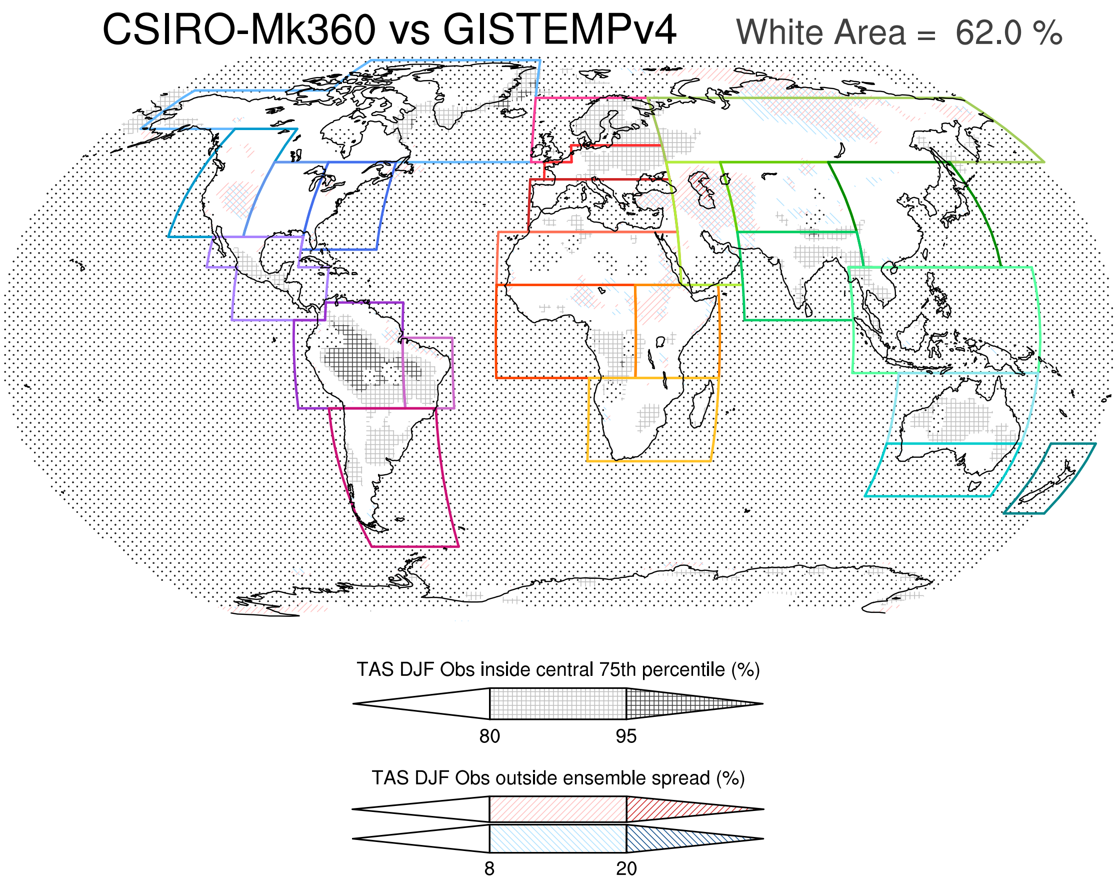
CSIRO-Mk360 vs GISTEMPv4
White Area = 62.0 %
TAS DJF Obs inside central 75th percentile (%)
80
95
TAS DJF Obs outside ensemble spread (%)
8
20

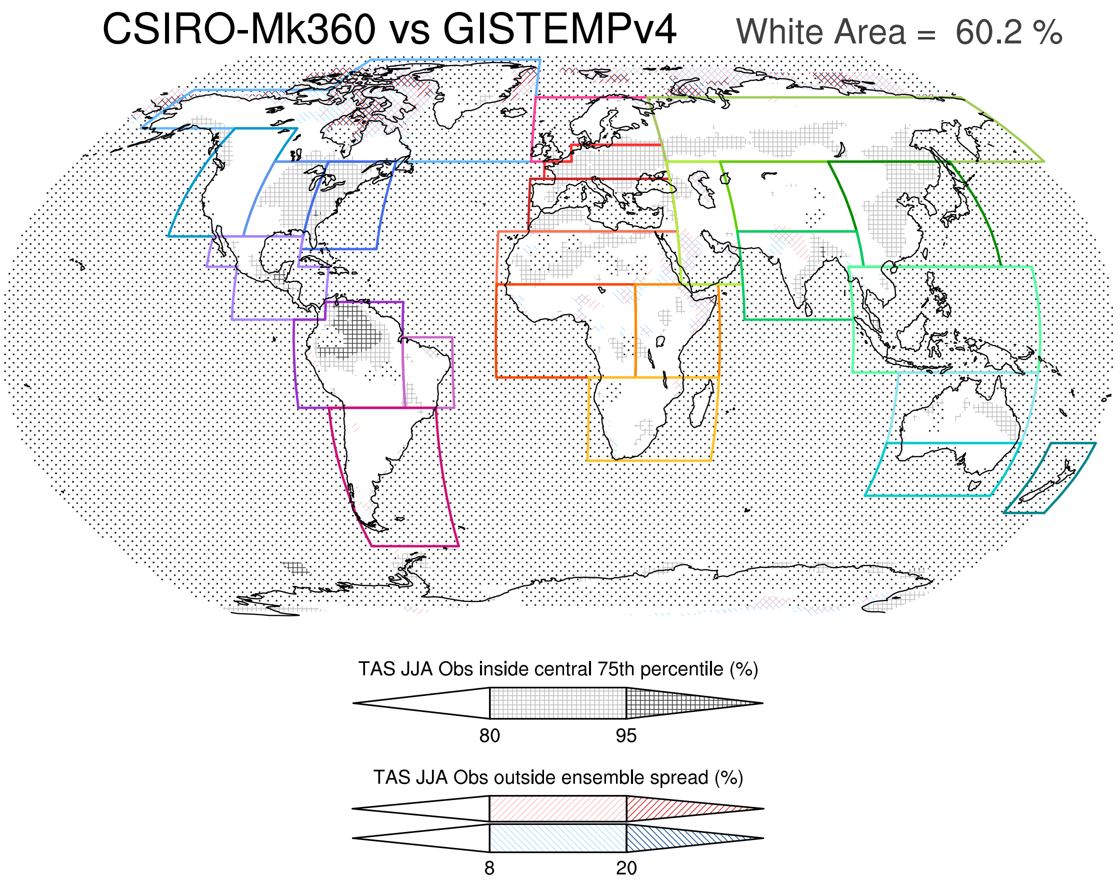
CSIRO-Mk360 vs GISTEMPv4
White Area = 60.2 %
TAS JJA Obs inside central 75th percentile (%)
80
95
TAS JJA Obs outside ensemble spread (%)
8
20

CSIRO-Mk360 vs GISTEMPv4 TAS DJF

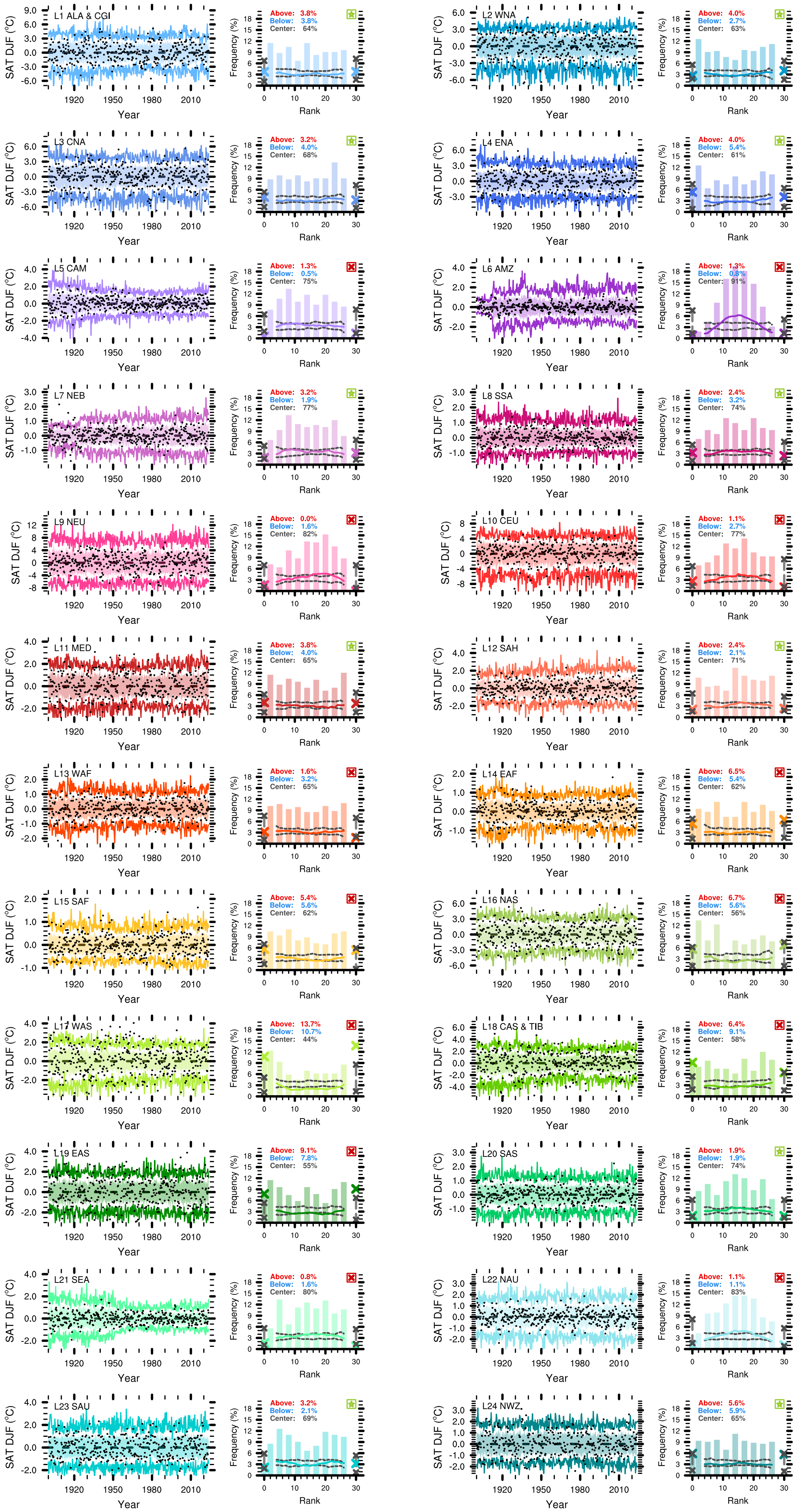

CSIRO-Mk360 vs GISTEMPv4 TAS JJA

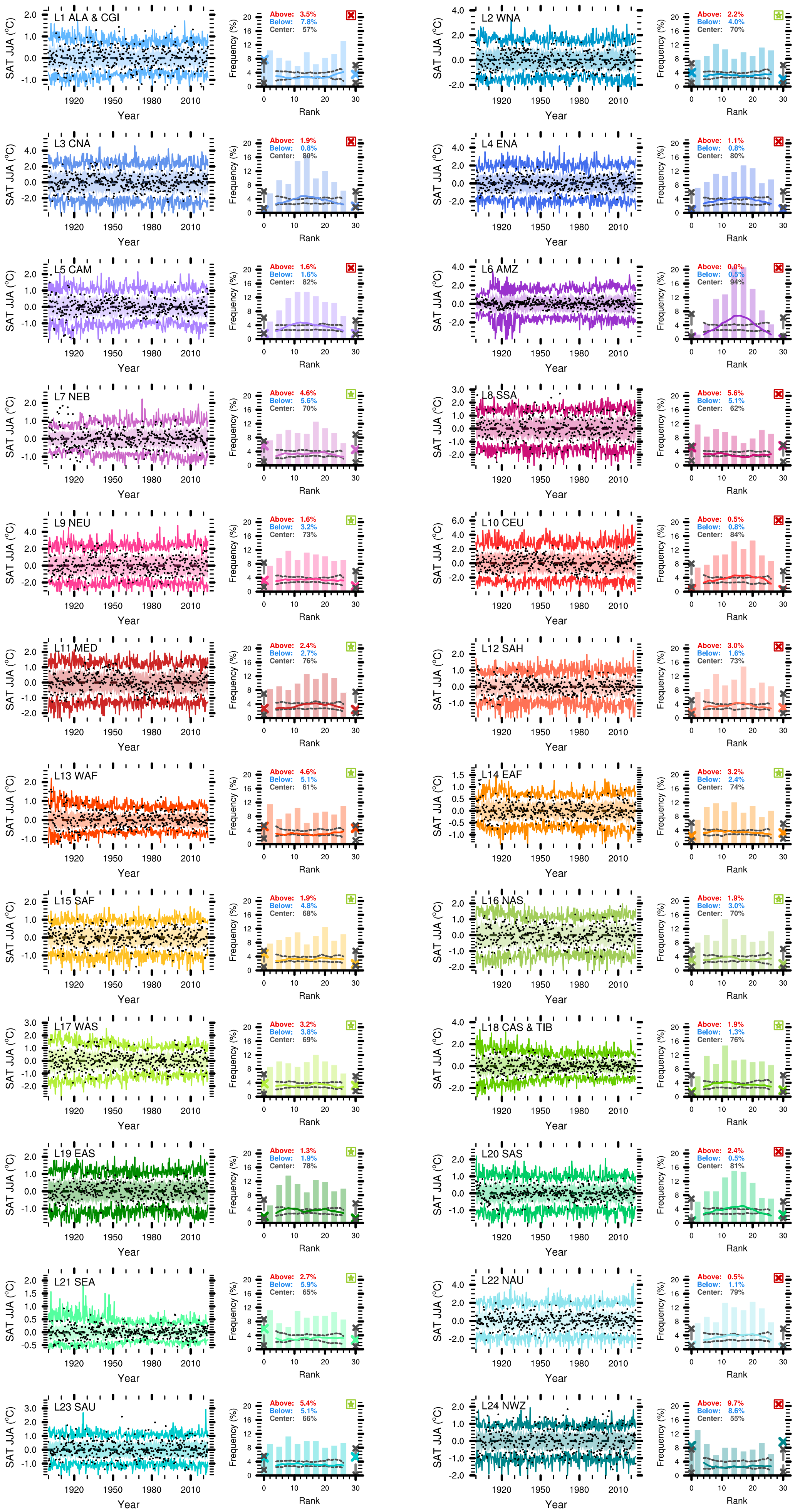

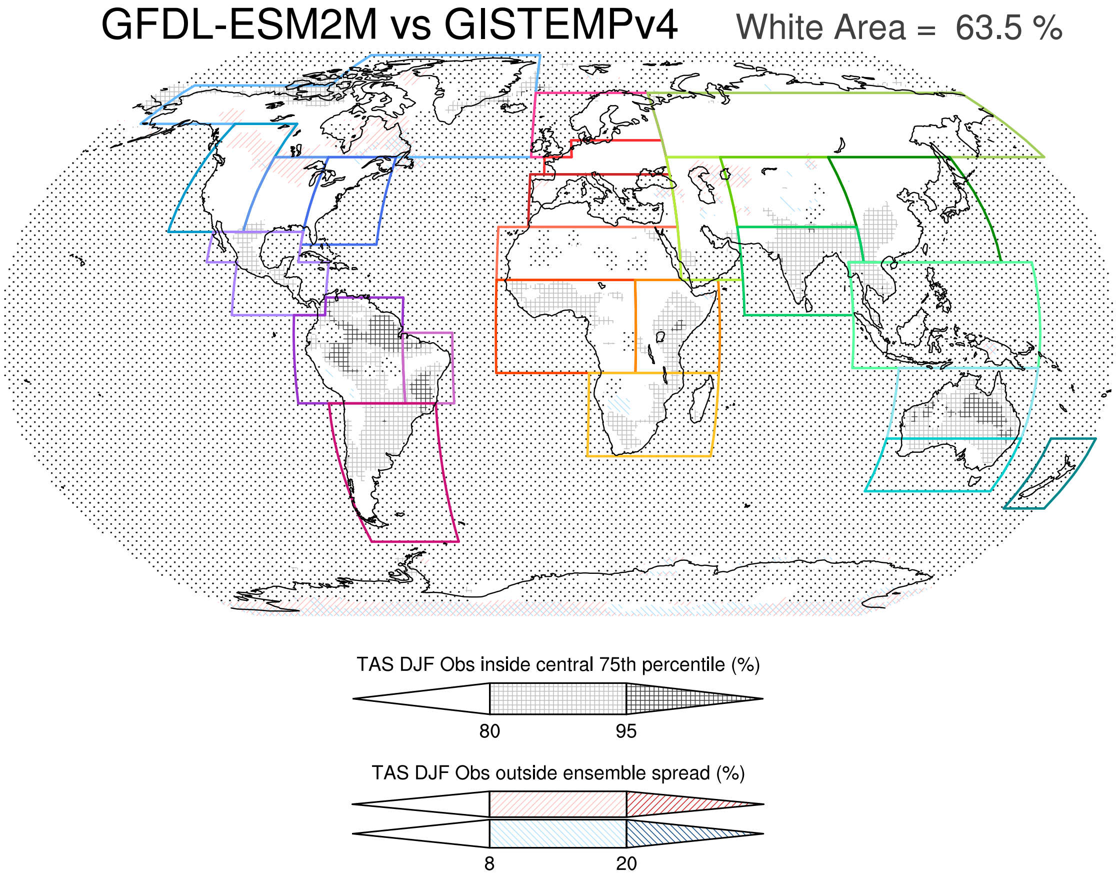

GFDL-ESM2M vs GISTEMPv4
White Area = 63.5 %
TAS DJF Obs inside central 75th percentile (%)
80
95
TAS DJF Obs outside ensemble spread (%)
8
20

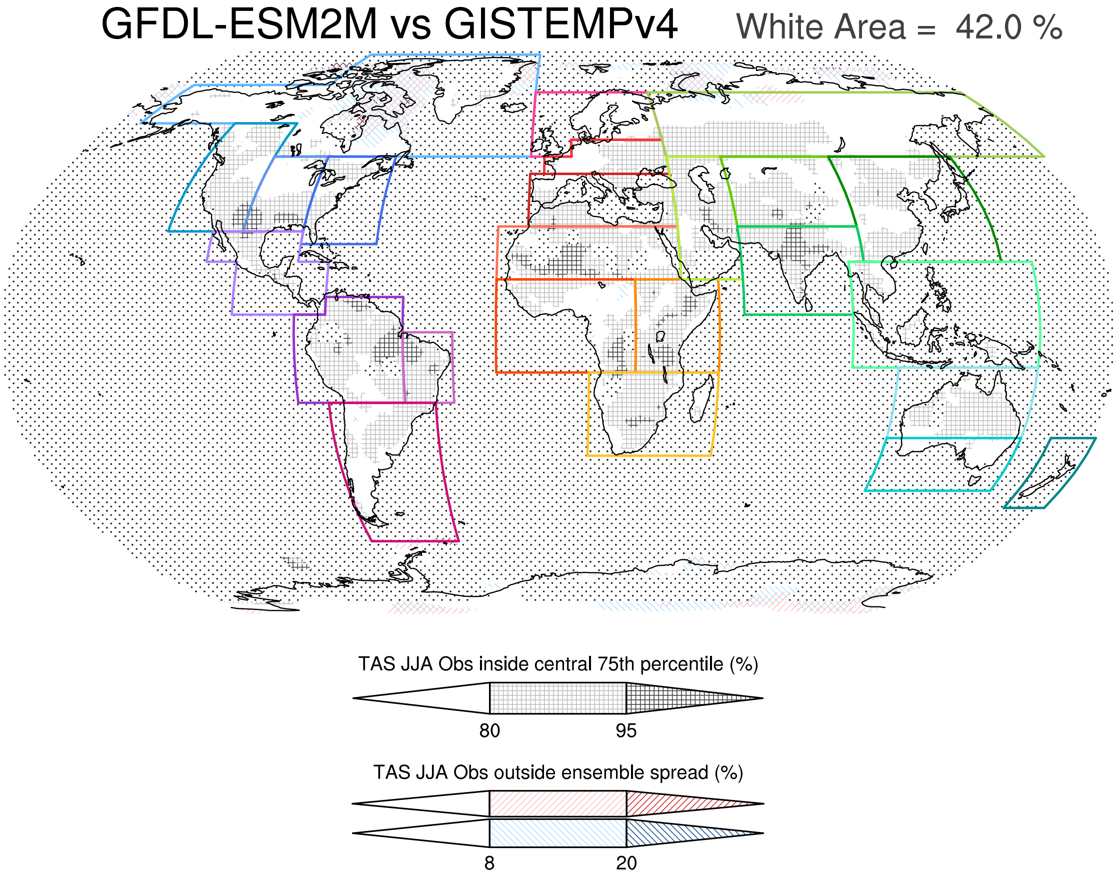

GFDL-ESM2M vs GISTEMPv4
White Area = 42.0 %
TAS JJA Obs inside central 75th percentile (%)
80
95
TAS JJA Obs outside ensemble spread (%)
8
20

GFDL-ESM2M vs GISTEMPv4 TAS DJF

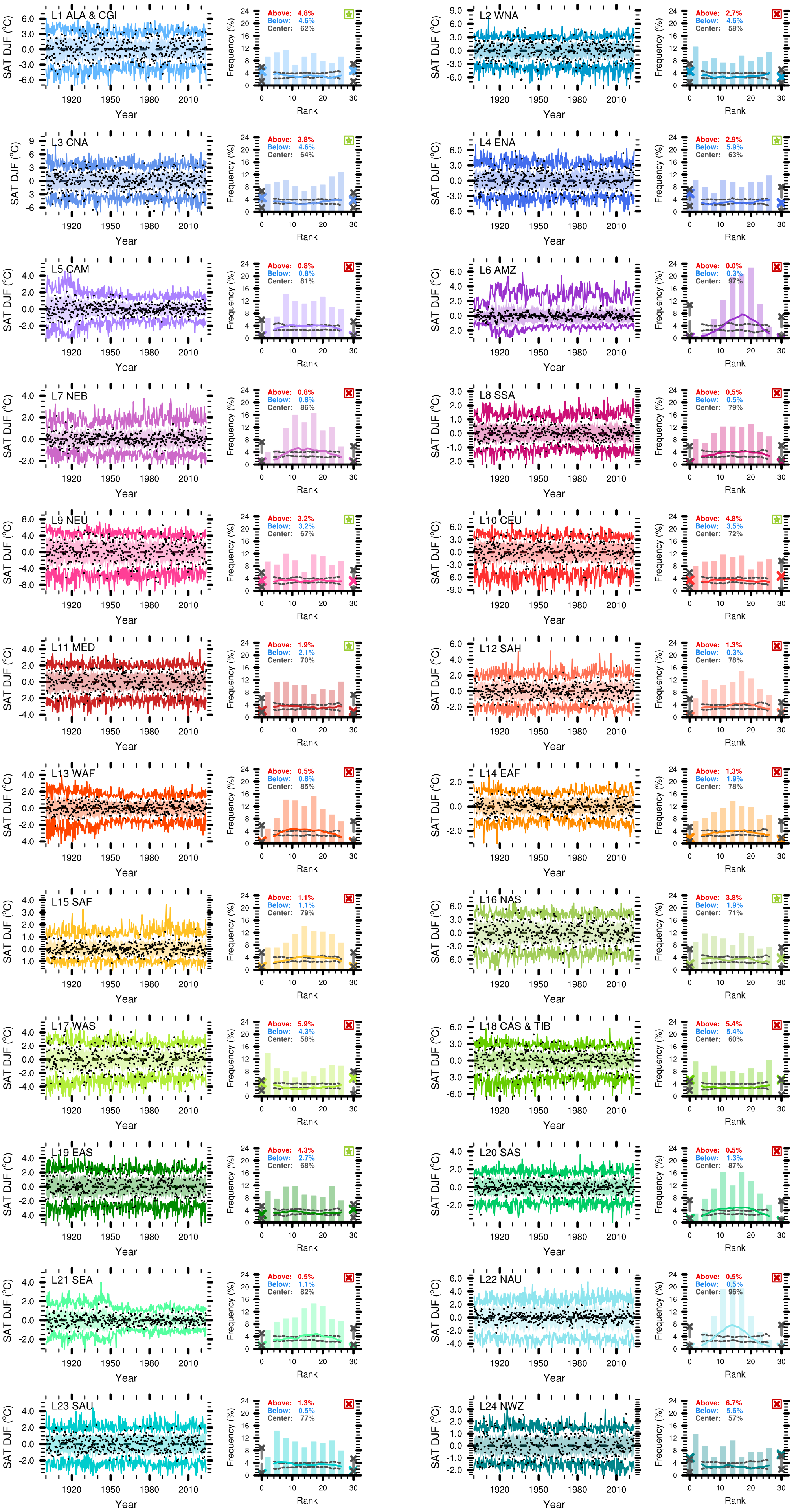

GFDL-ESM2M vs GISTEMPv4 TAS JJA

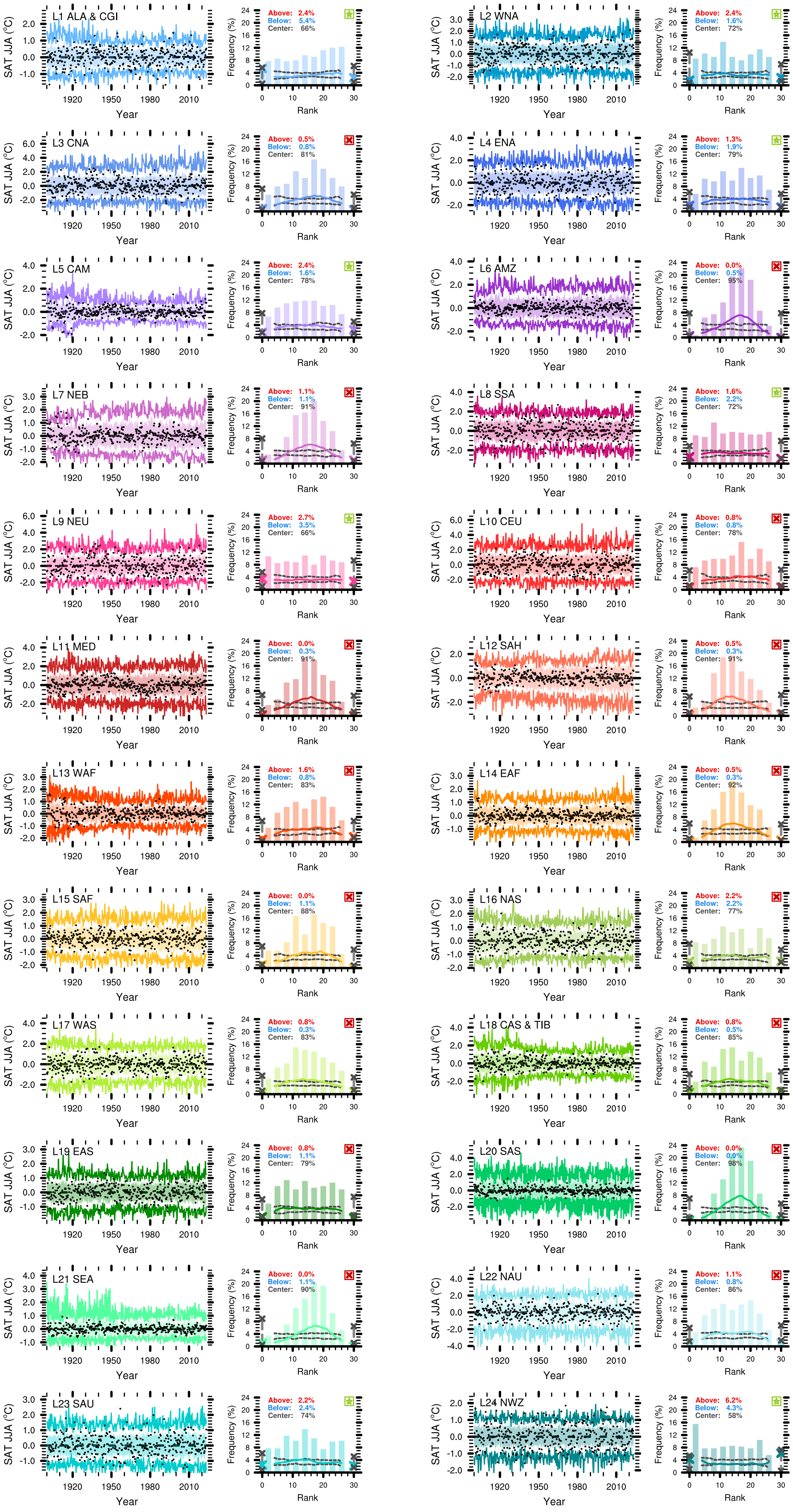

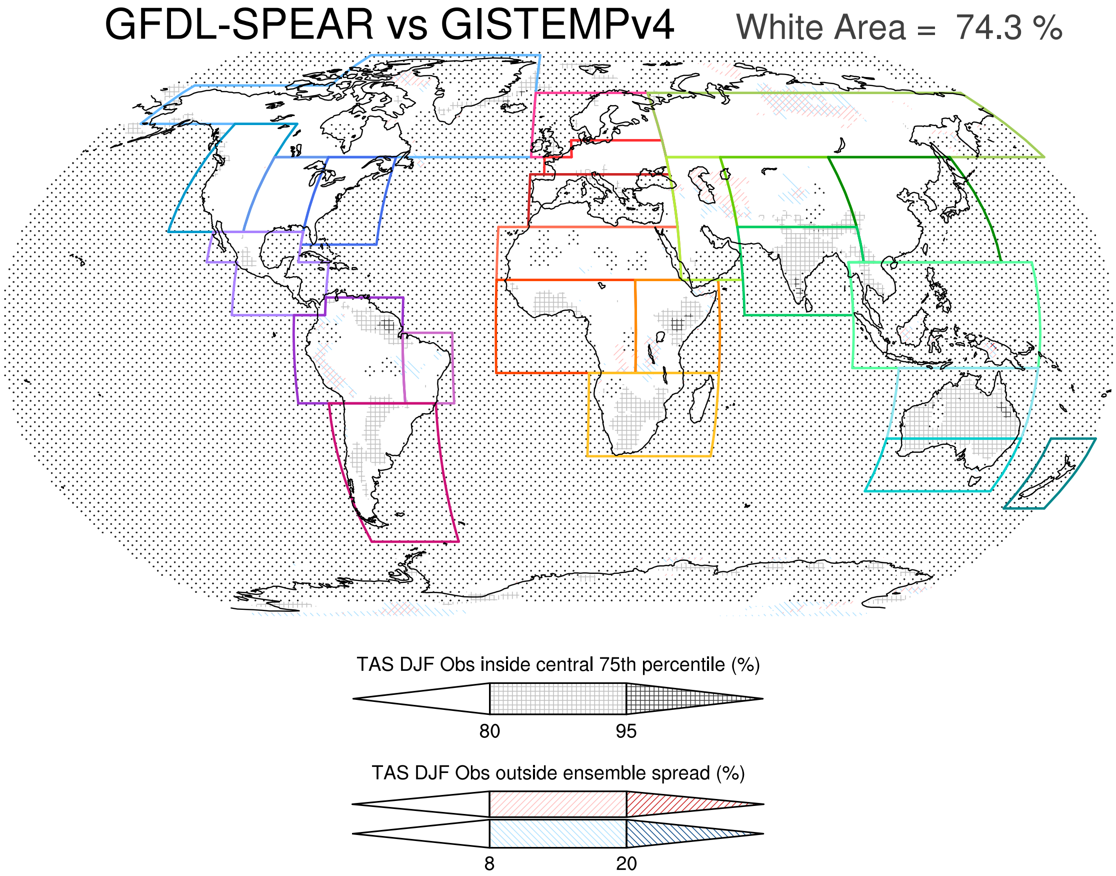
GFDL-SPEAR vs GISTEMPv4
White Area = 74.3 %
TAS DJF Obs inside central 75th percentile (%)
80
95
TAS DJF Obs outside ensemble spread (%)
8
20

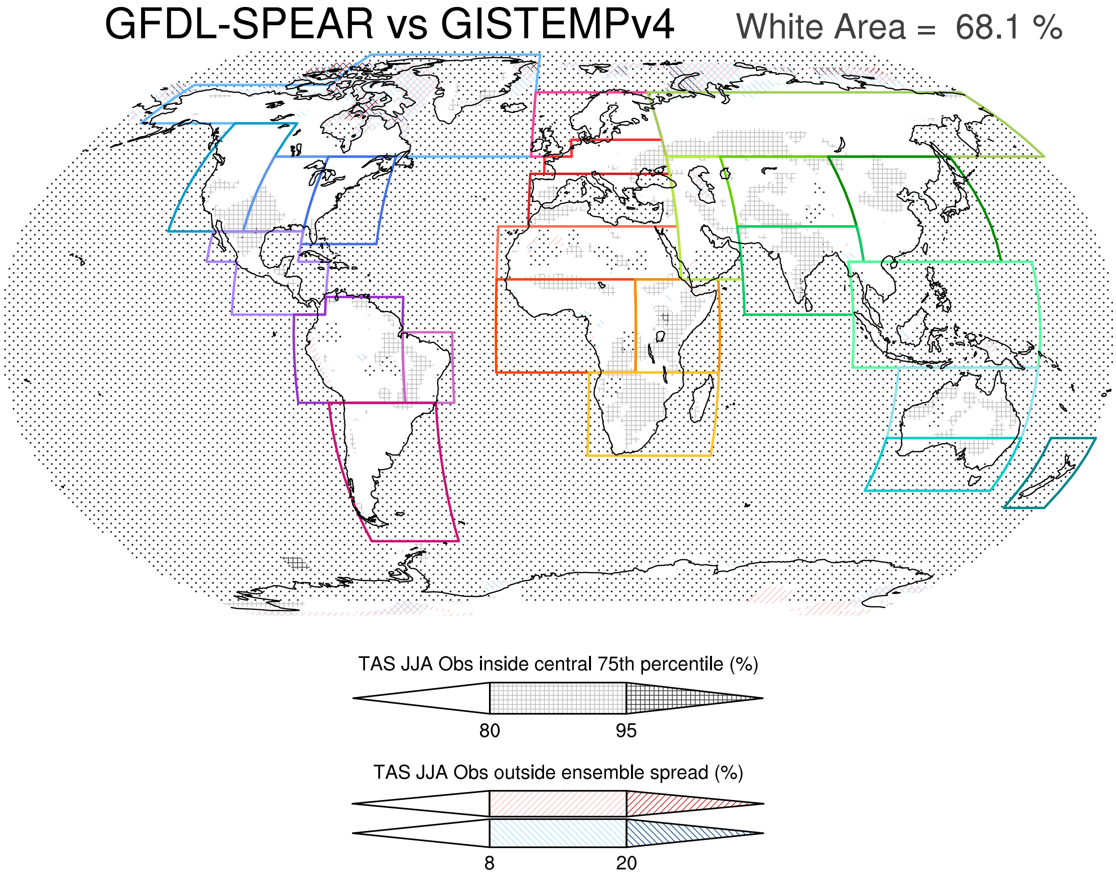
GFDL-SPEAR vs GISTEMPv4
White Area = 68.1 %
TAS JJA Obs inside central 75th percentile (%)
80
95
TAS JJA Obs outside ensemble spread (%)
8
20

GFDL-SPEAR vs GISTEMPv4 TAS DJF

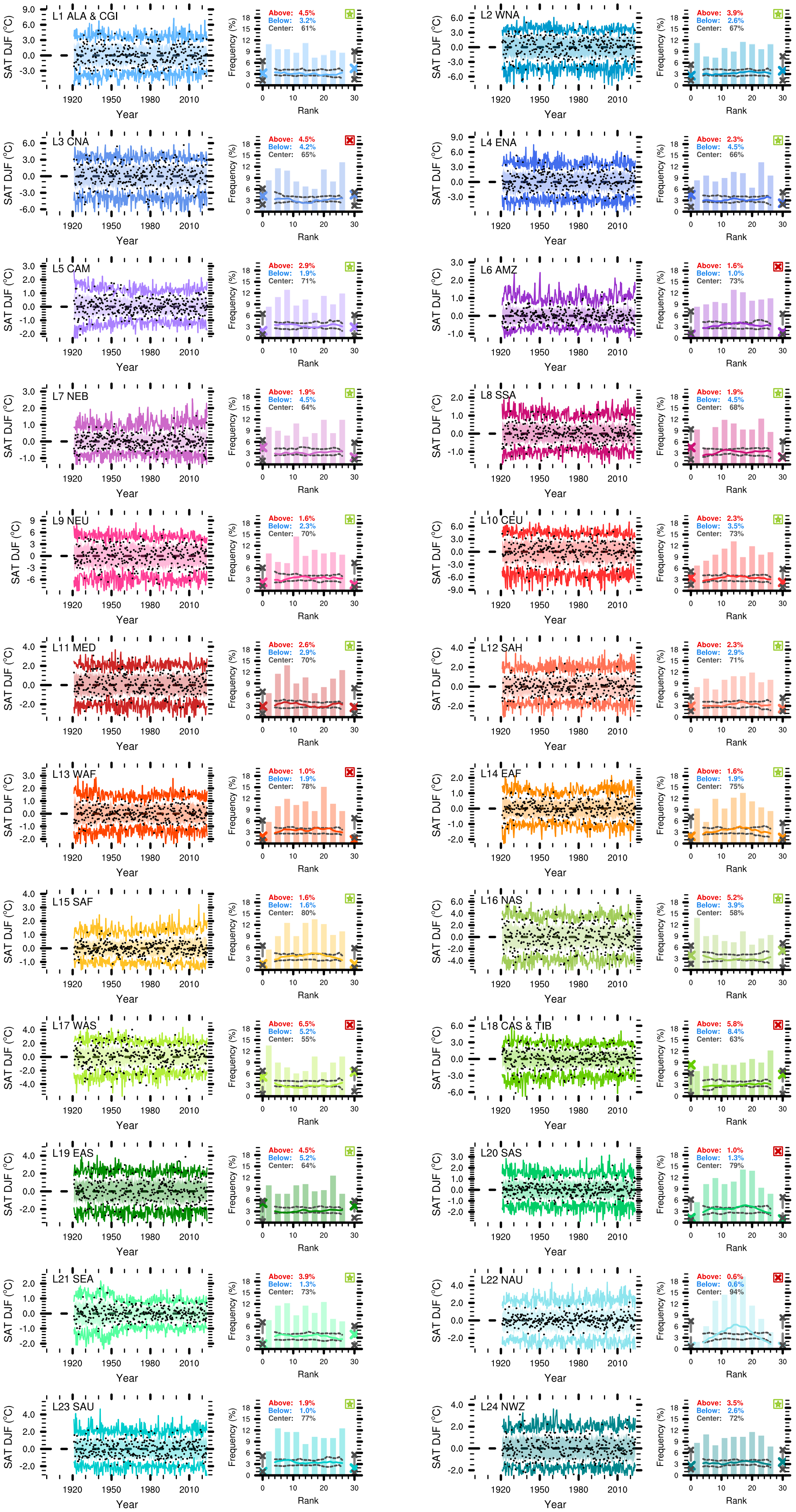

GFDL-SPEAR vs GISTEMPv4 TAS JJA

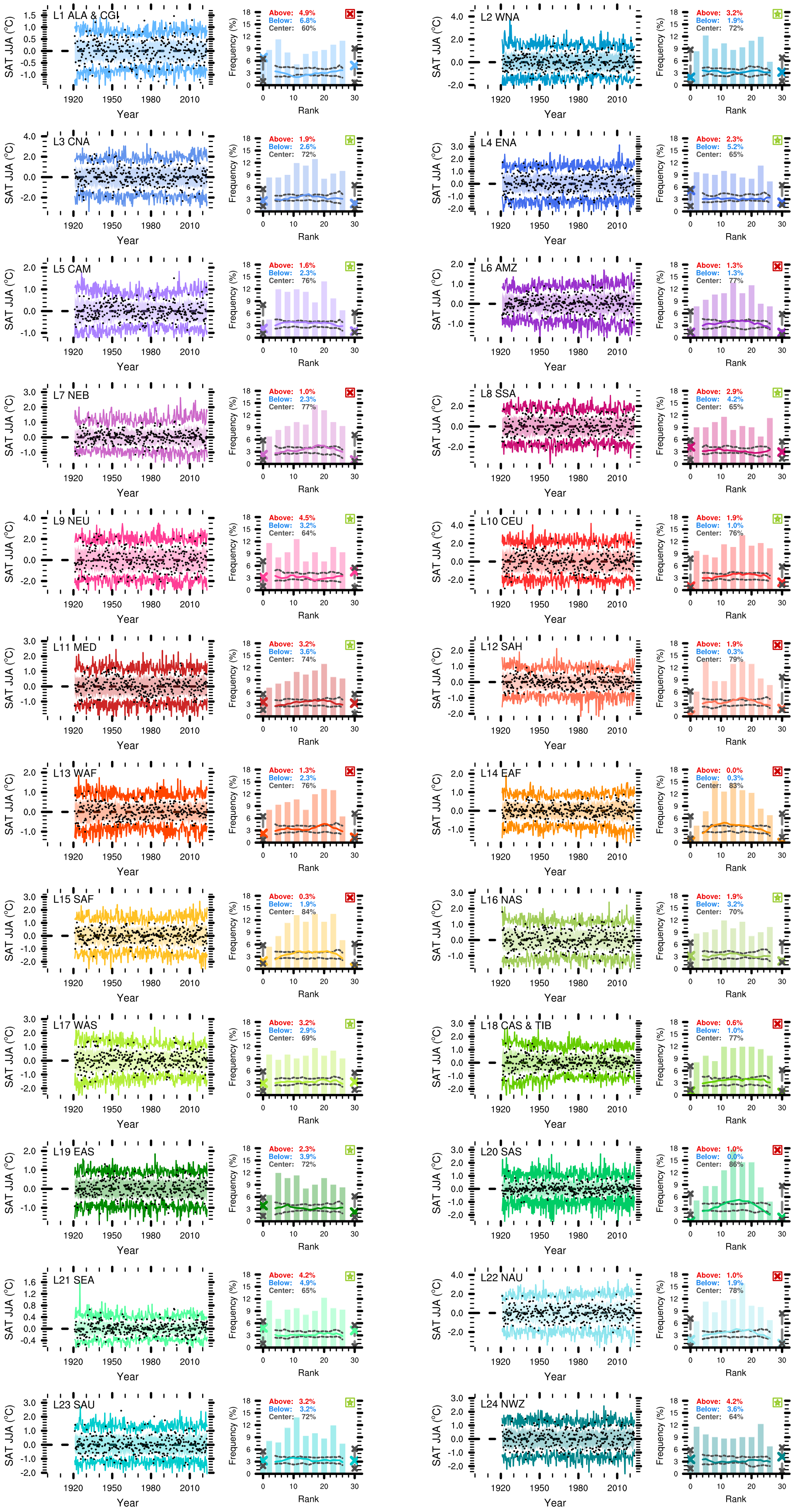

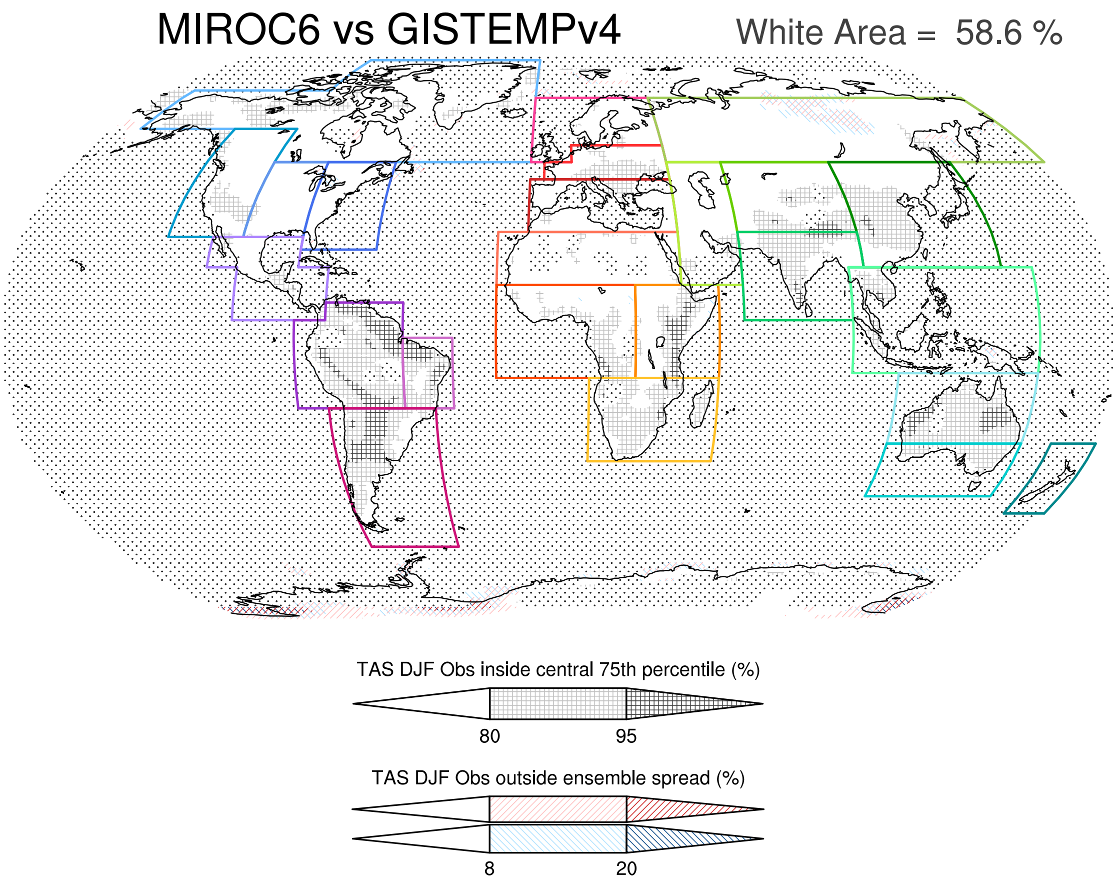
MIROC6 vs GISTEMPv4
White Area = 58.6 %
TAS DJF Obs inside central 75th percentile (%)
80
95
TAS DJF Obs outside ensemble spread (%)
8
20

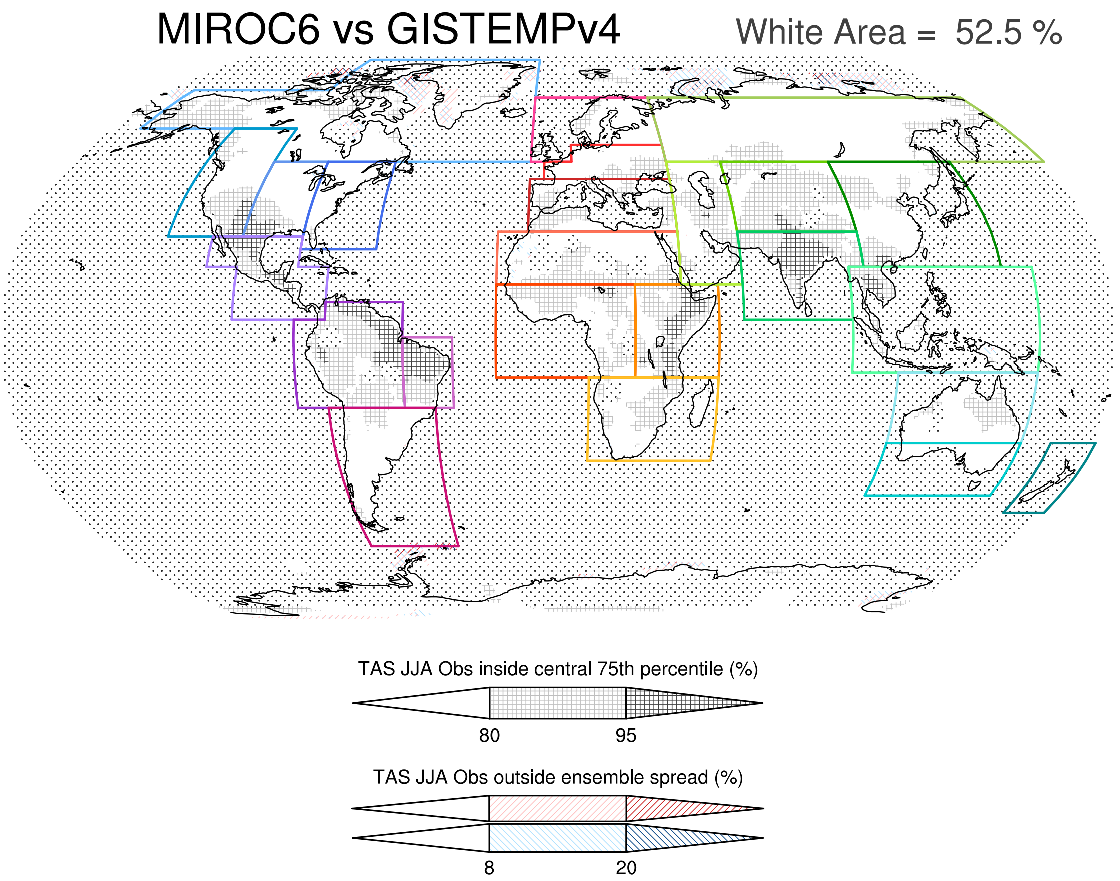
MIROC6 vs GISTEMPv4
White Area = 52.5 %
TAS JJA Obs inside central 75th percentile (%)
80
95
TAS JJA Obs outside ensemble spread (%)
8
20

MIROC6 vs GISTEMPv4 TAS DJF

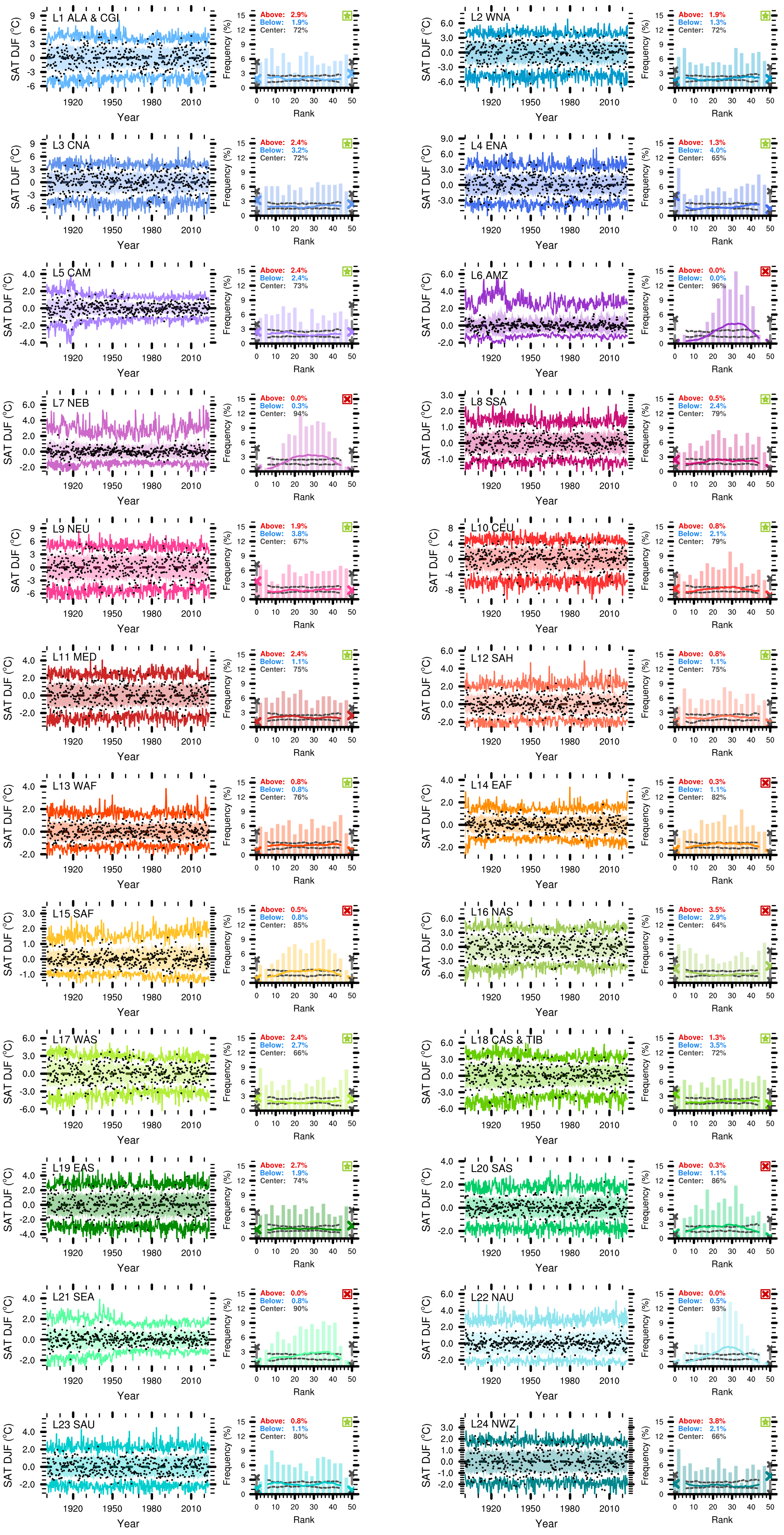

MIROC6 vs GISTEMPv4 TAS JJA

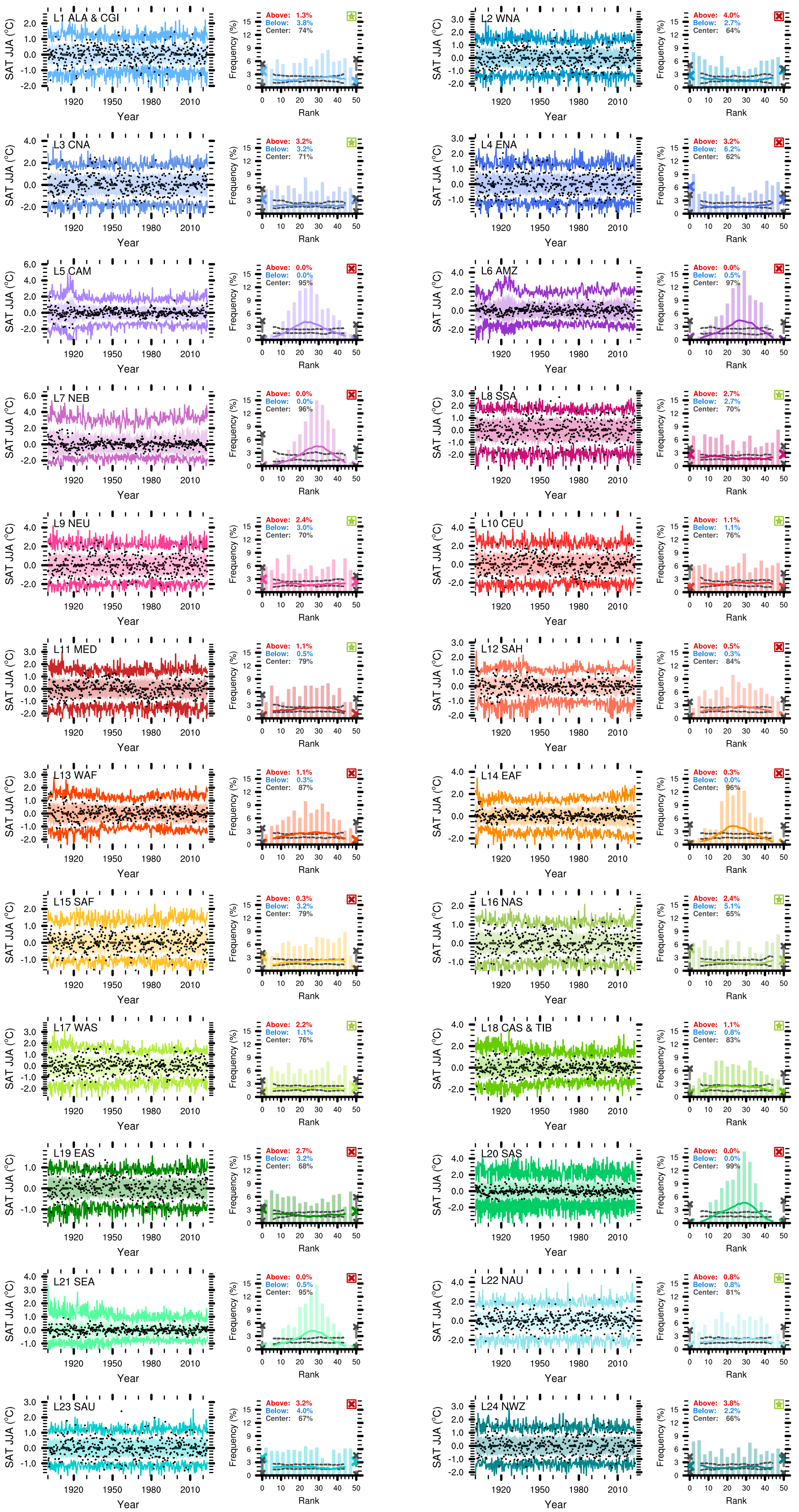

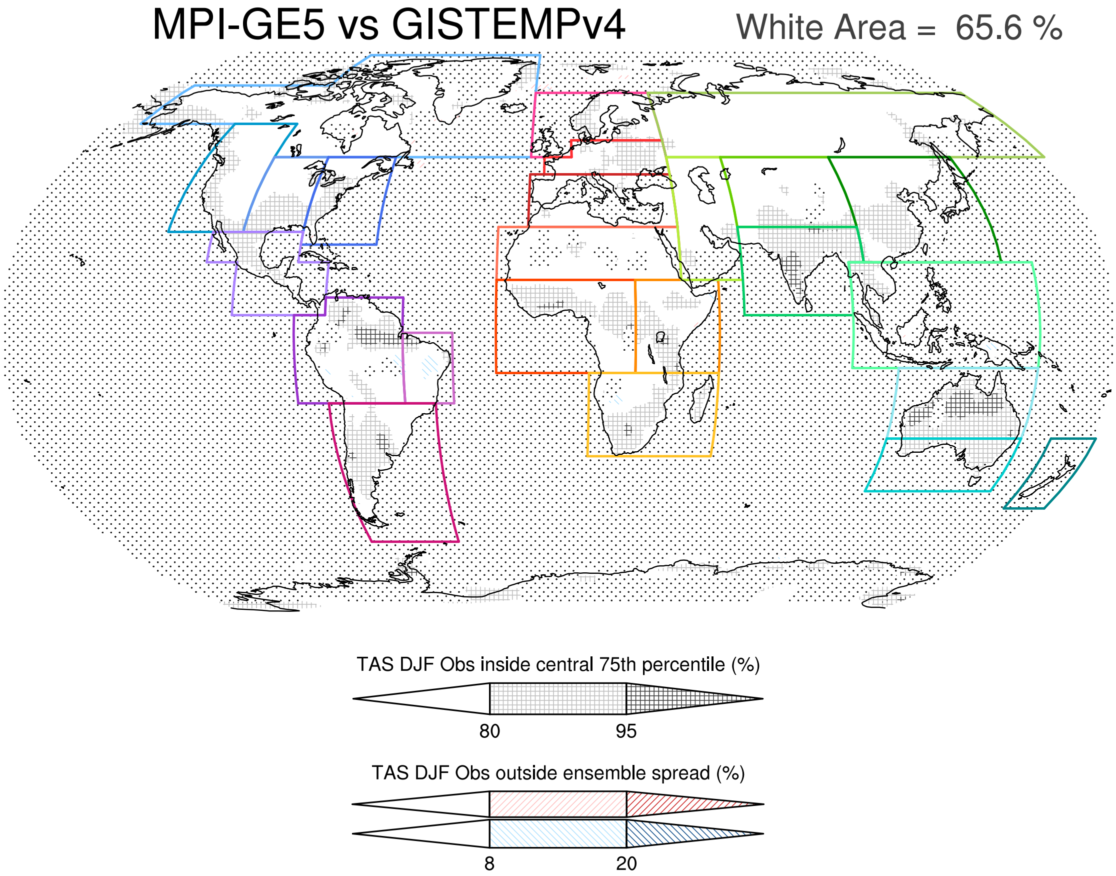
MPI-GE5 vs GISTEMPv4
White Area = 65.6 %
TAS DJF Obs inside central 75th percentile (%)
80
95
TAS DJF Obs outside ensemble spread (%)
8
20

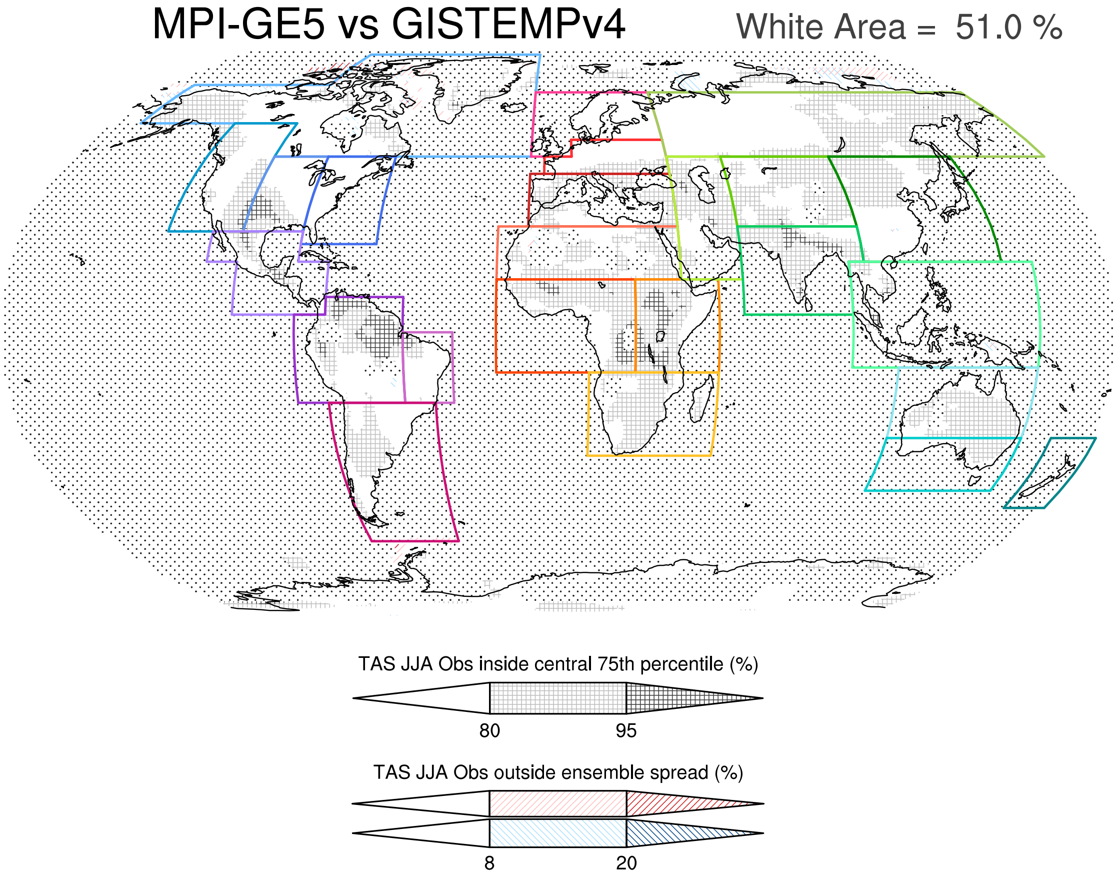
MPI-GE5 vs GISTEMPv4
White Area = 51.0 %
TAS JJA Obs inside central 75th percentile (%)
80
95
TAS JJA Obs outside ensemble spread (%)
8
20

MPI-GE5 vs GISTEMPv4 TAS DJF

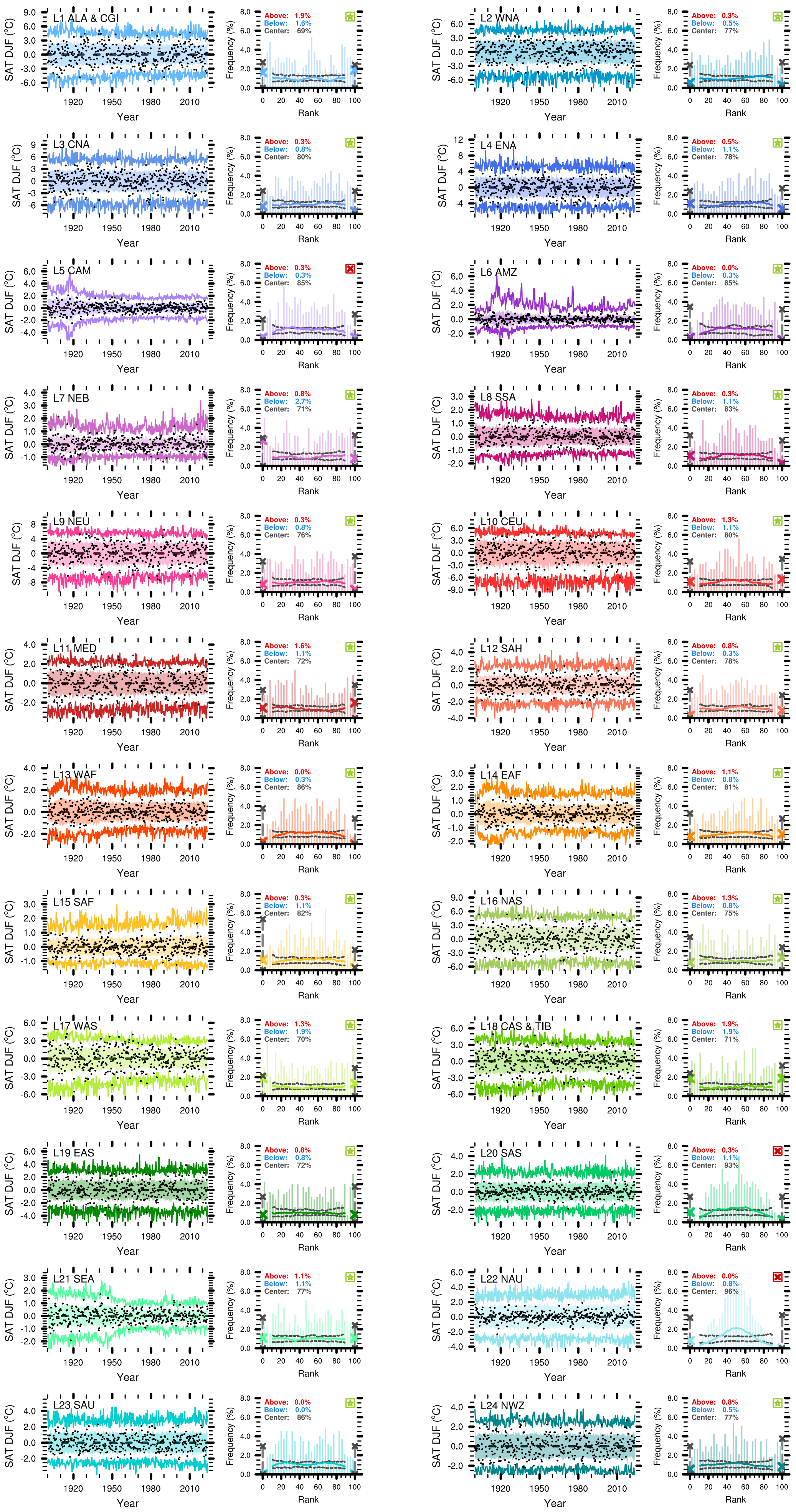

MPI-GE5 vs GISTEMPv4 TAS JJA

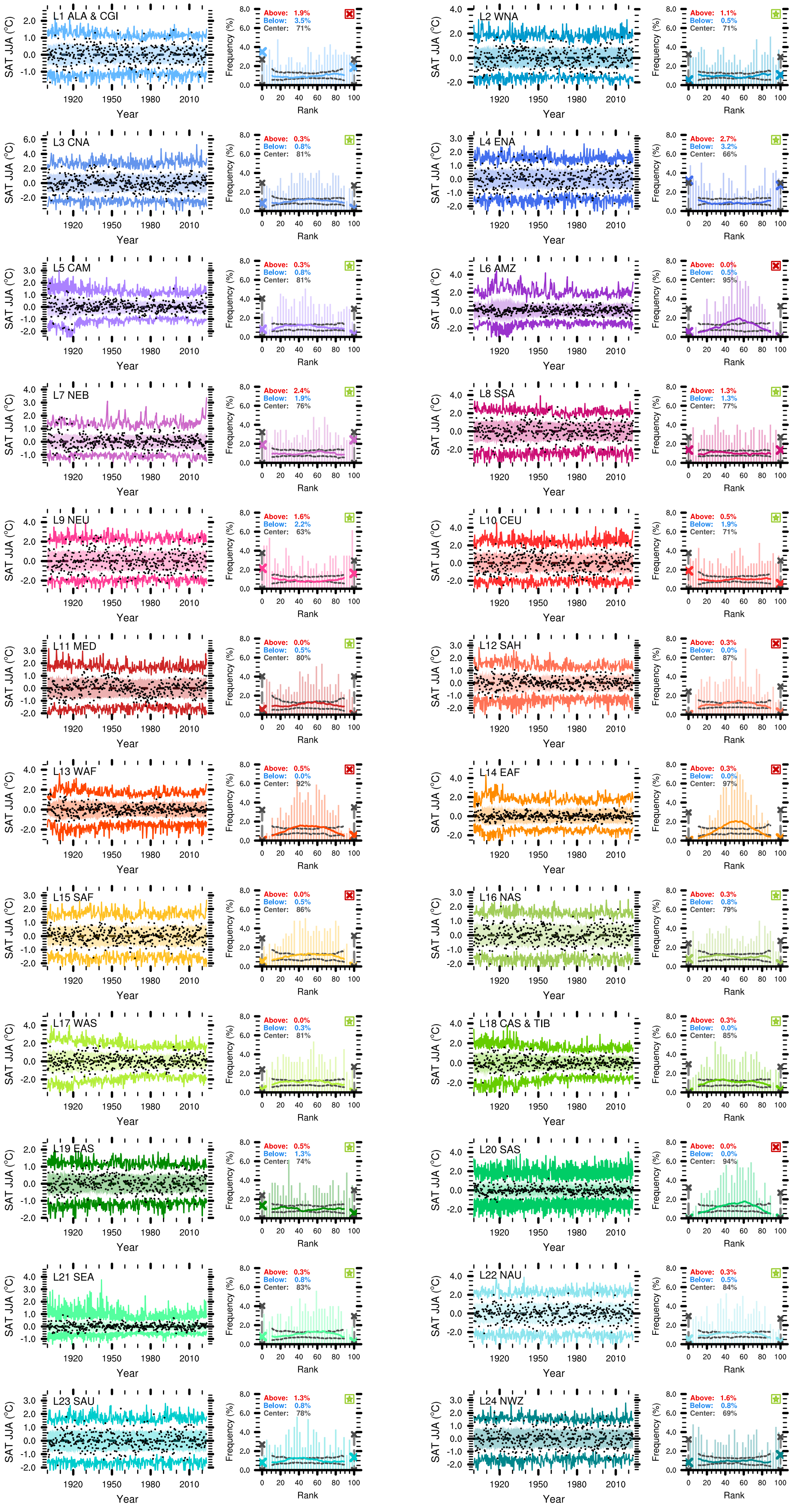

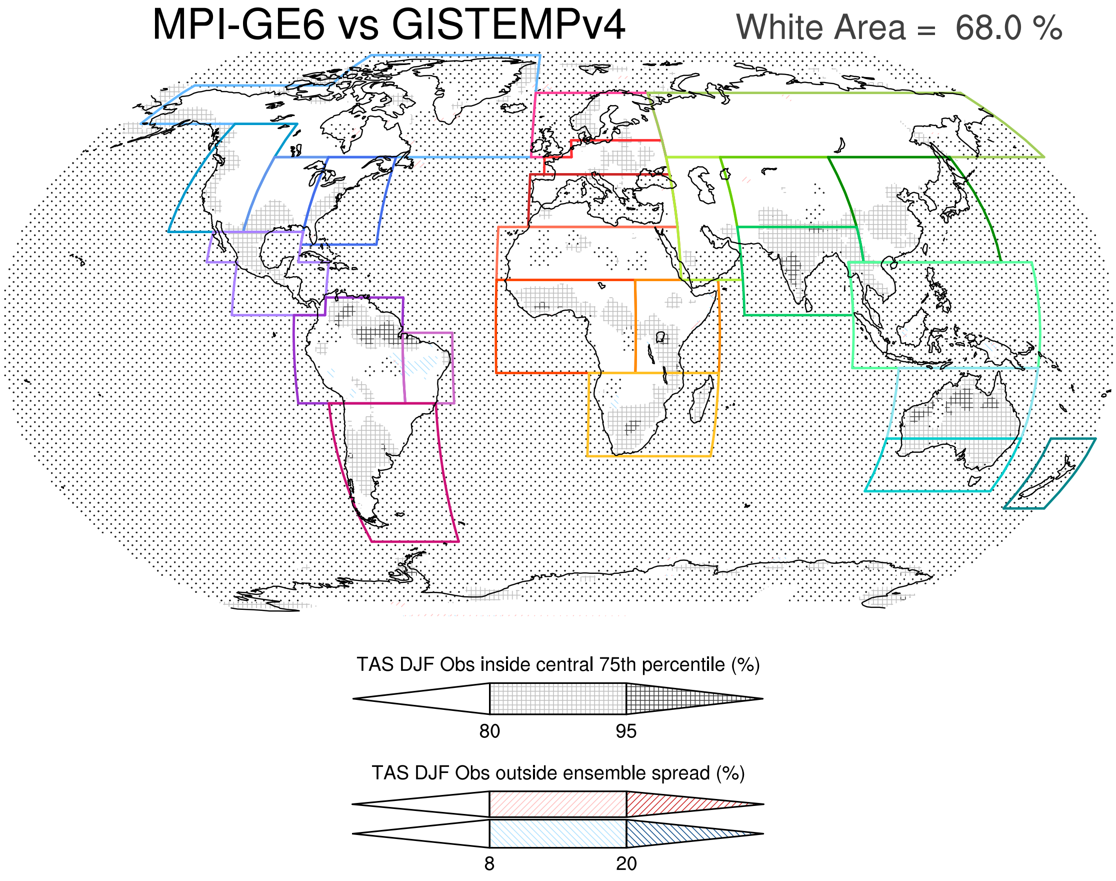
MPI-GE6 vs GISTEMPv4
White Area = 68.0 %
TAS DJF Obs inside central 75th percentile (%)
80
95
TAS DJF Obs outside ensemble spread (%)
8
20

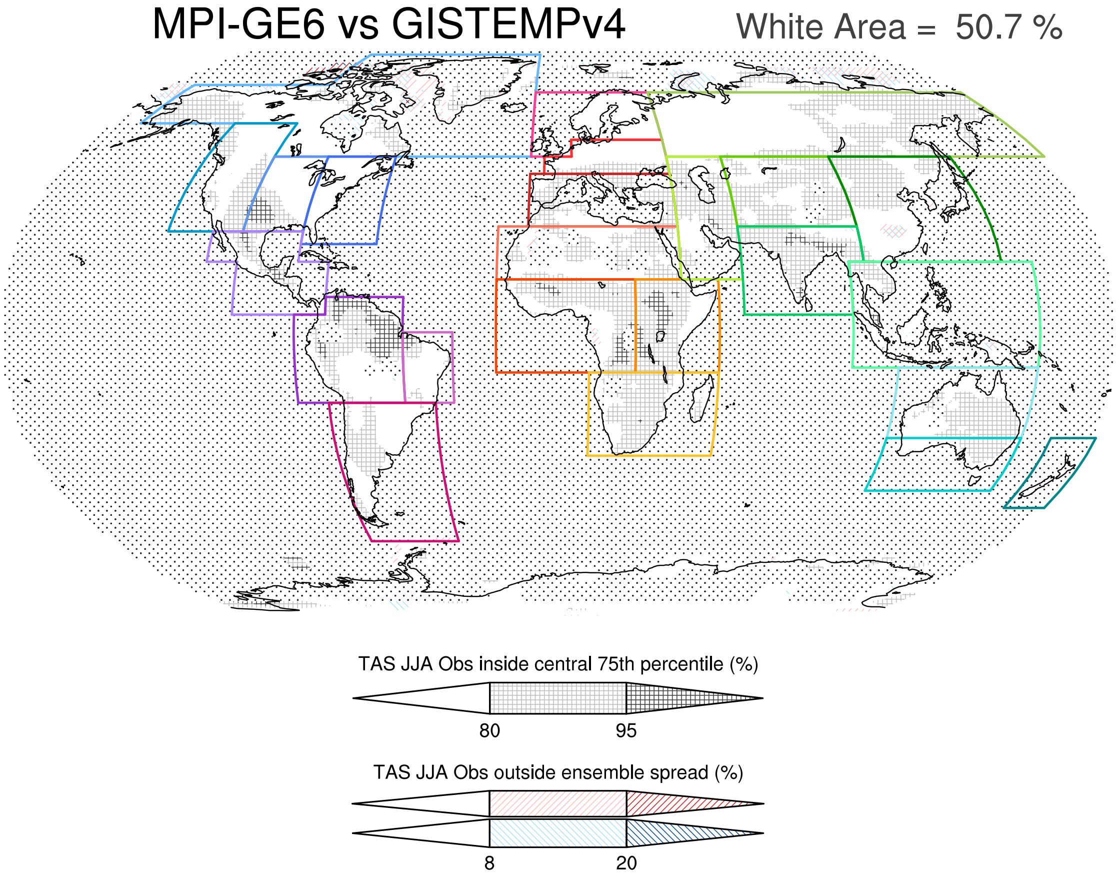
MPI-GE6 vs GISTEMPv4
White Area = 50.7 %
TAS JJA Obs inside central 75th percentile (%)
80
95
TAS JJA Obs outside ensemble spread (%)
8
20

MPI-GE6 vs GISTEMPv4 TAS DJF

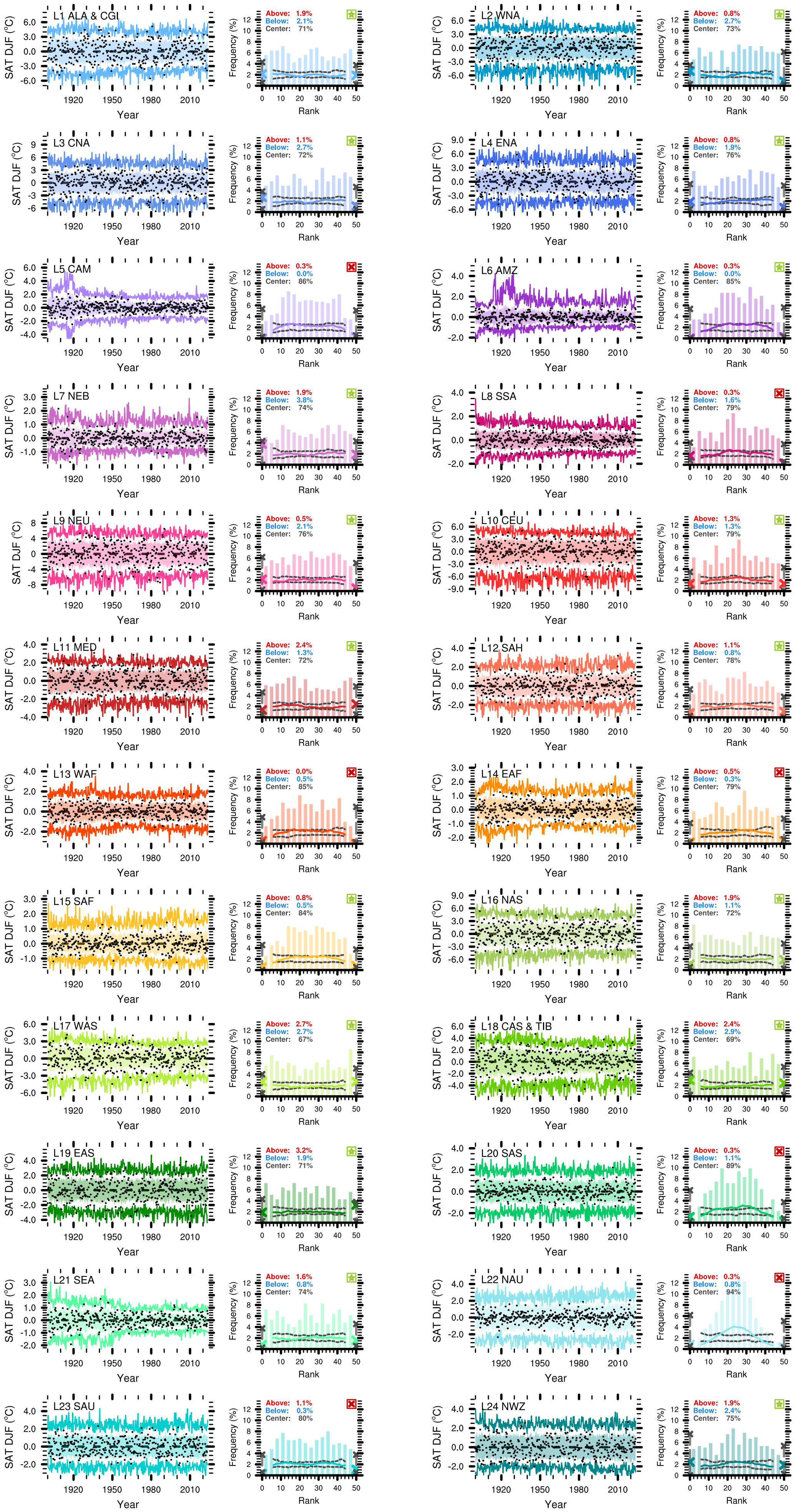

MPI-GE6 vs GISTEMPv4 TAS JJA

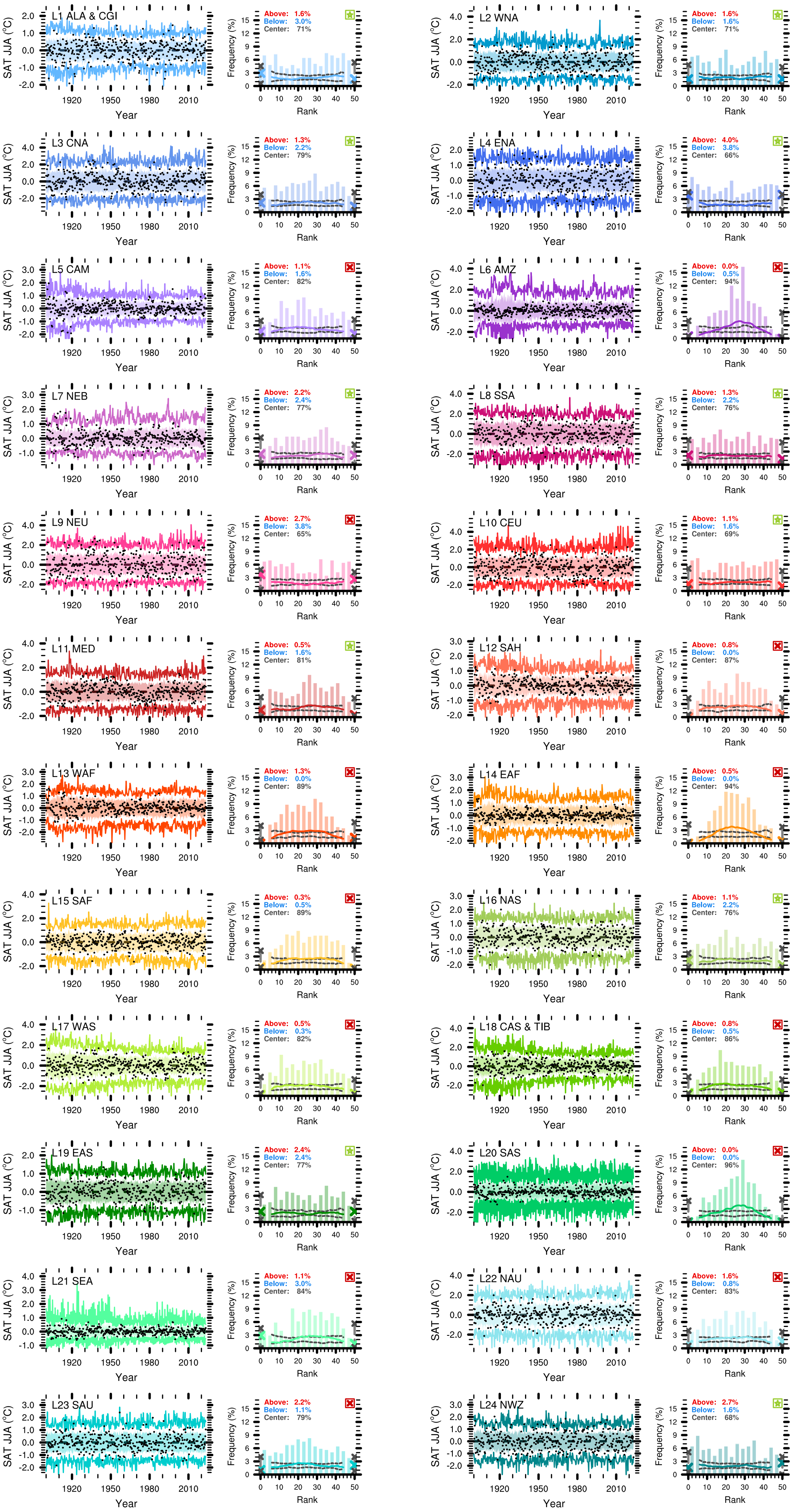

Non-Detrended Land Surface Air Temperatures

Rank-frequency variability evaluation framework for non-detrended 2m air temperature (TAS) anomalies over land grid cells.

Maps show grid-cell evaluation of the simulated DJF and JJA monthly mean temperature anomalies for the 11 SMILEs included against GISTEMPv4 observations globally. Gray hatching represents where observations cluster within the 75th percentile bounds of the ensemble (12.5th to 87.5th percentiles) for more than 80% of months (light grey) or for more than 95% of months (dark grey). Red and blue shading represents where observations are larger than the ensemble maximum (red) or smaller than the ensemble minimum (blue), respectively, for more than 8% of the months (light red and blue) or for more than 20% of the months (dark red and blue). Dotted areas represent ocean areas or grid cells where observations are missing and are therefore excluded from this analysis. Colored boxes demark the boundaries of each land region assessed. The percentage of assessed grid-cells that present none of these biases, unbiased grid-cells, in given at the top (white area). For each assessed region, models are considered to provide an adequate grid-cell representation when they exhibit more than 50% of unbiased grid cells in the region, fulfilling Criteria 2 of our evaluation framework.

Time series and rank frequency histograms show spatially aggregated DJF and JJA TAS for each land region for all 11 SMILEs. Time series show the ensemble maximum and minimum (color lines) and central 75th percentile ensemble spread (shading) are shown against observations (black dots).

Rank histograms represent the frequency of each place that observations would take in a list of ensemble members ordered by ascending temperature anomaly values. Rank histograms show the observations rank frequency accumulated for 3-rank bins (bars), the running mean rank frequency over a centered n/5 rank window (lines; for 1 to n-1 ranks) and the absolute frequencies of rank 0 and n (crosses), with n the number of ensemble members, for observations (color) and perfect model rank range (gray).

To illustrate how internal variability sampling may affect rank frequencies given the non-infinite record length considered, we include a comparison to the perfect-model rank range, which shows the range of rank frequencies that each ensemble member would take if it were observations (see Methods in main article for further details). If the rank exhibited by observations (colors) is within this perfect-model range (gray; allowing a maximum 10% deviation outside of this range), the rank frequency evaluation shows an adequate model performance, and any deviations from a perfectly frank rank histogram can be assumed to be within the range of deviations that could be caused by internal variability. In the case the observations rank frequency is within the perfect-model range for all rank windows, Criteria 1 of our evaluation framework is met and this is highlighted by a green star at the top right; if not, by a red cross. Percentages at the top left show the frequency of regionally averaged observations occurring above (red) or below (blue) ensemble limits, or clustering within the central 75th percentile range (gray), analogous to the grid-cell evaluation in Criteria 2.

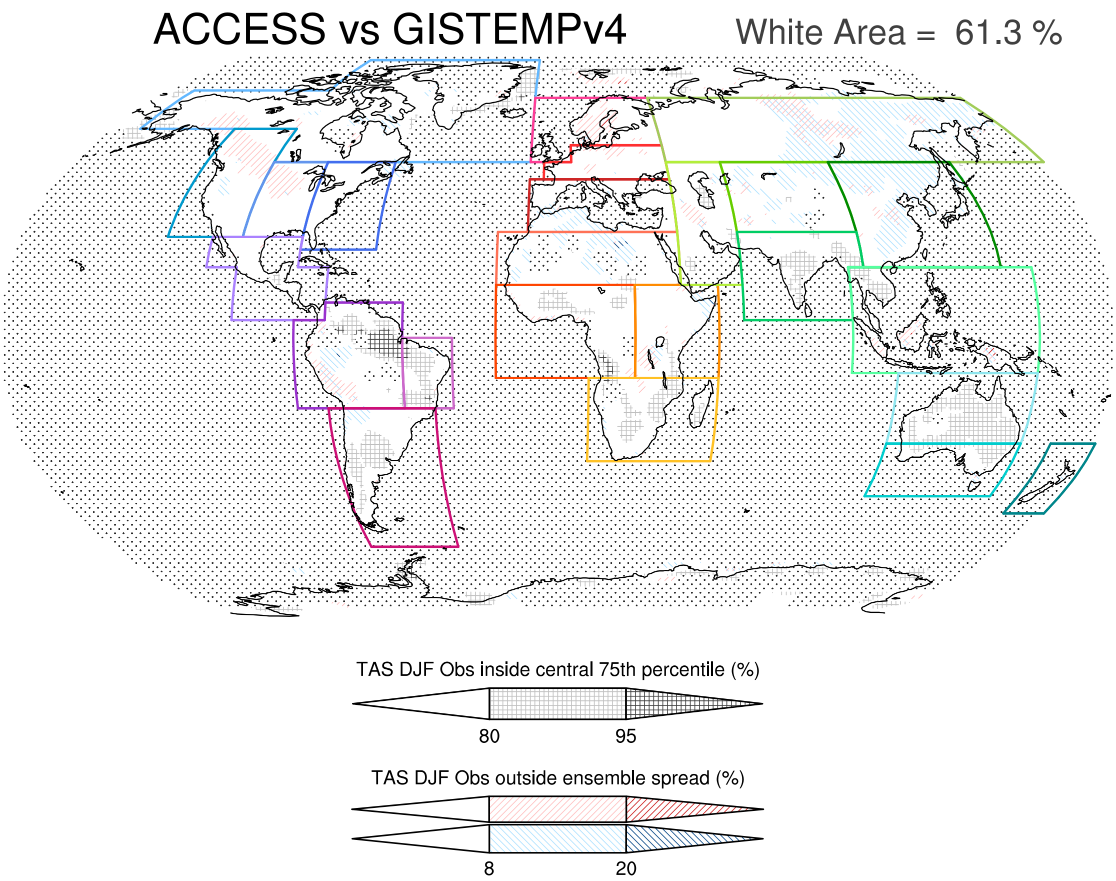
ACCESS vs GISTEMPv4
White Area = 61.3 %
TAS DJF Obs inside central 75th percentile (%)
80
95
TAS DJF Obs outside ensemble spread (%)
8
20

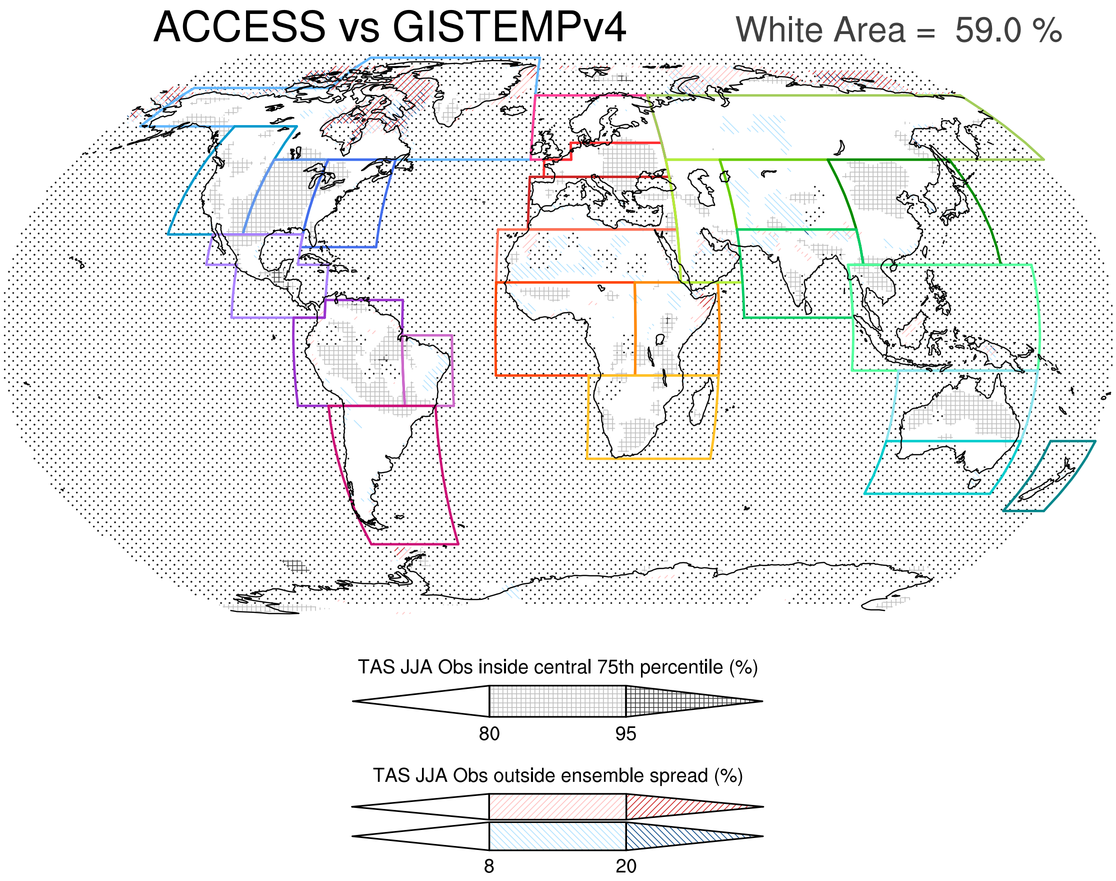
ACCESS vs GISTEMPv4
White Area = 59.0 %
TAS JJA Obs inside central 75th percentile (%)
80
95
TAS JJA Obs outside ensemble spread (%)
8
20

ACCESS vs GISTEMPv4 TAS DJF

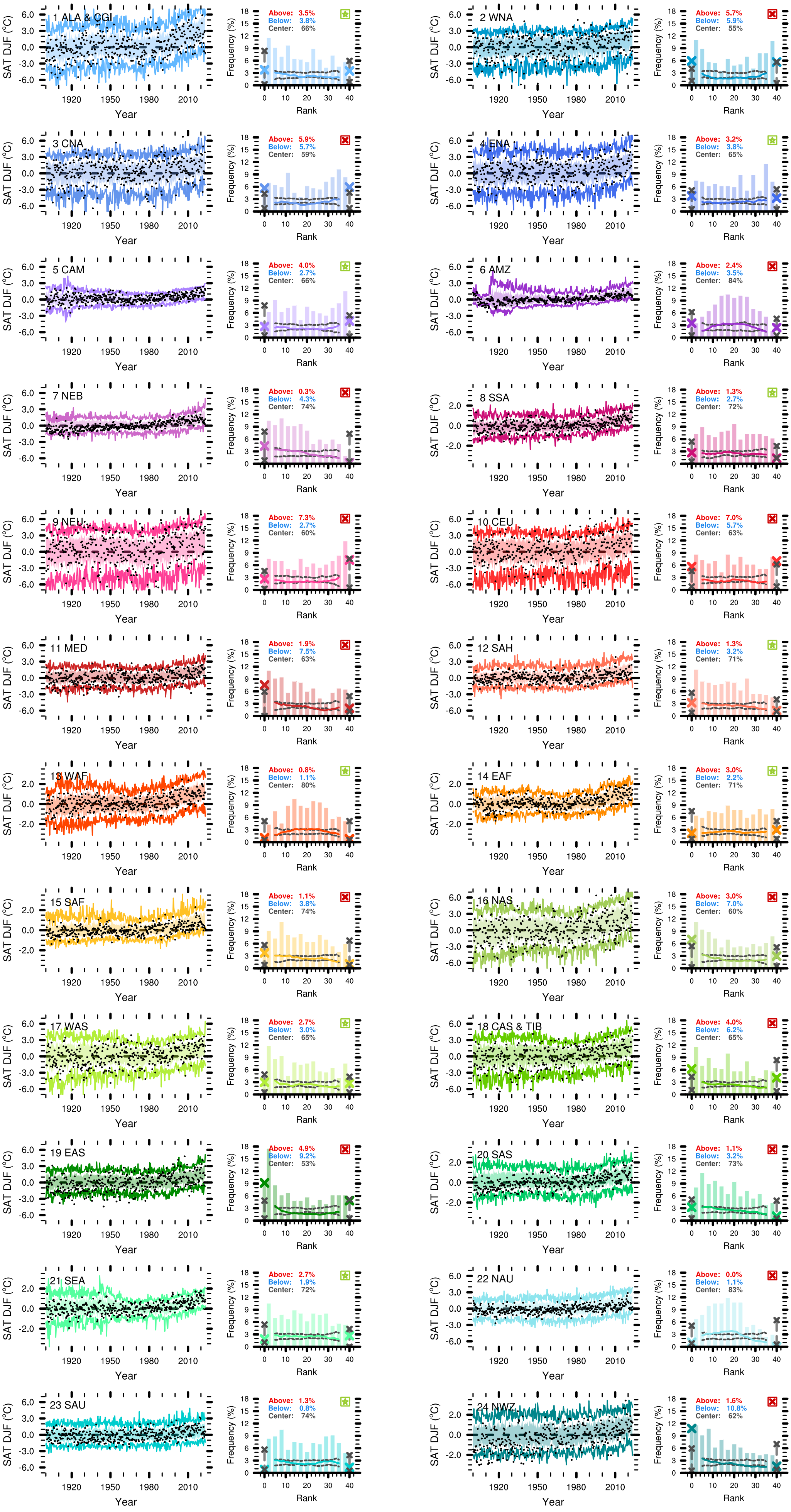

ACCESS vs GISTEMPv4 TAS JJA

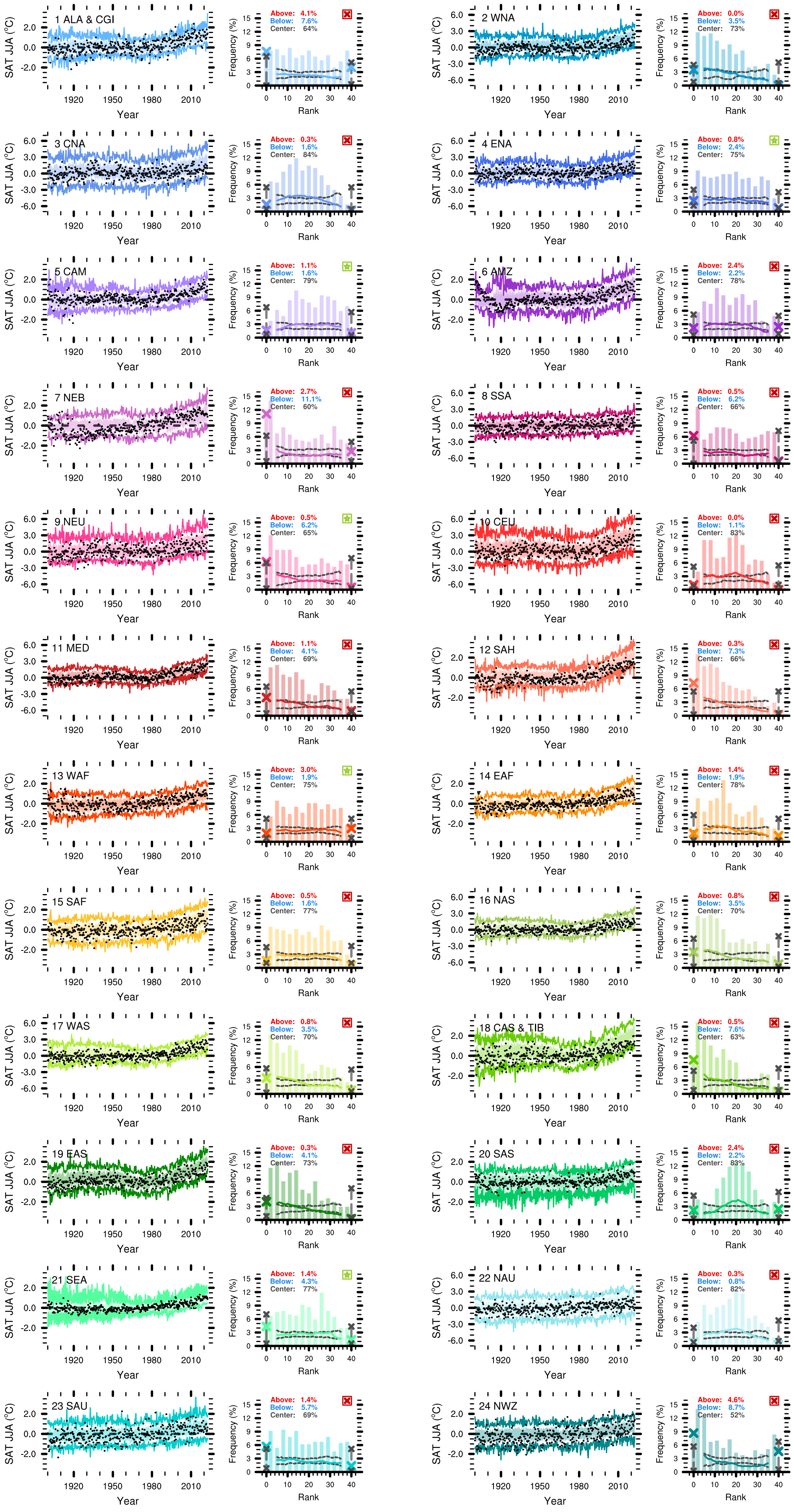

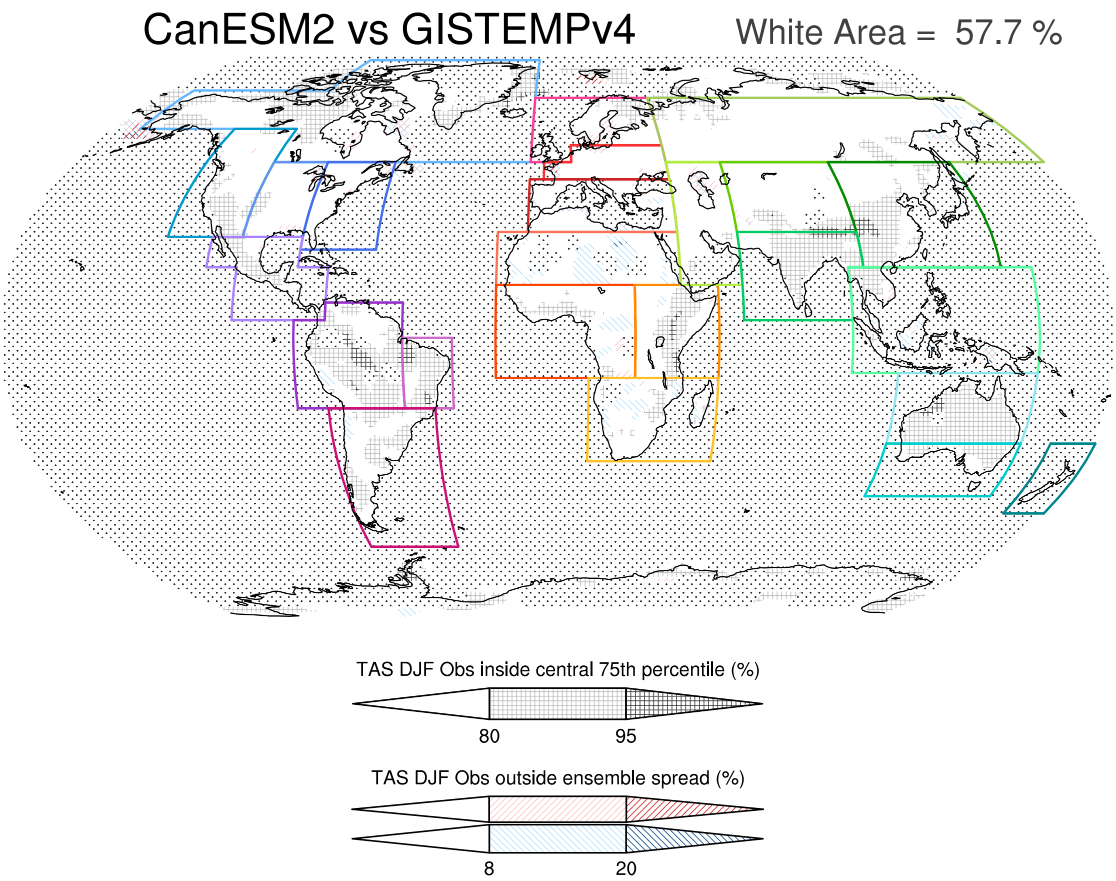
CanESM2 vs GISTEMPv4
White Area = 57.7 %
TAS DJF Obs inside central 75th percentile (%)
80
95
TAS DJF Obs outside ensemble spread (%)
8
20

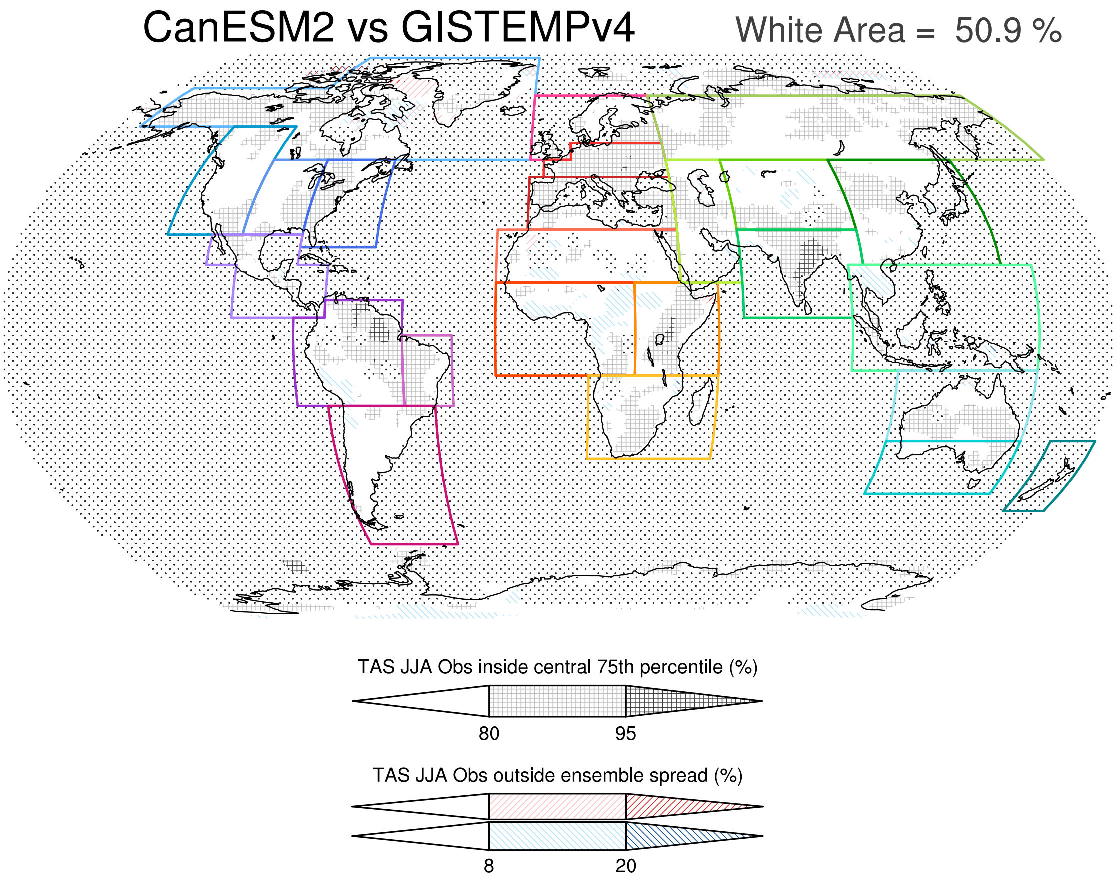
CanESM2 vs GISTEMPv4
White Area = 50.9 %
TAS JJA Obs inside central 75th percentile (%)
80
95
TAS JJA Obs outside ensemble spread (%)
8
20

CanESM2 vs GISTEMPv4 TAS DJF

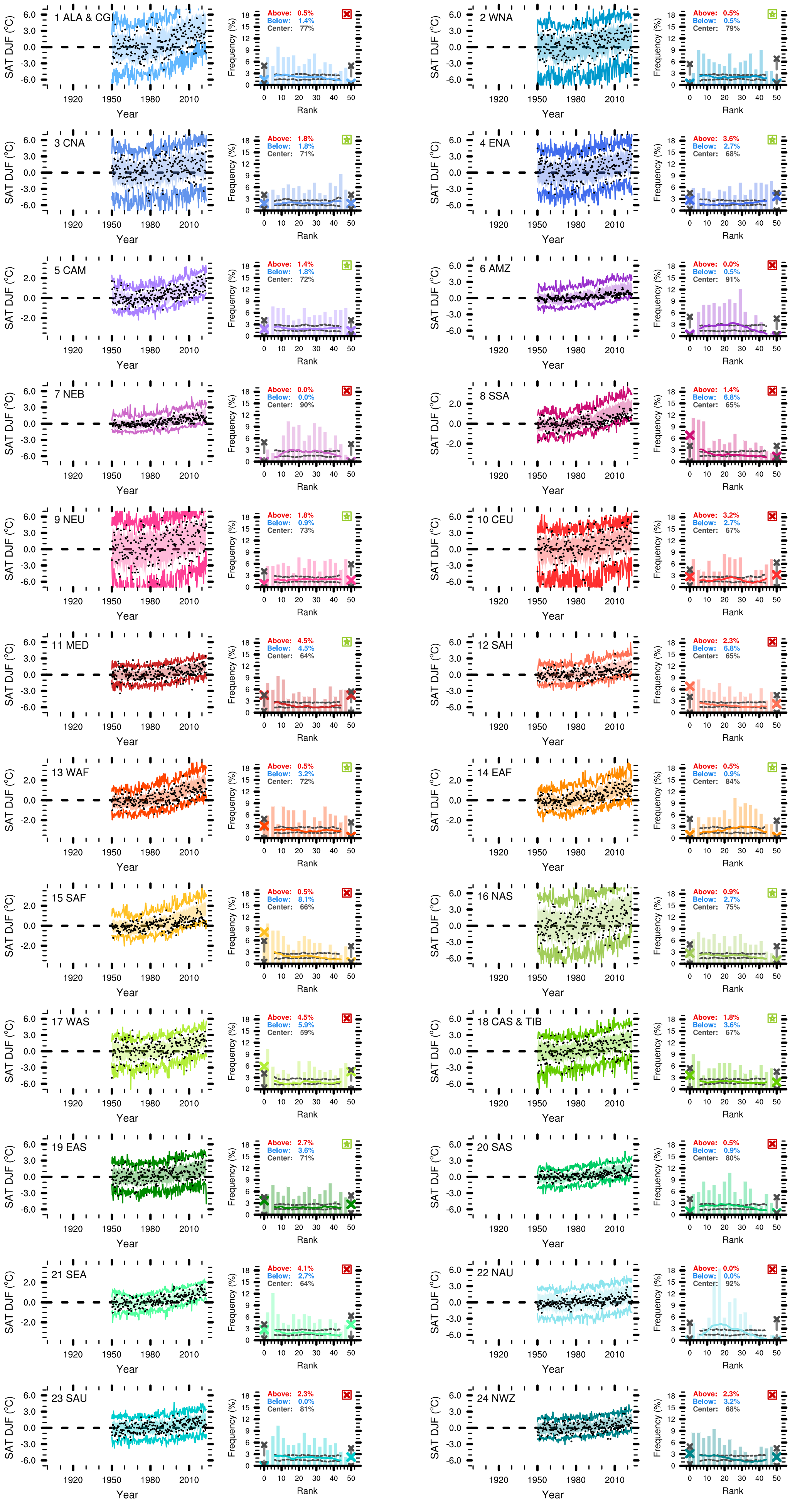

CanESM2 vs GISTEMPv4 TAS JJA

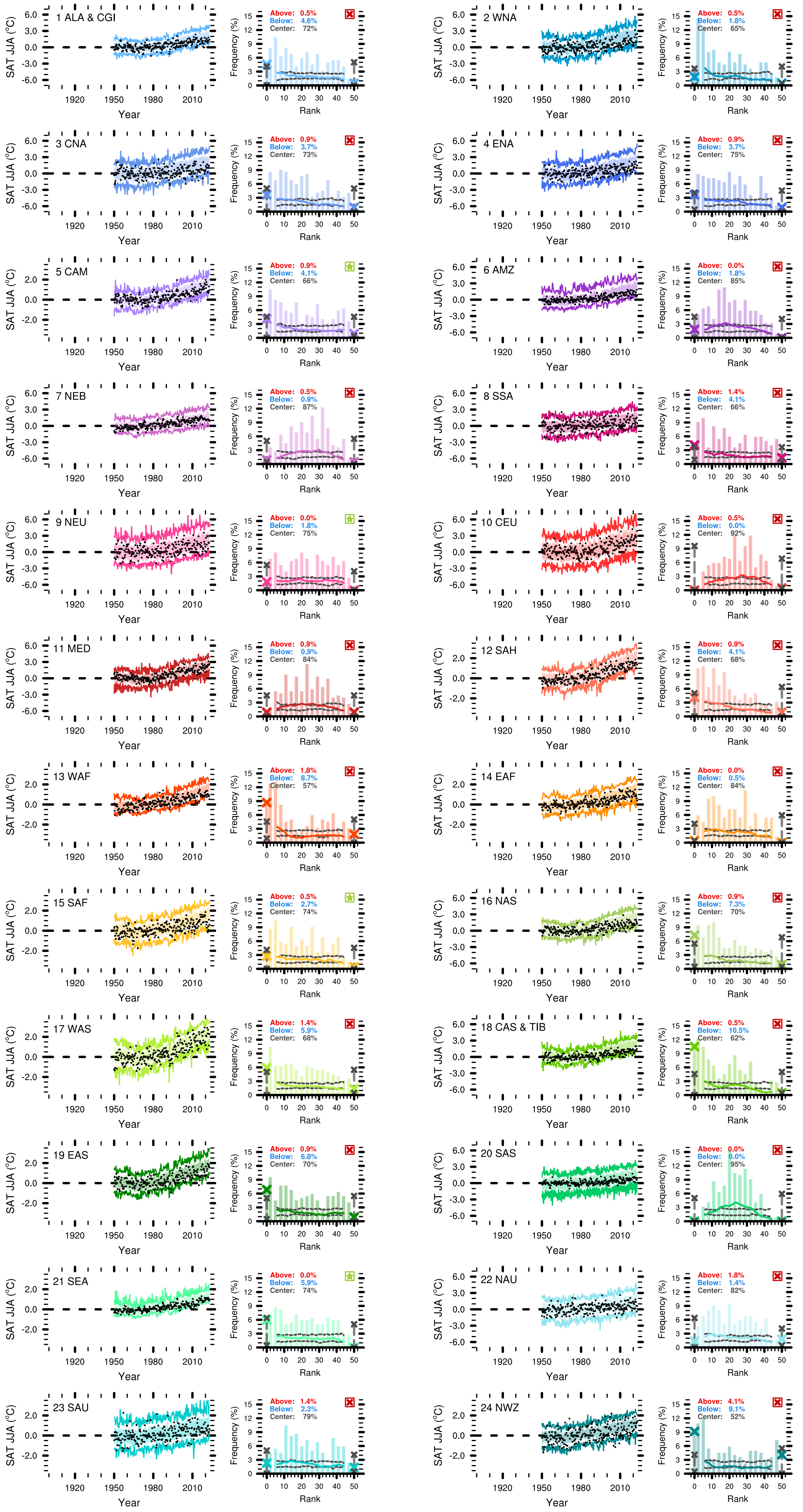

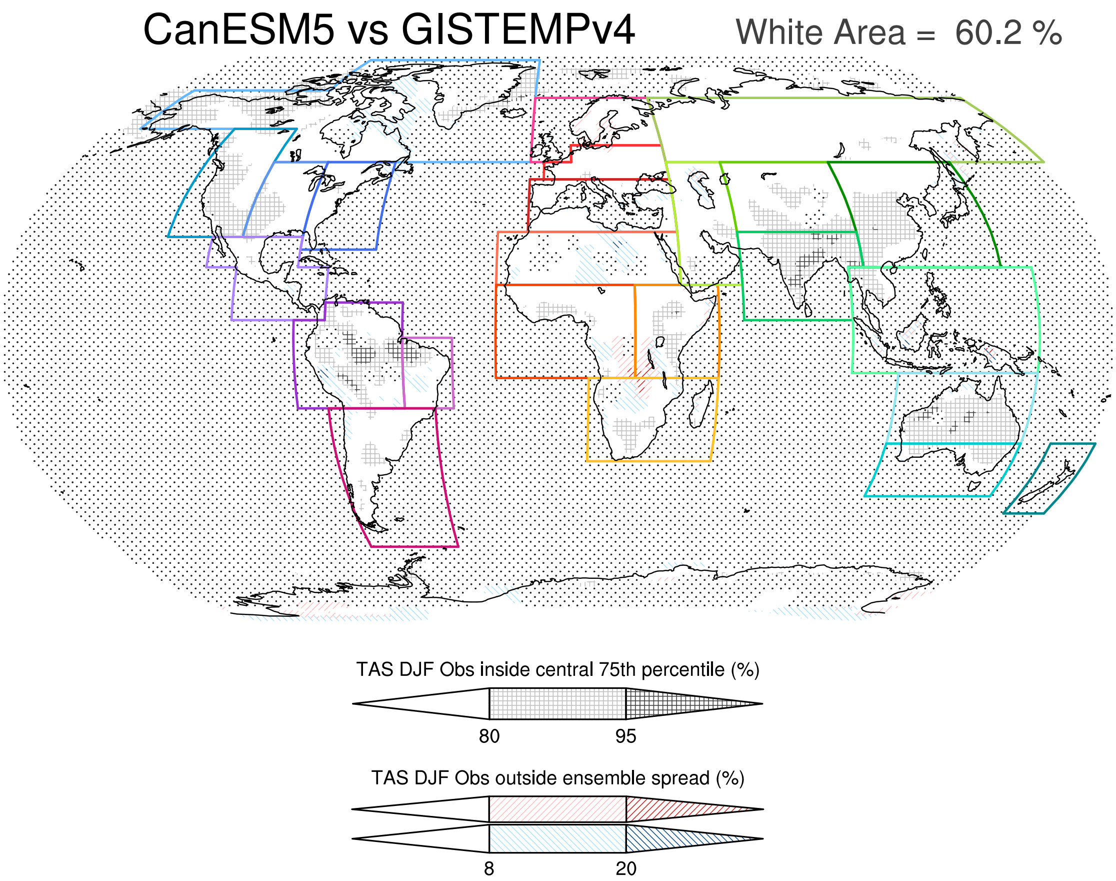
CanESM5 vs GISTEMPv4
White Area = 60.2 %
TAS DJF Obs inside central 75th percentile (%)
80
95
TAS DJF Obs outside ensemble spread (%)
8
20

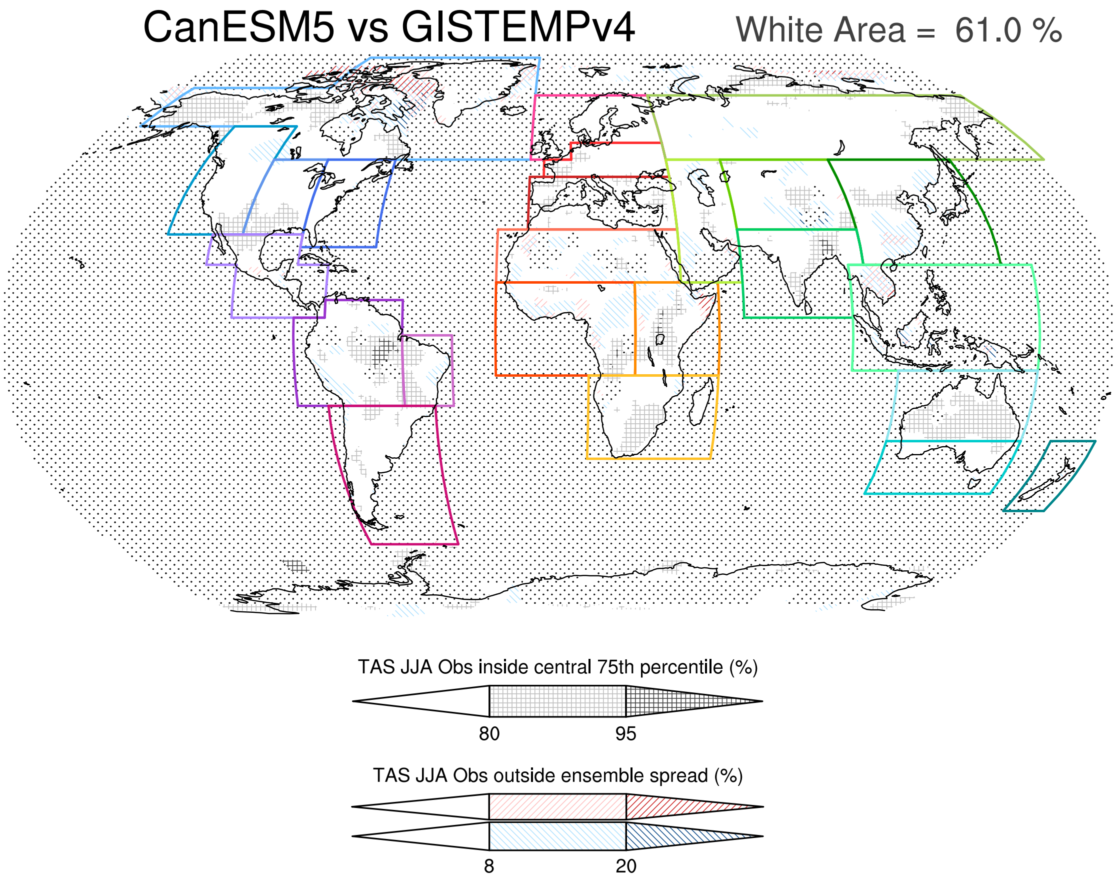
CanESM5 vs GISTEMPv4
White Area = 61.0 %
TAS JJA Obs inside central 75th percentile (%)
80
95
TAS JJA Obs outside ensemble spread (%)
8
20

CanESM5 vs GISTEMPv4 TAS DJF

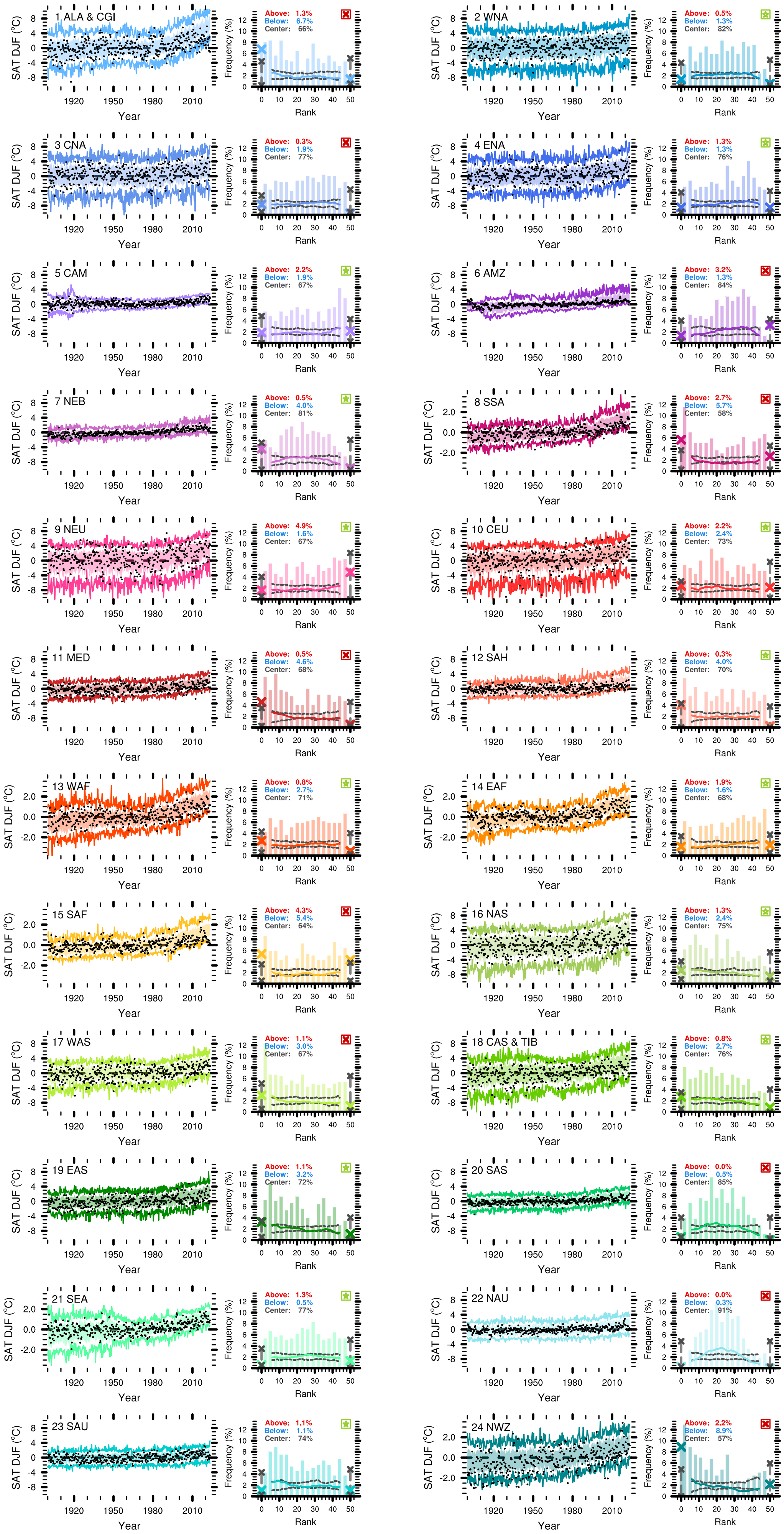

CanESM5 vs GISTEMPv4 TAS JJA

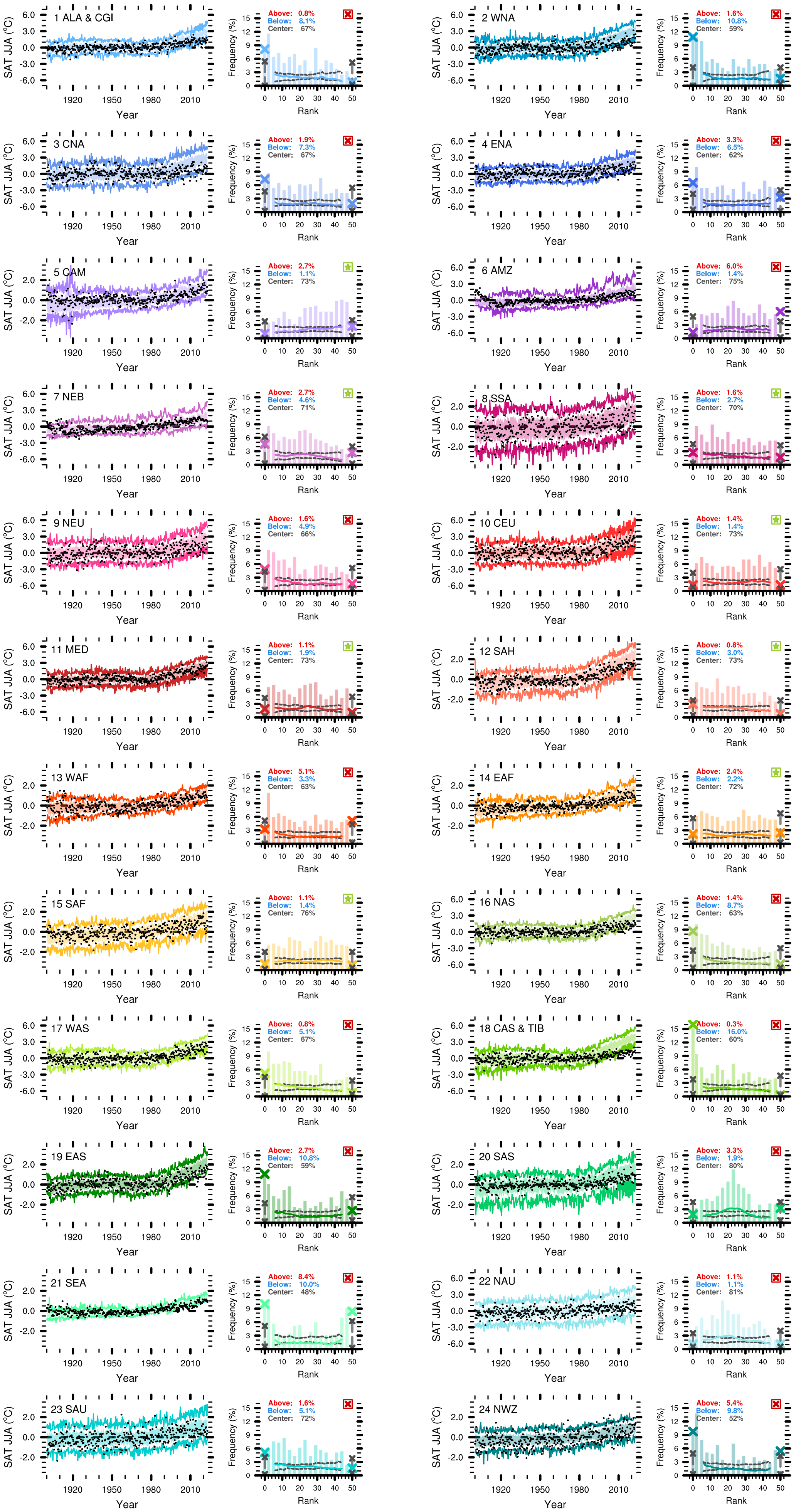

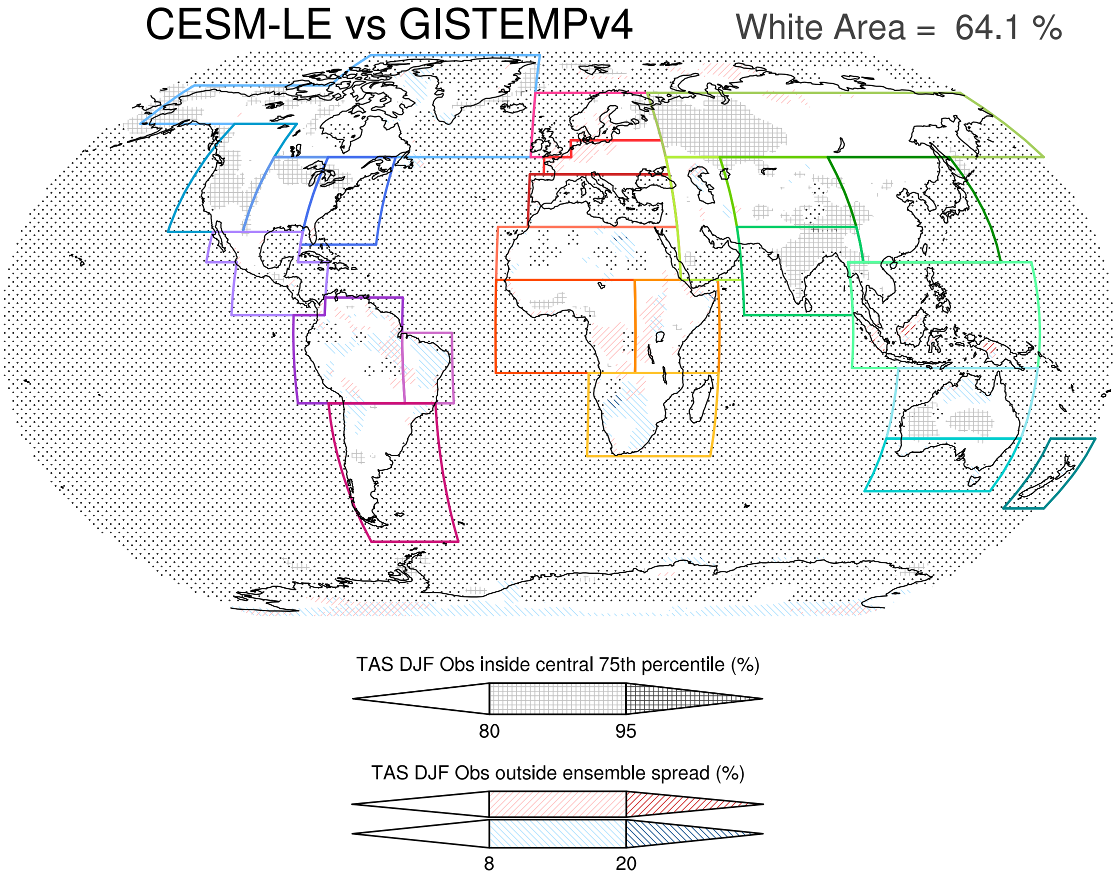
CESM-LE vs GISTEMPv4
White Area = 64.1 %
TAS DJF Obs inside central 75th percentile (%)
80
95
TAS DJF Obs outside ensemble spread (%)
8
20

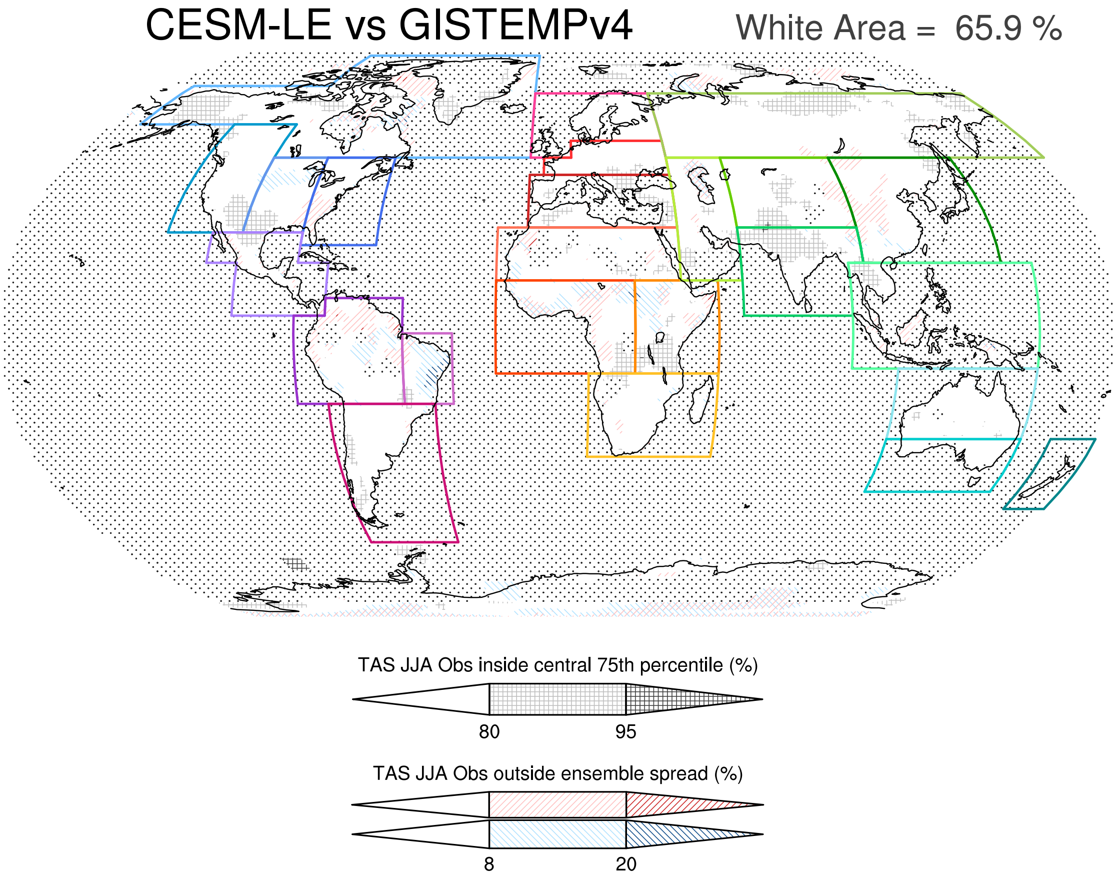
CESM-LE vs GISTEMPv4
White Area = 65.9 %
TAS JJA Obs inside central 75th percentile (%)
80
95
TAS JJA Obs outside ensemble spread (%)
8
20

CESM-LE vs GISTEMPv4 TAS DJF

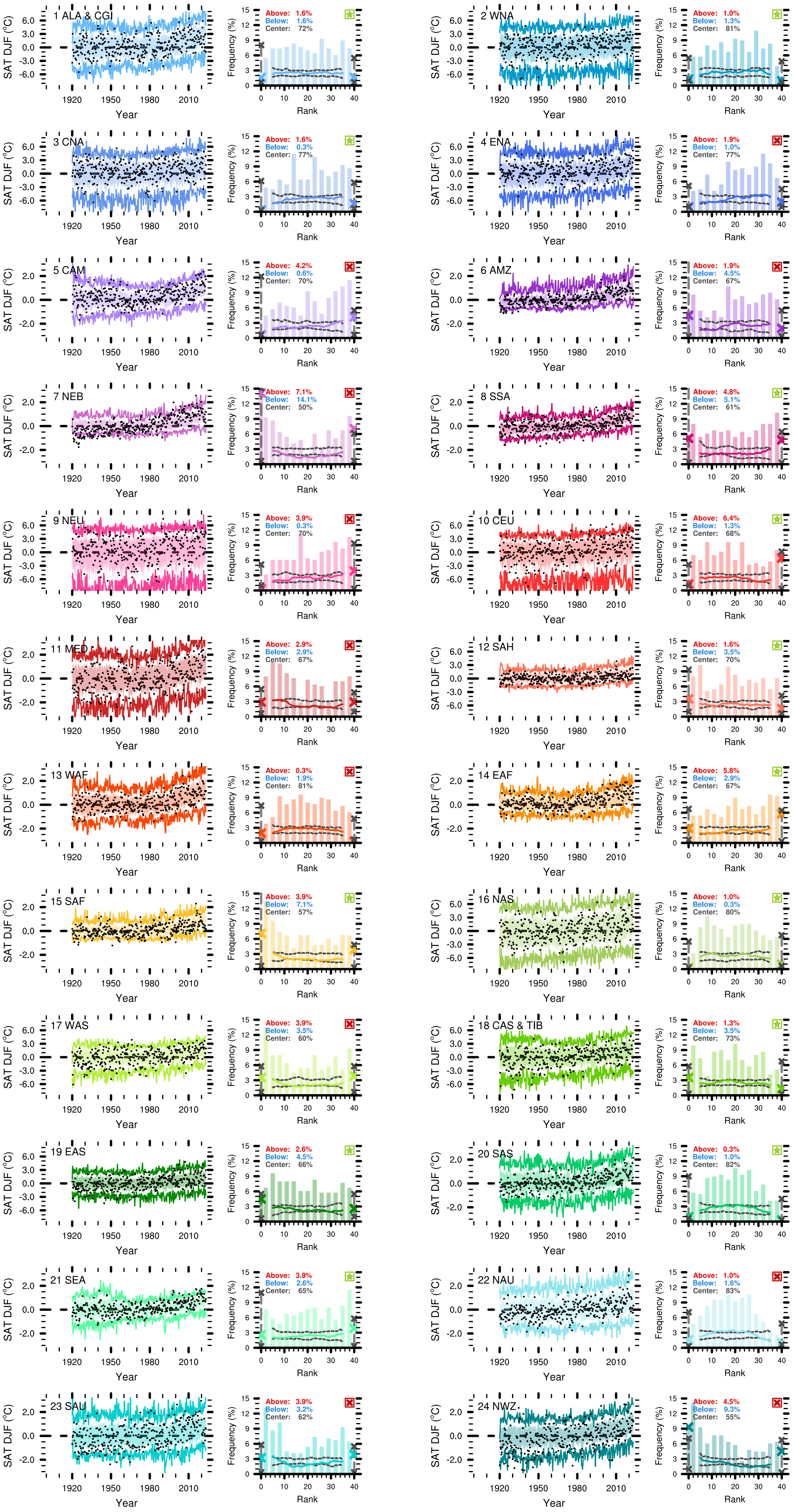

CESM-LE vs GISTEMPv4 TAS JJA

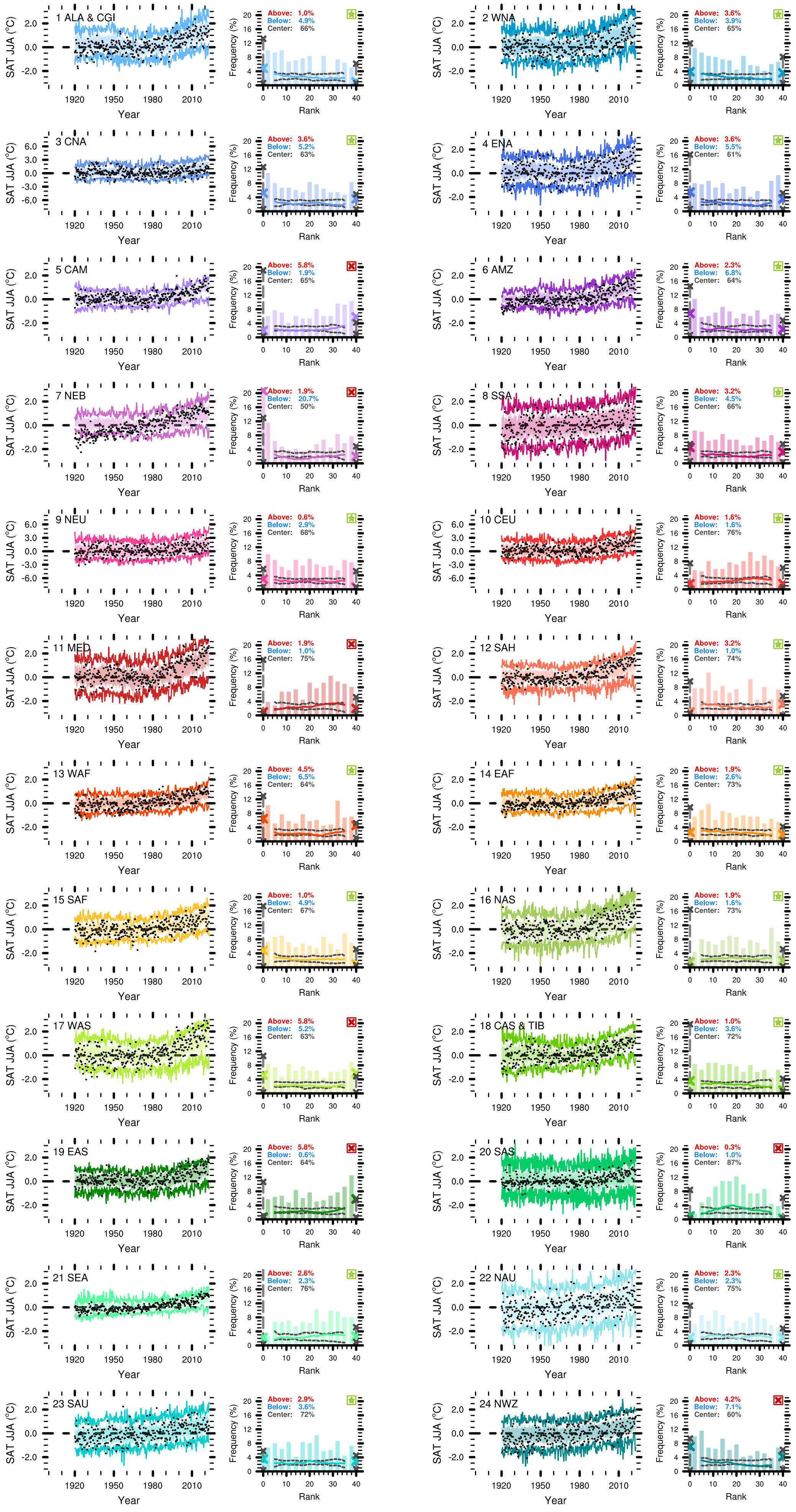

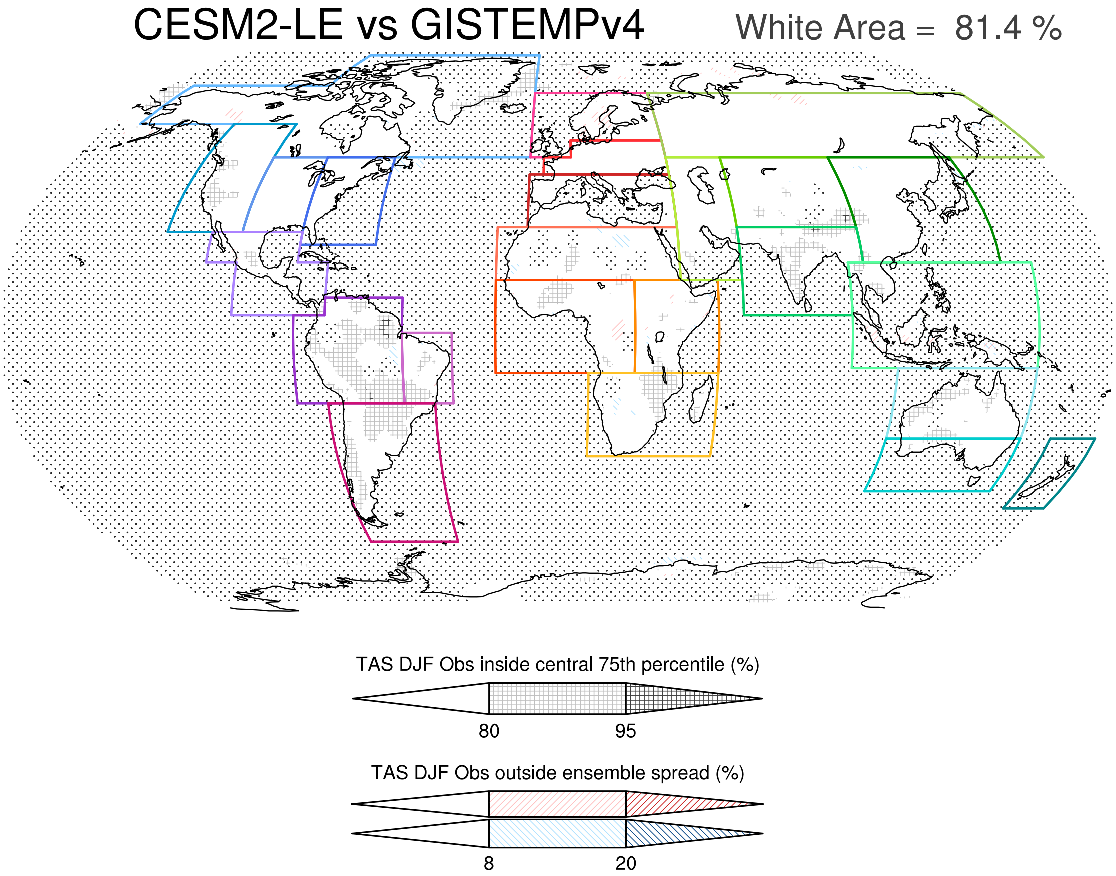
CESM2-LE vs GISTEMPv4
White Area = 81.4 %
TAS DJF Obs inside central 75th percentile (%)
80
95
TAS DJF Obs outside ensemble spread (%)
8
20

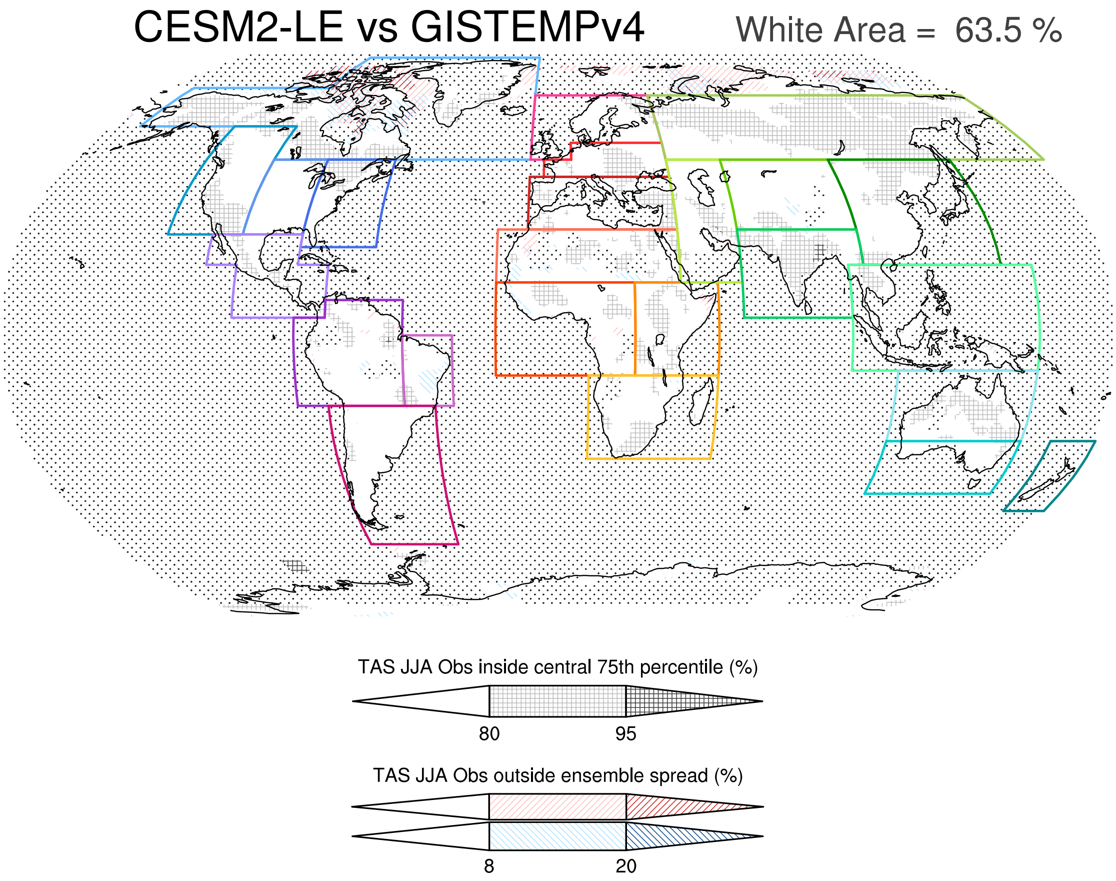
CESM2-LE vs GISTEMPv4
White Area = 63.5 %
TAS JJA Obs inside central 75th percentile (%)
80
95
TAS JJA Obs outside ensemble spread (%)
8
20

CESM2-LE vs GISTEMPv4 TAS DJF

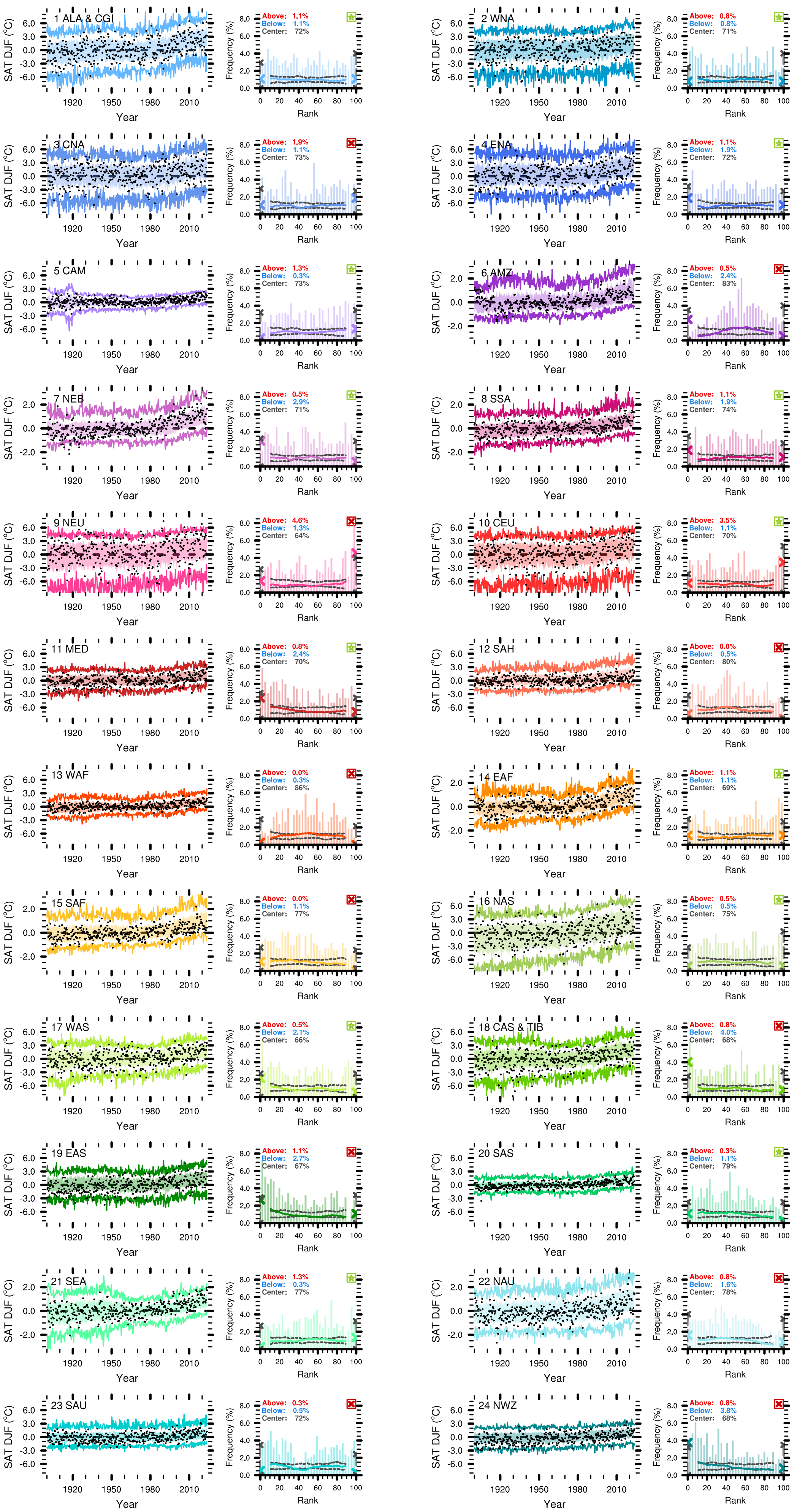

CESM2-LE vs GISTEMPv4 TAS JJA

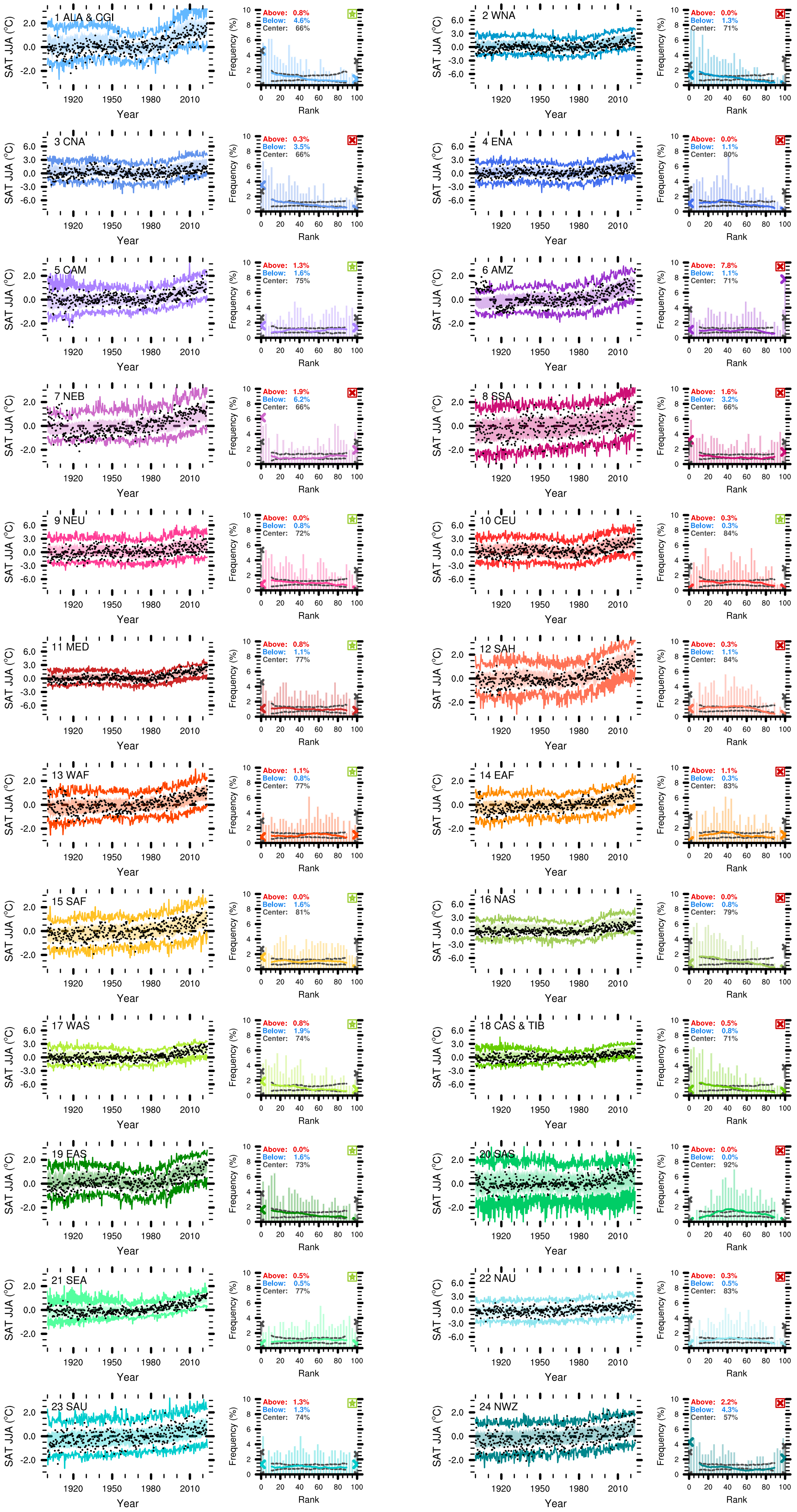

CSIRO-Mk360 vs GISTEMPv4 White Area = 57.4 %

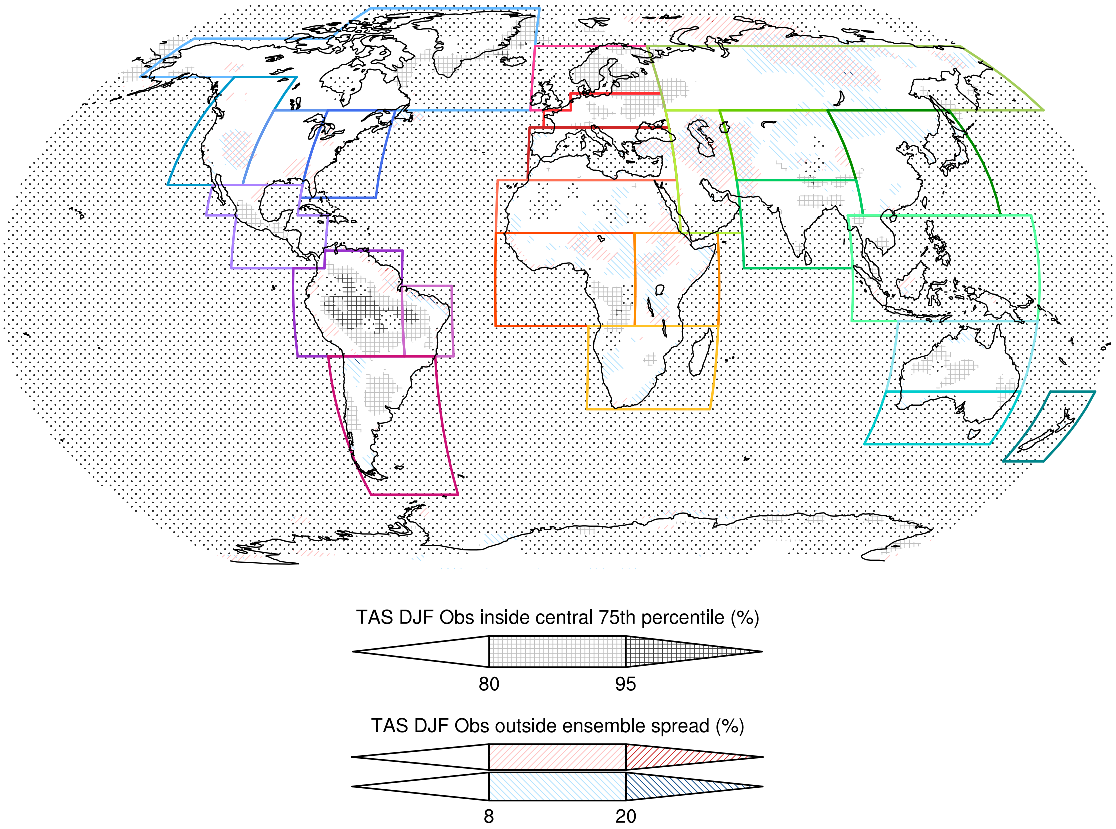

CSIRO-Mk360 vs GISTEMPv4 White Area = 54.9 %

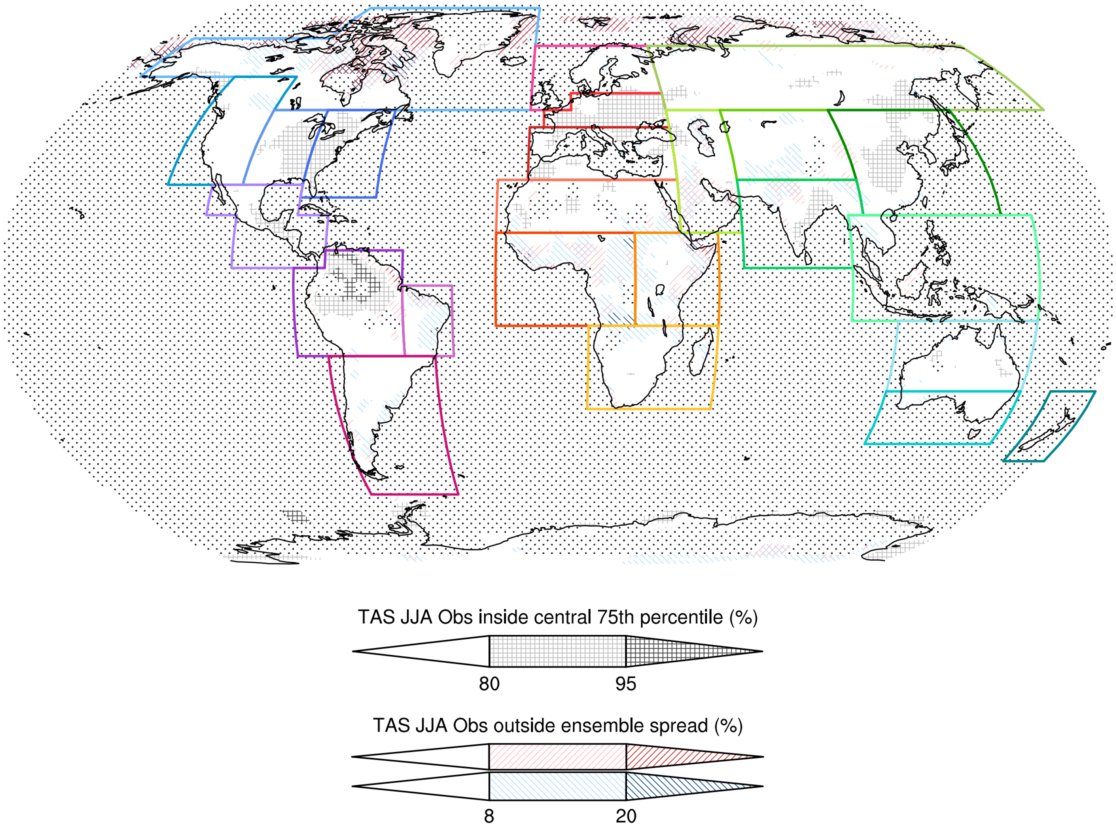

CSIRO-Mk360 vs GISTEMPv4 TAS DJF

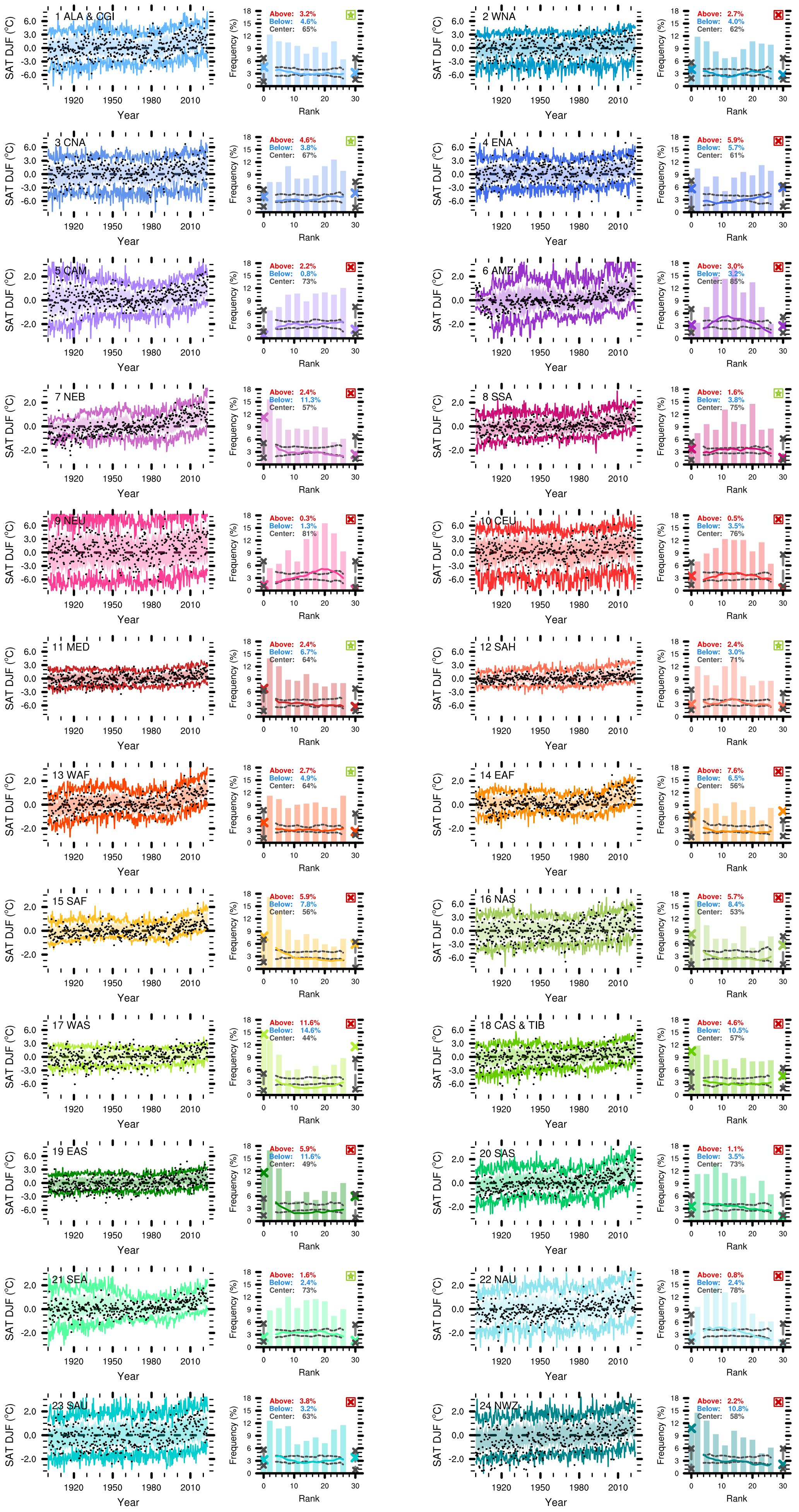

CSIRO-Mk360 vs GISTEMPv4 TAS JJA

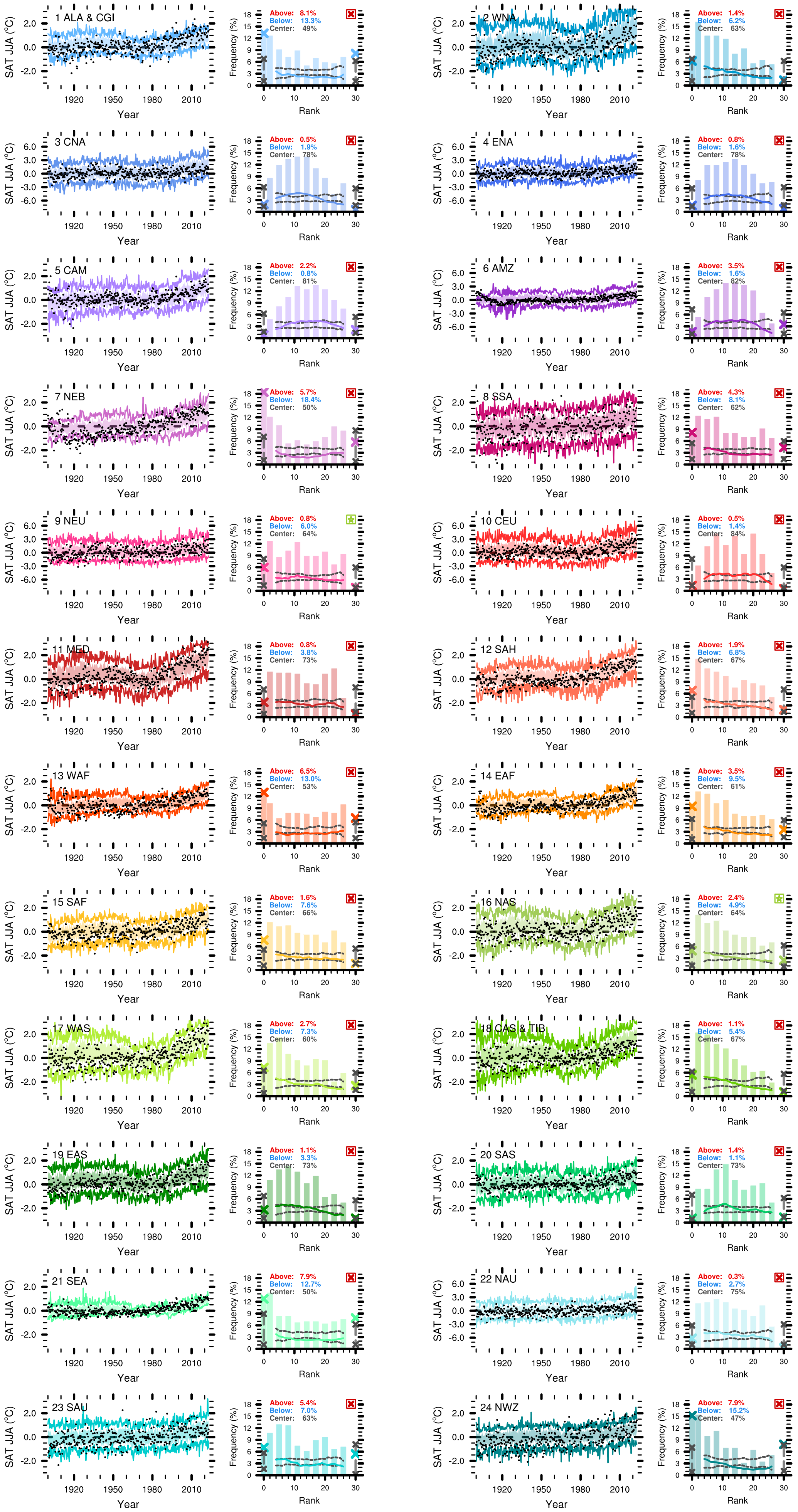

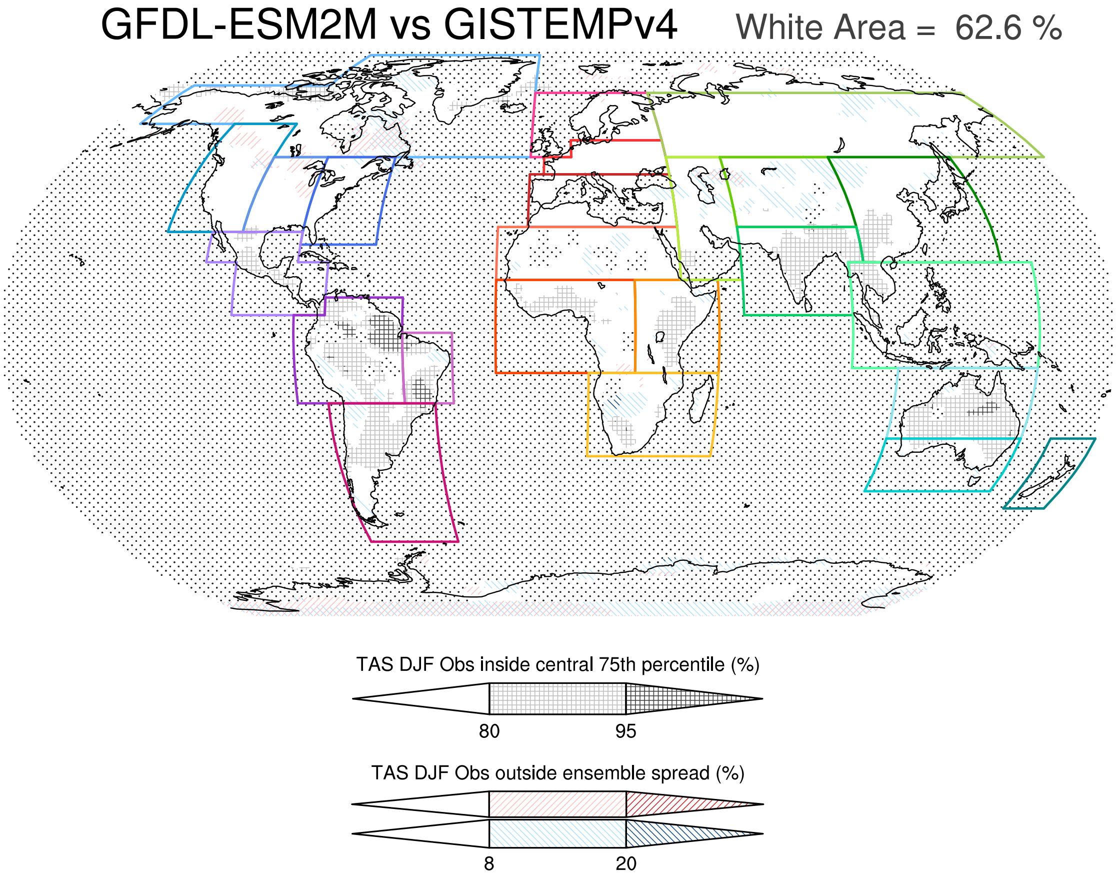
GFDL-ESM2M vs GISTEMPv4
White Area = 62.6 %
TAS DJF Obs inside central 75th percentile (%)
80
95
TAS DJF Obs outside ensemble spread (%)
8
20

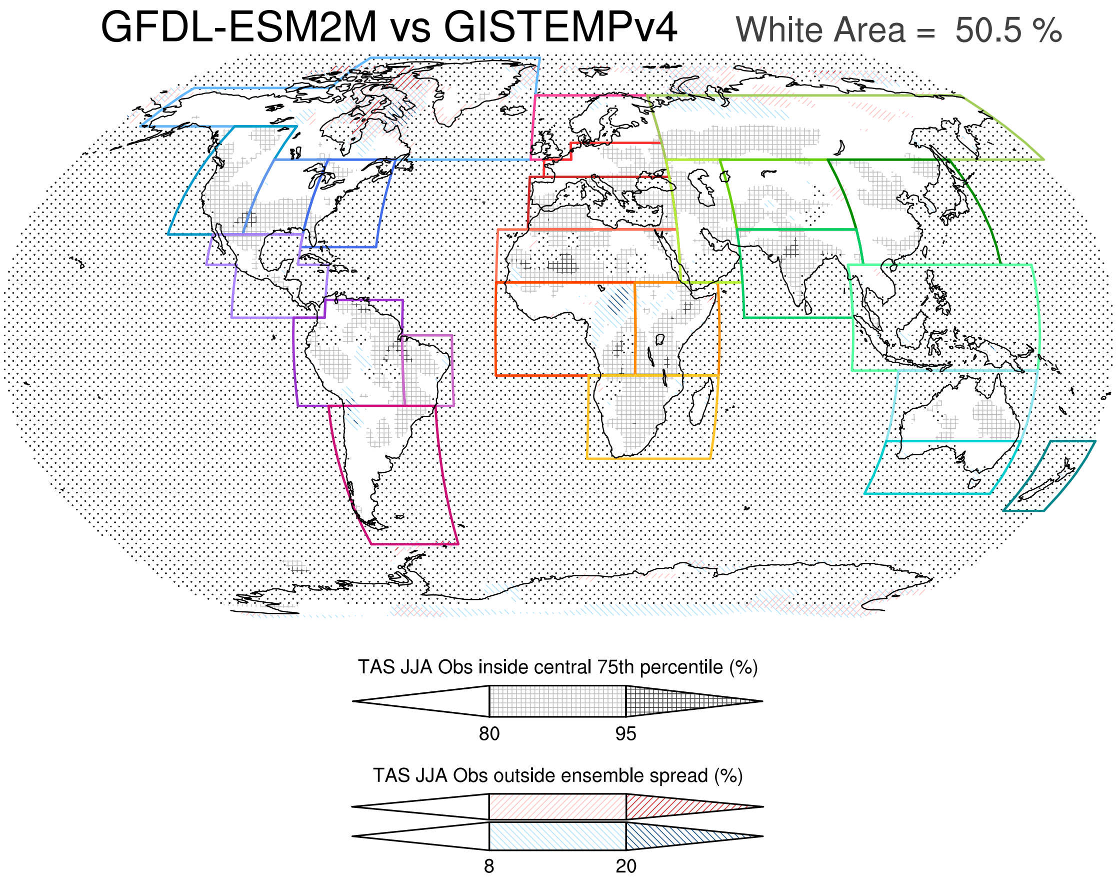
GFDL-ESM2M vs GISTEMPv4
White Area = 50.5 %
TAS JJA Obs inside central 75th percentile (%)
80
95
TAS JJA Obs outside ensemble spread (%)
8
20

GFDL-ESM2M vs GISTEMPv4 TAS DJF

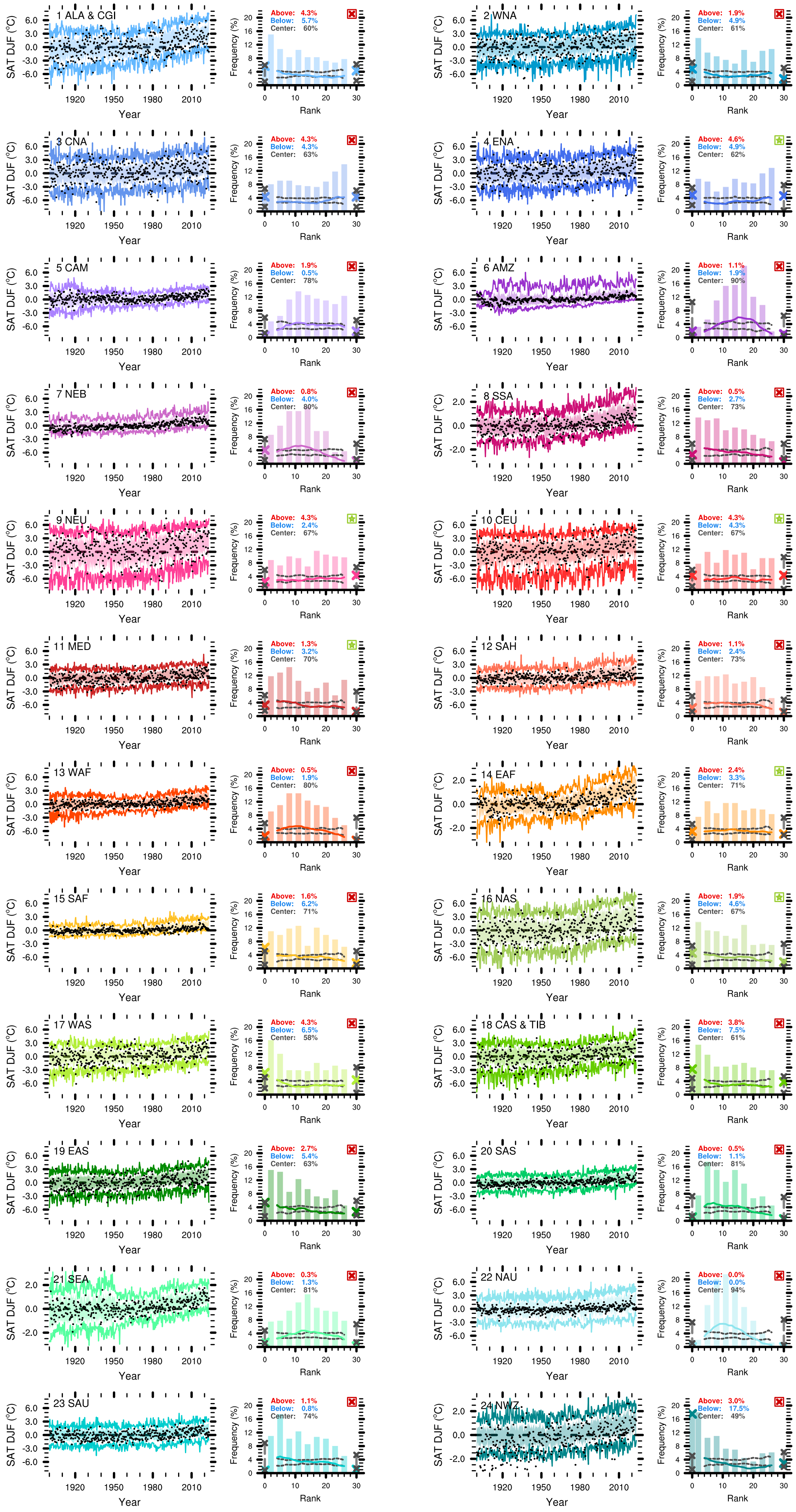

GFDL-ESM2M vs GISTEMPv4 TAS JJA

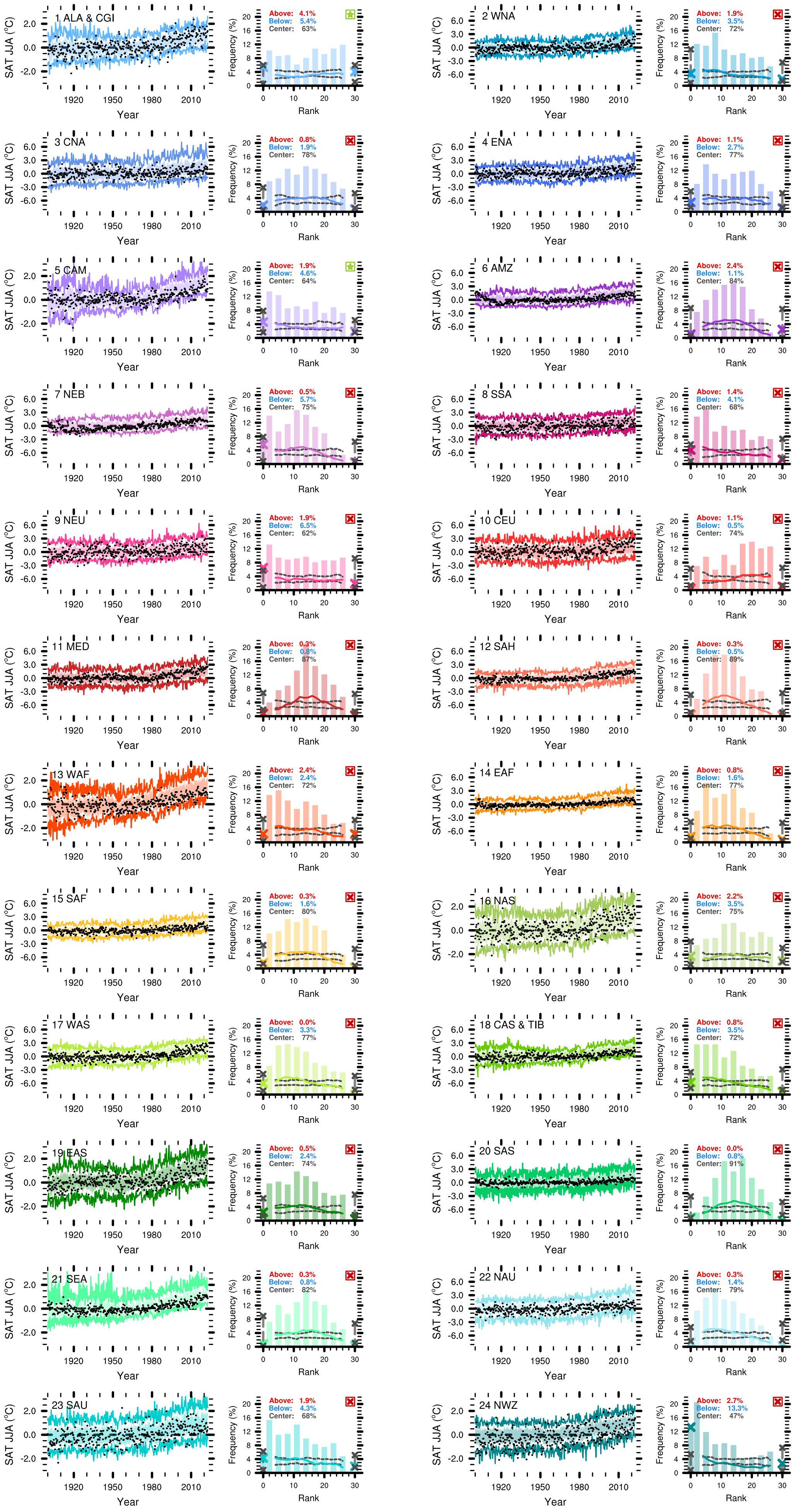

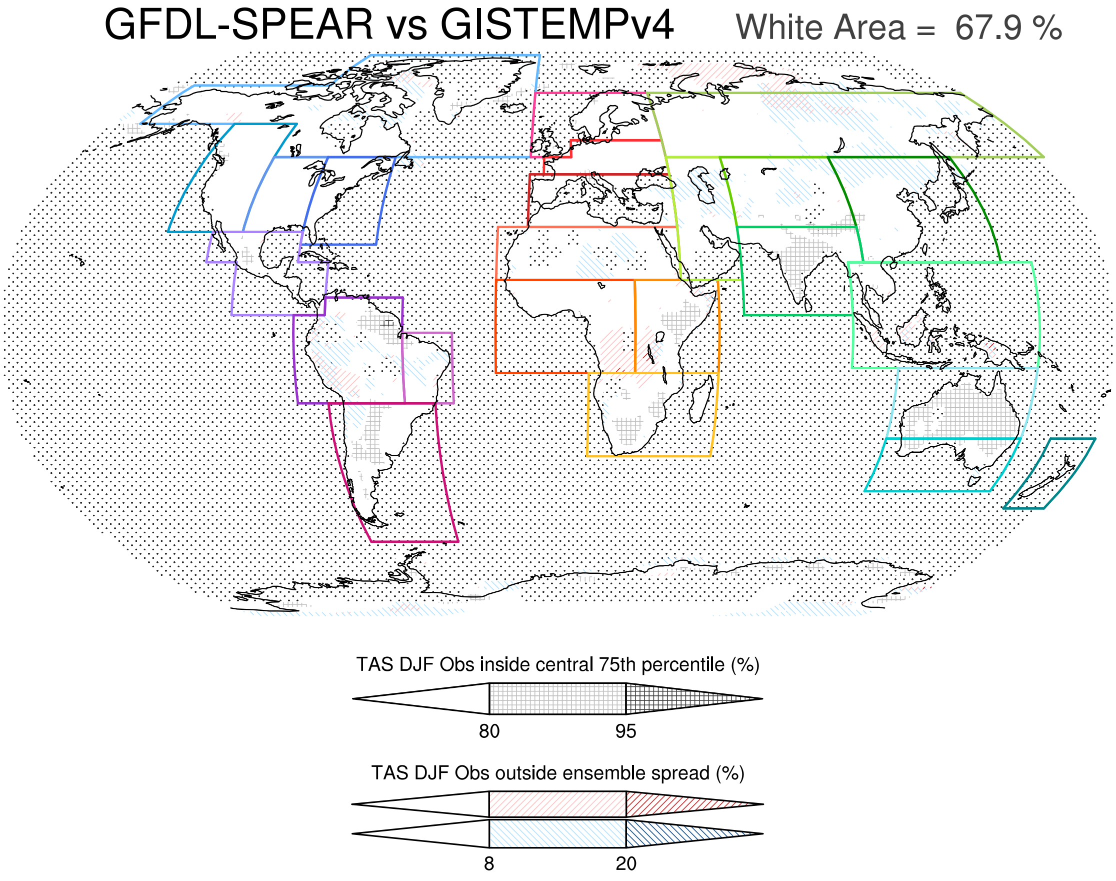

GFDL-SPEAR vs GISTEMPv4
White Area = 67.9 %
TAS DJF Obs inside central 75th percentile (%)
80
95
TAS DJF Obs outside ensemble spread (%)
8
20

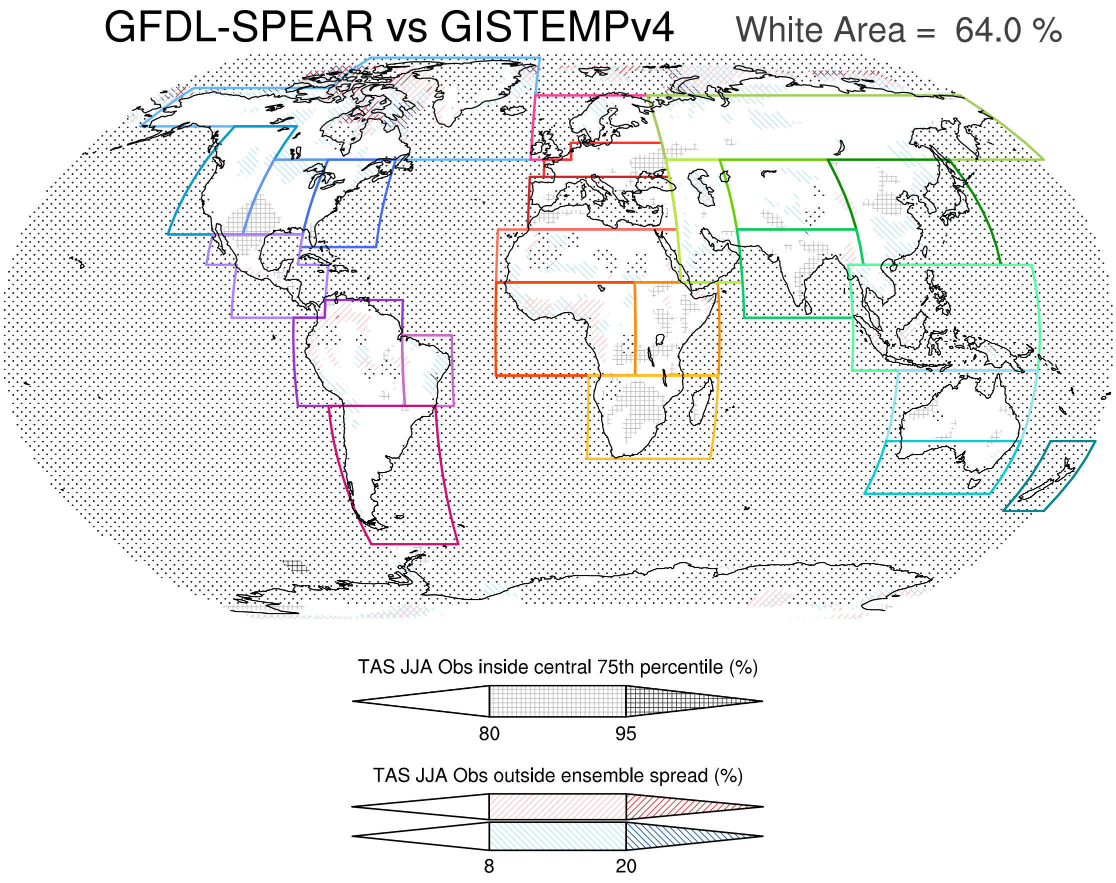

GFDL-SPEAR vs GISTEMPv4
White Area = 64.0 %
TAS JJA Obs inside central 75th percentile (%)
80
95
TAS JJA Obs outside ensemble spread (%)
8
20

GFDL-SPEAR vs GISTEMPv4 TAS DJF

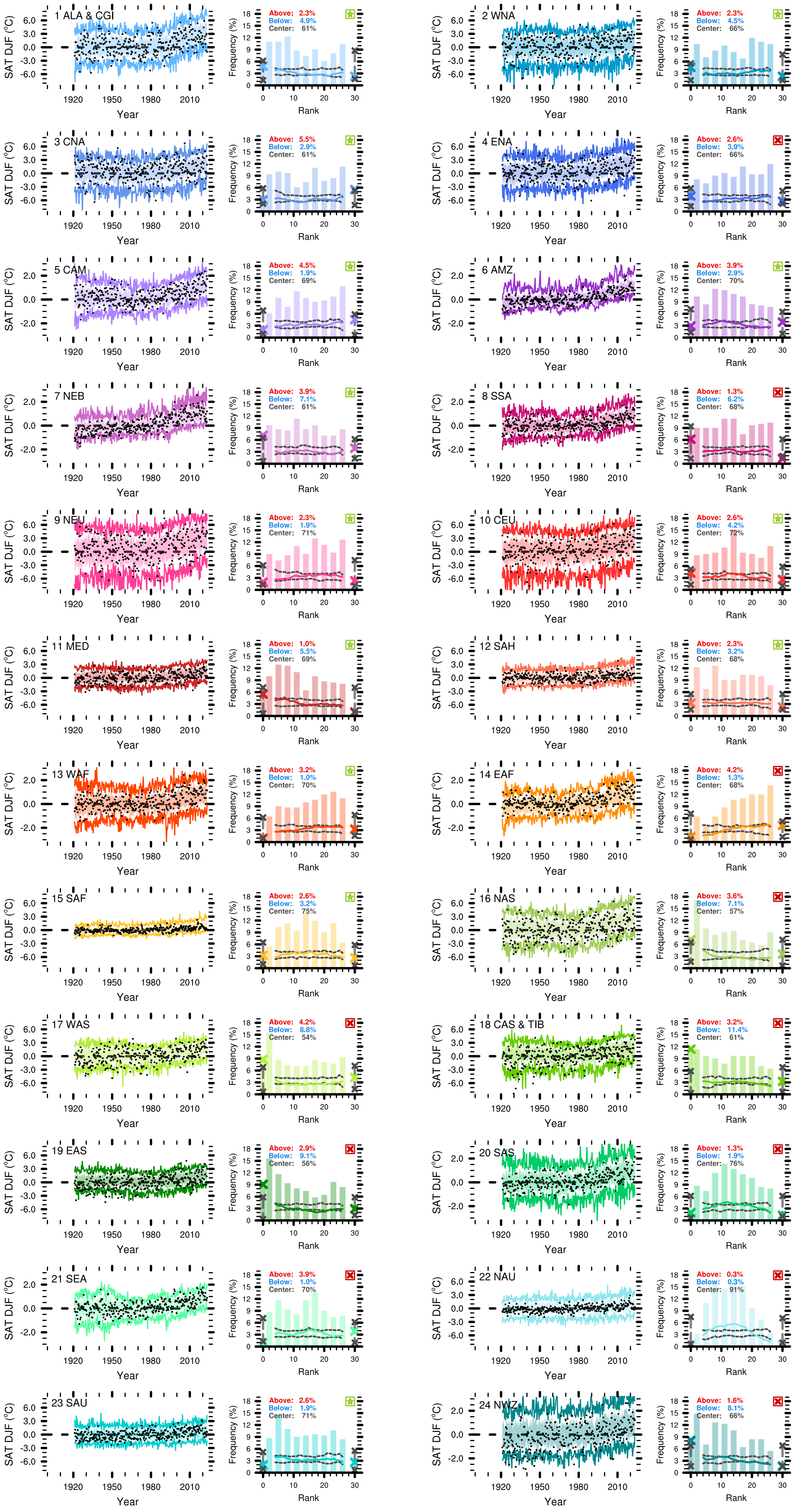

GFDL-SPEAR vs GISTEMPv4 TAS JJA

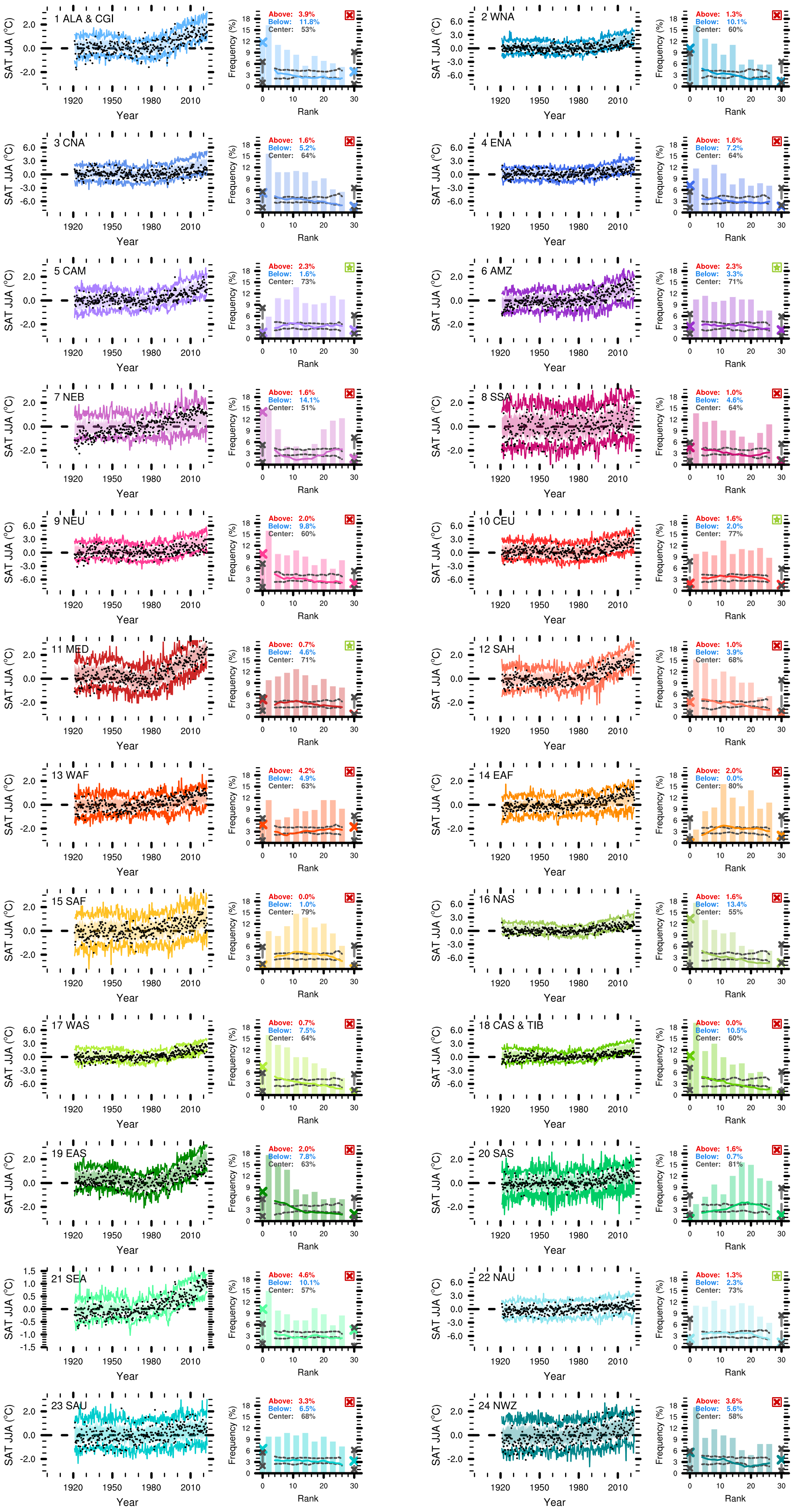

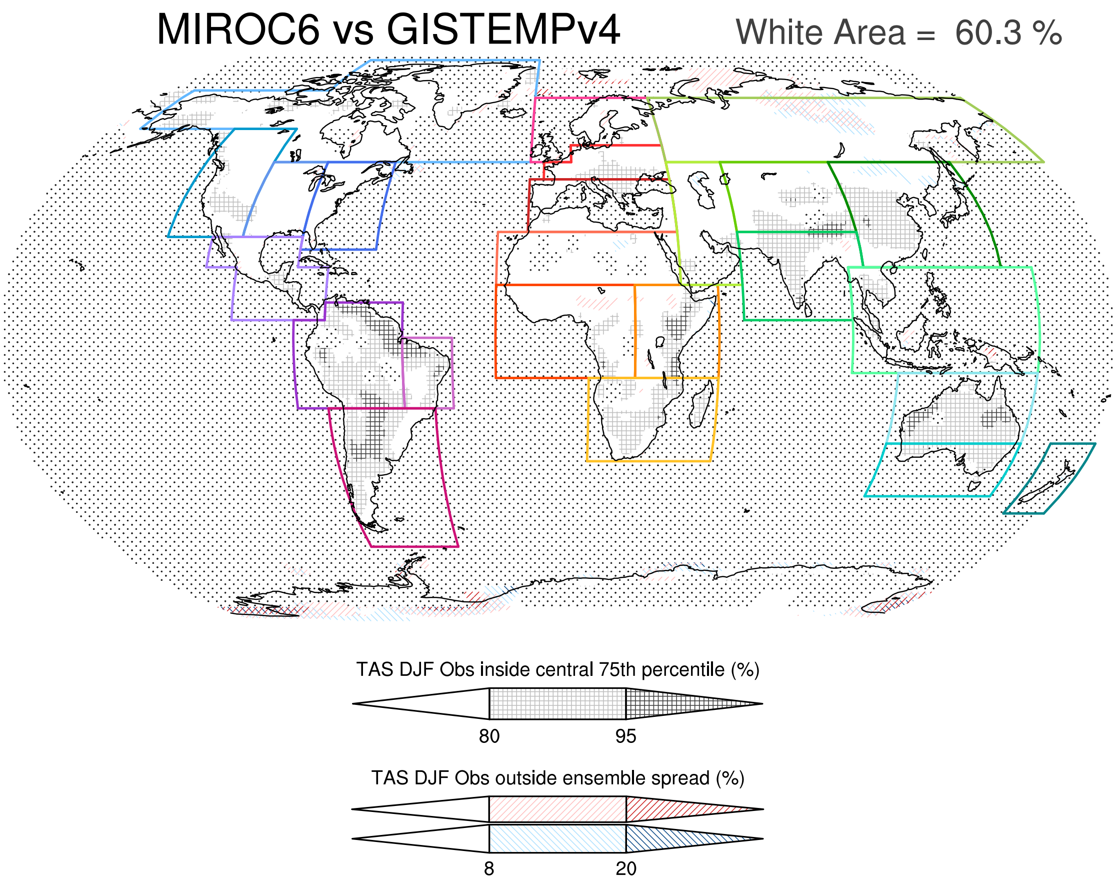
MIROC6 vs GISTEMPv4
White Area = 60.3 %
TAS DJF Obs inside central 75th percentile (%)
80
95
TAS DJF Obs outside ensemble spread (%)
8
20

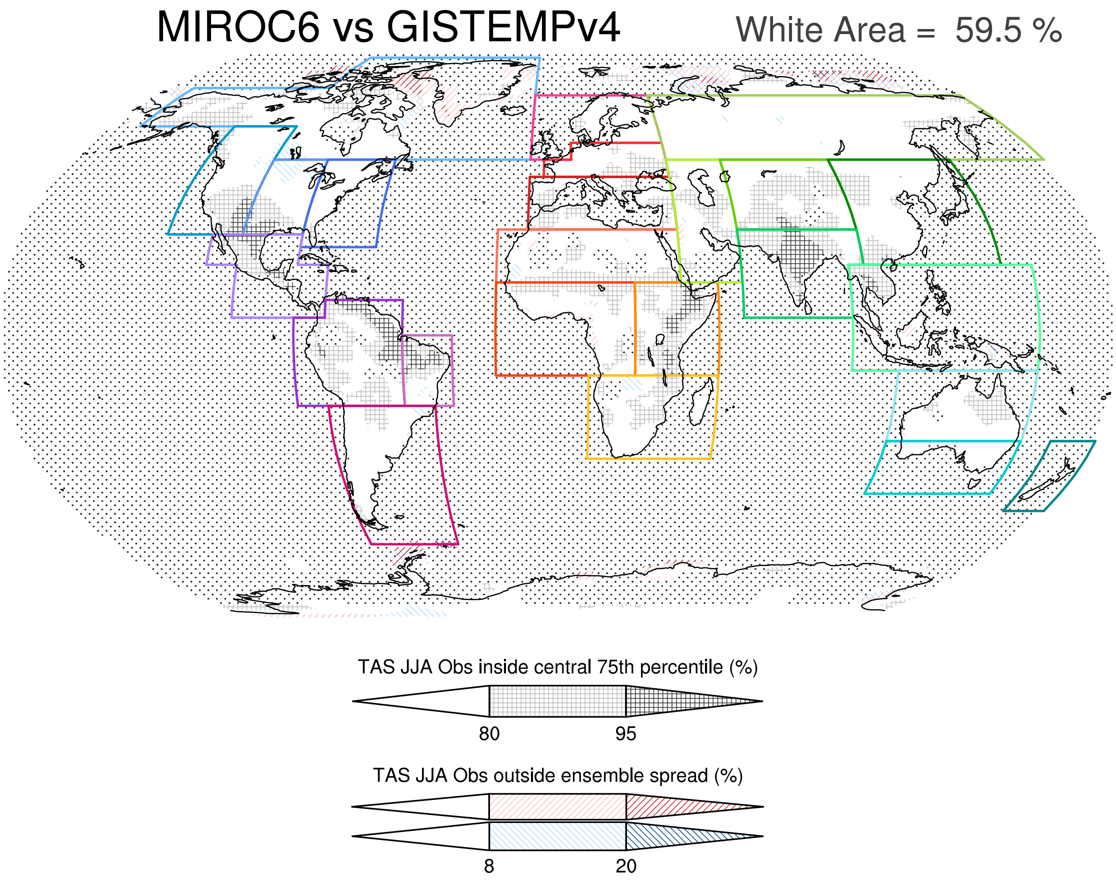
MIROC6 vs GISTEMPv4
White Area = 59.5 %
TAS JJA Obs inside central 75th percentile (%)
80
95
TAS JJA Obs outside ensemble spread (%)
8
20

MIROC6 vs GISTEMPv4 TAS DJF

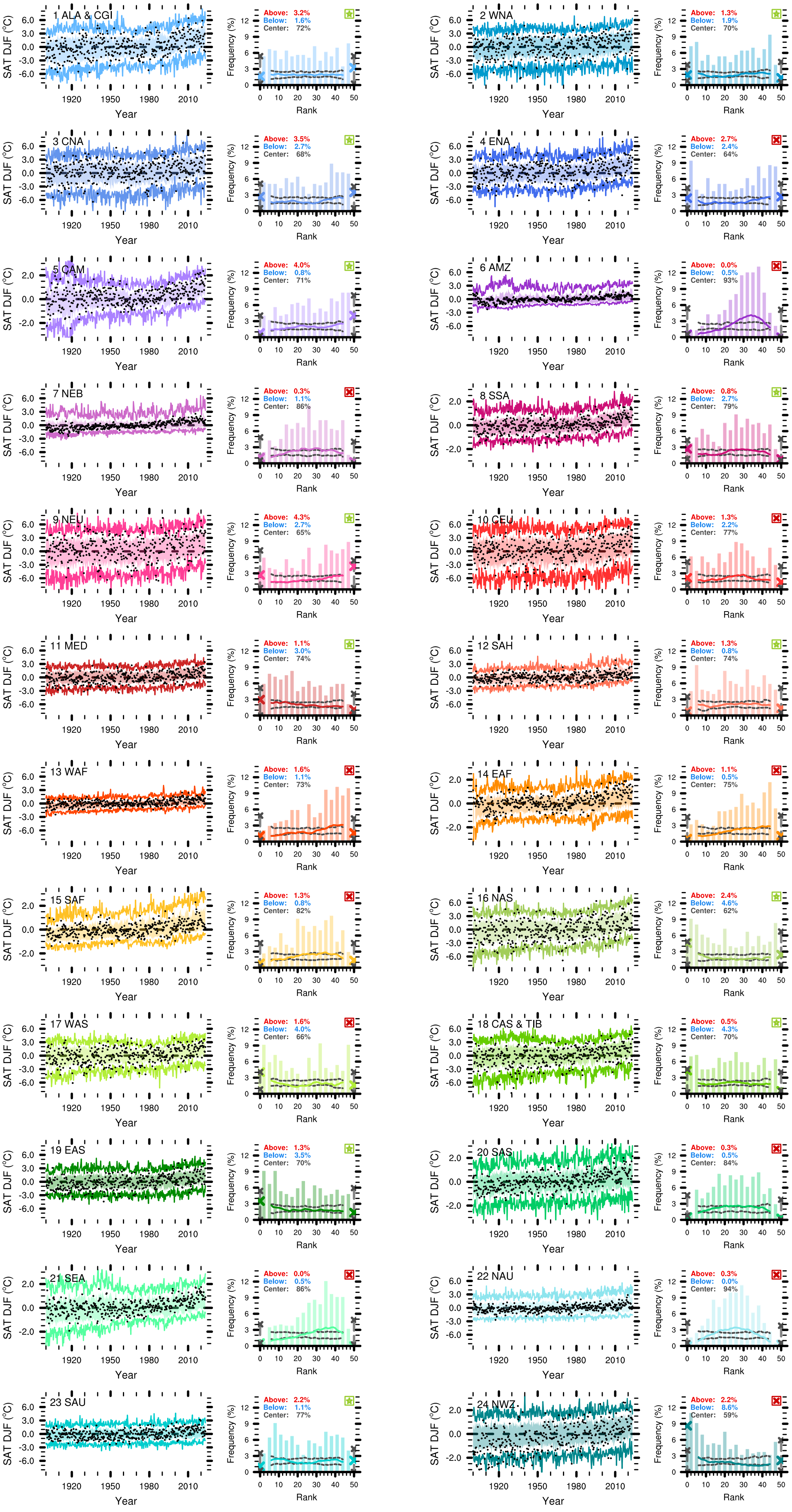

MIROC6 vs GISTEMPv4 TAS JJA

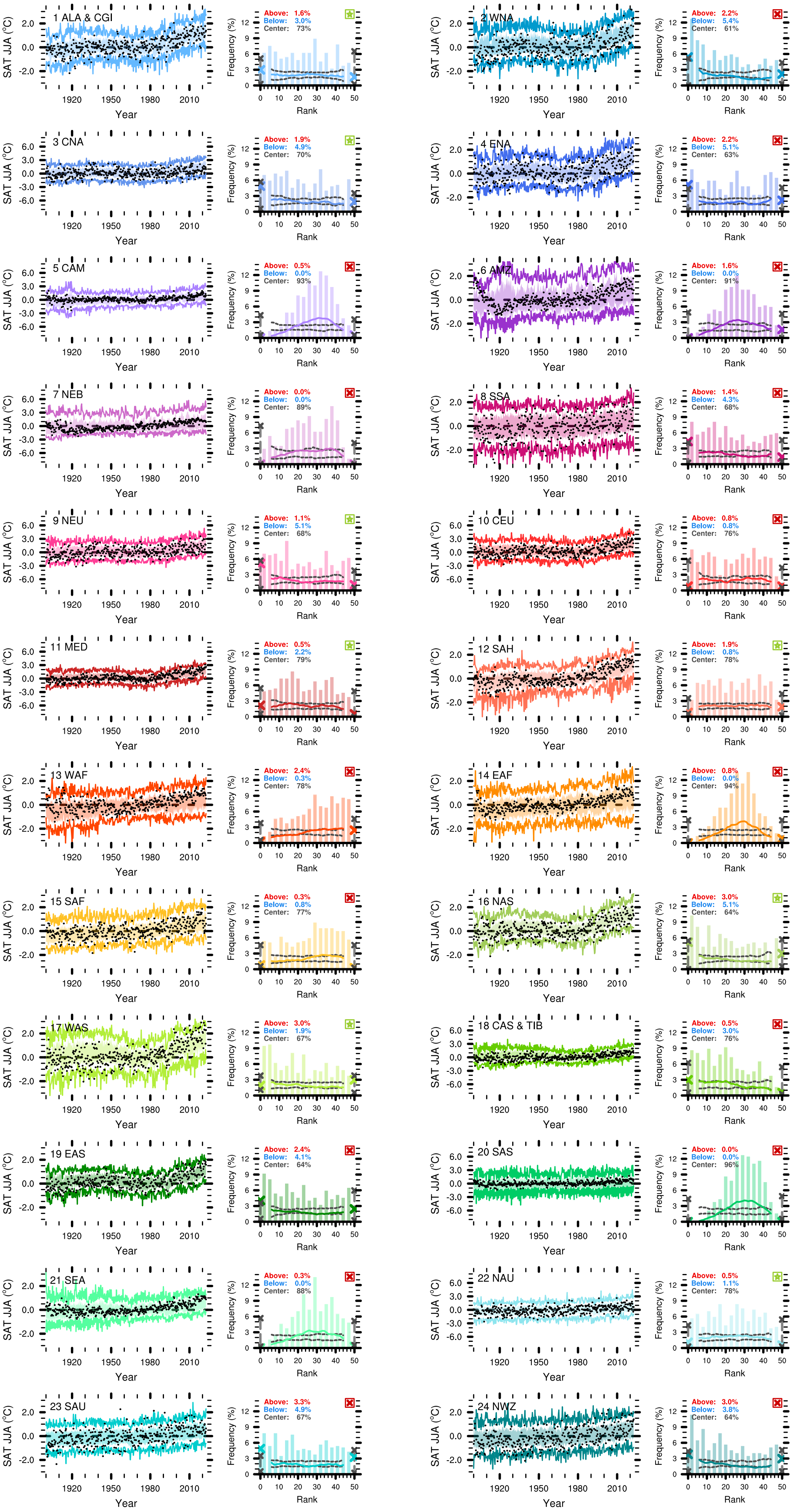

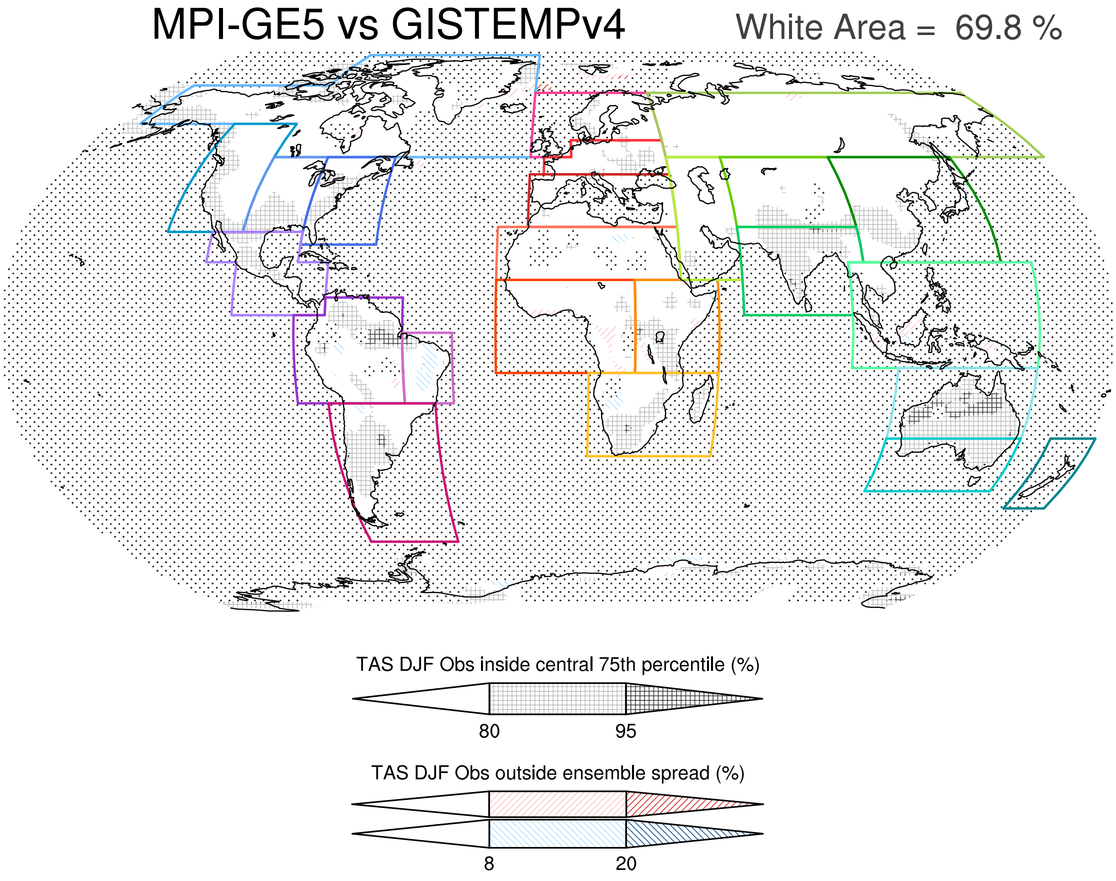

MPI-GE5 vs GISTEMPv4
White Area = 69.8 %
TAS DJF Obs inside central 75th percentile (%)
80
95
TAS DJF Obs outside ensemble spread (%)
8
20

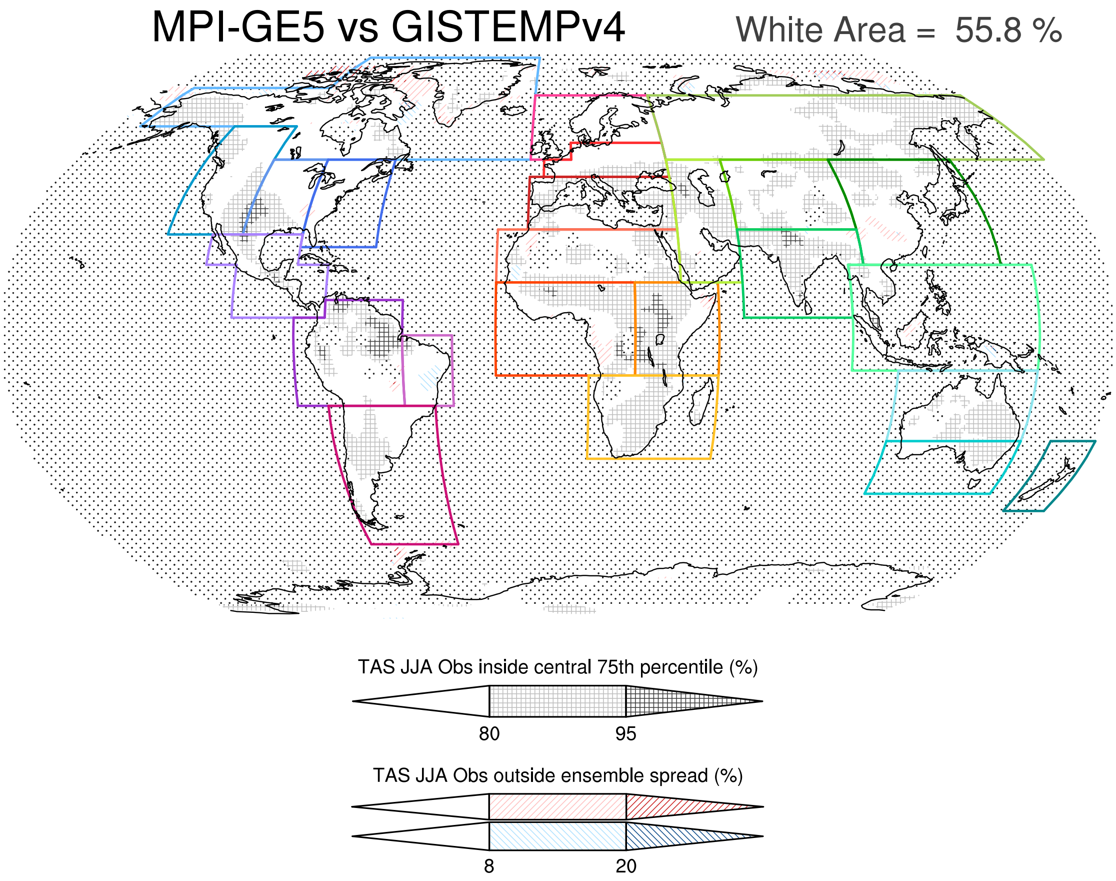

MPI-GE5 vs GISTEMPv4
White Area = 55.8 %
TAS JJA Obs inside central 75th percentile (%)
80
95
TAS JJA Obs outside ensemble spread (%)
8
20

MPI-GE5 vs GISTEMPv4 TAS DJF

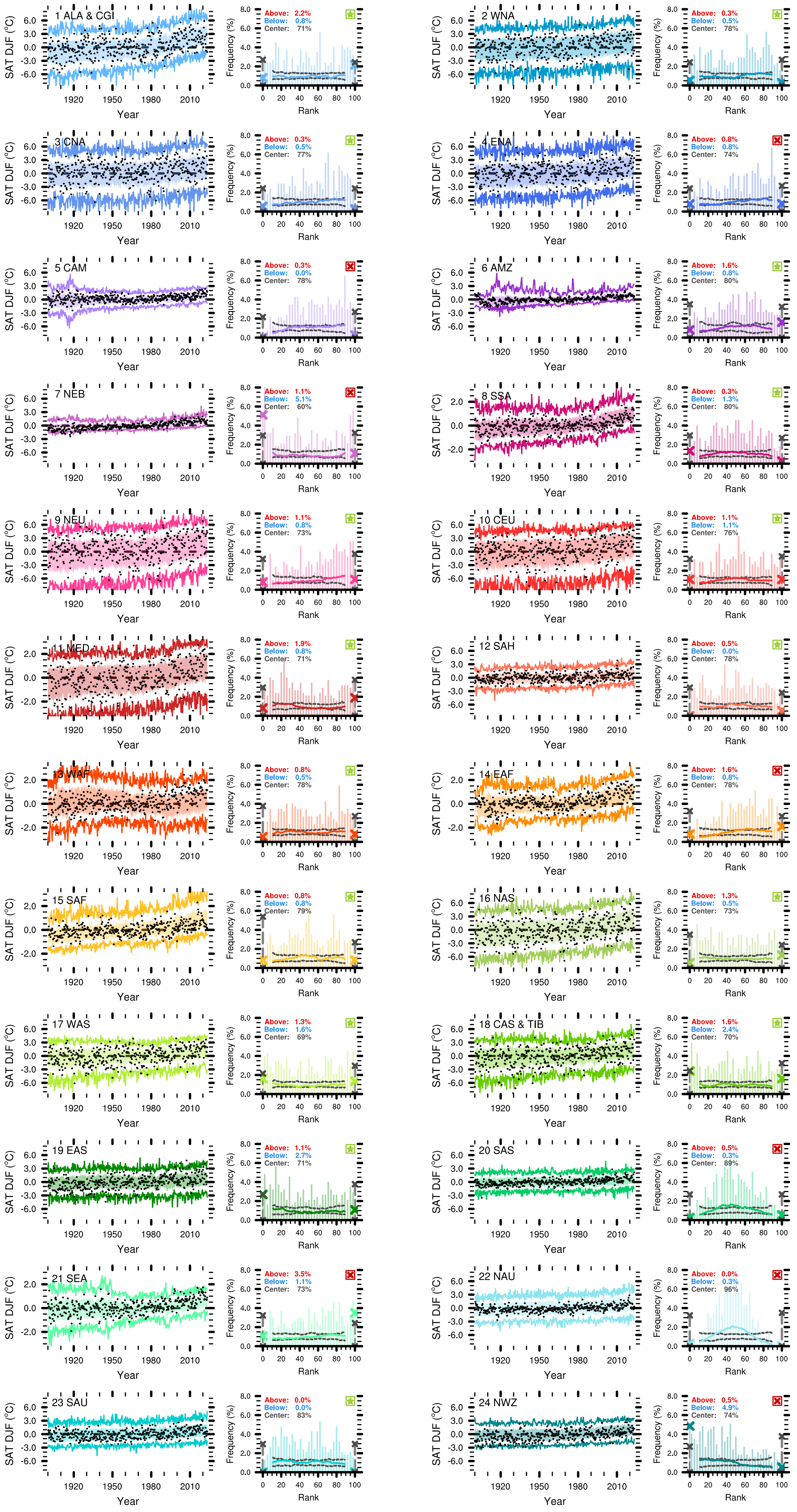

MPI-GE5 vs GISTEMPv4 TAS JJA

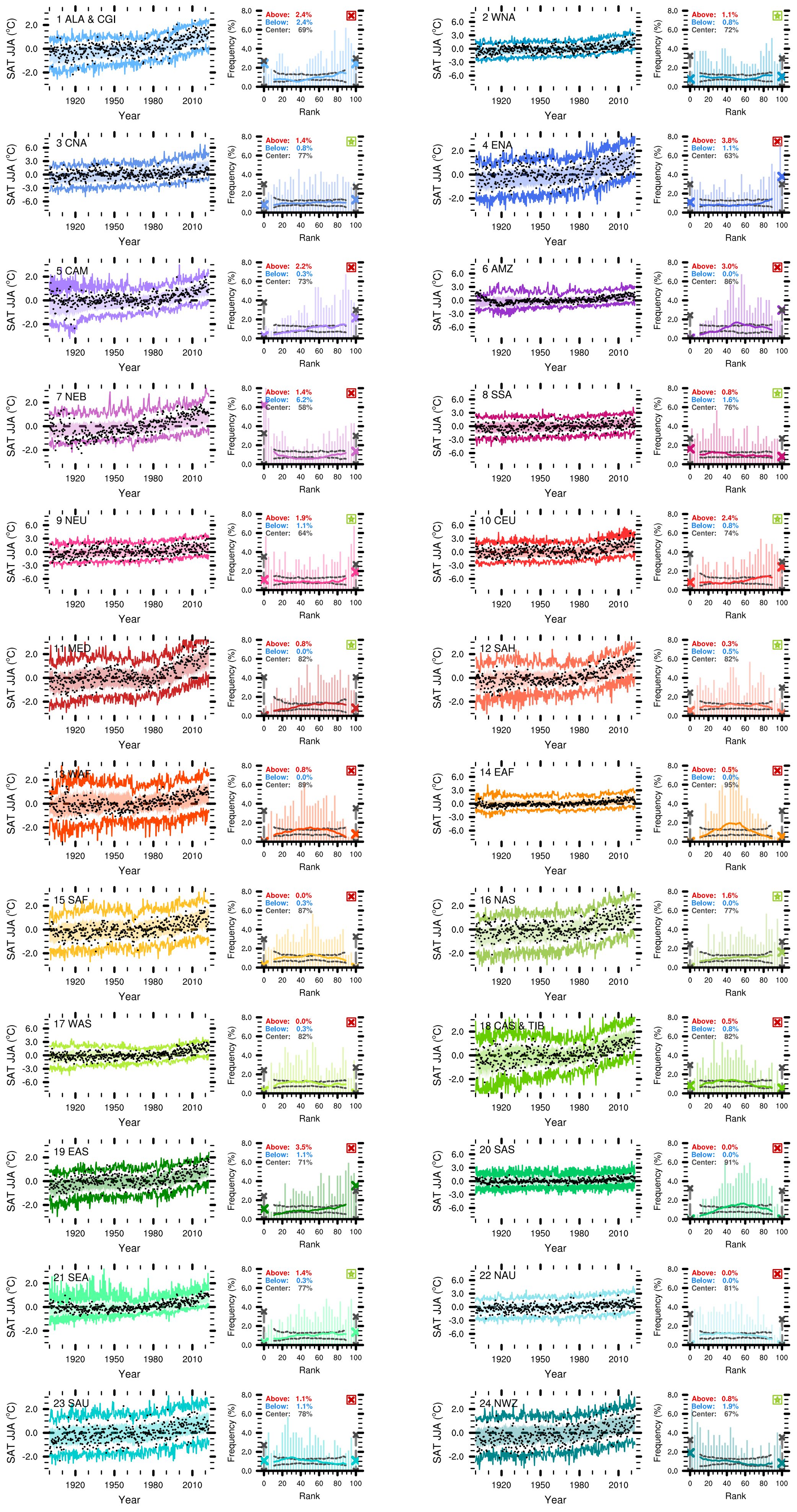

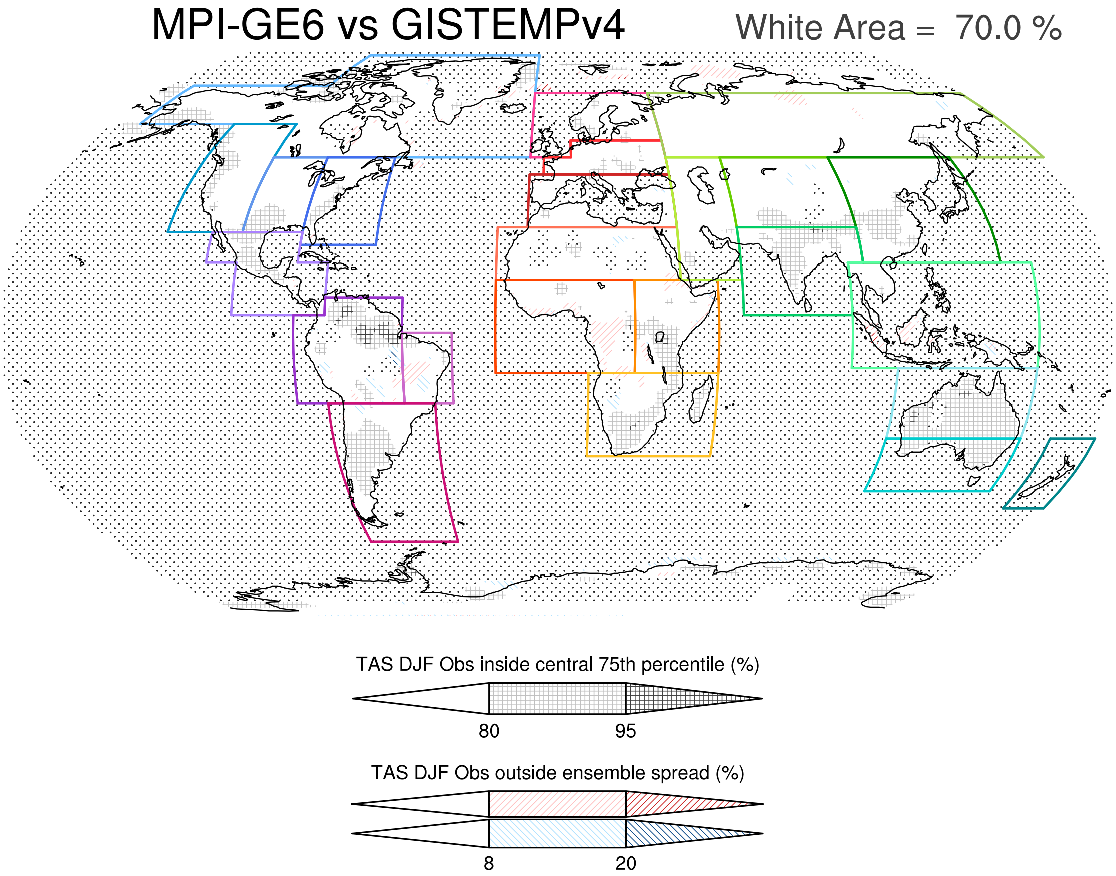

MPI-GE6 vs GISTEMPv4
White Area = 70.0 %
TAS DJF Obs inside central 75th percentile (%)
80
95
TAS DJF Obs outside ensemble spread (%)
8
20

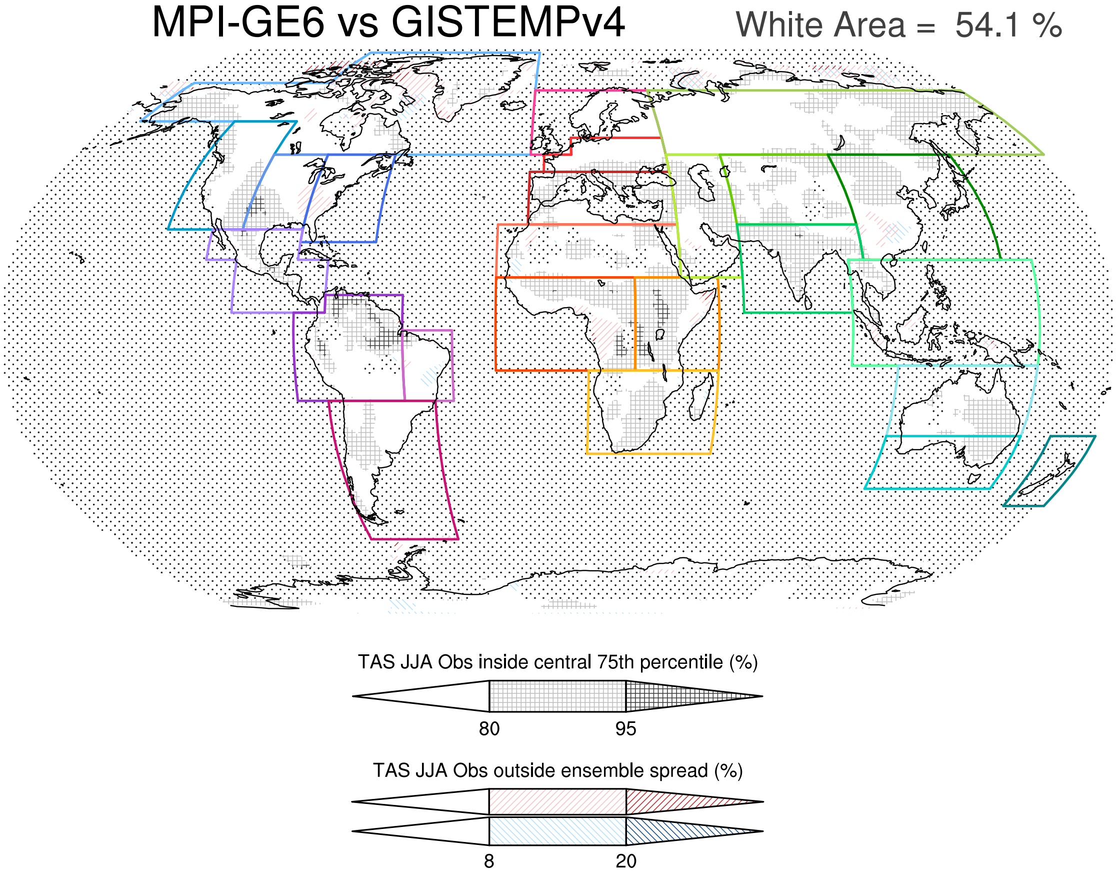

MPI-GE6 vs GISTEMPv4
White Area = 54.1 %
TAS JJA Obs inside central 75th percentile (%)
80
95
TAS JJA Obs outside ensemble spread (%)
8
20

MPI-GE6 vs GISTEMPv4 TAS DJF

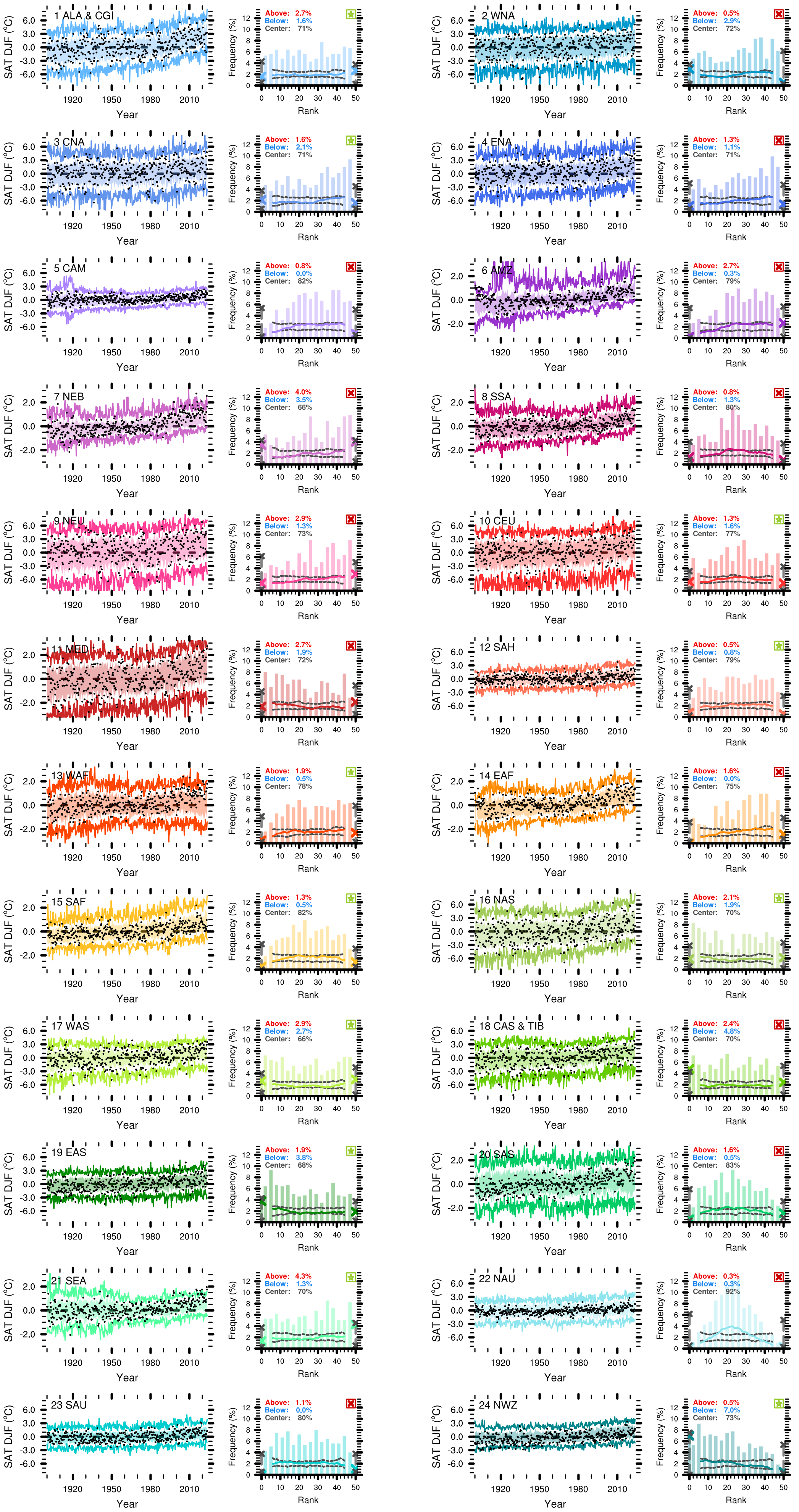

MPI-GE6 vs GISTEMPv4 TAS JJA

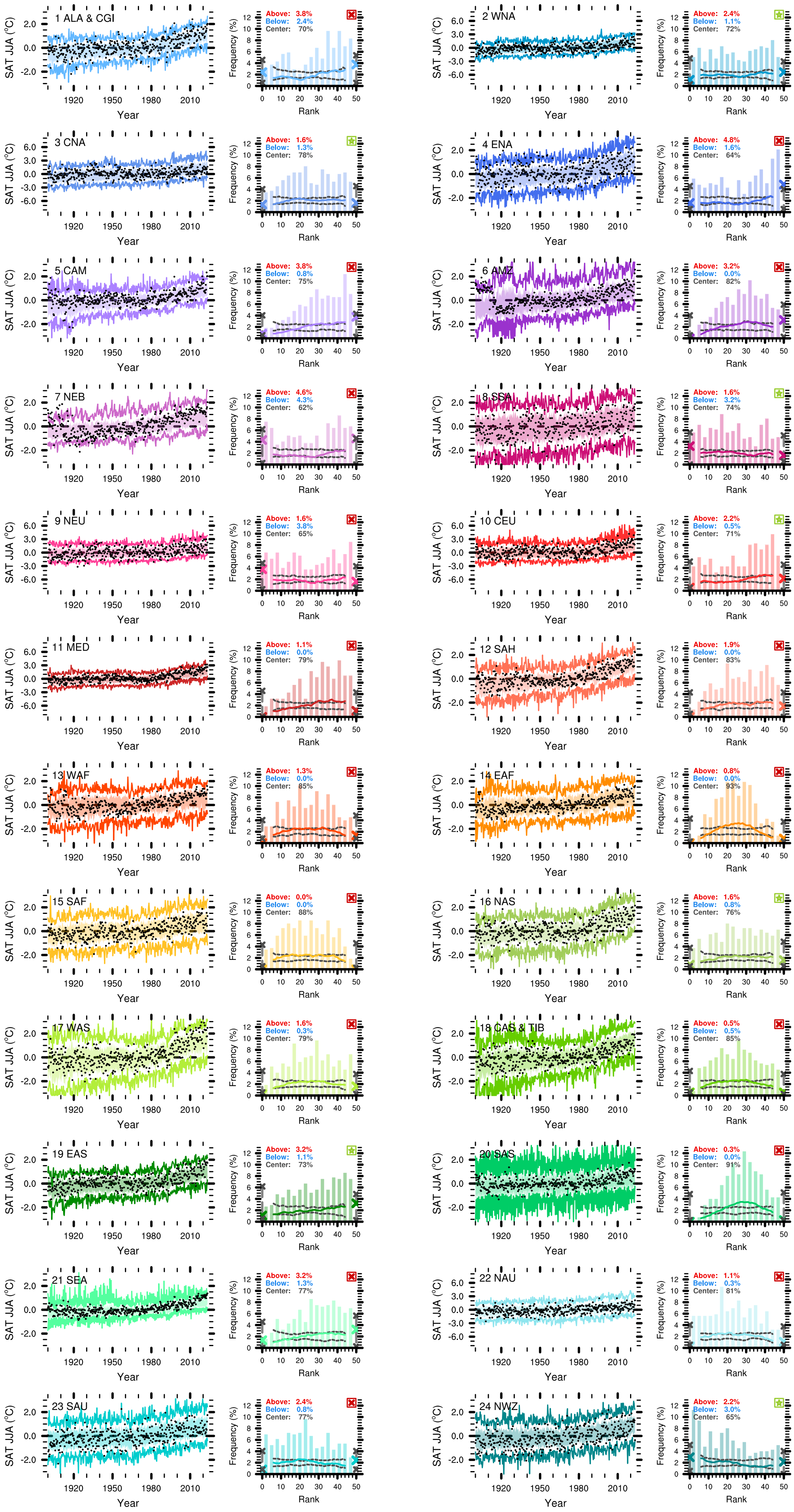

Detrended Ocean Surface Temperatures

Rank-frequency variability evaluation framework for detrended sea surface temperature (SST) anomalies over ocean grid cells.

Maps show grid-cell evaluation of the simulated DJF and JJA monthly mean temperature anomalies for the 11 SMILEs included against ERSSTv5 observations globally. Gray hatching represents where observations cluster within the 75th percentile bounds of the ensemble (12.5th to 87.5th percentiles) for more than 80% of months (light grey) or for more than 95% of months (dark grey). Red and blue shading represents where observations are larger than the ensemble maximum (red) or smaller than the ensemble minimum (blue), respectively, for more than 8% of the months (light red and blue) or for more than 20% of the months (dark red and blue). Dotted areas represent land areas or grid cells where observations are missing and are therefore excluded from this analysis. Colored boxes demark the boundaries of each ocean region assessed. The percentage of assessed grid-cells that present none of these biases, unbiased grid-cells, in given at the top (white area). For each assessed region, models are considered to provide an adequate grid-cell representation when they exhibit more than 50% of unbiased grid cells in the region, fulfilling Criteria 2 of our evaluation framework.

Time series and rank frequency histograms show spatially aggregated DJF and JJA SST for each ocean region for all 11 SMILEs. Time series show the ensemble maximum and minimum (color lines) and central 75th percentile ensemble spread (shading) are shown against observations (black dots).

Rank histograms represent the frequency of each place that observations would take in a list of ensemble members ordered by ascending temperature anomaly values. Rank histograms show the observations rank frequency accumulated for 3-rank bins (bars), the running mean rank frequency over a centered n/5 rank window (lines; for 1 to n-1 ranks) and the absolute frequencies of rank 0 and n (crosses), with n the number of ensemble members, for observations (color) and perfect model rank range (gray).

To illustrate how internal variability sampling may affect rank frequencies given the non-infinite record length considered, we include a comparison to the perfect-model rank range, which shows the range of rank frequencies that each ensemble member would take if it were observations (see Methods in main article for further details). If the rank exhibited by observations (colors) is within this perfect-model range (gray; allowing a maximum 10% deviation outside of this range), the rank frequency evaluation shows an adequate model performance, and any deviations from a perfectly frank rank histogram can be assumed to be within the range of deviations that could be caused by internal variability. In the case the observations rank frequency is within the perfect-model range for all rank windows, Criteria 1 of our evaluation framework is met and this is highlighted by a green star at the top right; if not, by a red cross. Percentages at the top left show the frequency of regionally averaged observations occurring above (red) or below (blue) ensemble limits, or clustering within the central 75th percentile range (gray), analogous to the grid-cell evaluation in Criteria 2.

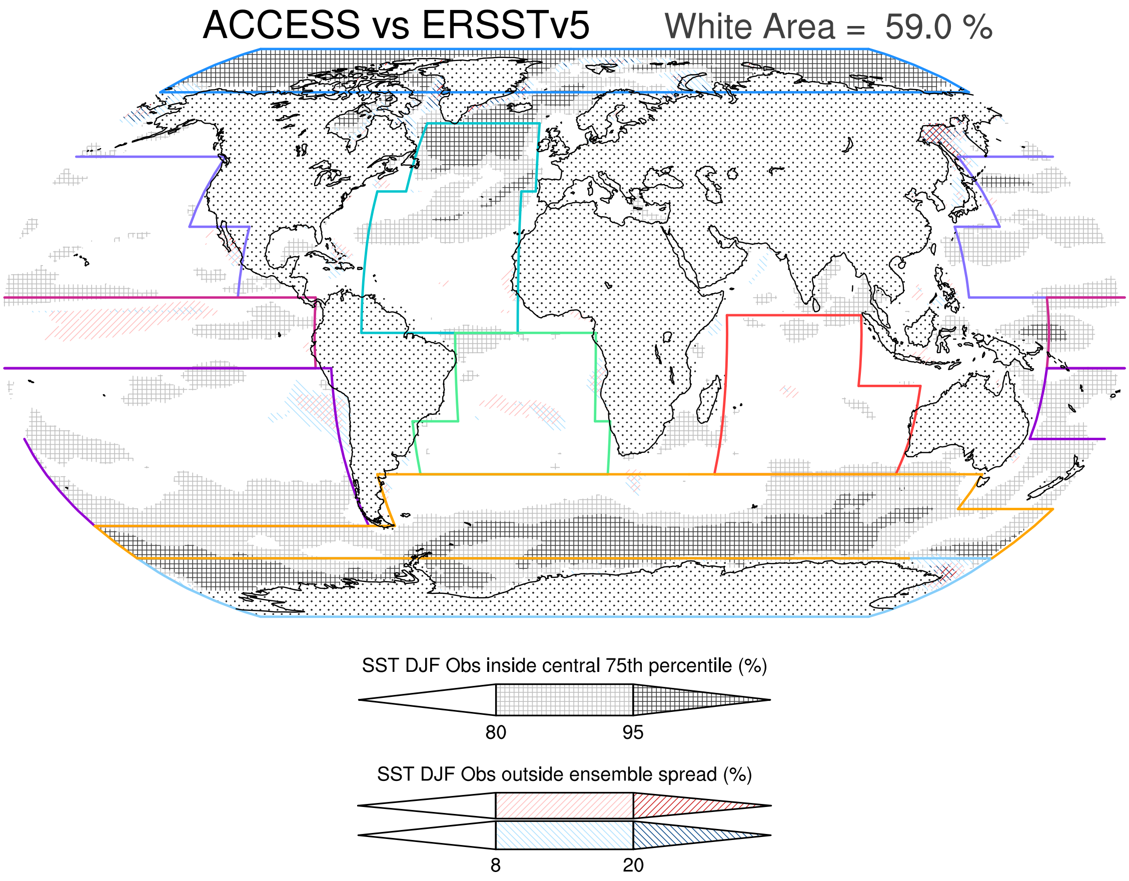
ACCESS vs ERSSTv5
White Area = 59.0 %
SST DJF Obs inside central 75th percentile (%)
80
95
SST DJF Obs outside ensemble spread (%)
8
20

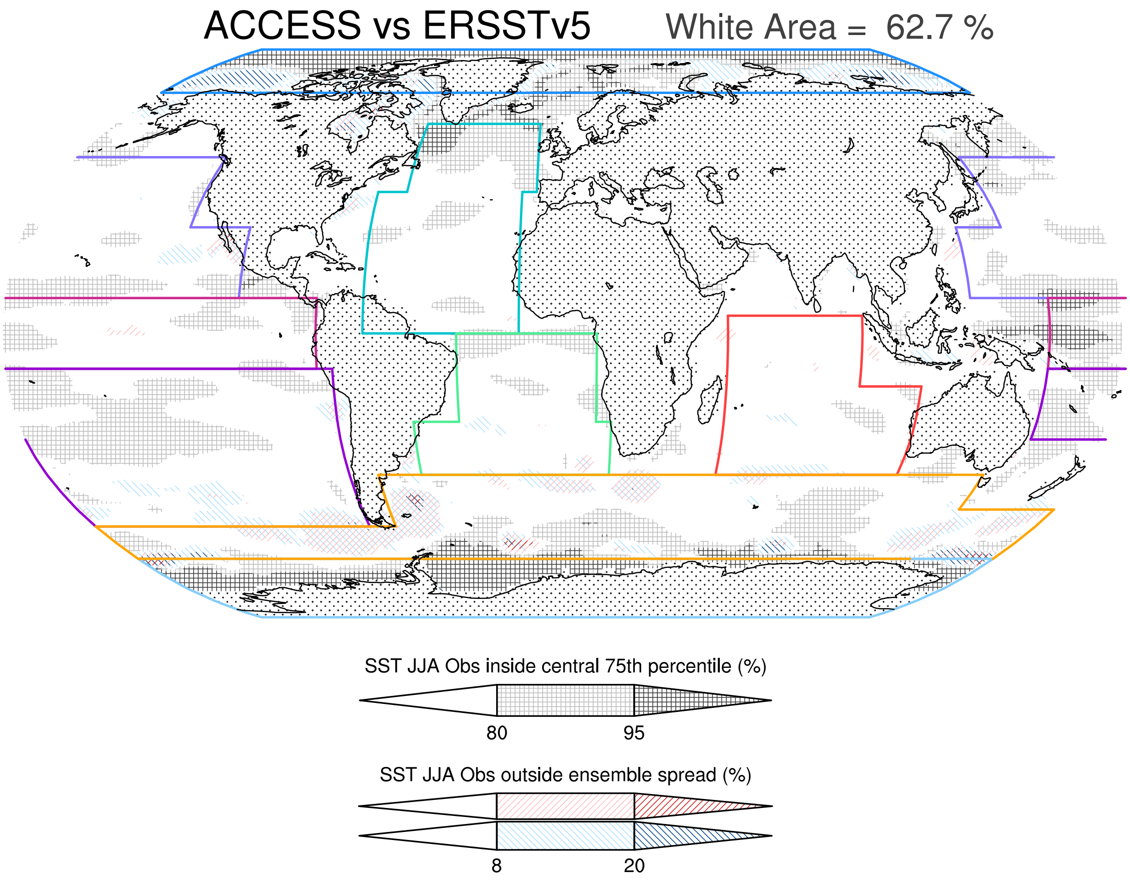
ACCESS vs ERSSTv5
White Area = 62.7 %
SST JJA Obs inside central 75th percentile (%)
80
95
SST JJA Obs outside ensemble spread (%)
8
20

ACCESS vs ERSSTv5 SST DJF

ACCESS vs ERSSTv5 SST JJA

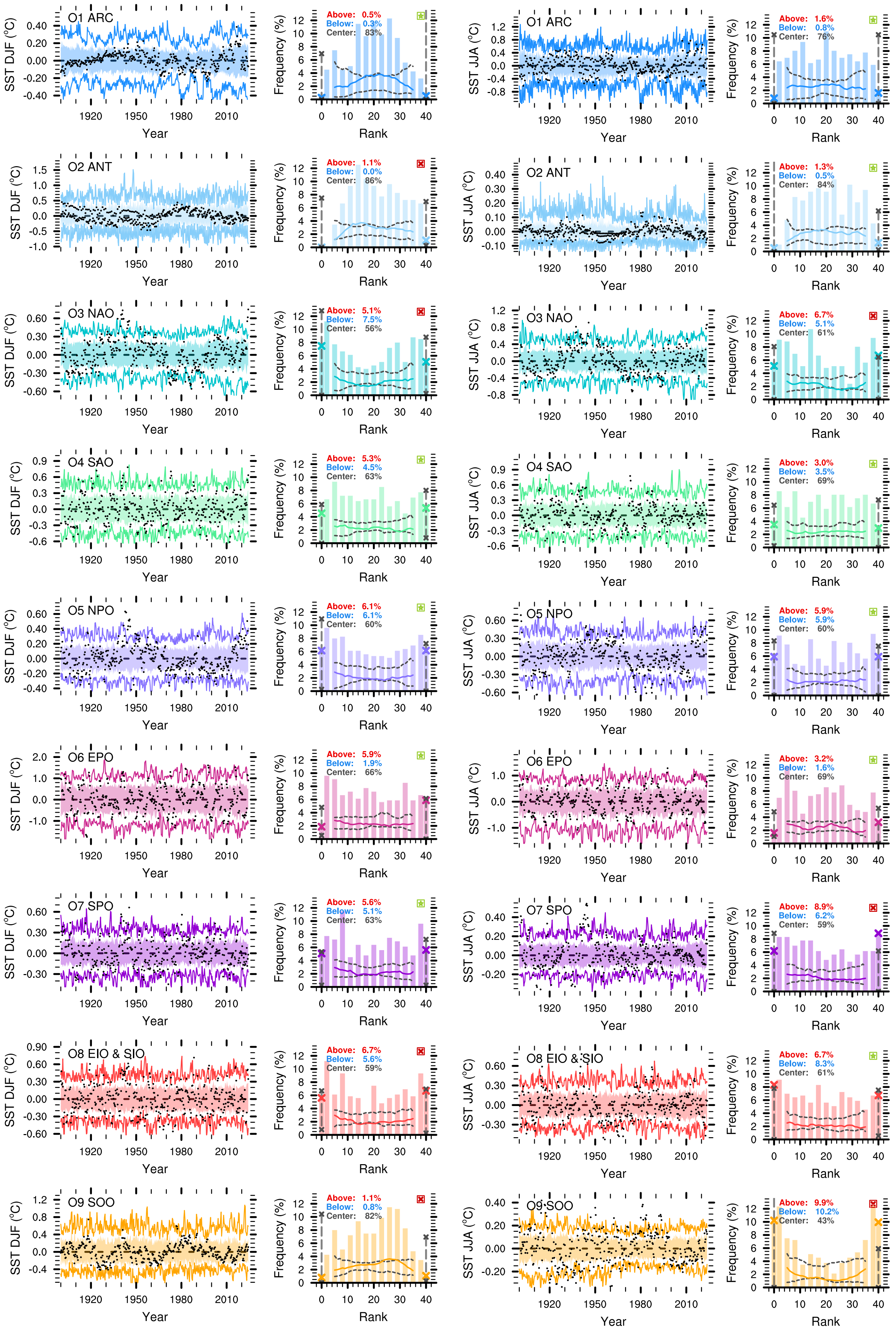

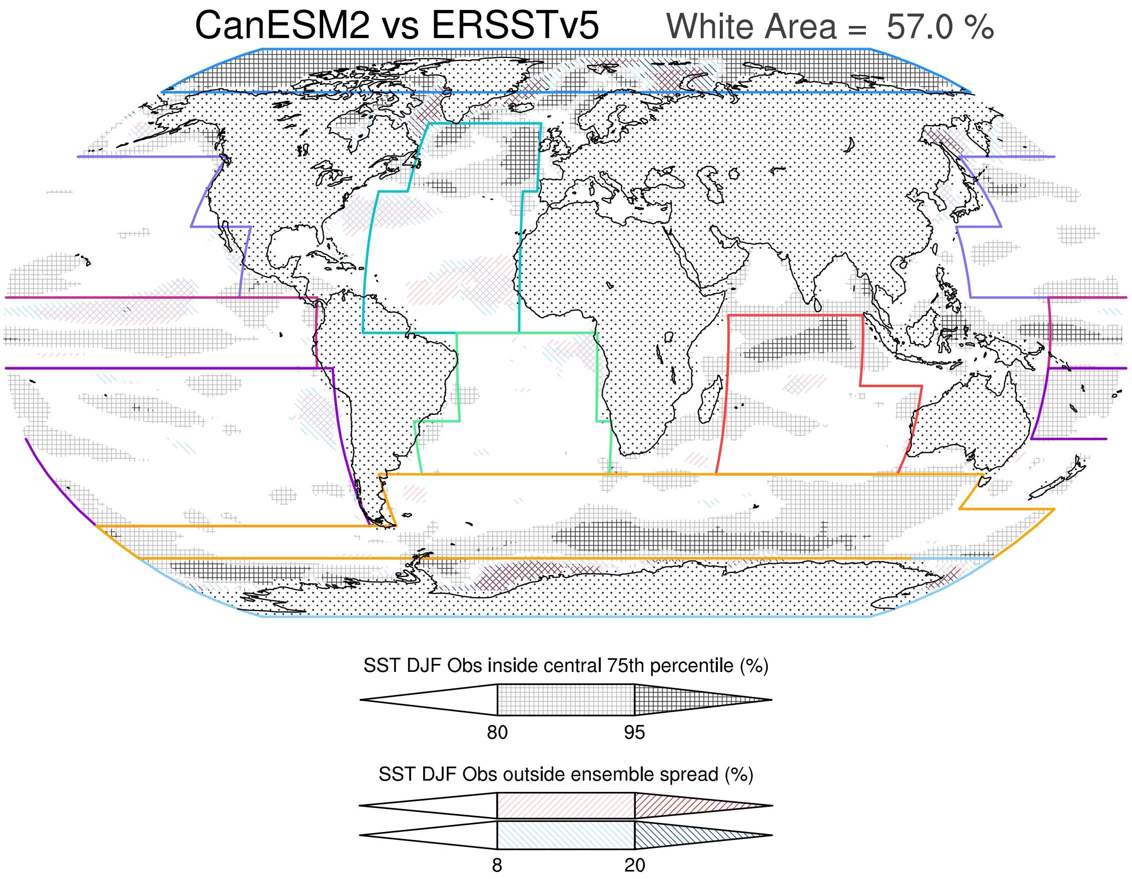
CanESM2 vs ERSSTv5
White Area = 57.0 %
SST DJF Obs inside central 75th percentile (%)
80
95
SST DJF Obs outside ensemble spread (%)
8
20

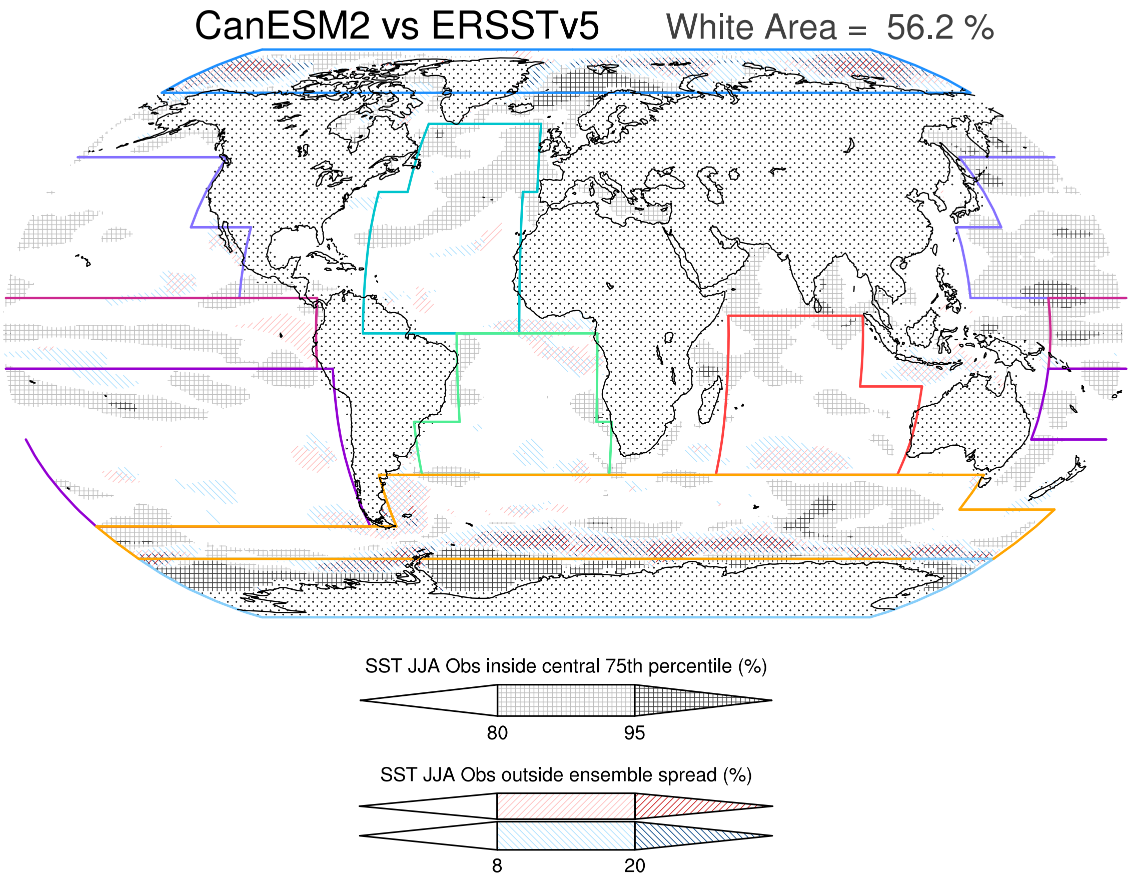
CanESM2 vs ERSSTv5
White Area = 56.2 %
SST JJA Obs inside central 75th percentile (%)
80
95
SST JJA Obs outside ensemble spread (%)
8
20

CanESM2 vs ERSSTv5 SST DJF

CanESM2 vs ERSSTv5 SST JJA

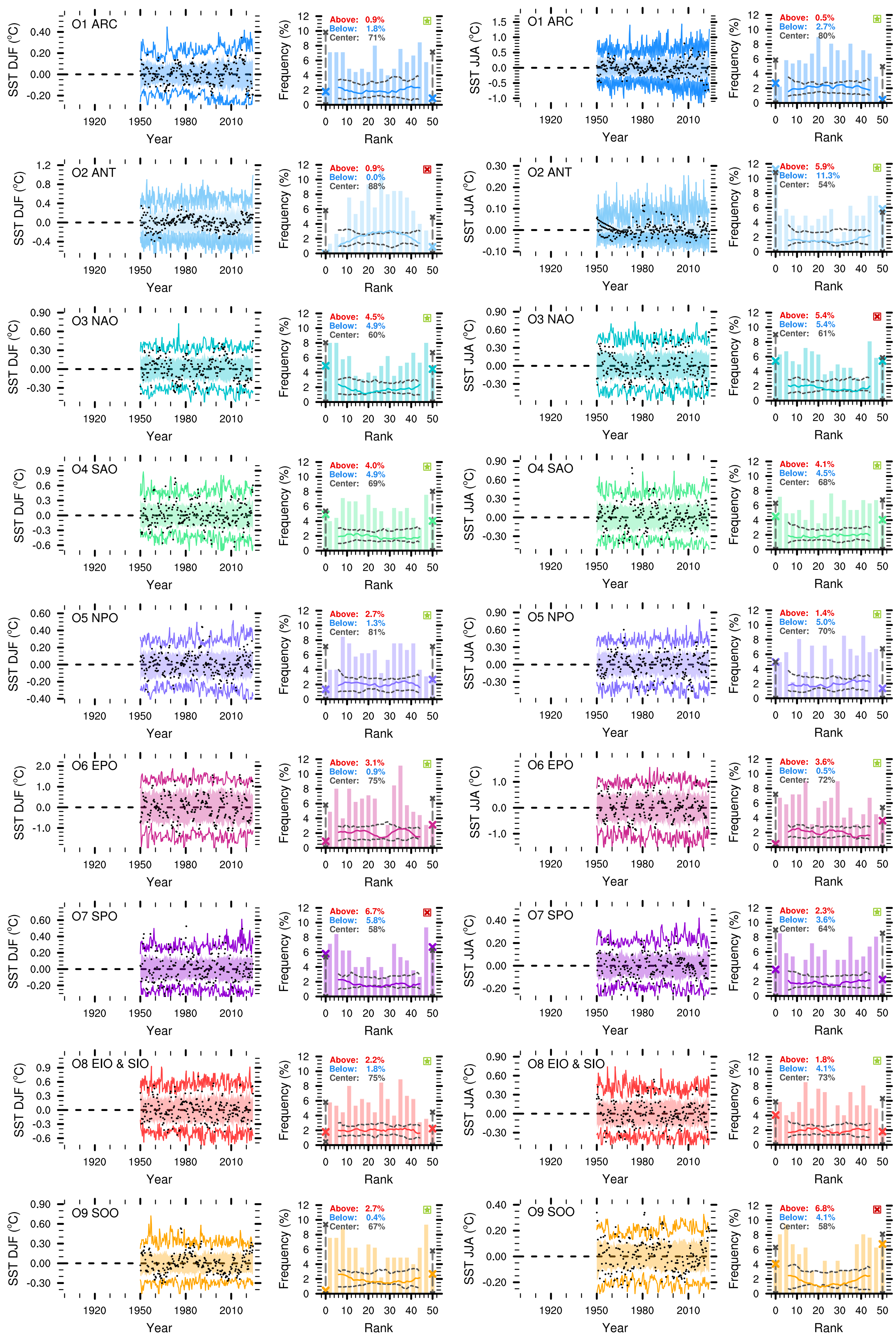

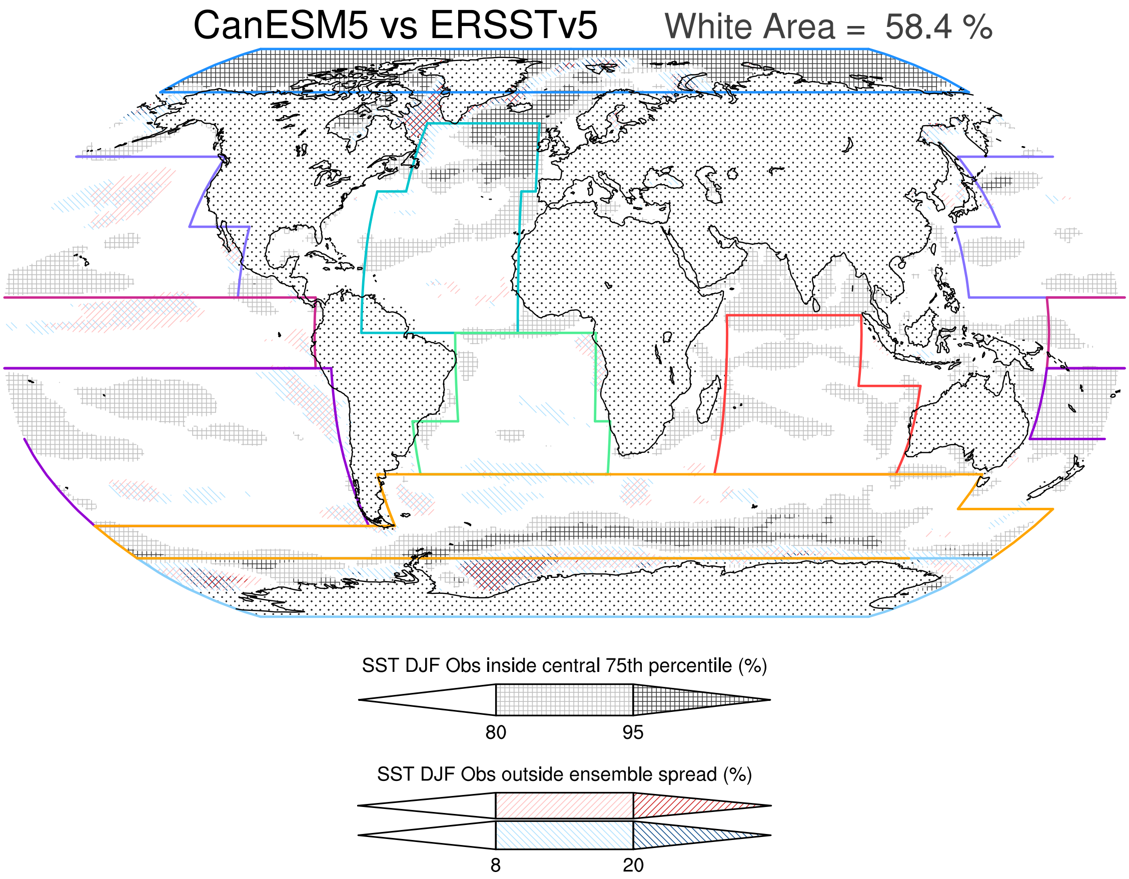
CanESM5 vs ERSSTv5
White Area = 58.4 %
SST DJF Obs inside central 75th percentile (%)
80
95
SST DJF Obs outside ensemble spread (%)
8
20

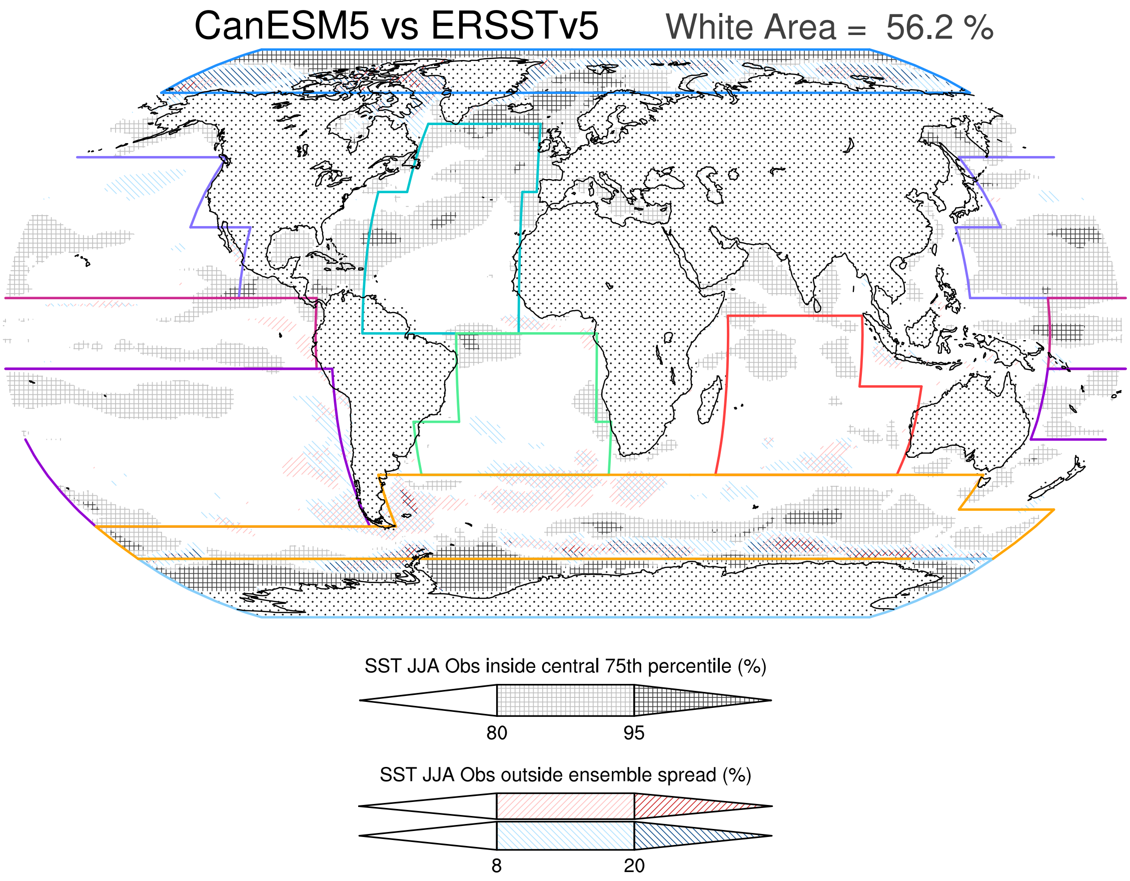
CanESM5 vs ERSSTv5
White Area = 56.2 %
SST JJA Obs inside central 75th percentile (%)
80
95
SST JJA Obs outside ensemble spread (%)
8
20

CanESM5 vs ERSSTv5 SST DJF

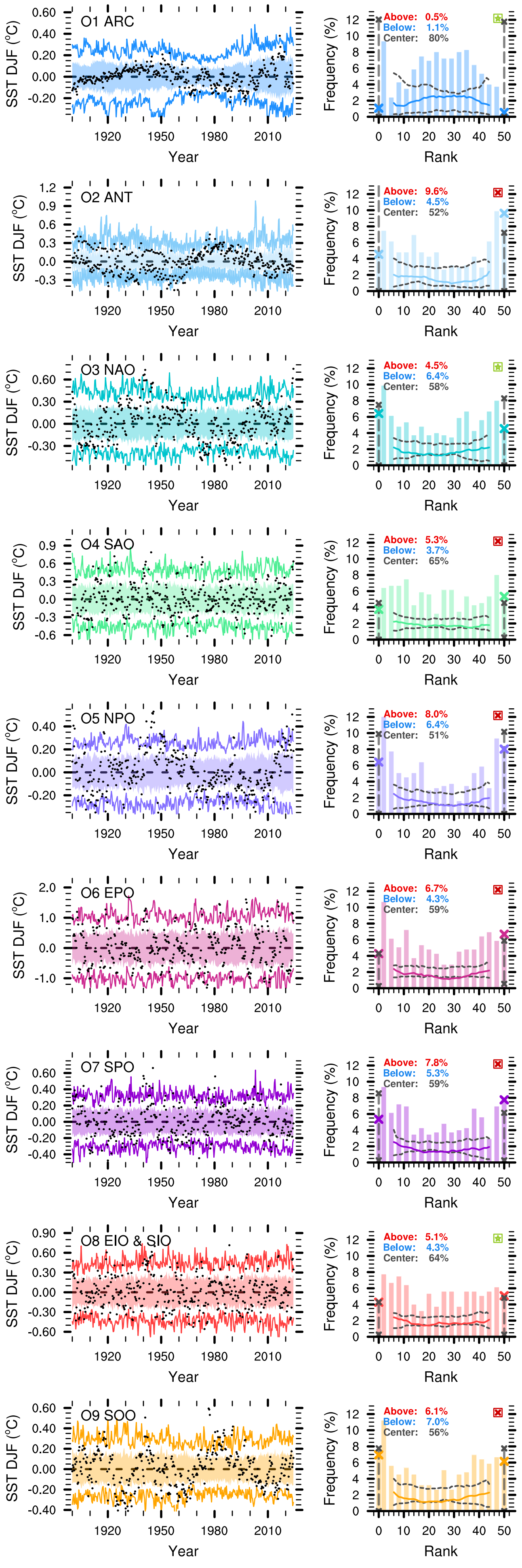

CanESM5 vs ERSSTv5 SST JJA

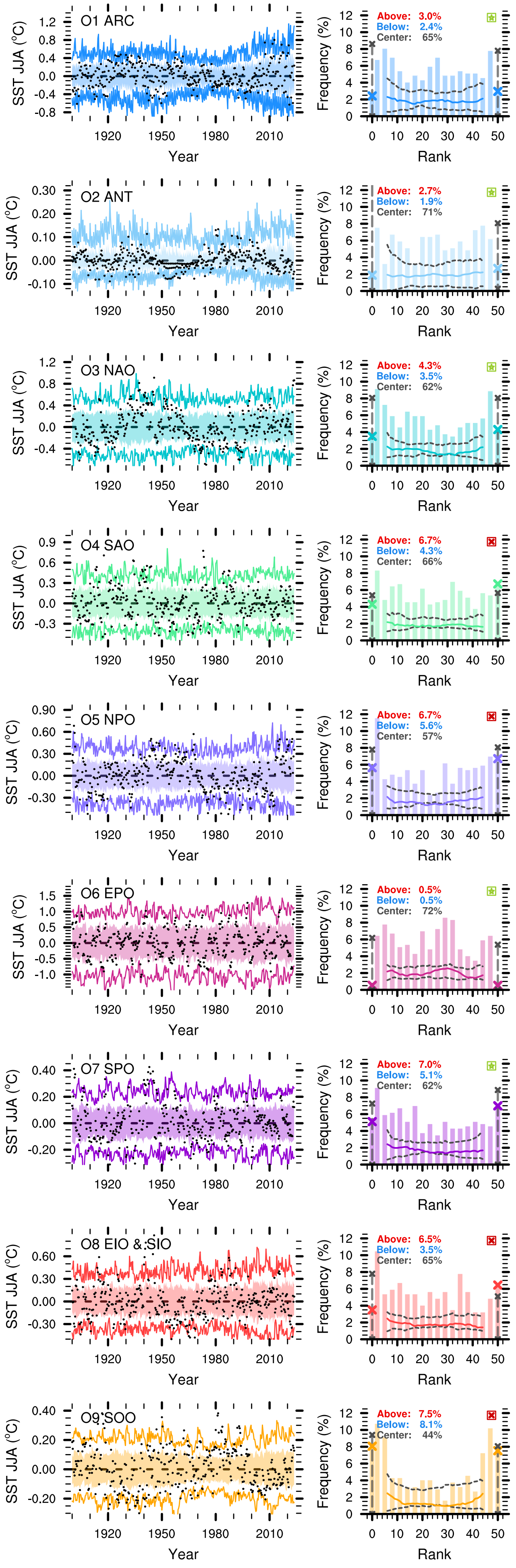

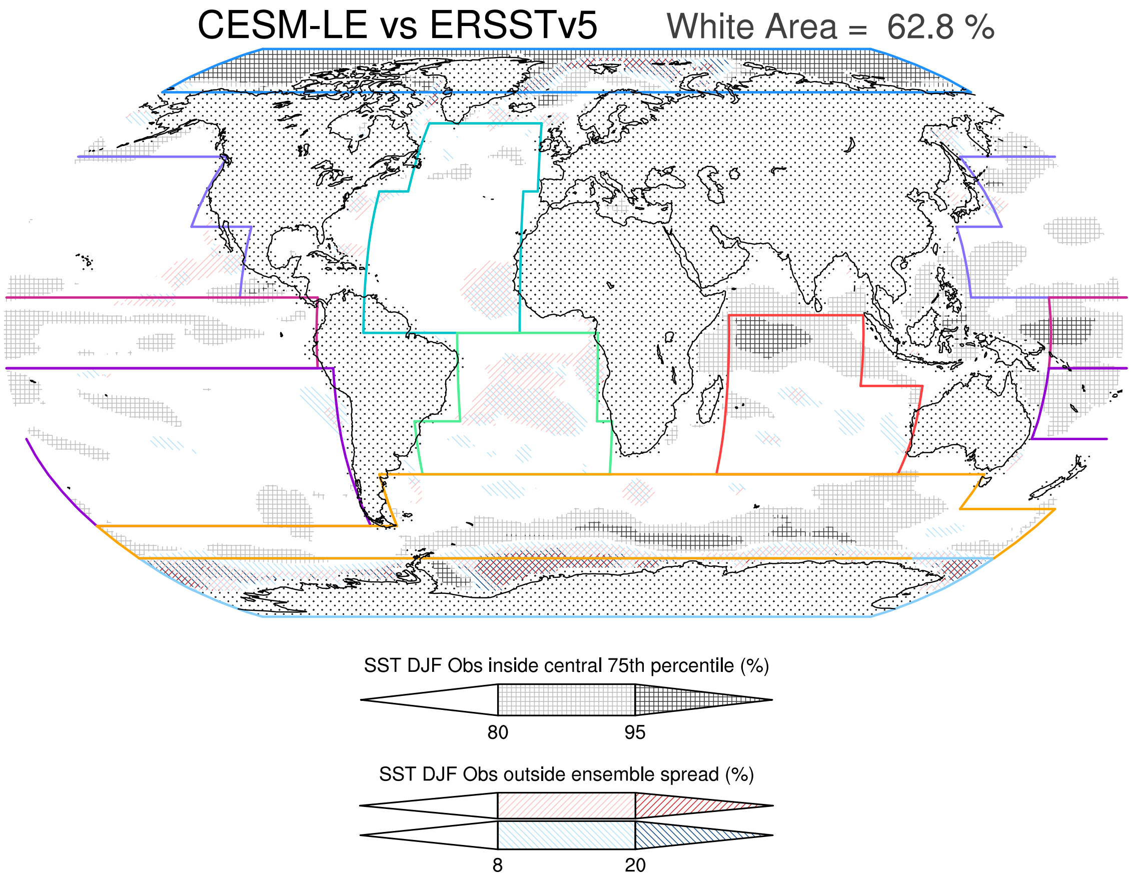
CESM-LE vs ERSSTv5
White Area = 62.8 %
SST DJF Obs inside central 75th percentile (%)
80
95
SST DJF Obs outside ensemble spread (%)
8
20

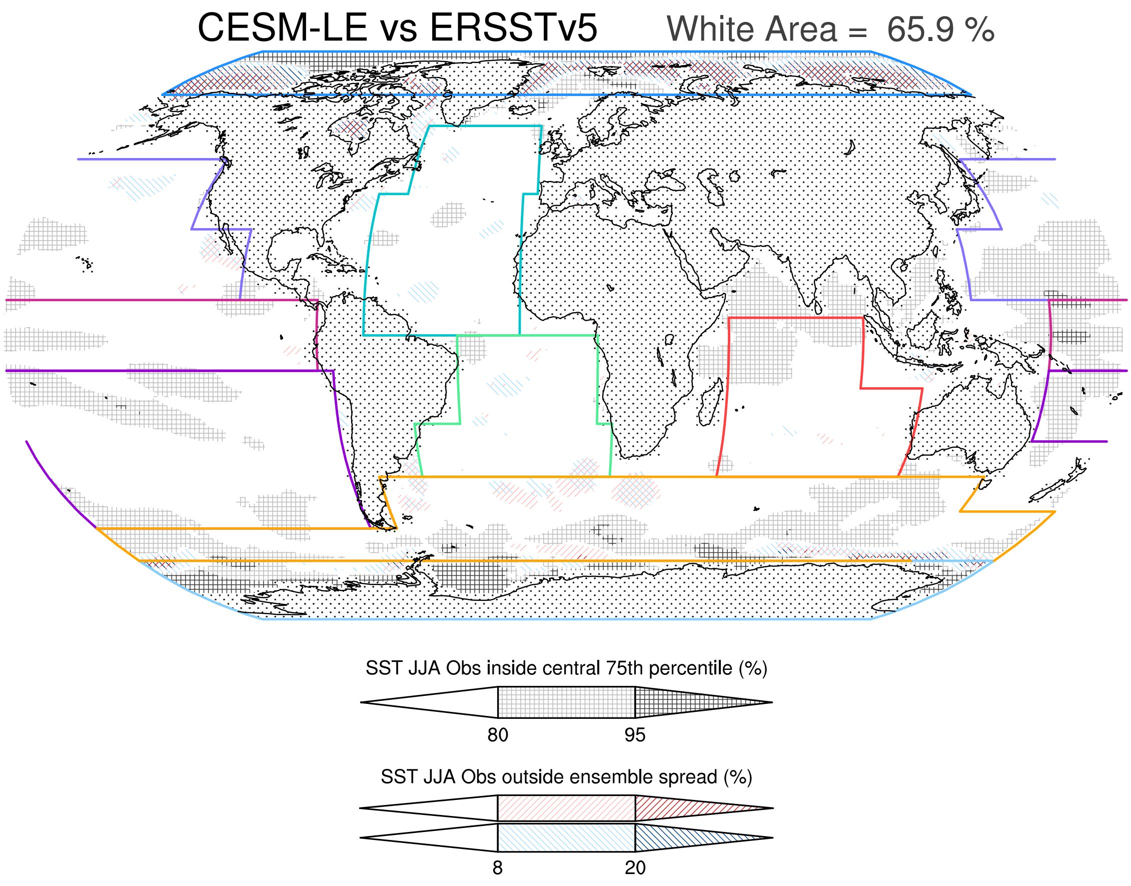
CESM-LE vs ERSSTv5
White Area = 65.9 %
SST JJA Obs inside central 75th percentile (%)
80
95
SST JJA Obs outside ensemble spread (%)
8
20

CESM-LE vs ERSSTv5 SST DJF   CESM-LE vs ERSSTv5 SST JJA

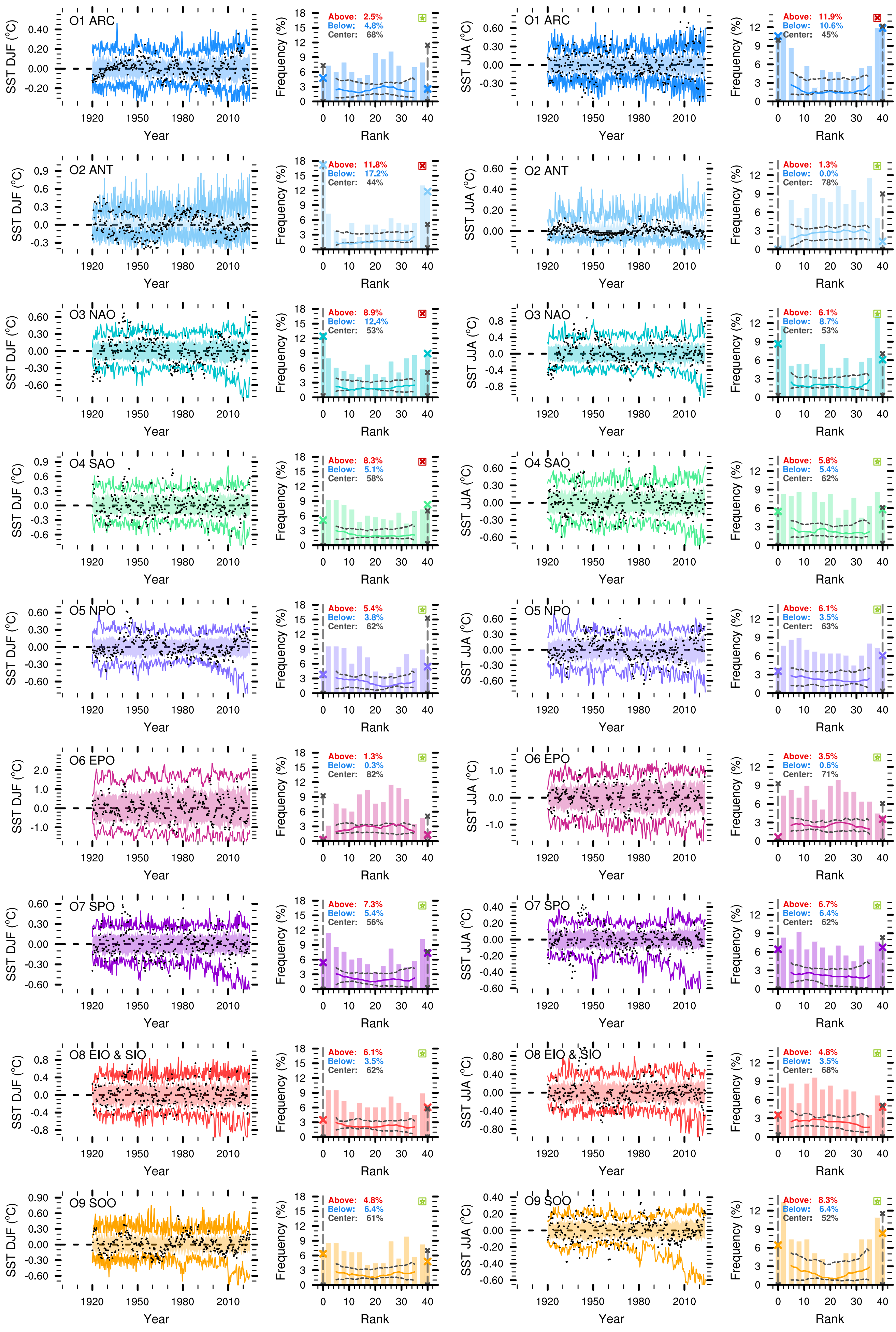

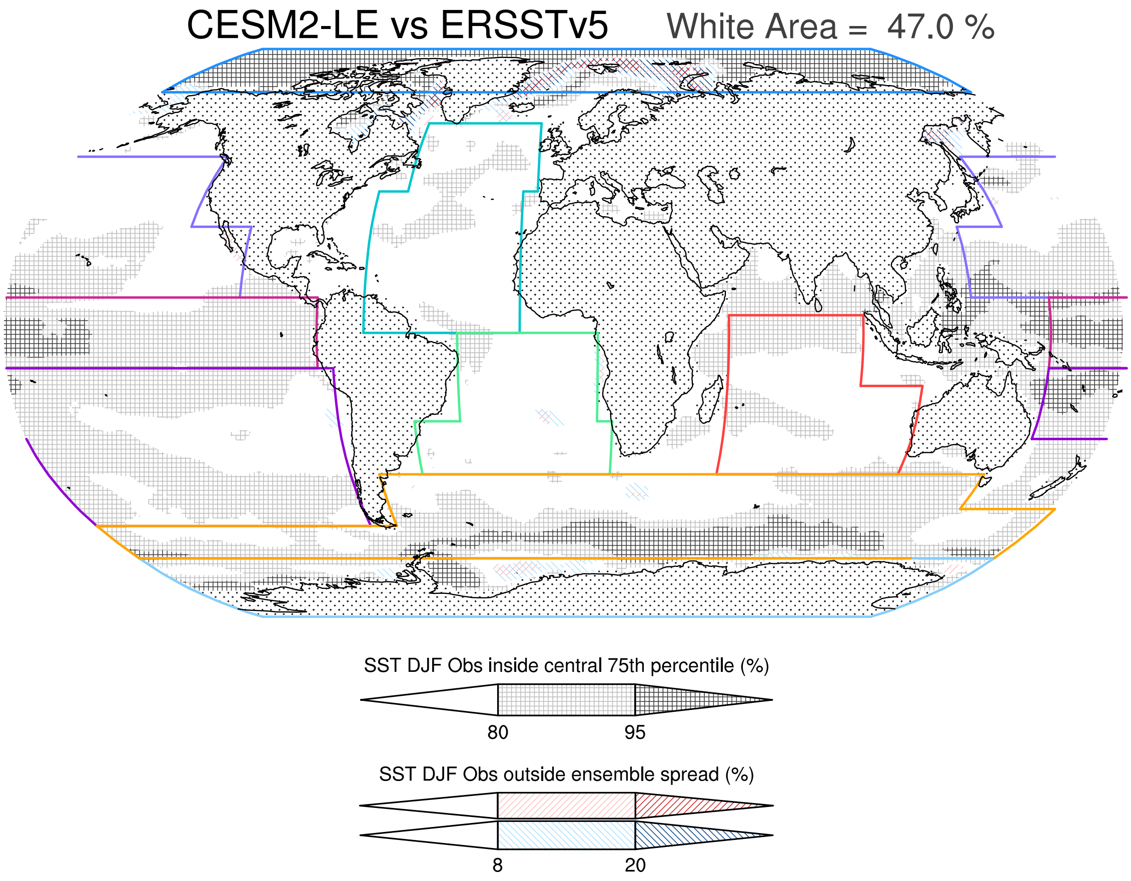

CESM2-LE vs ERSSTv5
White Area = 47.0 %
SST DJF Obs inside central 75th percentile (%)
80
95
SST DJF Obs outside ensemble spread (%)
8
20

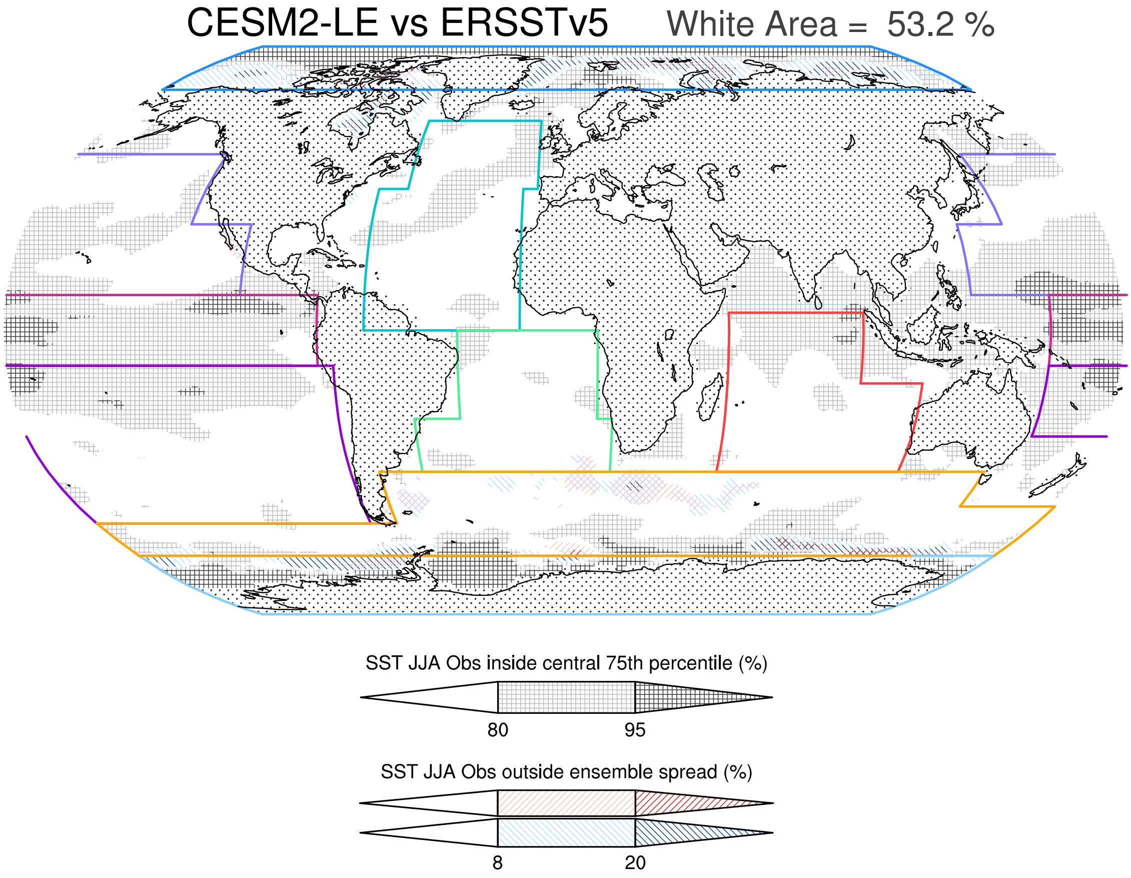

CESM2-LE vs ERSSTv5
White Area = 53.2 %
SST JJA Obs inside central 75th percentile (%)
80
95
SST JJA Obs outside ensemble spread (%)
8
20

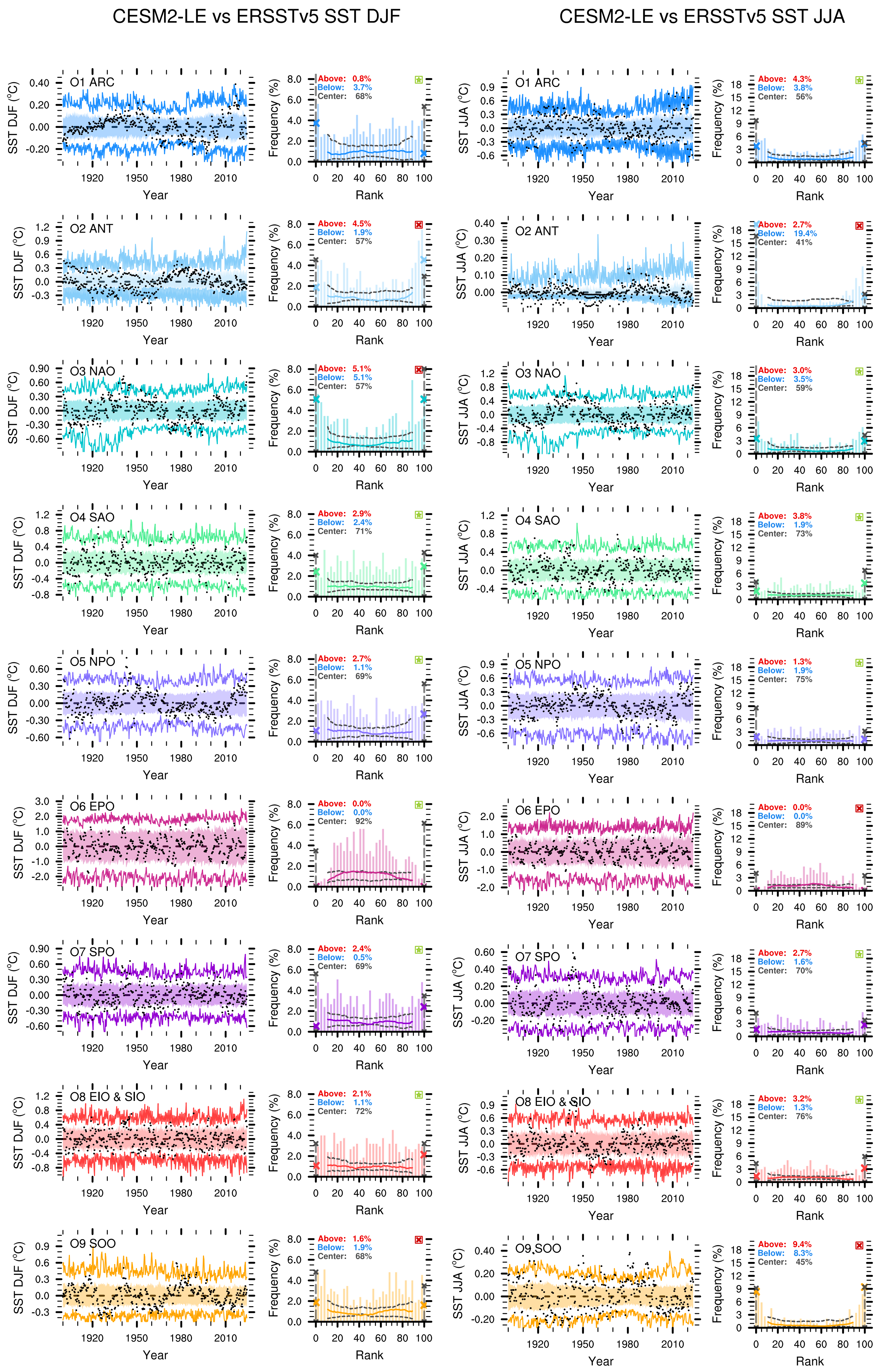
CESM2-LE vs ERSSTv5 SST DJF
CESM2-LE vs ERSSTv5 SST JJA
O1 ARC
Above: 0.8%
Below: 3.7%
Center: 68%
O1 ARC
Above: 4.3%
Below: 3.8%
Center: 56%
O2 ANT
Above: 4.5%
Below: 1.9%
Center: 57%
O2 ANT
Above: 2.7%
Below: 19.4%
Center: 41%
O3 NAO
Above: 5.1%
Below: 5.1%
Center: 57%
O3 NAO
Above: 3.0%
Below: 3.5%
Center: 59%
O4 SAO
Above: 2.9%
Below: 2.4%
Center: 71%
O4 SAO
Above: 3.8%
Below: 1.9%
Center: 73%
O5 NPO
Above: 2.7%
Below: 1.1%
Center: 69%
O5 NPO
Above: 1.3%
Below: 1.9%
Center: 75%
O6 EPO
Above: 0.0%
Below: 0.0%
Center: 92%
O6 EPO
Above: 0.0%
Below: 0.0%
Center: 89%
O7 SPO
Above: 2.4%
Below: 0.5%
Center: 69%
O7 SPO
Above: 2.7%
Below: 1.6%
Center: 70%
O8 EIO & SIO
Above: 2.1%
Below: 1.1%
Center: 72%
O8 EIO & SIO
Above: 3.2%
Below: 1.3%
Center: 76%
O9 SOO
Above: 1.6%
Below: 1.9%
Center: 68%
O9 SOO
Above: 9.4%
Below: 8.3%
Center: 45%
SST DJF (°C)
SST JJA (°C)
Year
Frequency (%)
Rank

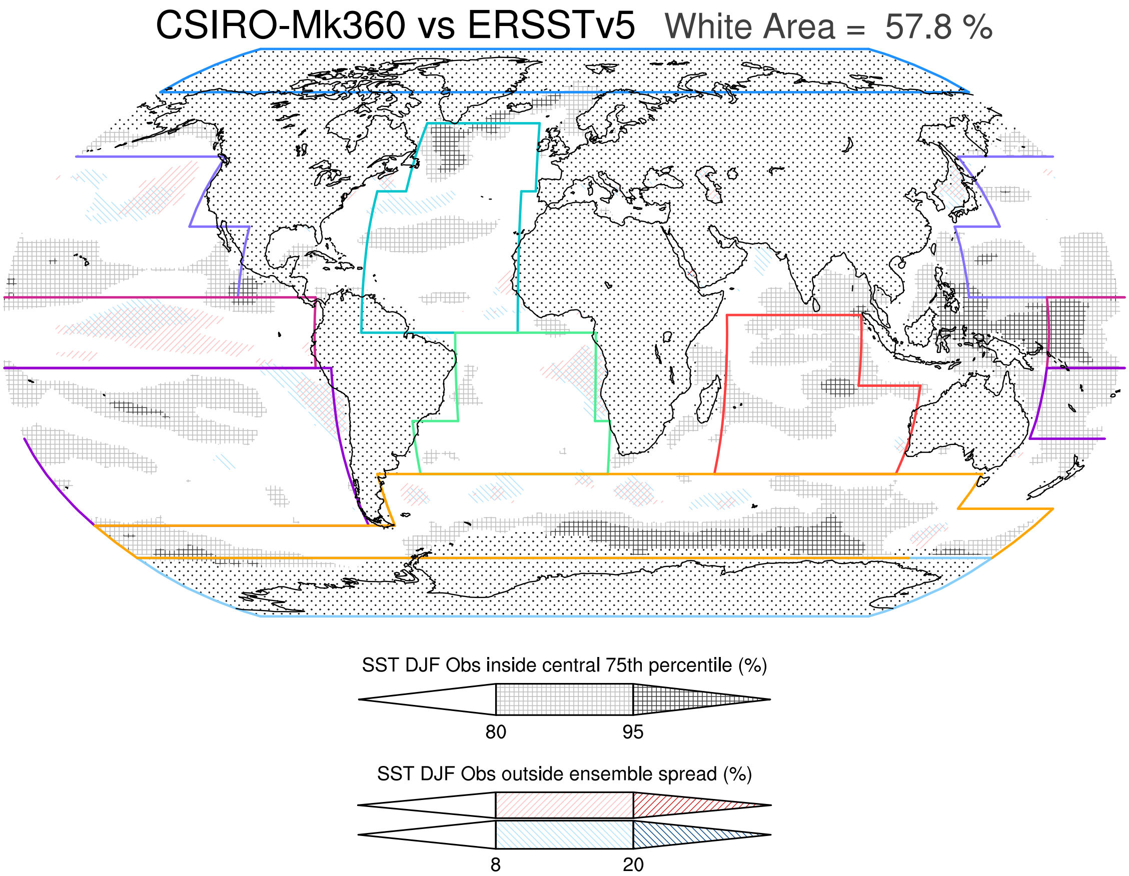
CSIRO-Mk360 vs ERSSTv5 White Area = 57.8 %
SST DJF Obs inside central 75th percentile (%)
80
95
SST DJF Obs outside ensemble spread (%)
8
20

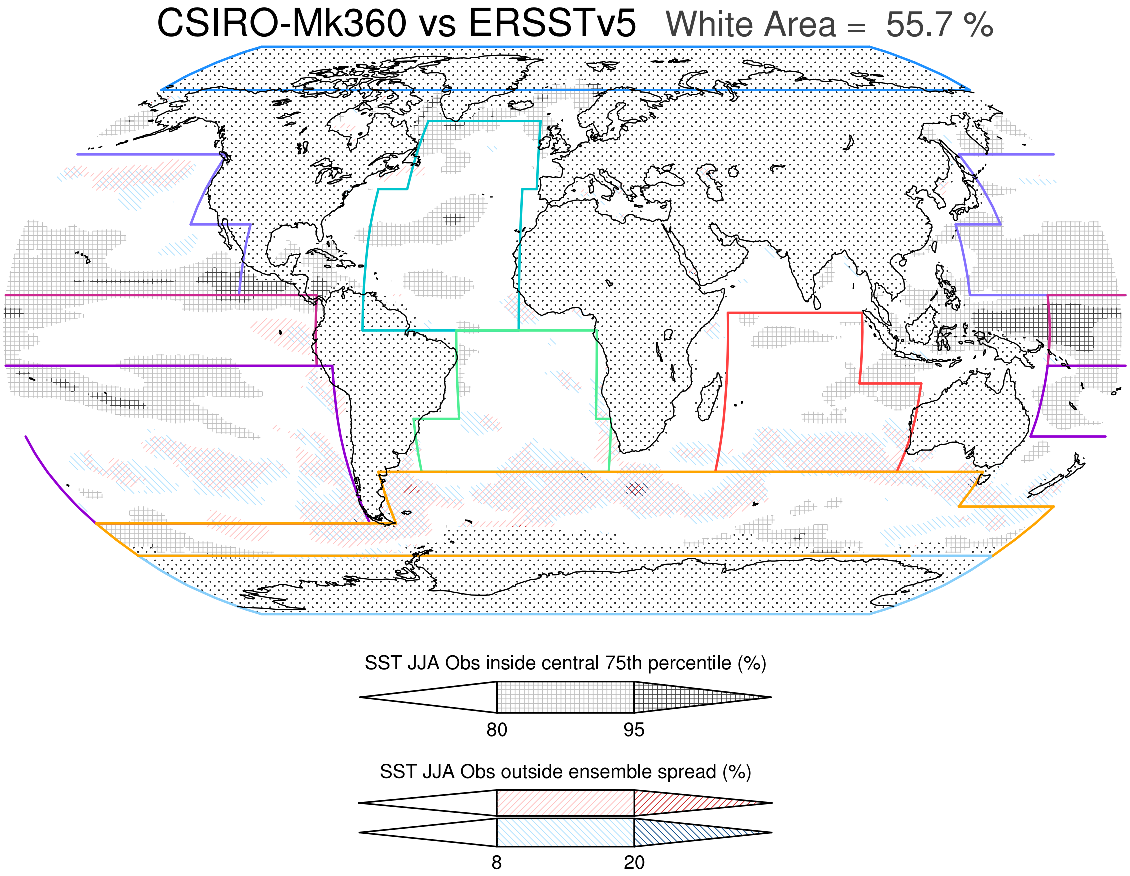
CSIRO-Mk360 vs ERSSTv5 White Area = 55.7 %
SST JJA Obs inside central 75th percentile (%)
80
95
SST JJA Obs outside ensemble spread (%)
8
20

CSIRO-Mk360 vs ERSSTv5 SST DJF

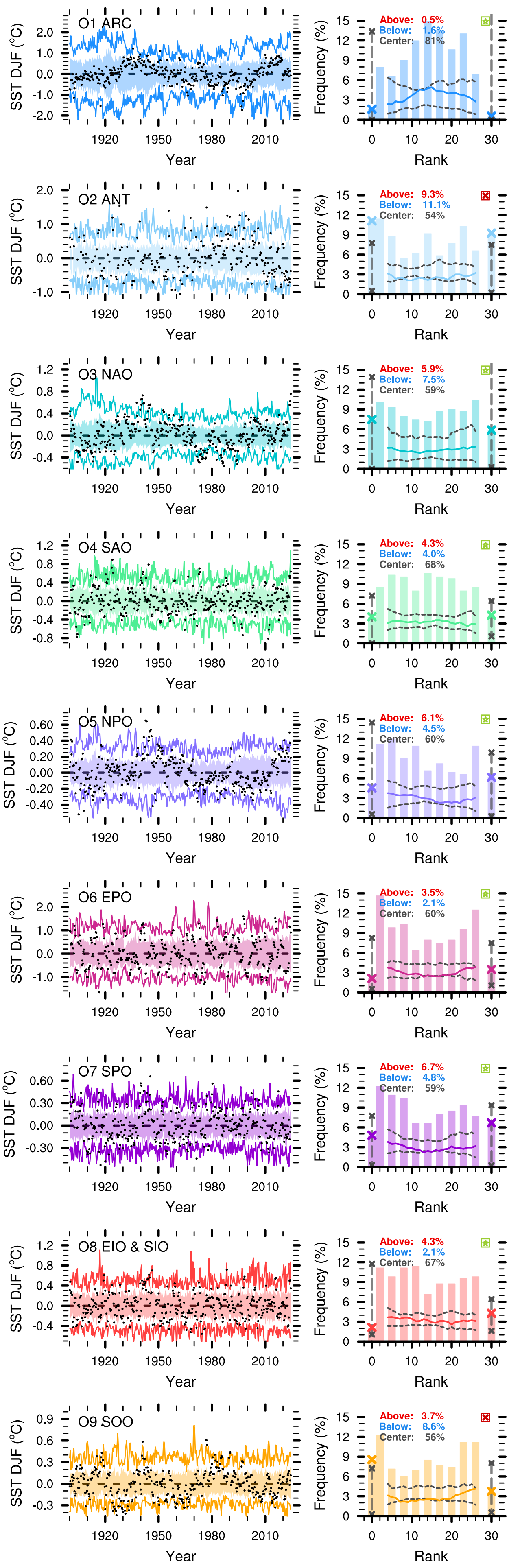

CSIRO-Mk360 vs ERSSTv5 SST JJA

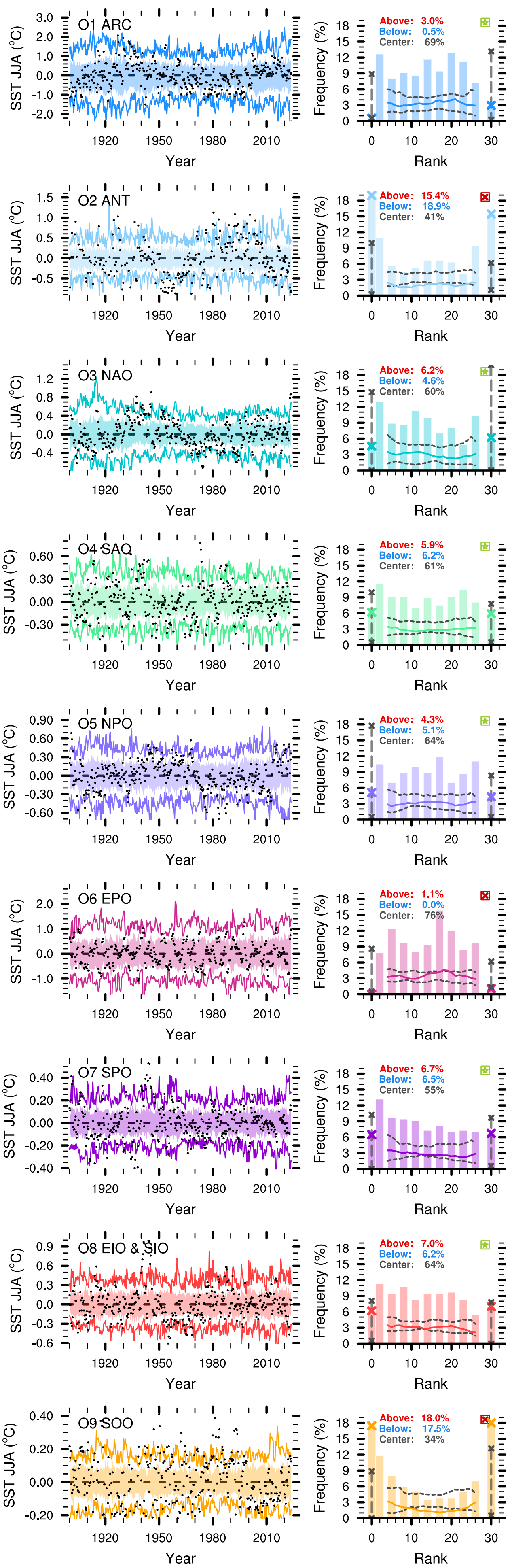

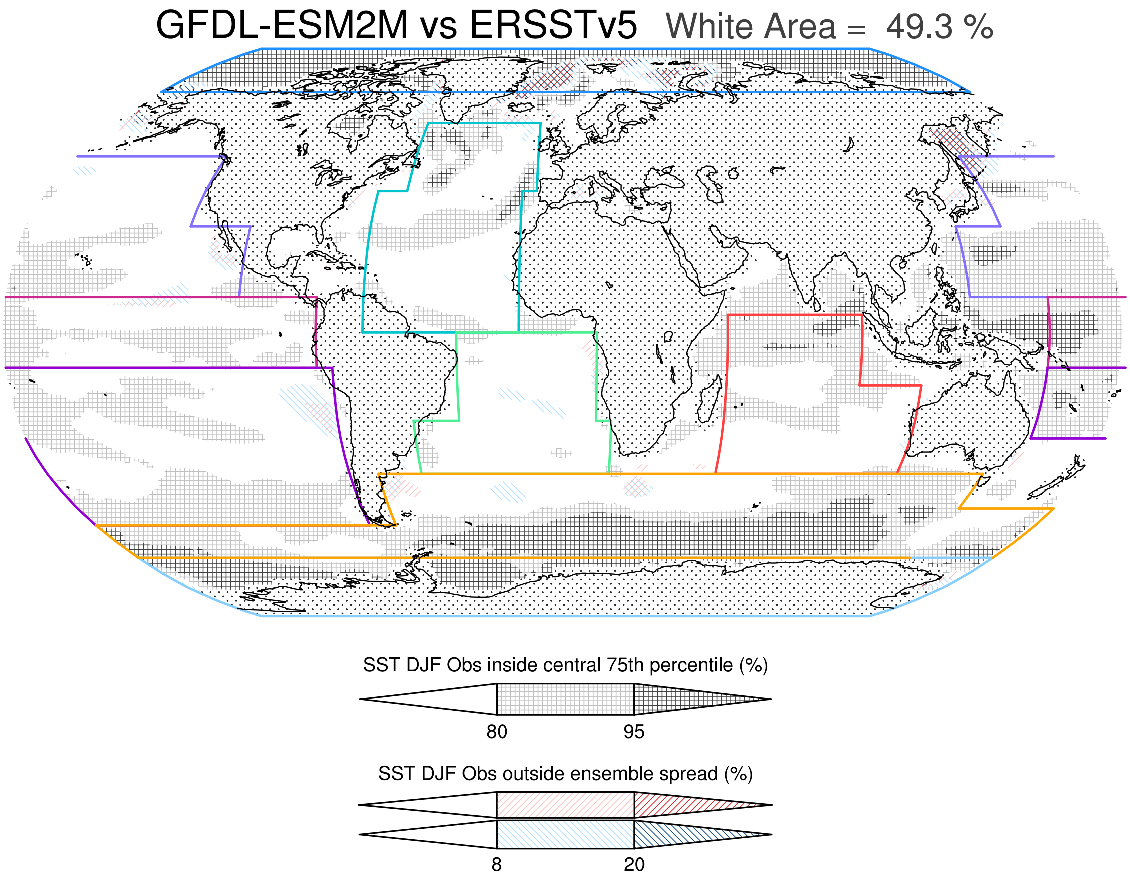
GFDL-ESM2M vs ERSSTv5 White Area = 49.3 %
SST DJF Obs inside central 75th percentile (%)
80
95
SST DJF Obs outside ensemble spread (%)
8
20

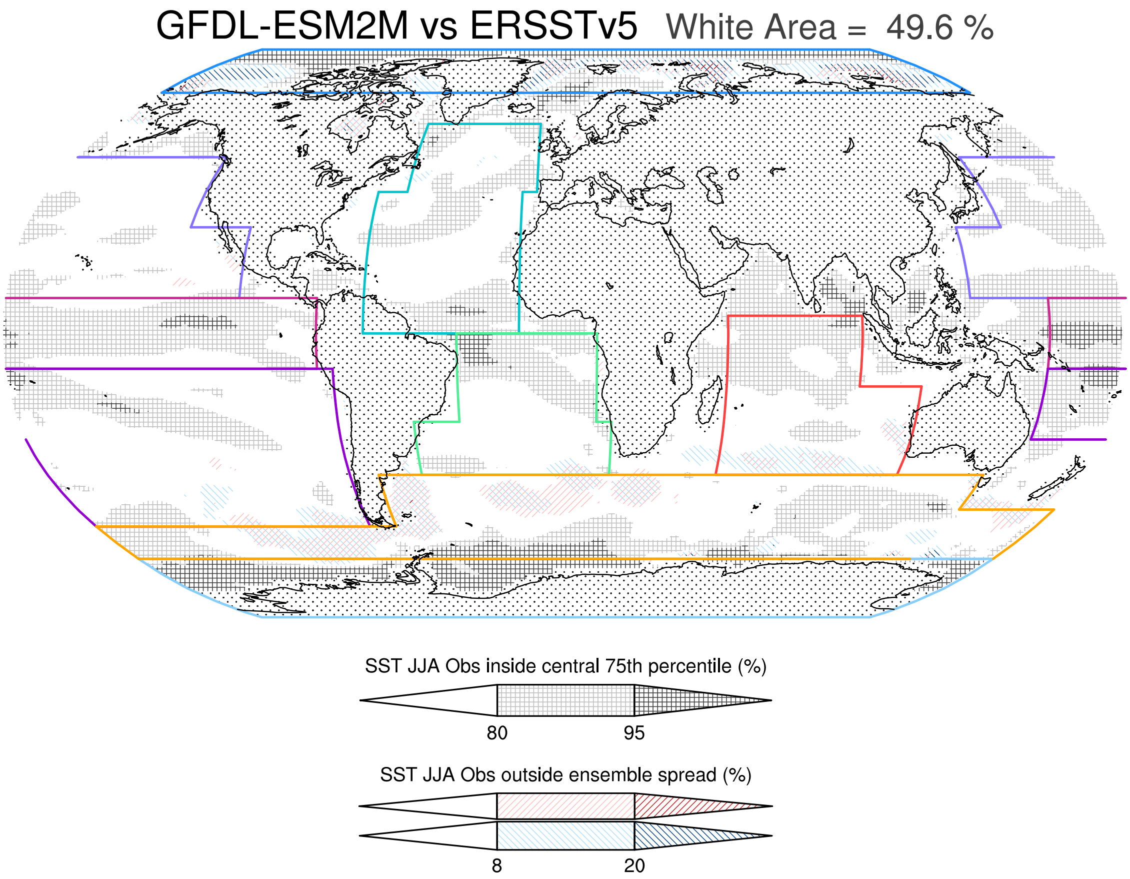
GFDL-ESM2M vs ERSSTv5 White Area = 49.6 %
SST JJA Obs inside central 75th percentile (%)
80
95
SST JJA Obs outside ensemble spread (%)
8
20

GFDL-ESM2M vs ERSSTv5 SST DJF

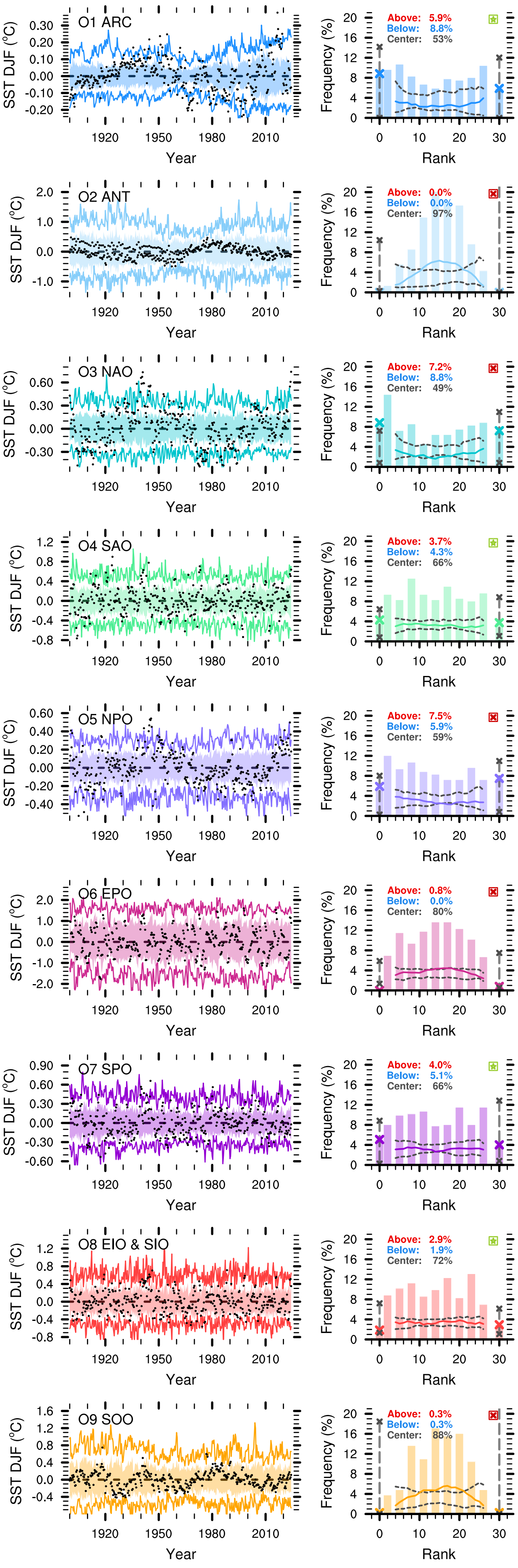

GFDL-ESM2M vs ERSSTv5 SST JJA

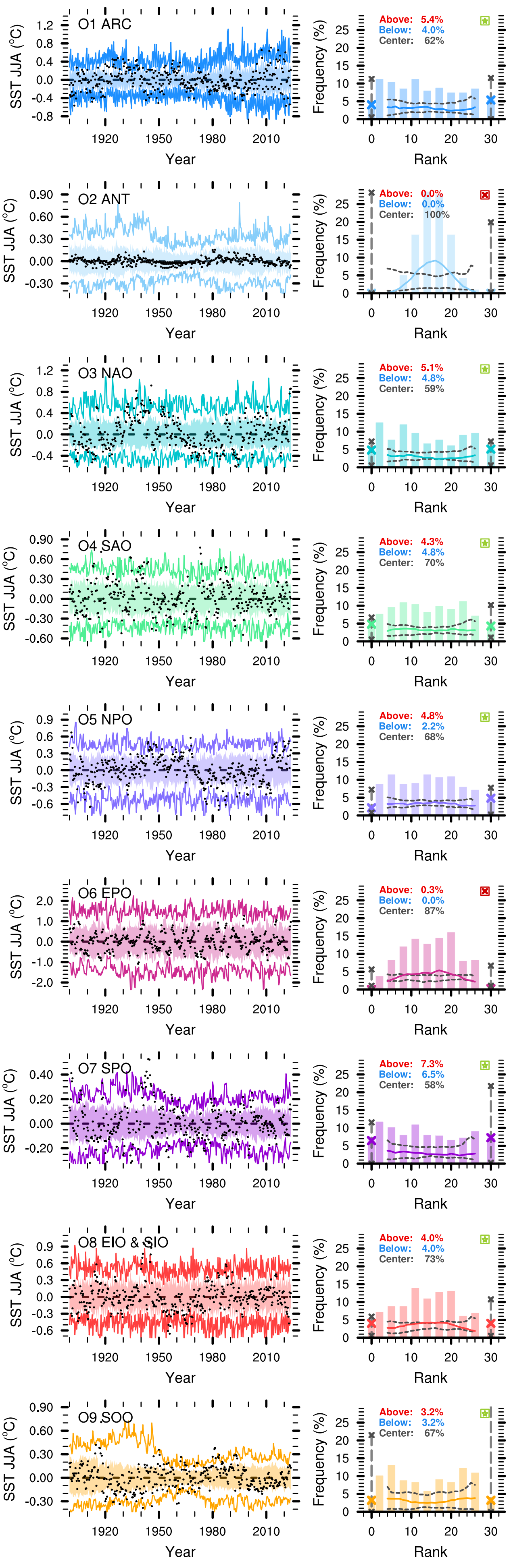

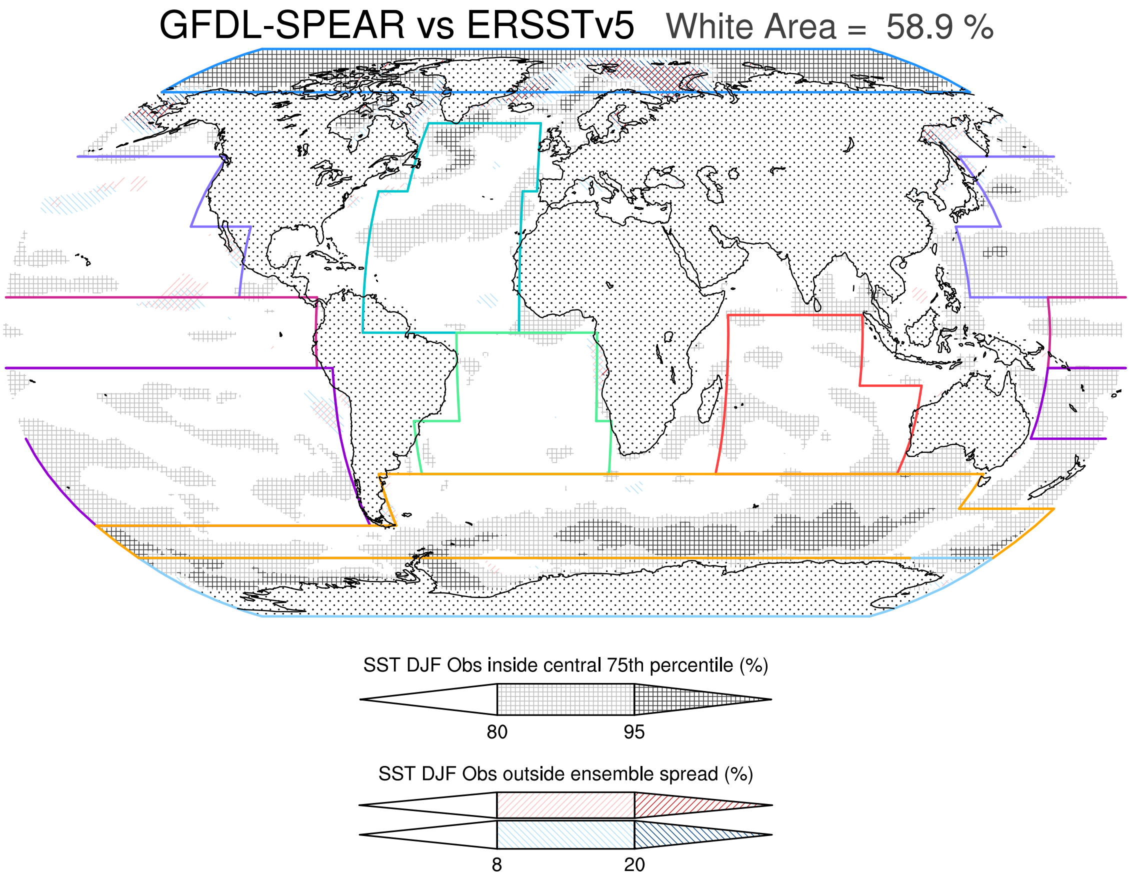
GFDL-SPEAR vs ERSSTv5 White Area = 58.9 %
SST DJF Obs inside central 75th percentile (%)
80
95
SST DJF Obs outside ensemble spread (%)
8
20

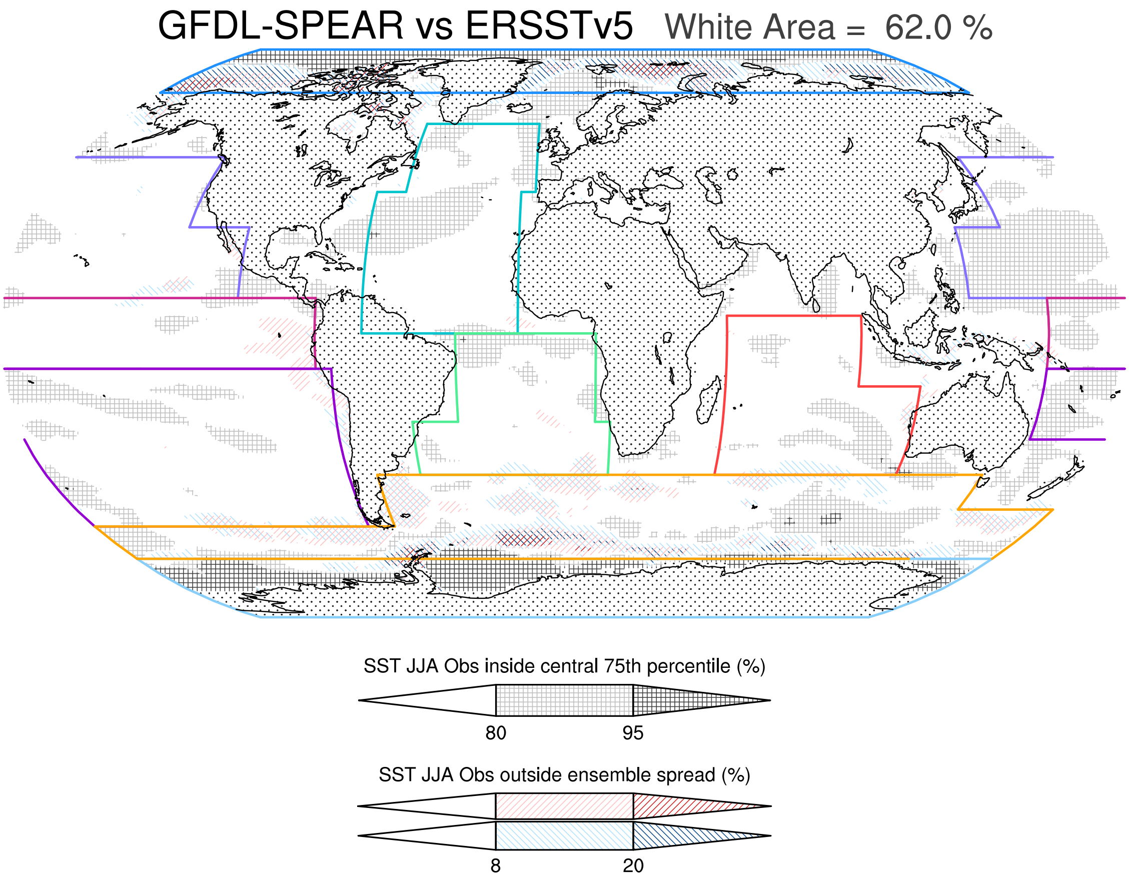
GFDL-SPEAR vs ERSSTv5 White Area = 62.0 %
SST JJA Obs inside central 75th percentile (%)
80
95
SST JJA Obs outside ensemble spread (%)
8
20

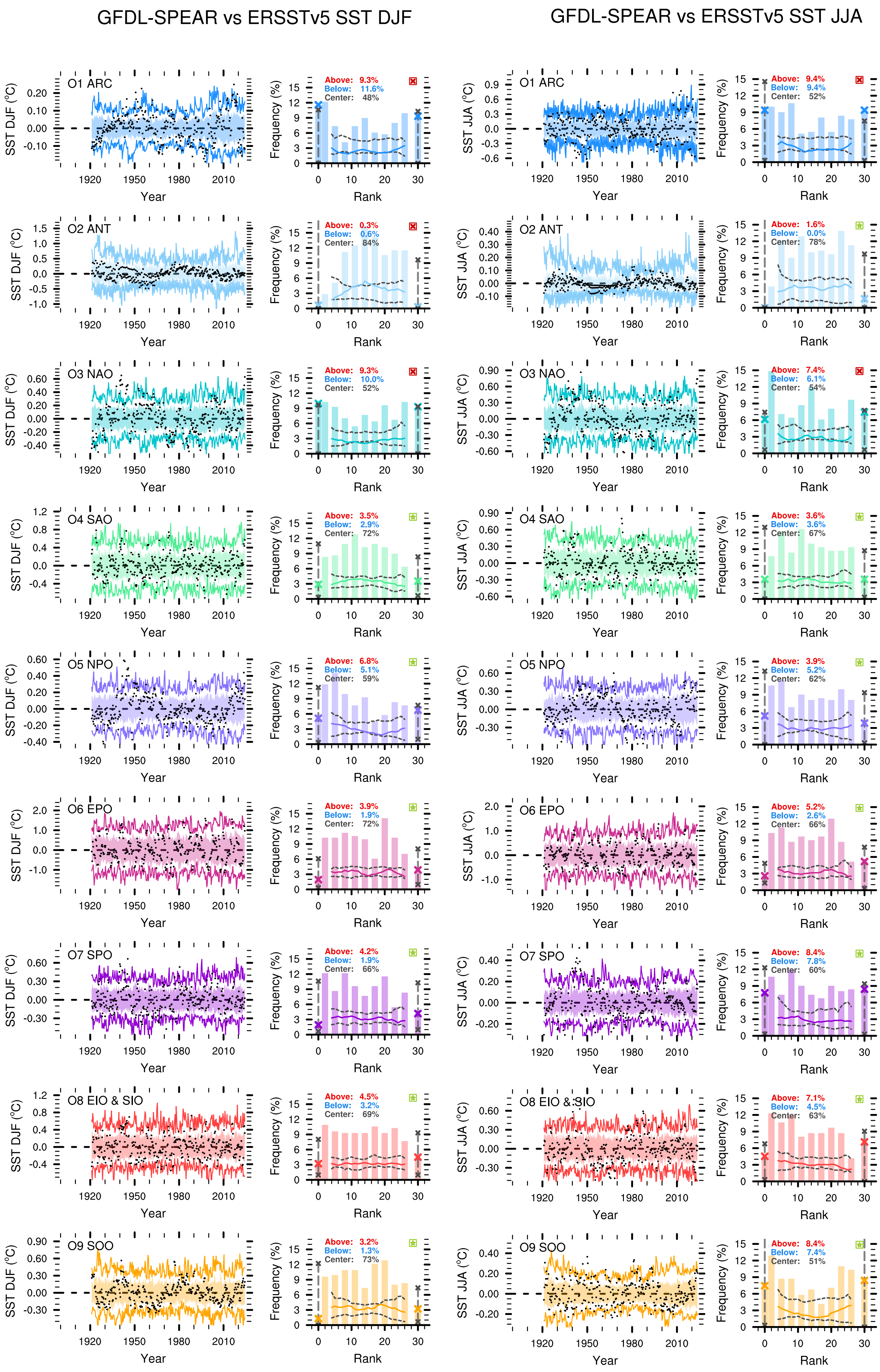

GFDL-SPEAR vs ERSSTv5 SST DJF
GFDL-SPEAR vs ERSSTv5 SST JJA
O1 ARC
Above: 9.3%
Below: 11.6%
Center: 48%
O2 ANT
Above: 0.3%
Below: 0.6%
Center: 84%
O3 NAO
Above: 9.3%
Below: 10.0%
Center: 52%
O4 SAO
Above: 3.5%
Below: 2.9%
Center: 72%
O5 NPO
Above: 6.8%
Below: 5.1%
Center: 59%
O6 EPO
Above: 3.9%
Below: 1.9%
Center: 72%
O7 SPO
Above: 4.2%
Below: 1.9%
Center: 66%
O8 EIO & SIO
Above: 4.5%
Below: 3.2%
Center: 69%
O9 SOO
Above: 3.2%
Below: 1.3%
Center: 73%
O1 ARC
Above: 9.4%
Below: 9.4%
Center: 52%
O2 ANT
Above: 1.6%
Below: 0.0%
Center: 78%
O3 NAO
Above: 7.4%
Below: 6.1%
Center: 54%
O4 SAO
Above: 3.6%
Below: 3.6%
Center: 67%
O5 NPO
Above: 3.9%
Below: 5.2%
Center: 62%
O6 EPO
Above: 5.2%
Below: 2.6%
Center: 66%
O7 SPO
Above: 8.4%
Below: 7.8%
Center: 60%
O8 EIO & SIO
Above: 7.1%
Below: 4.5%
Center: 63%
O9 SOO
Above: 8.4%
Below: 7.4%
Center: 51%
SST DJF (°C)
SST JJA (°C)
Frequency (%)
Year
Rank

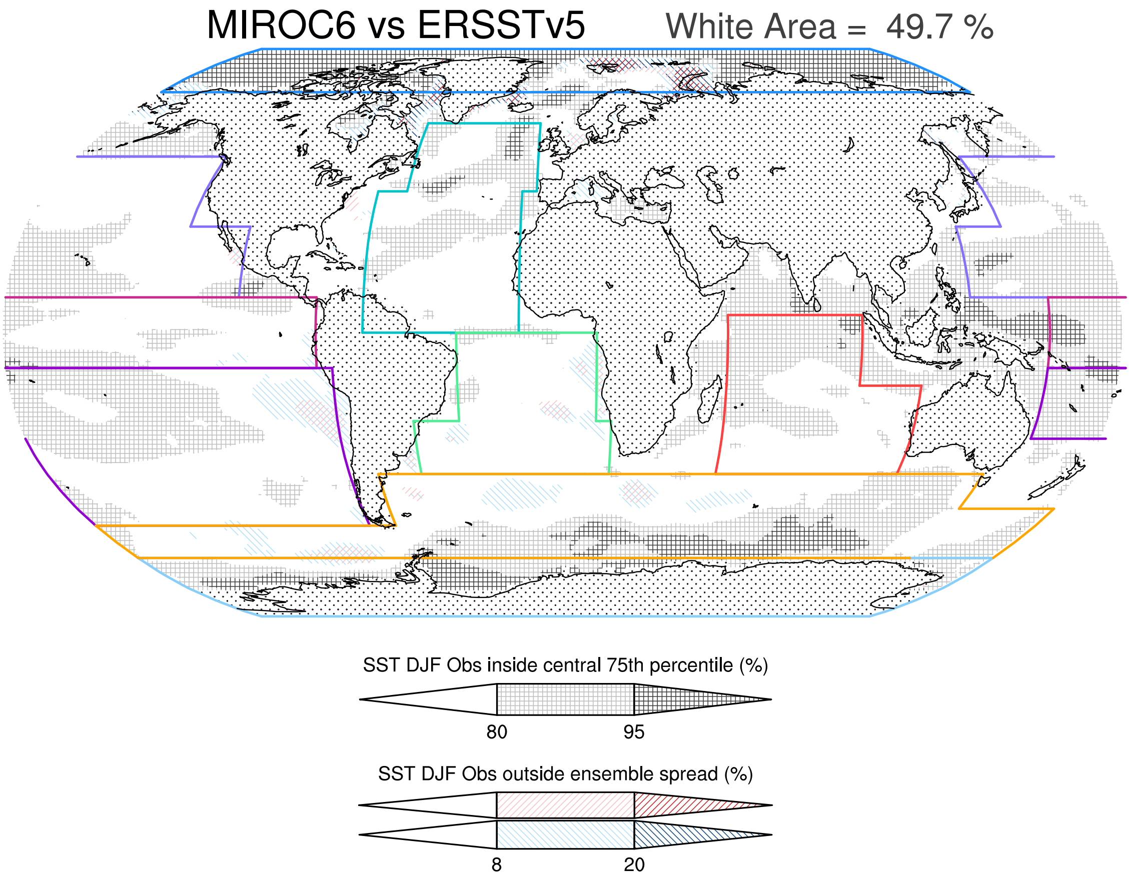
MIROC6 vs ERSSTv5
White Area = 49.7 %
SST DJF Obs inside central 75th percentile (%)
80
95
SST DJF Obs outside ensemble spread (%)
8
20

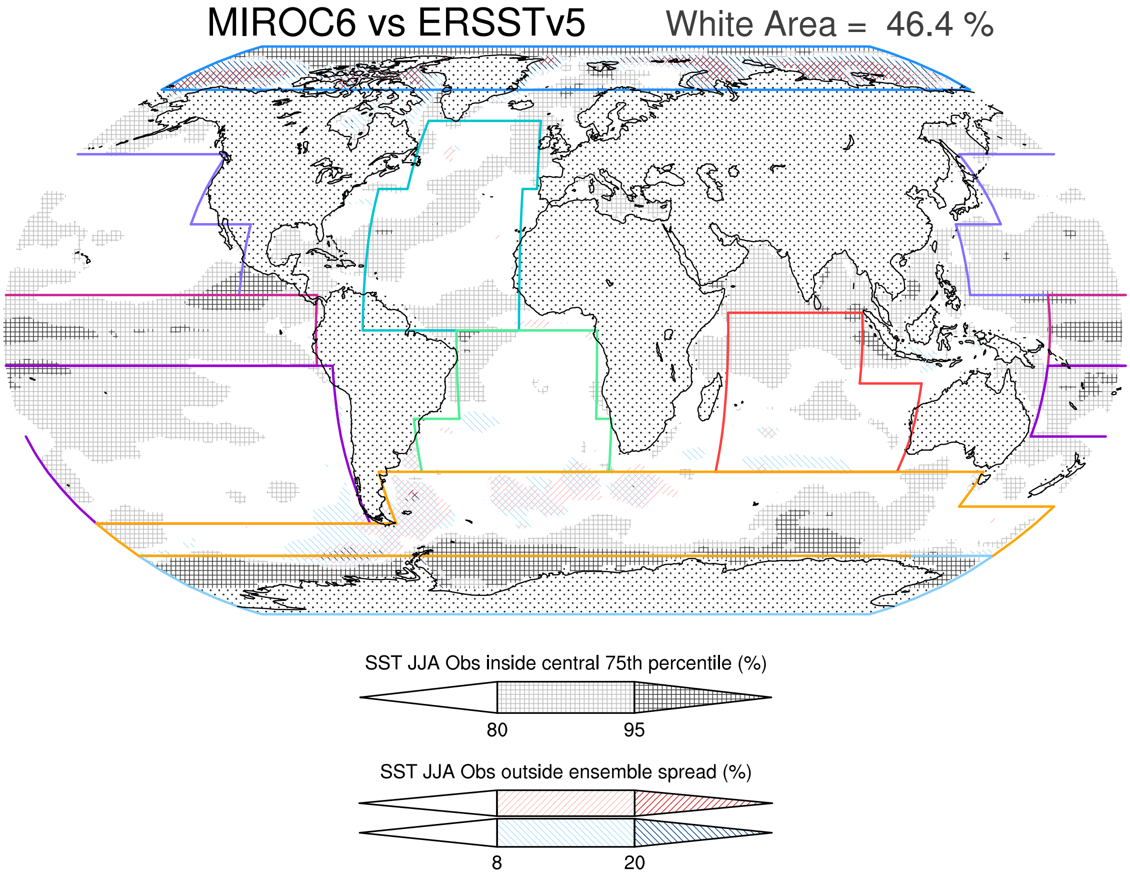
MIROC6 vs ERSSTv5
White Area = 46.4 %
SST JJA Obs inside central 75th percentile (%)
80
95
SST JJA Obs outside ensemble spread (%)
8
20

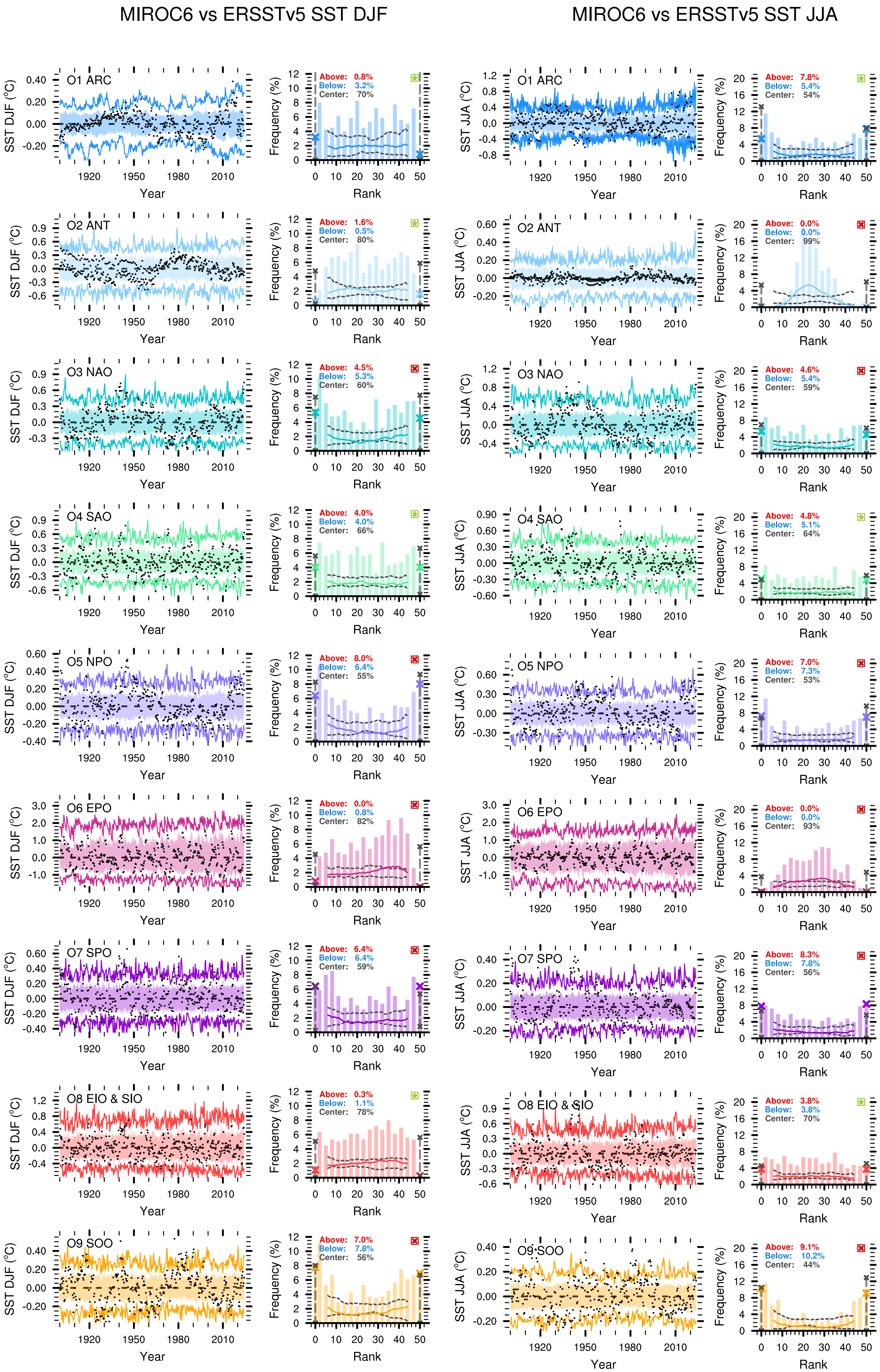
MIROC6 vs ERSSTv5 SST DJF
MIROC6 vs ERSSTv5 SST JJA
SST DJF (°C)
SST JJA (°C)
Year
Frequency (%)
Rank
O1 ARC
Above: 0.8%
Below: 3.2%
Center: 70%
O2 ANT
Above: 1.6%
Below: 0.5%
Center: 80%
O3 NAO
Above: 4.5%
Below: 5.3%
Center: 60%
O4 SAO
Above: 4.0%
Below: 4.0%
Center: 66%
O5 NPO
Above: 8.0%
Below: 6.4%
Center: 55%
O6 EPO
Above: 0.0%
Below: 0.8%
Center: 82%
O7 SPO
Above: 6.4%
Below: 6.4%
Center: 59%
O8 EIO & SIO
Above: 0.3%
Below: 1.1%
Center: 78%
O9 SOO
Above: 7.0%
Below: 7.8%
Center: 56%
O1 ARC
Above: 7.8%
Below: 5.4%
Center: 54%
O2 ANT
Above: 0.0%
Below: 0.0%
Center: 99%
O3 NAO
Above: 4.6%
Below: 5.4%
Center: 59%
O4 SAO
Above: 4.8%
Below: 5.1%
Center: 64%
O5 NPO
Above: 7.0%
Below: 7.3%
Center: 53%
O6 EPO
Above: 0.0%
Below: 0.0%
Center: 93%
O7 SPO
Above: 8.3%
Below: 7.8%
Center: 56%
O8 EIO & SIO
Above: 3.8%
Below: 3.8%
Center: 70%
O9 SOO
Above: 9.1%
Below: 10.2%
Center: 44%

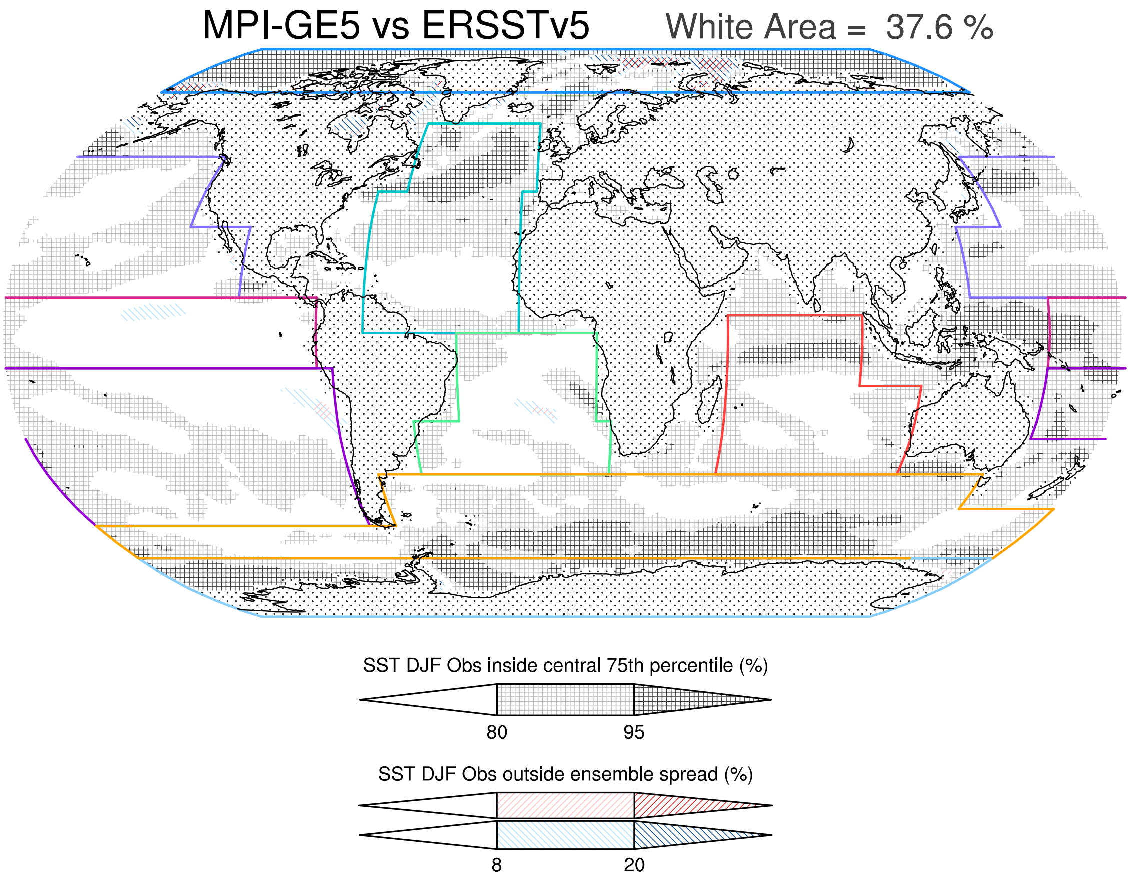
MPI-GE5 vs ERSSTv5
White Area = 37.6 %
SST DJF Obs inside central 75th percentile (%)
80
95
SST DJF Obs outside ensemble spread (%)
8
20

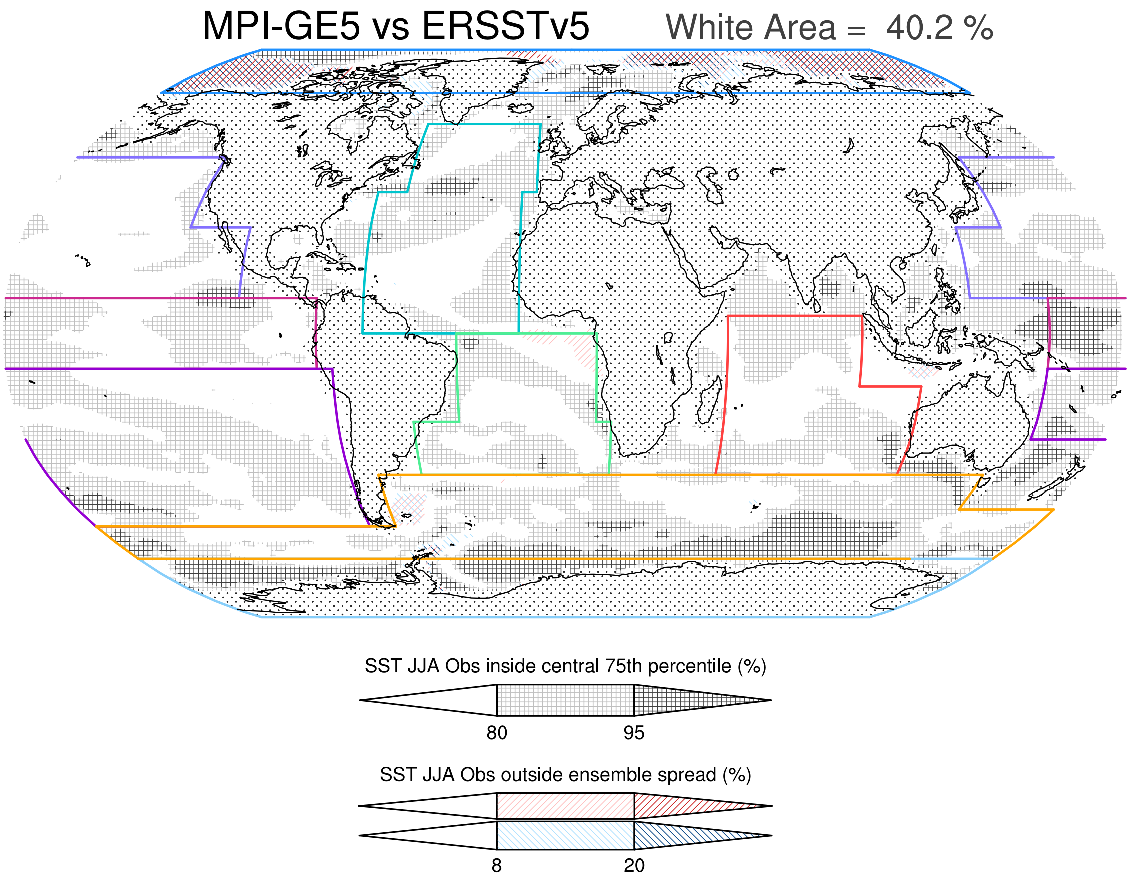
MPI-GE5 vs ERSSTv5
White Area = 40.2 %
SST JJA Obs inside central 75th percentile (%)
80
95
SST JJA Obs outside ensemble spread (%)
8
20

MPI-GE5 vs ERSSTv5 SST DJF

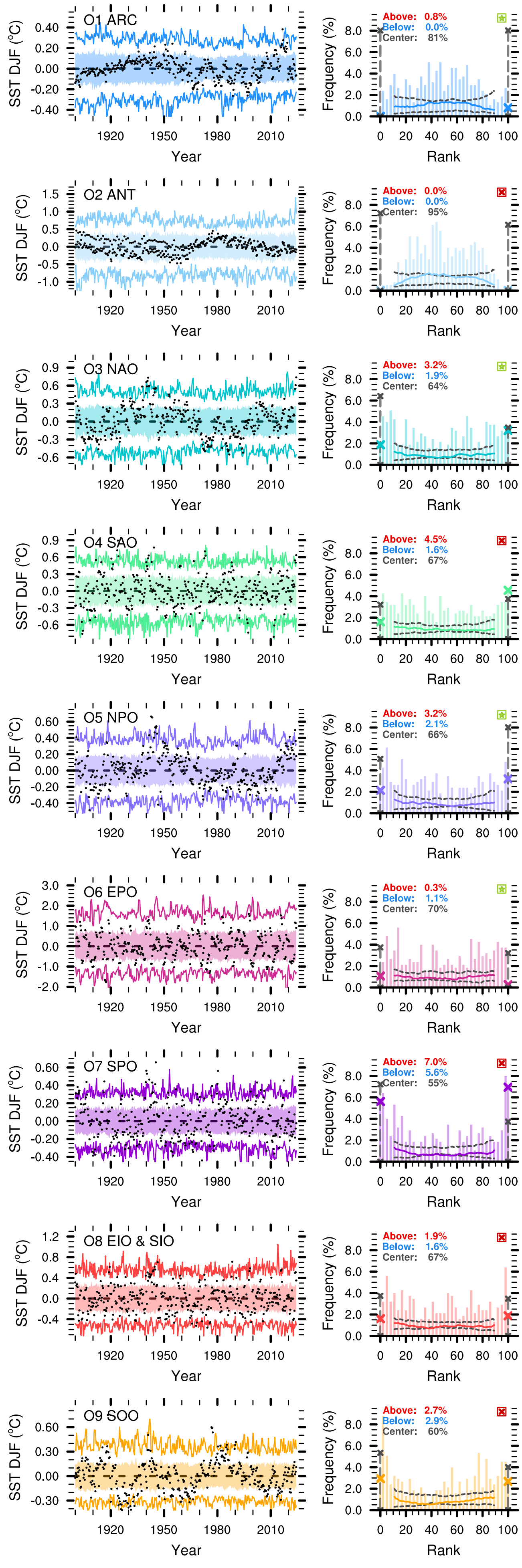

MPI-GE5 vs ERSSTv5 SST JJA

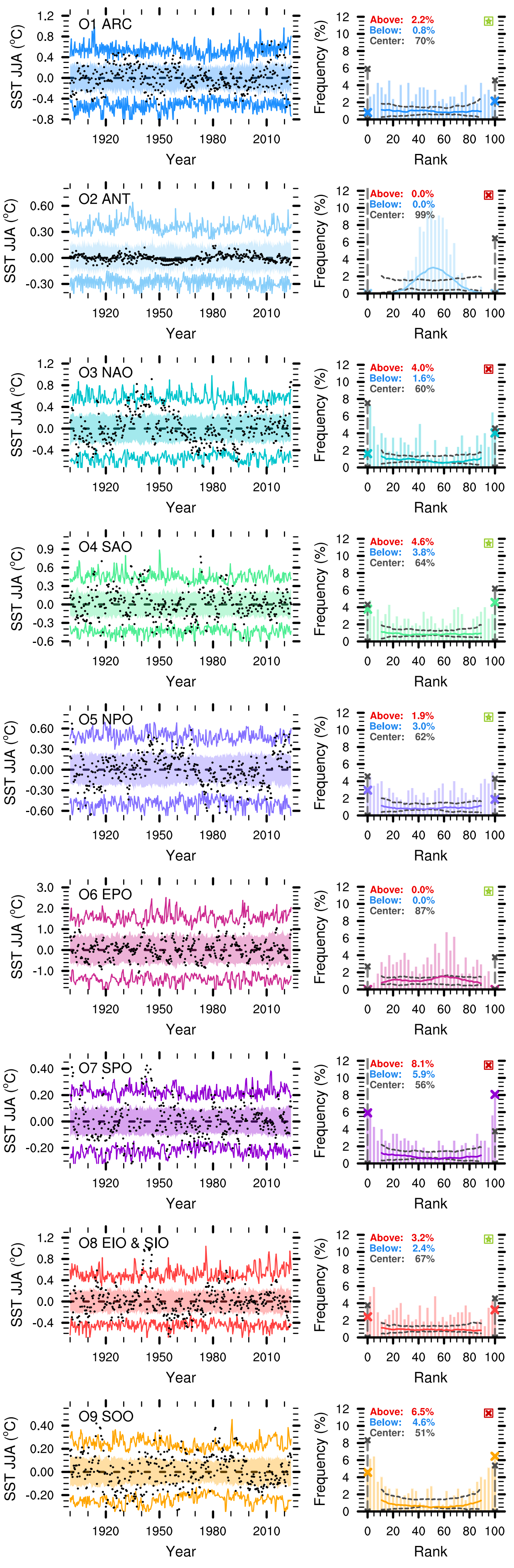

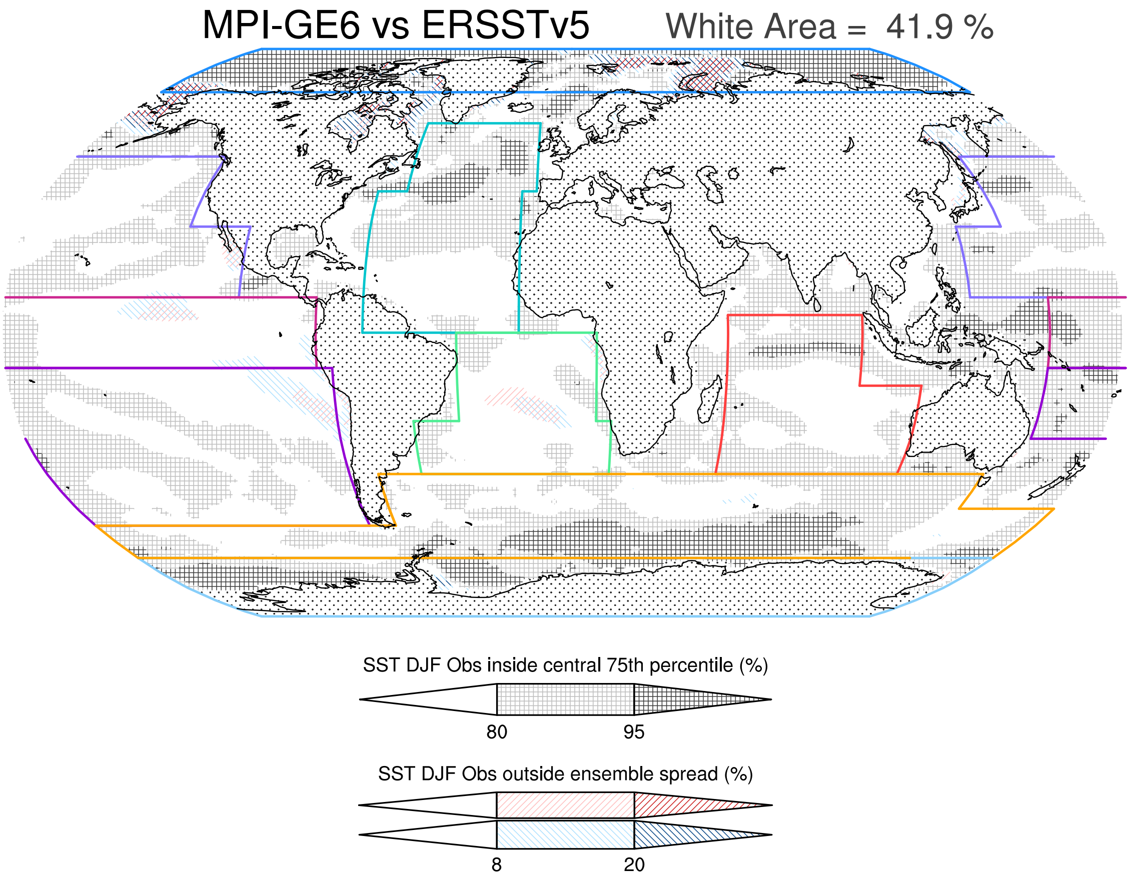

MPI-GE6 vs ERSSTv5
White Area = 41.9 %
SST DJF Obs inside central 75th percentile (%)
80
95
SST DJF Obs outside ensemble spread (%)
8
20

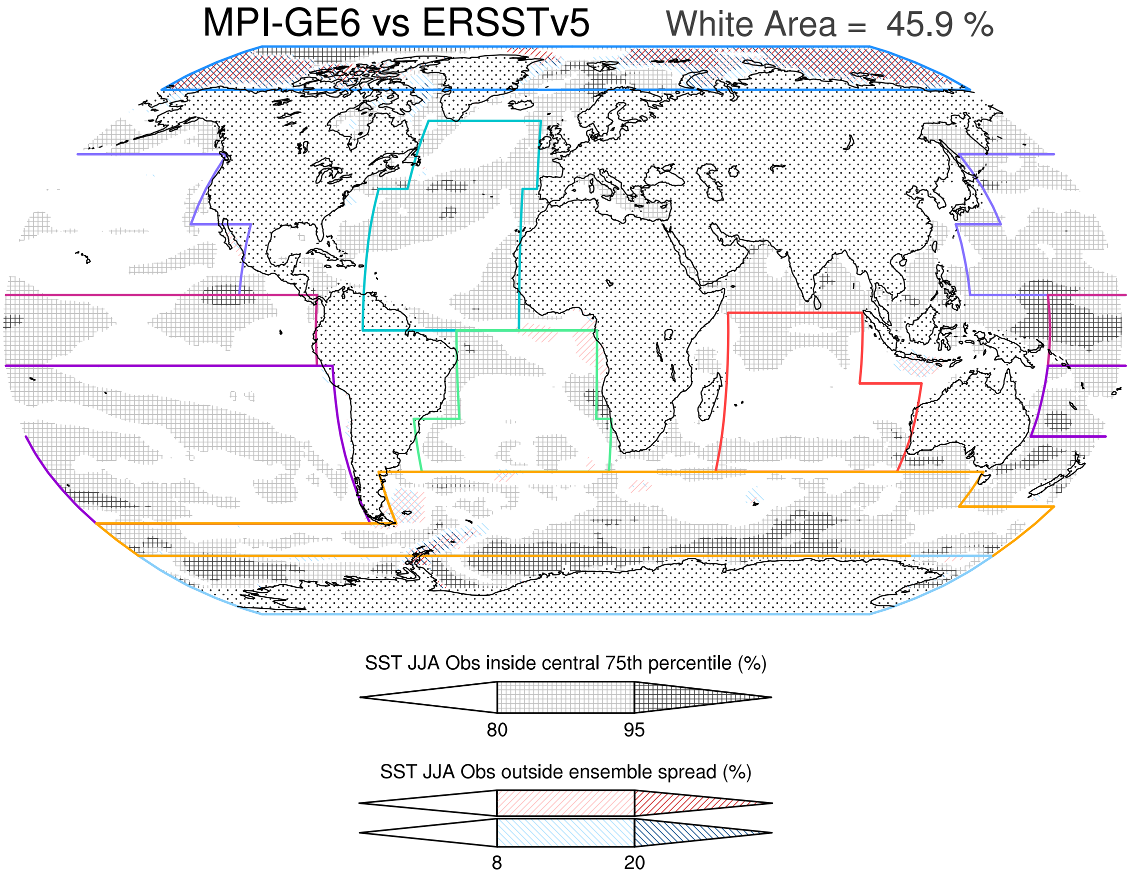

MPI-GE6 vs ERSSTv5
White Area = 45.9 %
SST JJA Obs inside central 75th percentile (%)
80
95
SST JJA Obs outside ensemble spread (%)
8
20

MPI-GE6 vs ERSSTv5 SST DJF

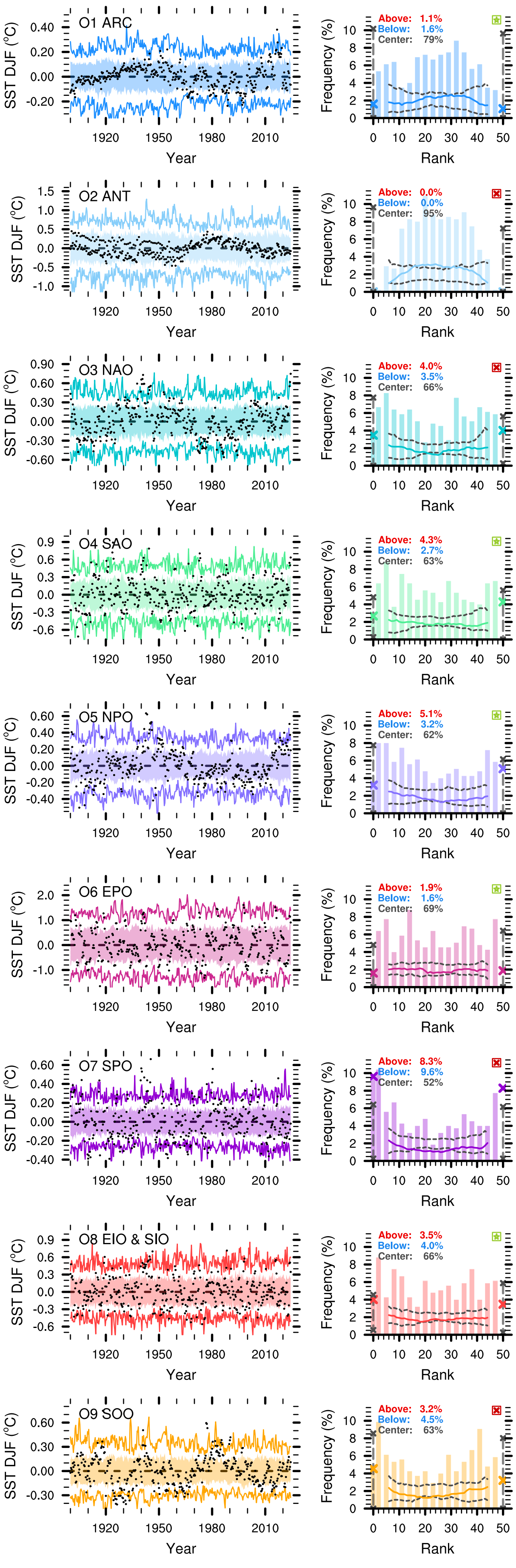

MPI-GE6 vs ERSSTv5 SST JJA

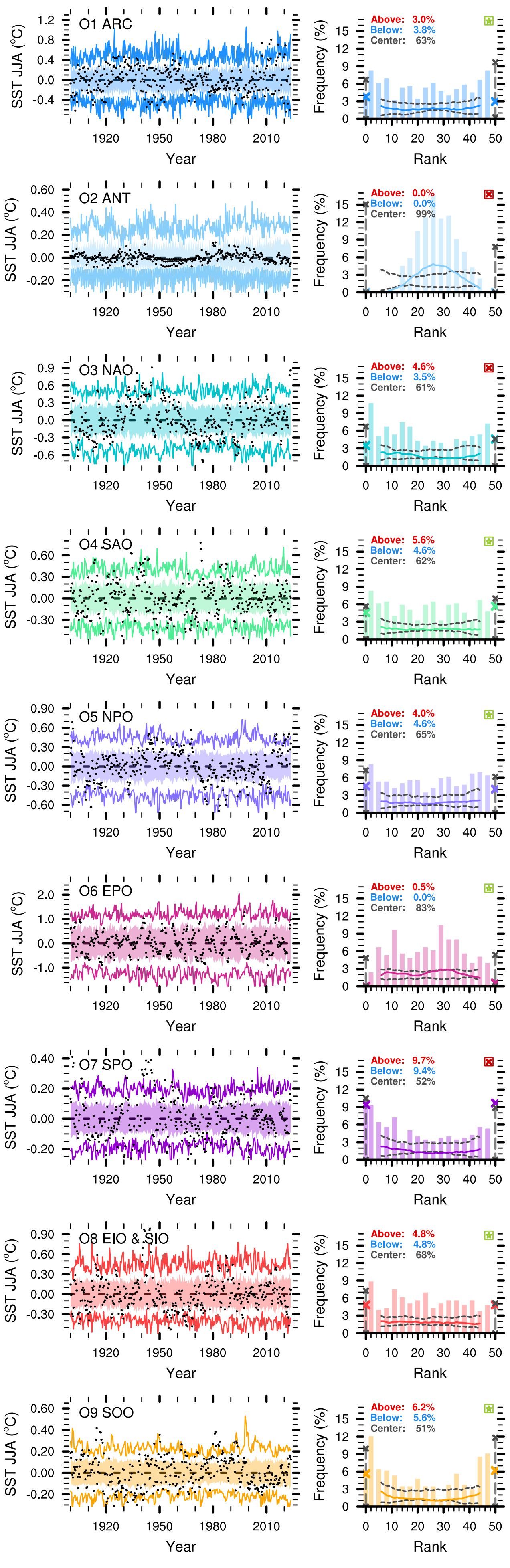

Non-Detrended Ocean Surface Temperatures

Rank-frequency variability evaluation framework for non-detrended sea surface temperature (SST) anomalies over ocean grid cells.

Maps show grid-cell evaluation of the simulated DJF and JJA monthly mean temperature anomalies for the 11 SMILEs included against ERSSTv5 observations globally. Gray hatching represents where observations cluster within the 75th percentile bounds of the ensemble (12.5th to 87.5th percentiles) for more than 80% of months (light grey) or for more than 95% of months (dark grey). Red and blue shading represents where observations are larger than the ensemble maximum (red) or smaller than the ensemble minimum (blue), respectively, for more than 8% of the months (light red and blue) or for more than 20% of the months (dark red and blue). Dotted areas represent land areas or grid cells where observations are missing and are therefore excluded from this analysis. Colored boxes demark the boundaries of each ocean region assessed. The percentage of assessed grid-cells that present none of these biases, unbiased grid-cells, in given at the top (white area). For each assessed region, models are considered to provide an adequate grid-cell representation when they exhibit more than 50% of unbiased grid cells in the region, fulfilling Criteria 2 of our evaluation framework.

Time series and rank frequency histograms show spatially aggregated DJF and JJA SST for each ocean region for all 11 SMILEs. Time series show the ensemble maximum and minimum (color lines) and central 75th percentile ensemble spread (shading) are shown against observations (black dots).

Rank histograms represent the frequency of each place that observations would take in a list of ensemble members ordered by ascending temperature anomaly values. Rank histograms show the observations rank frequency accumulated for 3-rank bins (bars), the running mean rank frequency over a centered n/5 rank window (lines; for 1 to n-1 ranks) and the absolute frequencies of rank 0 and n (crosses), with n the number of ensemble members, for observations (color) and perfect model rank range (gray).

To illustrate how internal variability sampling may affect rank frequencies given the non-infinite record length considered, we include a comparison to the perfect-model rank range, which shows the range of rank frequencies that each ensemble member would take if it were observations (see Methods in main article for further details). If the rank exhibited by observations (colors) is within this perfect-model range (gray; allowing a maximum 10% deviation outside of this range), the rank frequency evaluation shows an adequate model performance, and any deviations from a perfectly frank rank histogram can be assumed to be within the range of deviations that could be caused by internal variability. In the case the observations rank frequency is within the perfect-model range for all rank windows, Criteria 1 of our evaluation framework is met and this is highlighted by a green star at the top right; if not, by a red cross. Percentages at the top left show the frequency of regionally averaged observations occurring above (red) or below (blue) ensemble limits, or clustering within the central 75th percentile range (gray), analogous to the grid-cell evaluation in Criteria 2.

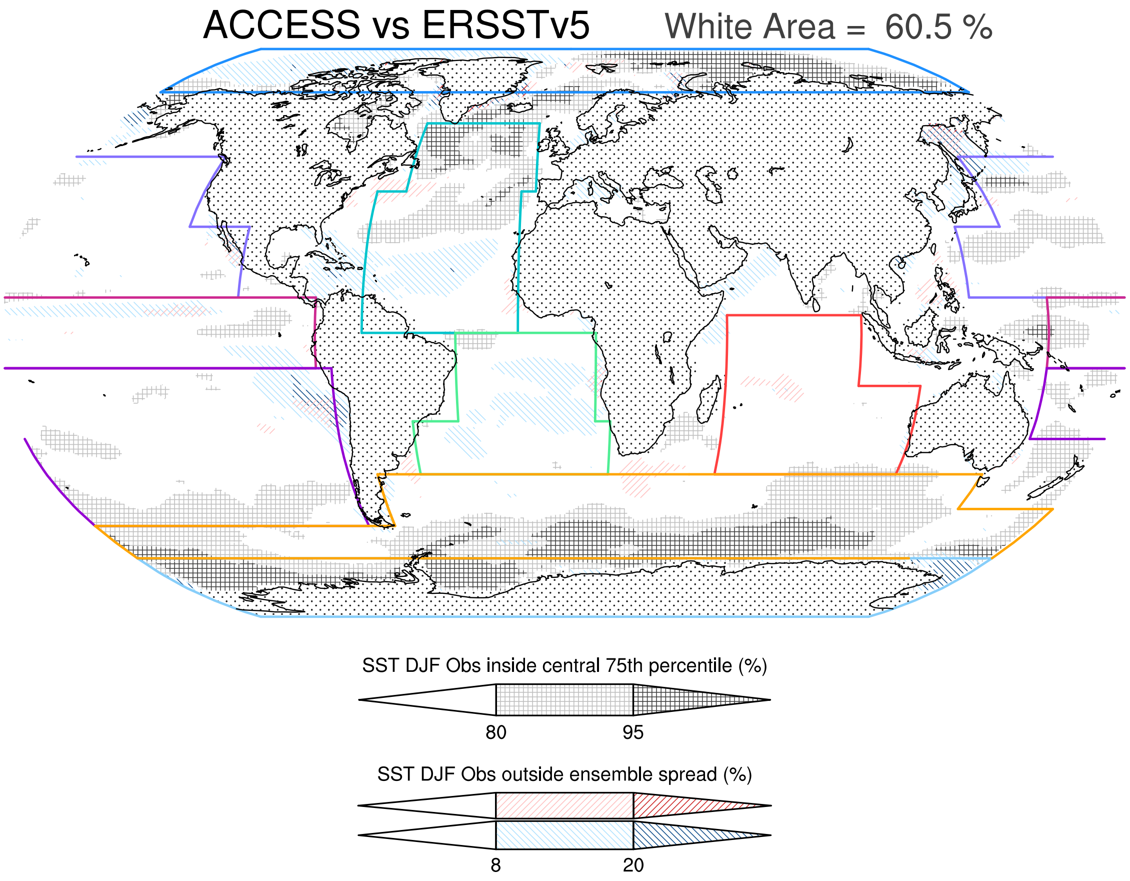
ACCESS vs ERSSTv5
White Area = 60.5 %
SST DJF Obs inside central 75th percentile (%)
80
95
SST DJF Obs outside ensemble spread (%)
8
20

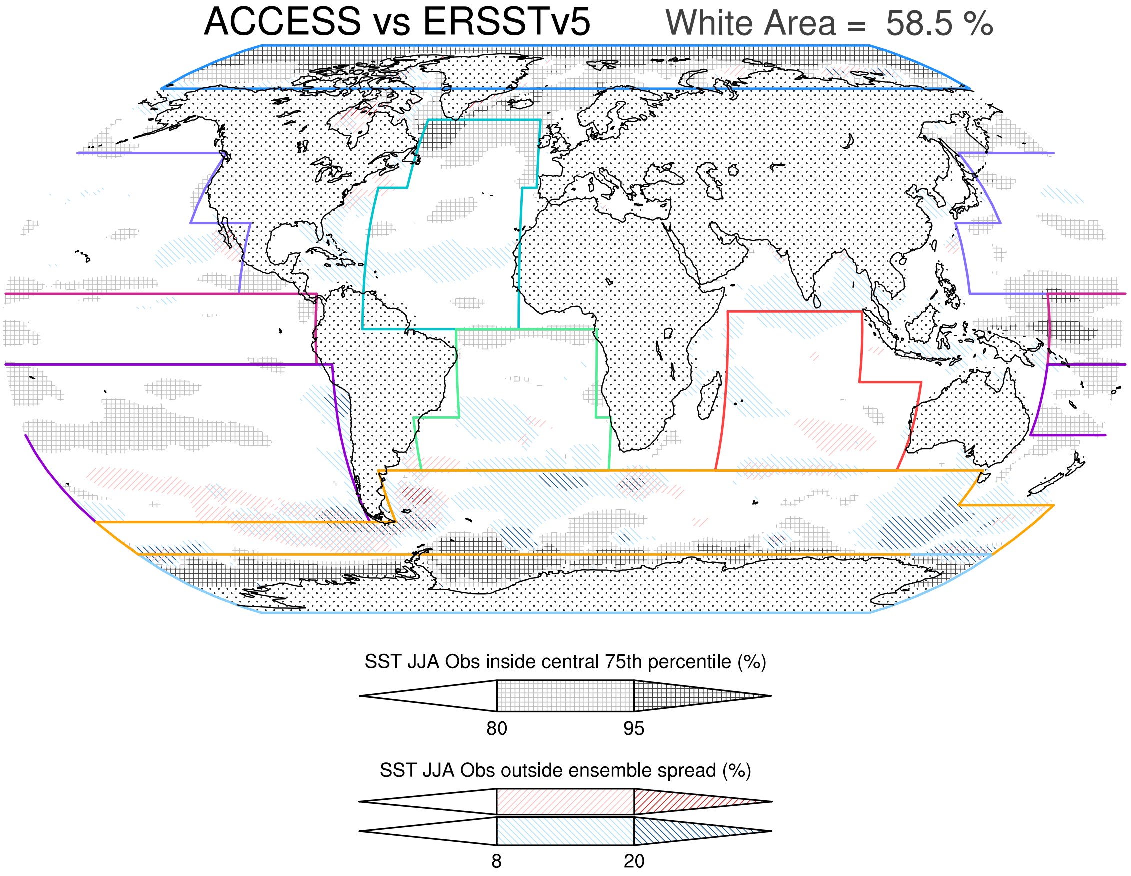
ACCESS vs ERSSTv5
White Area = 58.5 %
SST JJA Obs inside central 75th percentile (%)
80
95
SST JJA Obs outside ensemble spread (%)
8
20

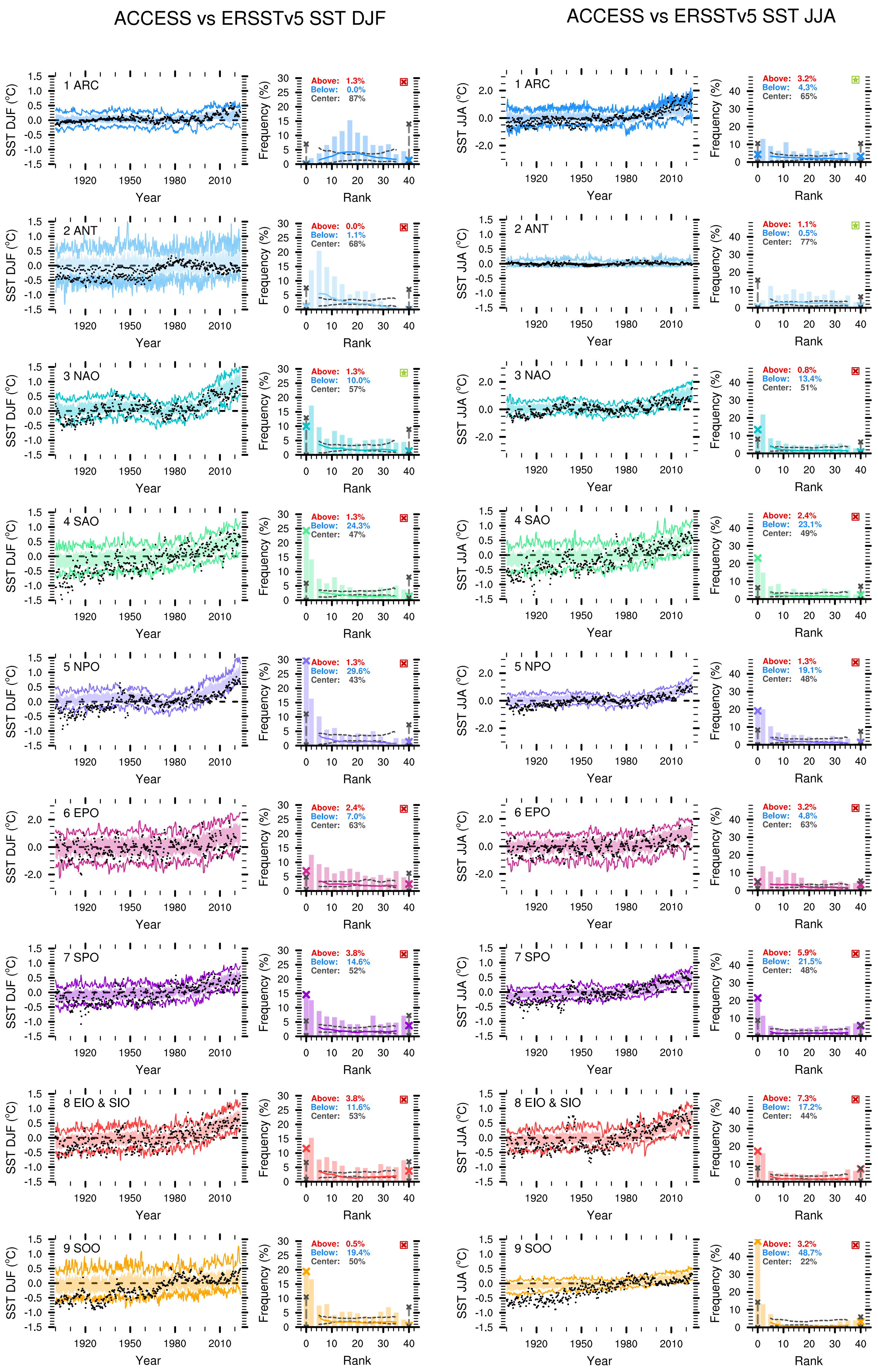
ACCESS vs ERSSTv5 SST DJF
ACCESS vs ERSSTv5 SST JJA
1 ARC
Above: 1.3% Below: 0.0% Center: 87%
Above: 3.2% Below: 4.3% Center: 65%
2 ANT
Above: 0.0% Below: 1.1% Center: 68%
Above: 1.1% Below: 0.5% Center: 77%
3 NAO
Above: 1.3% Below: 10.0% Center: 57%
Above: 0.8% Below: 13.4% Center: 51%
4 SAO
Above: 1.3% Below: 24.3% Center: 47%
Above: 2.4% Below: 23.1% Center: 49%
5 NPO
Above: 1.3% Below: 29.6% Center: 43%
Above: 1.3% Below: 19.1% Center: 48%
6 EPO
Above: 2.4% Below: 7.0% Center: 63%
Above: 3.2% Below: 4.8% Center: 63%
7 SPO
Above: 3.8% Below: 14.6% Center: 52%
Above: 5.9% Below: 21.5% Center: 48%
8 EIO & SIO
Above: 3.8% Below: 11.6% Center: 53%
Above: 7.3% Below: 17.2% Center: 44%
9 SOO
Above: 0.5% Below: 19.4% Center: 50%
Above: 3.2% Below: 48.7% Center: 22%
SST DJF (°C)
SST JJA (°C)
Year
Frequency (%)
Rank

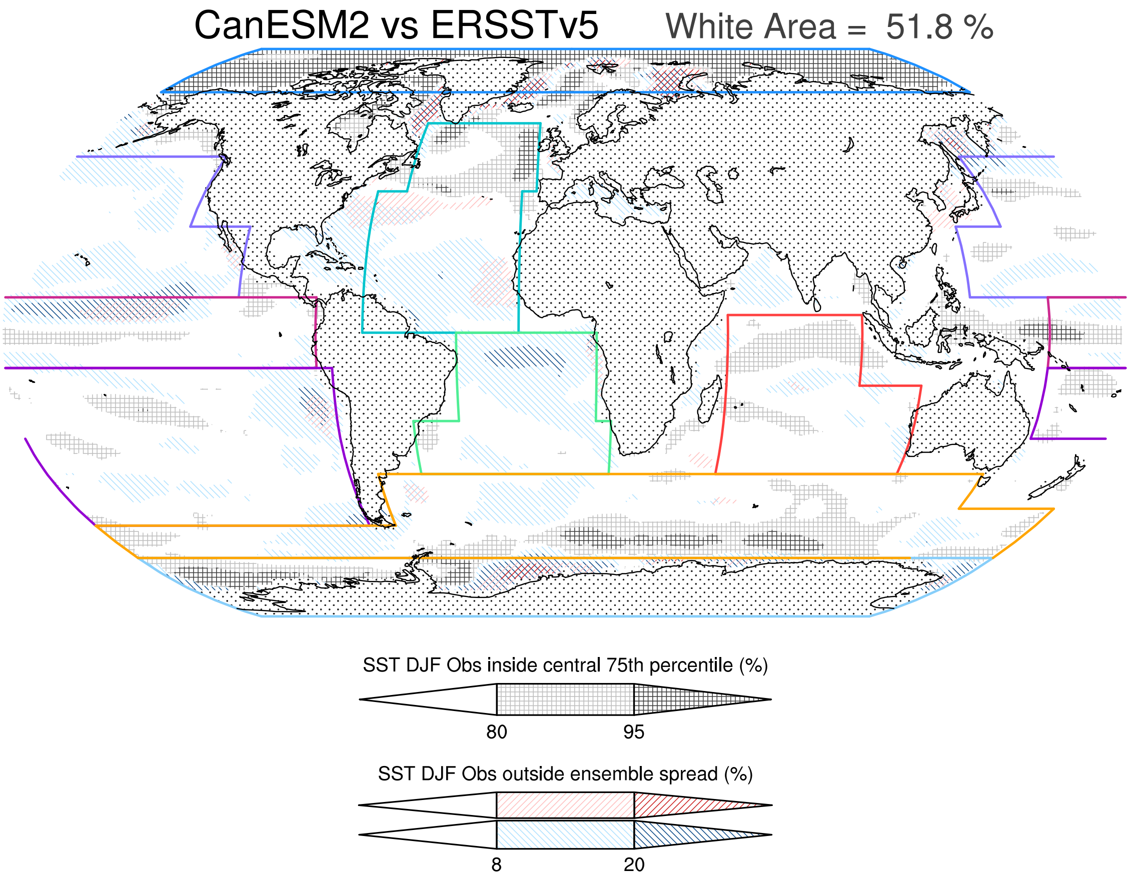
CanESM2 vs ERSSTv5
White Area = 51.8 %
SST DJF Obs inside central 75th percentile (%)
80
95
SST DJF Obs outside ensemble spread (%)
8
20

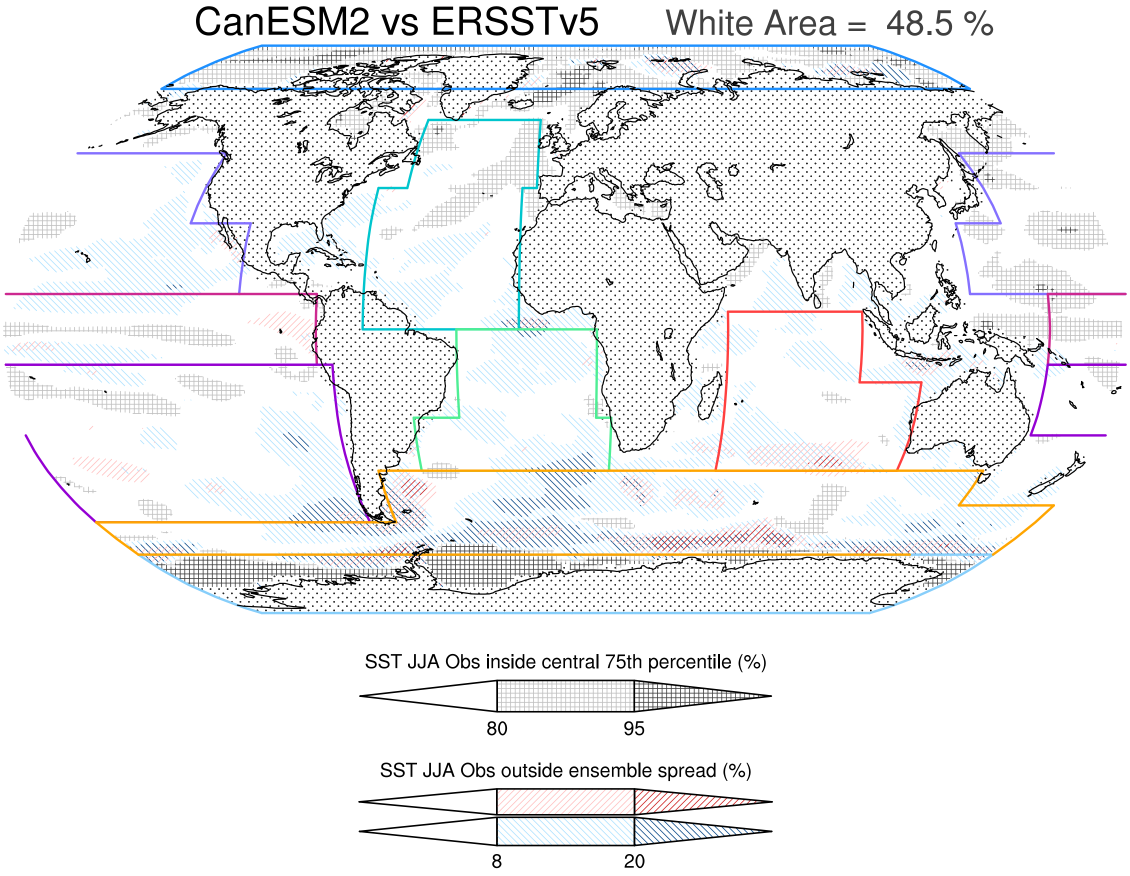
CanESM2 vs ERSSTv5
White Area = 48.5 %
SST JJA Obs inside central 75th percentile (%)
80
95
SST JJA Obs outside ensemble spread (%)
8
20

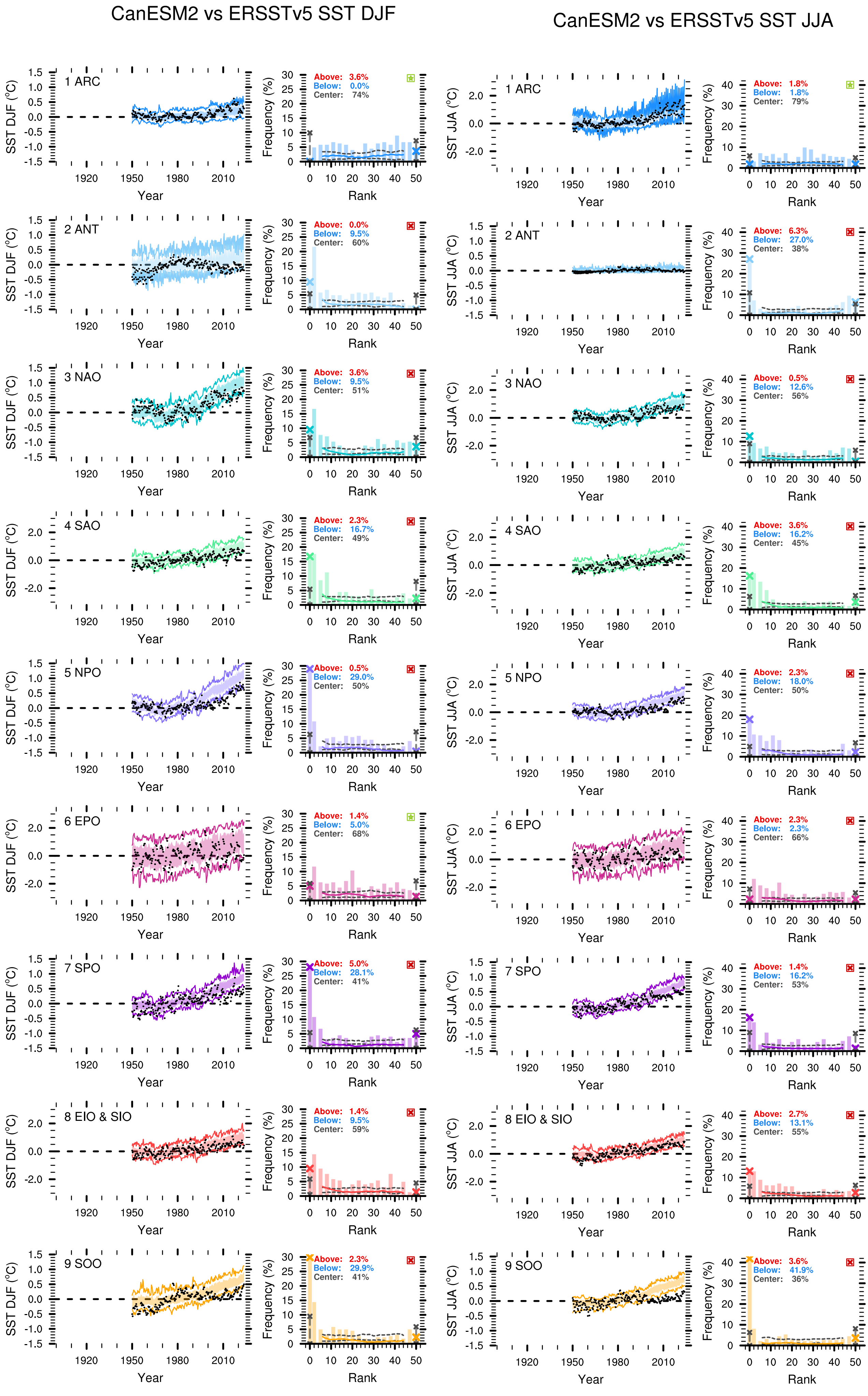
CanESM2 vs ERSSTv5 SST DJF
CanESM2 vs ERSSTv5 SST JJA
1 ARC
2 ANT
3 NAO
4 SAO
5 NPO
6 EPO
7 SPO
8 EIO & SIO
9 SOO
SST DJF (°C)
SST JJA (°C)
Year
Frequency (%)
Rank
Above: 3.6% Below: 0.0% Center: 74%
Above: 1.8% Below: 1.8% Center: 79%
Above: 0.0% Below: 9.5% Center: 60%
Above: 6.3% Below: 27.0% Center: 38%
Above: 3.6% Below: 9.5% Center: 51%
Above: 0.5% Below: 12.6% Center: 56%
Above: 2.3% Below: 16.7% Center: 49%
Above: 3.6% Below: 16.2% Center: 45%
Above: 0.5% Below: 29.0% Center: 50%
Above: 2.3% Below: 18.0% Center: 50%
Above: 1.4% Below: 5.0% Center: 68%
Above: 2.3% Below: 2.3% Center: 66%
Above: 5.0% Below: 28.1% Center: 41%
Above: 1.4% Below: 16.2% Center: 53%
Above: 1.4% Below: 9.5% Center: 59%
Above: 2.7% Below: 13.1% Center: 55%
Above: 2.3% Below: 29.9% Center: 41%
Above: 3.6% Below: 41.9% Center: 36%

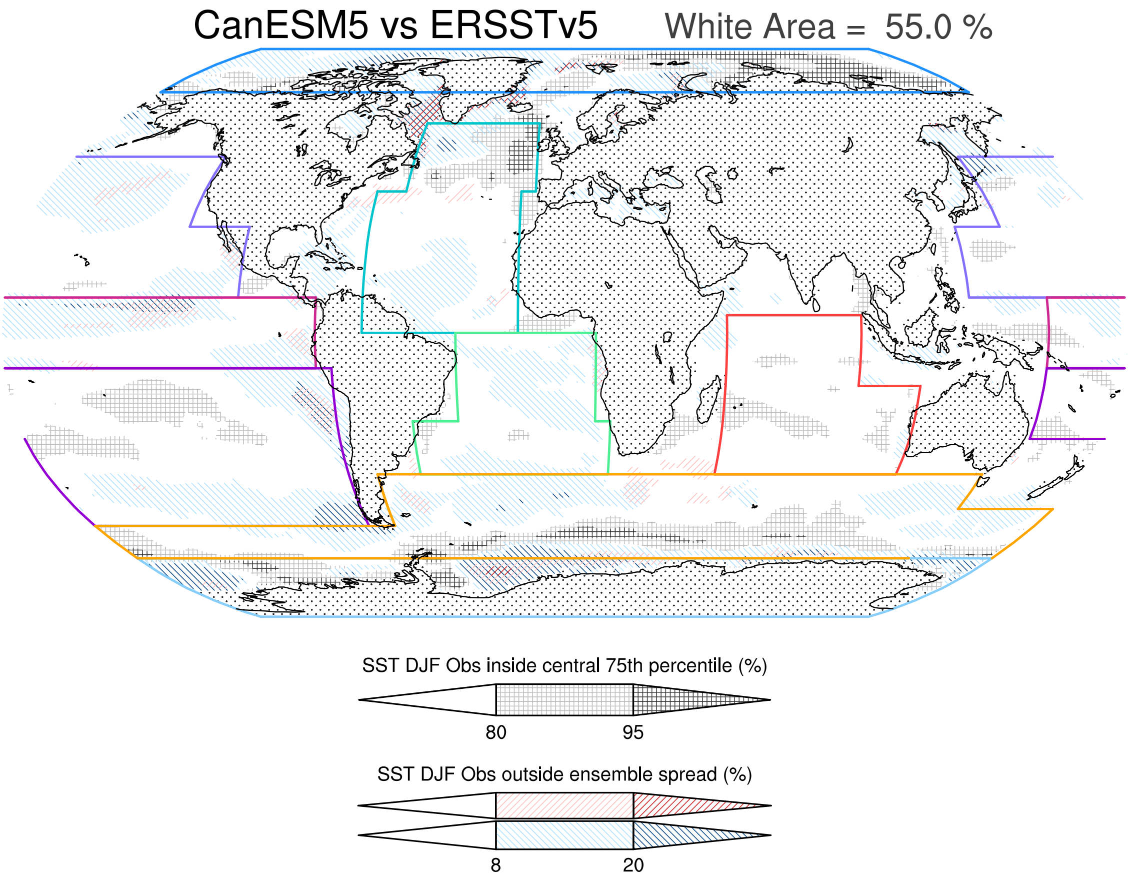

CanESM5 vs ERSSTv5
White Area = 55.0 %
SST DJF Obs inside central 75th percentile (%)
80
95
SST DJF Obs outside ensemble spread (%)
8
20

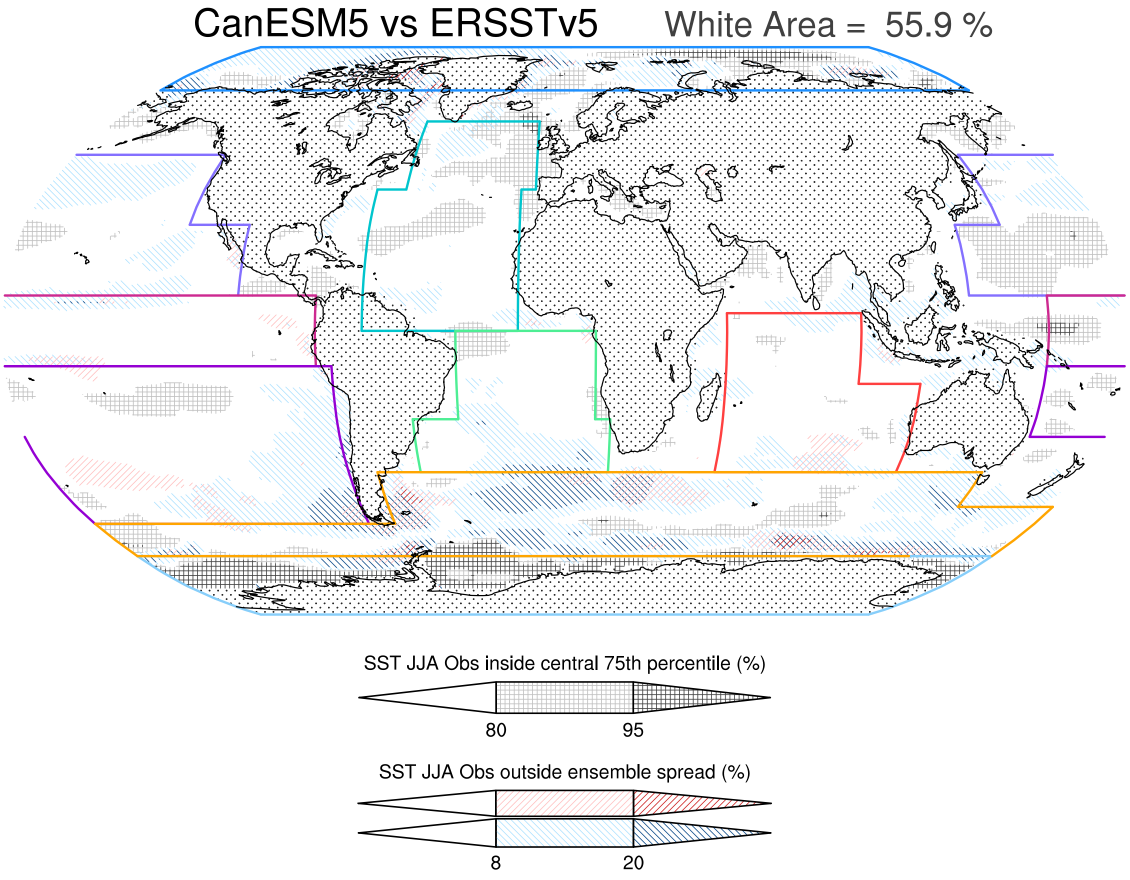

CanESM5 vs ERSSTv5
White Area = 55.9 %
SST JJA Obs inside central 75th percentile (%)
80
95
SST JJA Obs outside ensemble spread (%)
8
20

CanESM5 vs ERSSTv5 SST DJF

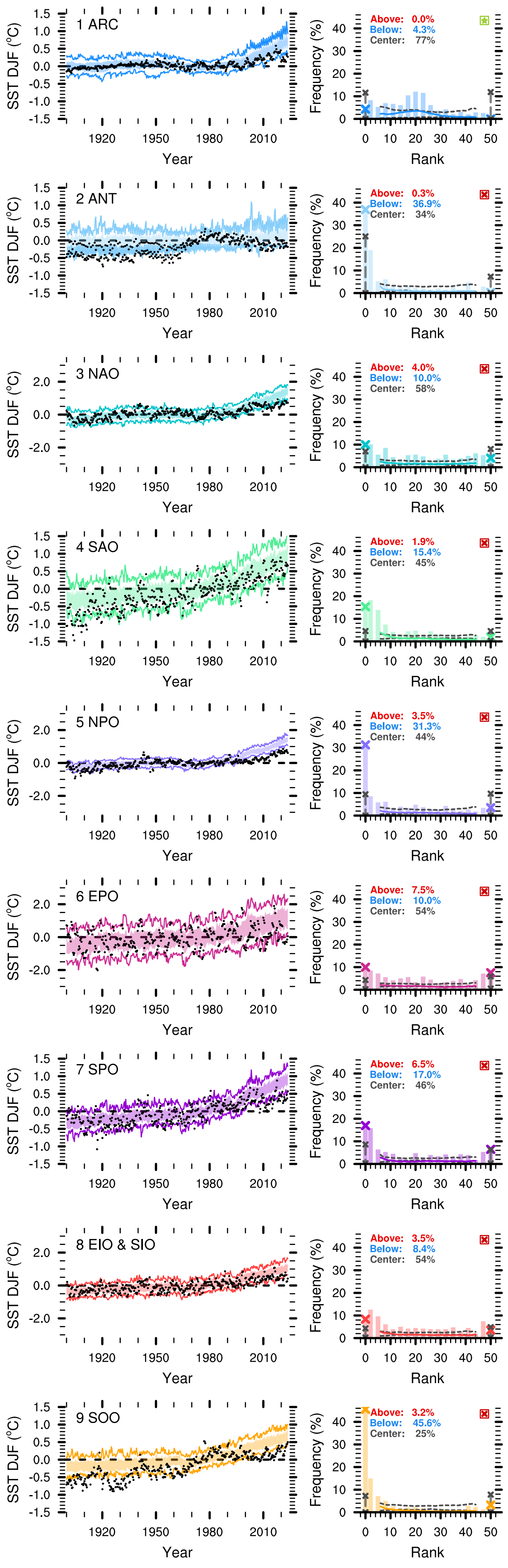

CanESM5 vs ERSSTv5 SST JJA

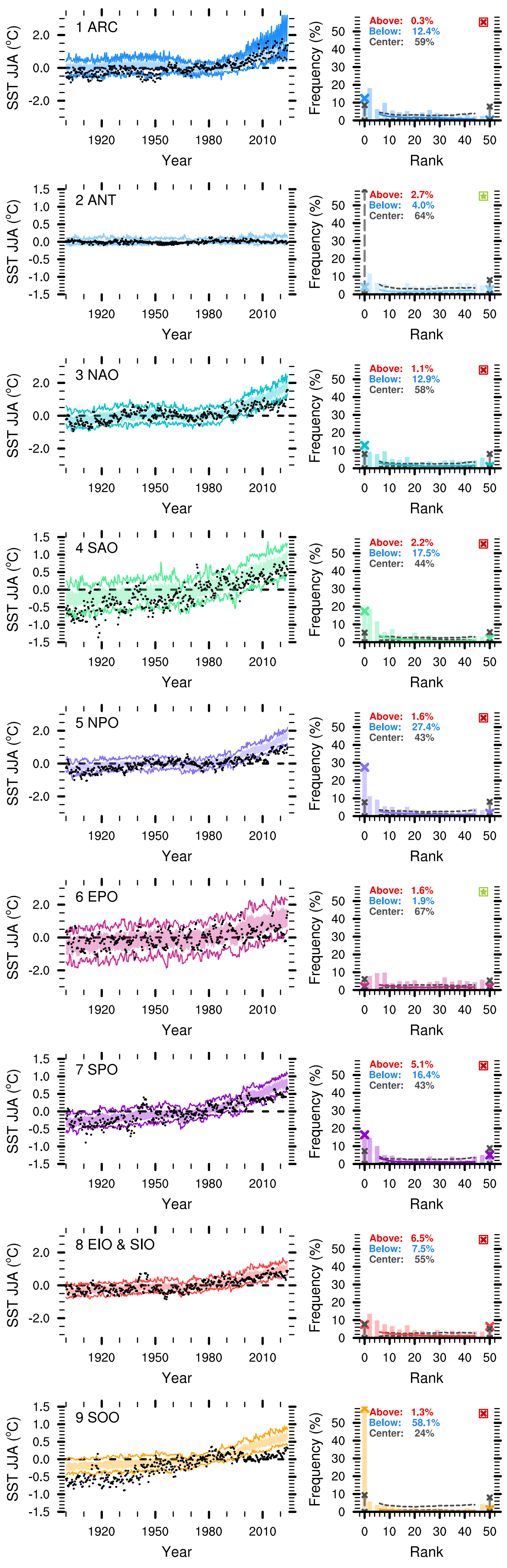

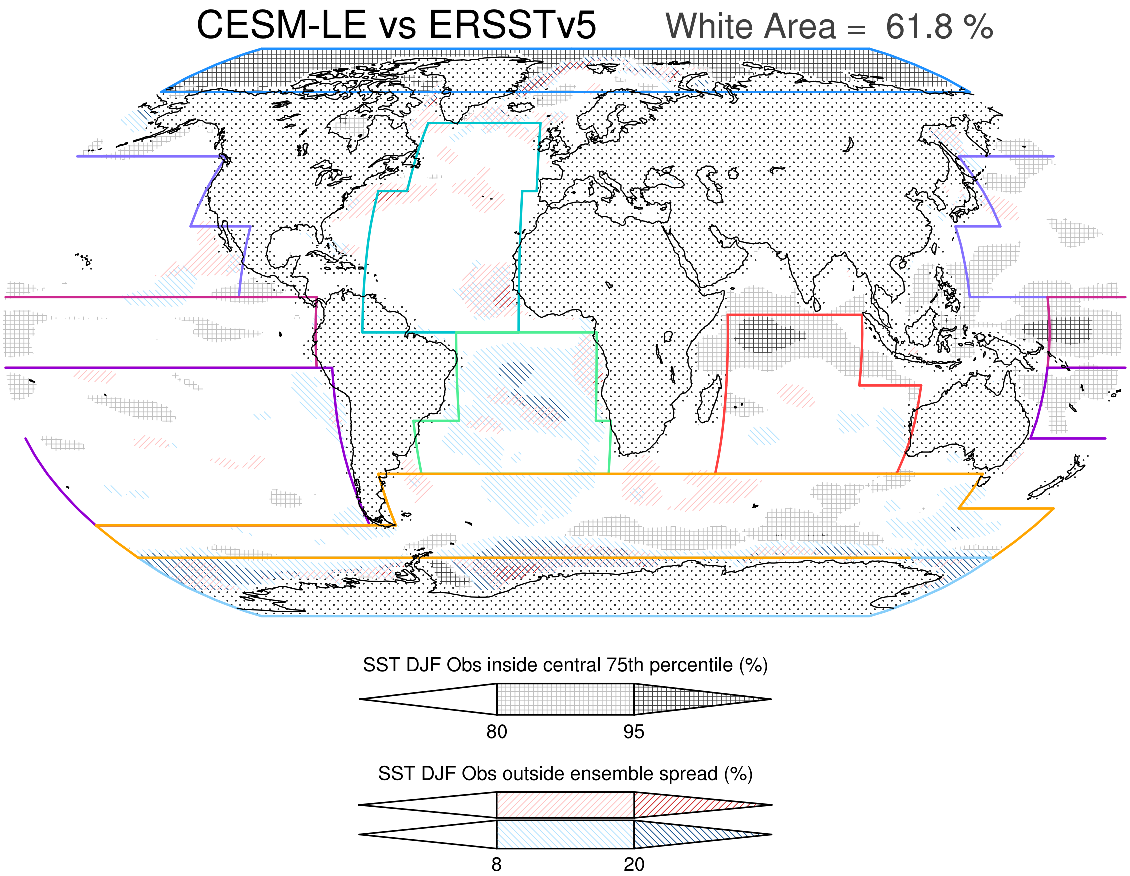
CESM-LE vs ERSSTv5
White Area = 61.8 %
SST DJF Obs inside central 75th percentile (%)
80
95
SST DJF Obs outside ensemble spread (%)
8
20

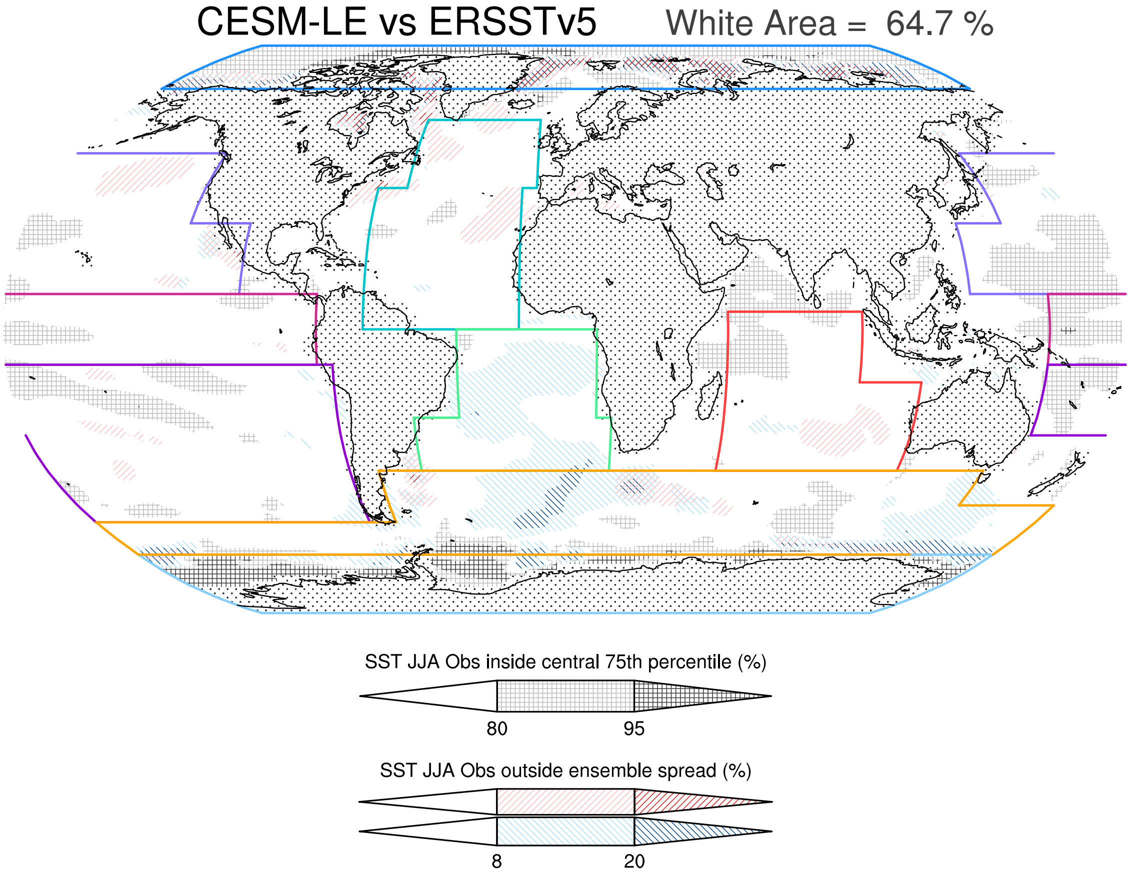
CESM-LE vs ERSSTv5
White Area = 64.7 %
SST JJA Obs inside central 75th percentile (%)
80
95
SST JJA Obs outside ensemble spread (%)
8
20

CESM-LE vs ERSSTv5 SST DJF

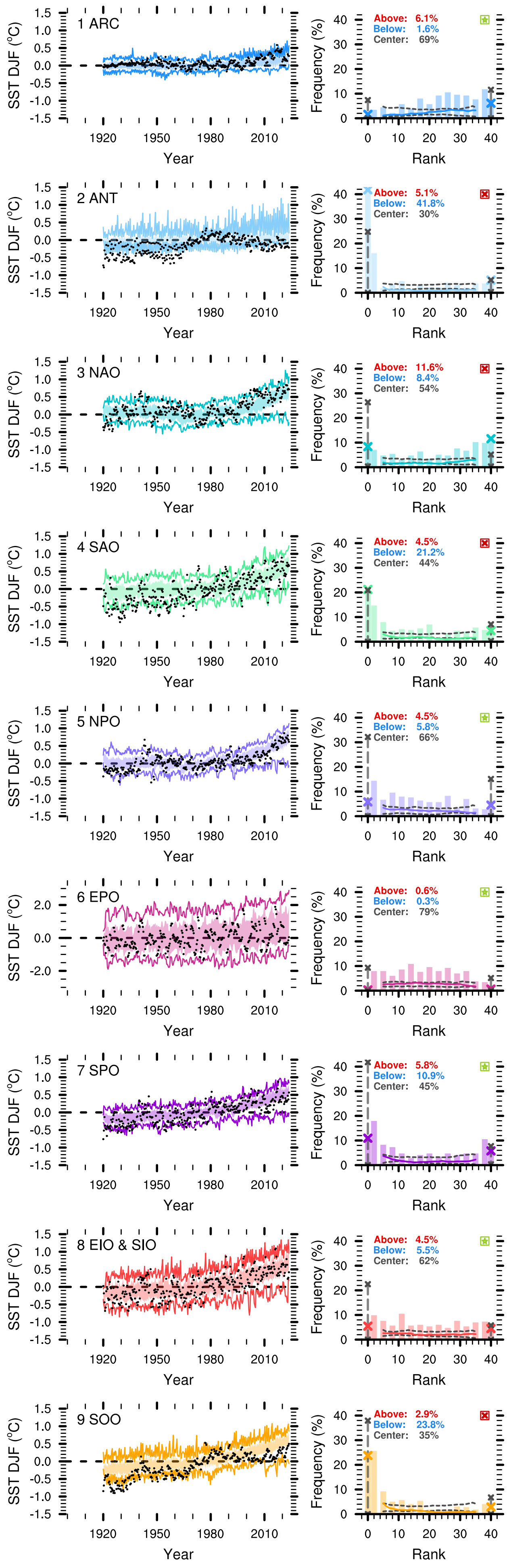

CESM-LE vs ERSSTv5 SST JJA

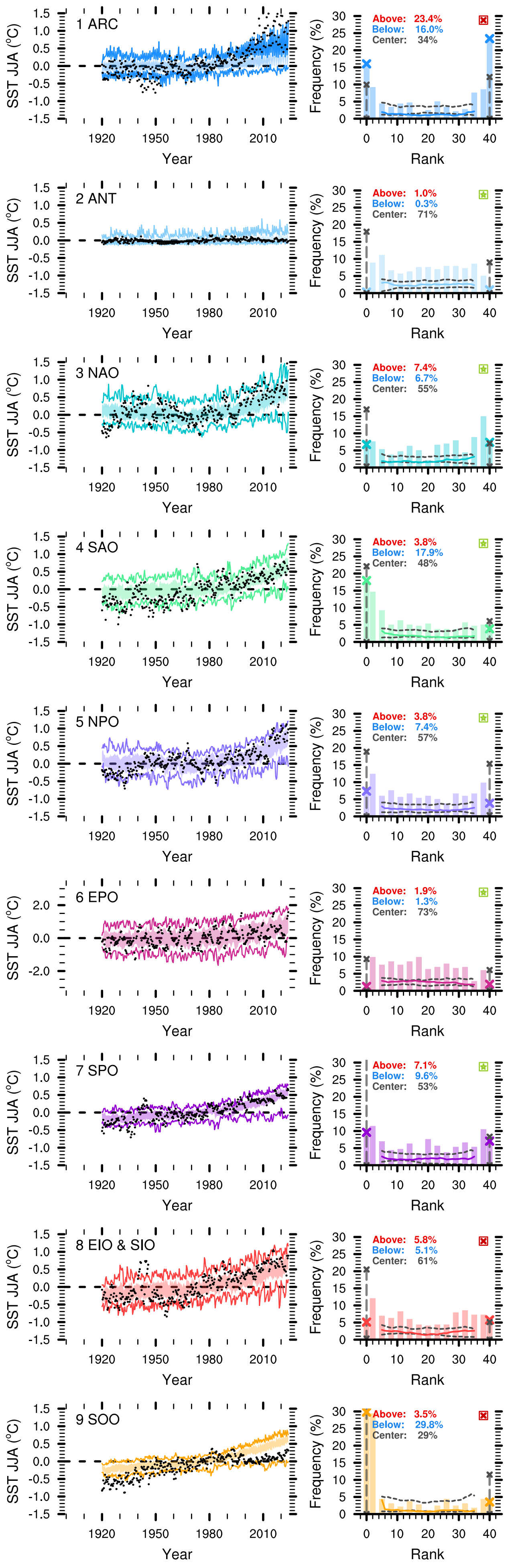

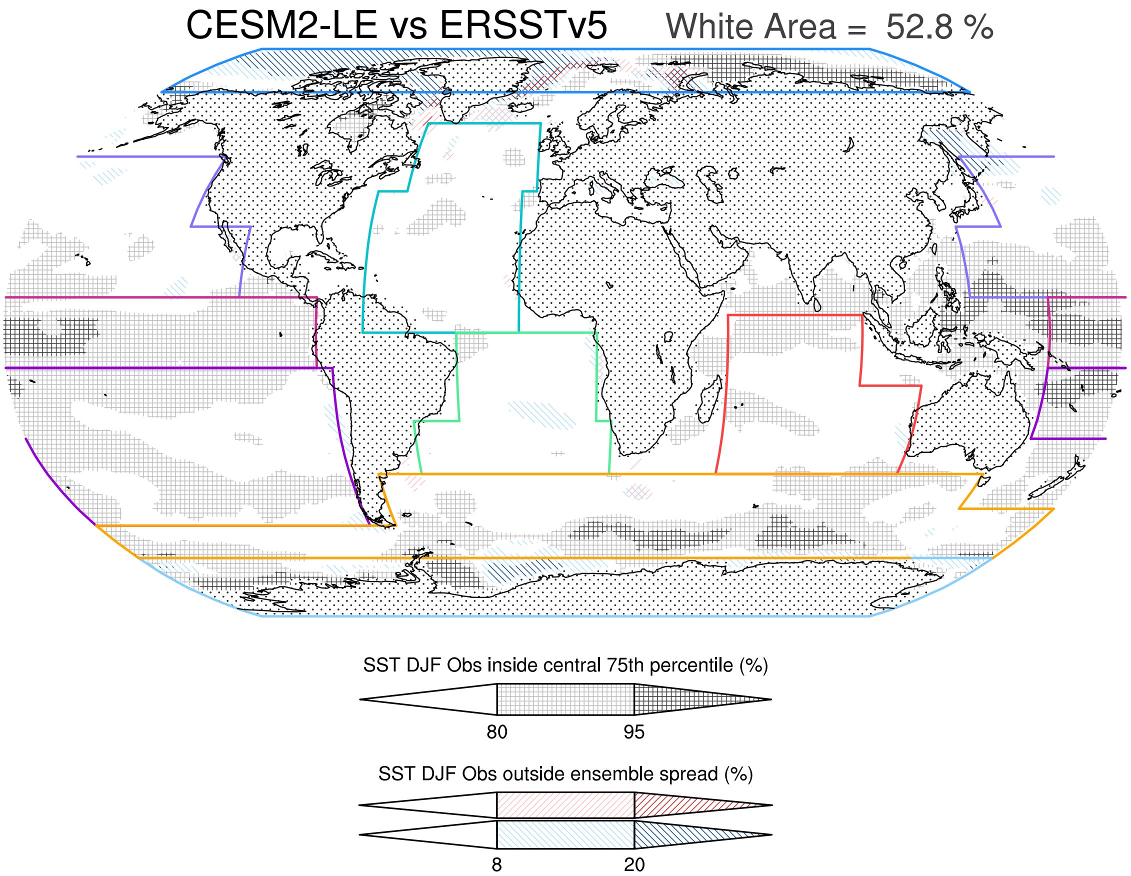

CESM2-LE vs ERSSTv5
White Area = 52.8 %
SST DJF Obs inside central 75th percentile (%)
80
95
SST DJF Obs outside ensemble spread (%)
8
20

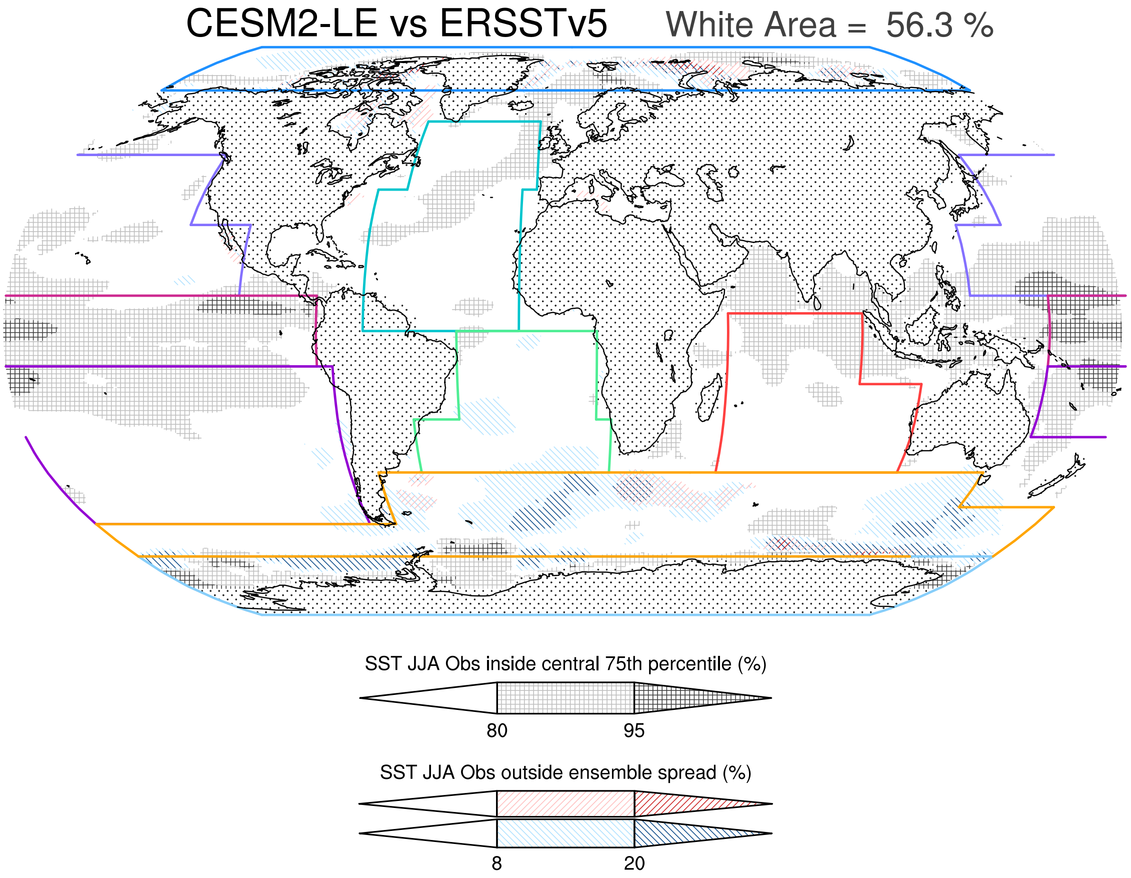

CESM2-LE vs ERSSTv5
White Area = 56.3 %
SST JJA Obs inside central 75th percentile (%)
80
95
SST JJA Obs outside ensemble spread (%)
8
20

CESM2-LE vs ERSSTv5 SST DJF

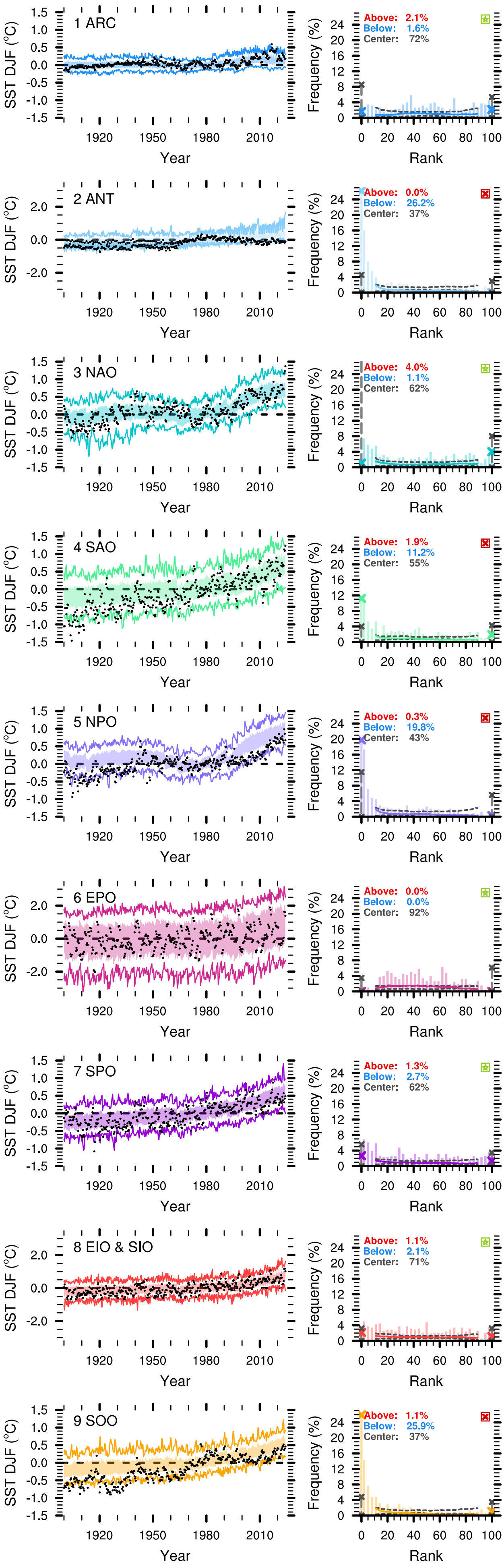

CESM2-LE vs ERSSTv5 SST JJA

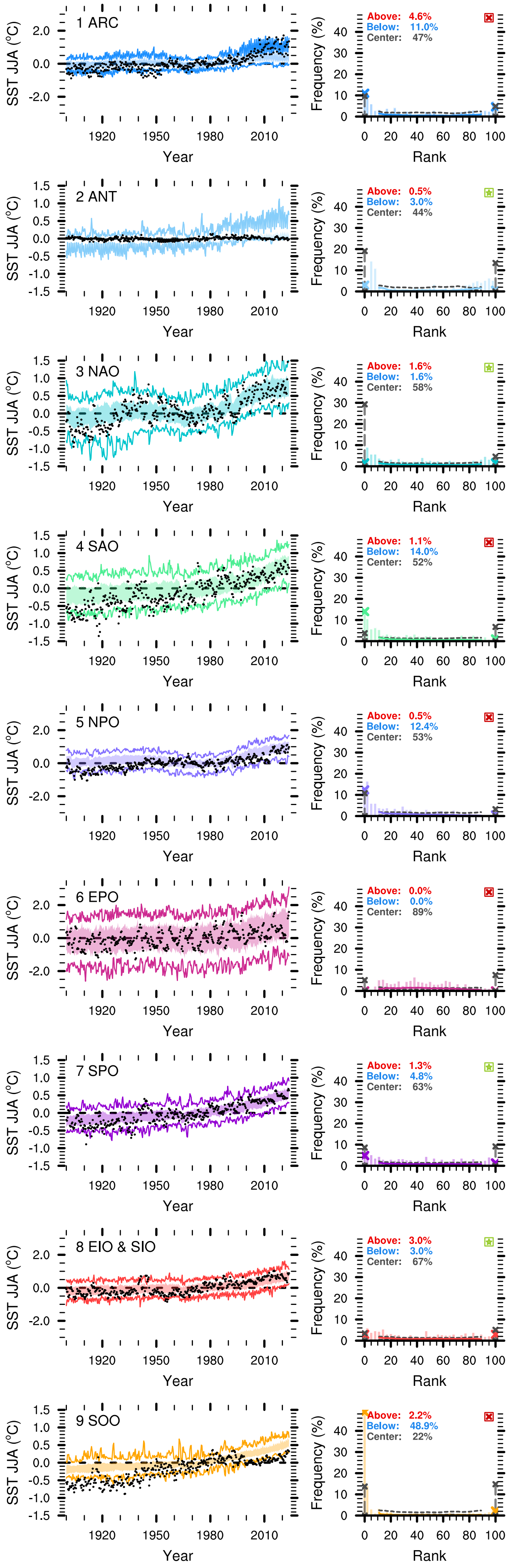

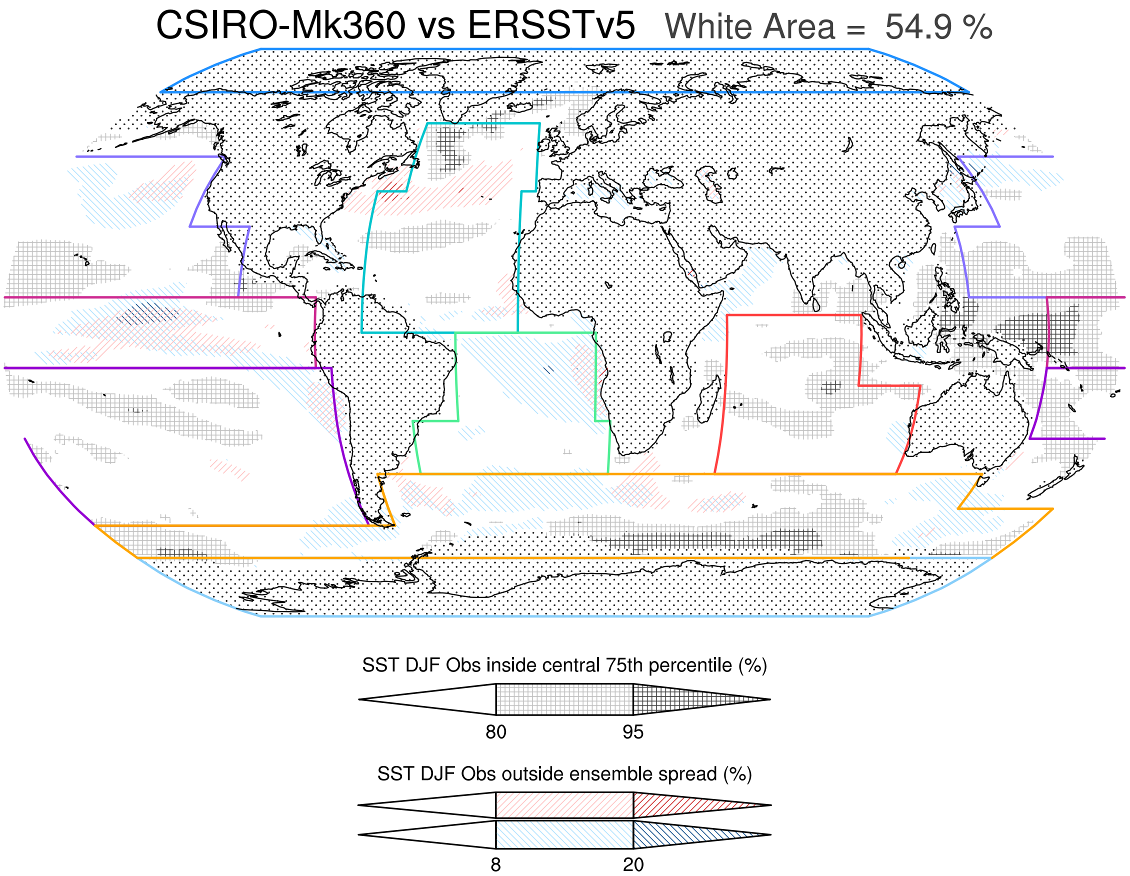
CSIRO-Mk360 vs ERSSTv5   White Area = 54.9 %
SST DJF Obs inside central 75th percentile (%)
80
95
SST DJF Obs outside ensemble spread (%)
8
20

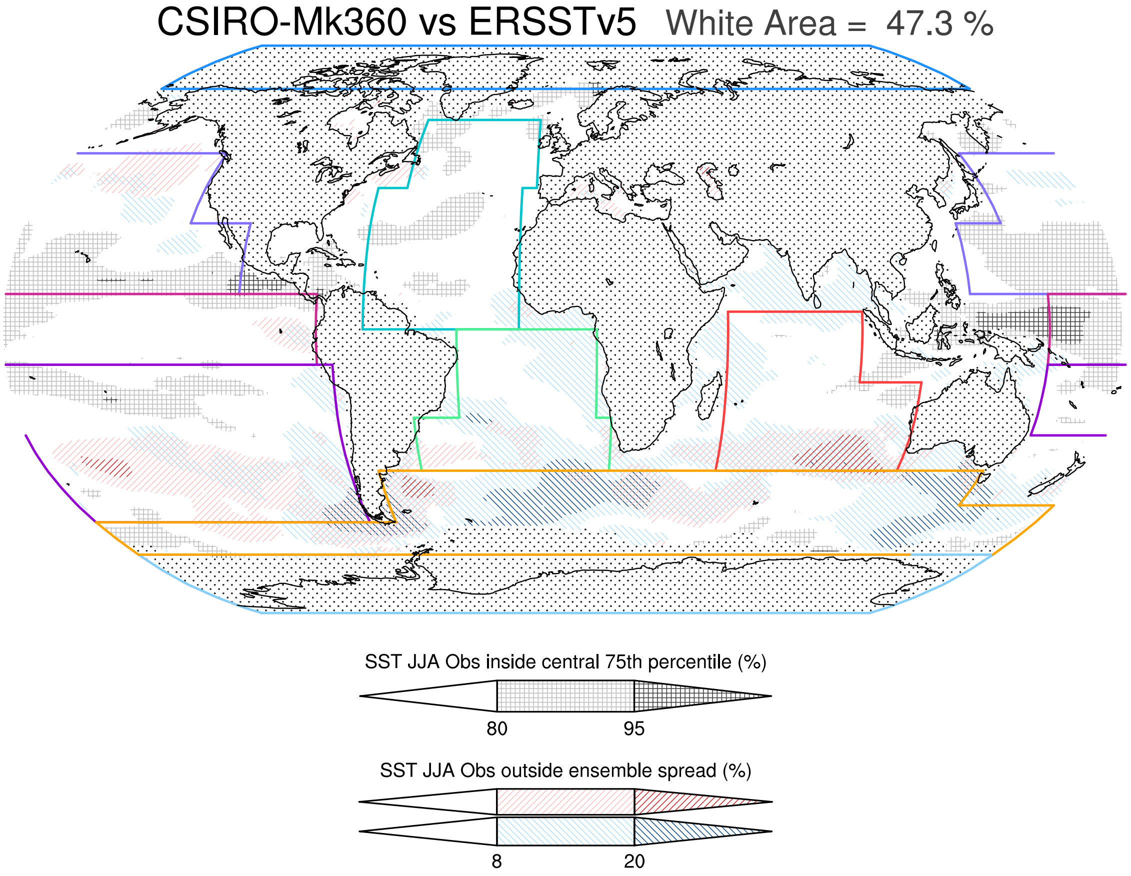
CSIRO-Mk360 vs ERSSTv5   White Area = 47.3 %
SST JJA Obs inside central 75th percentile (%)
80
95
SST JJA Obs outside ensemble spread (%)
8
20

CSIRO-Mk360 vs ERSSTv5 SST DJF

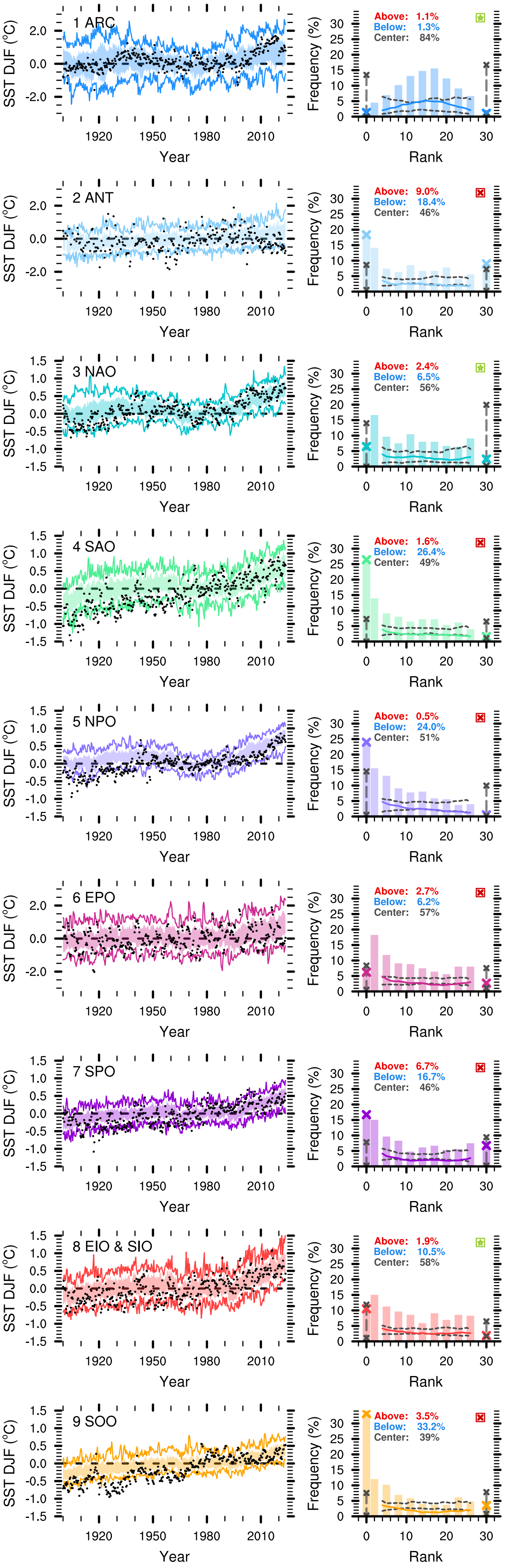

CSIRO-Mk360 vs ERSSTv5 SST JJA

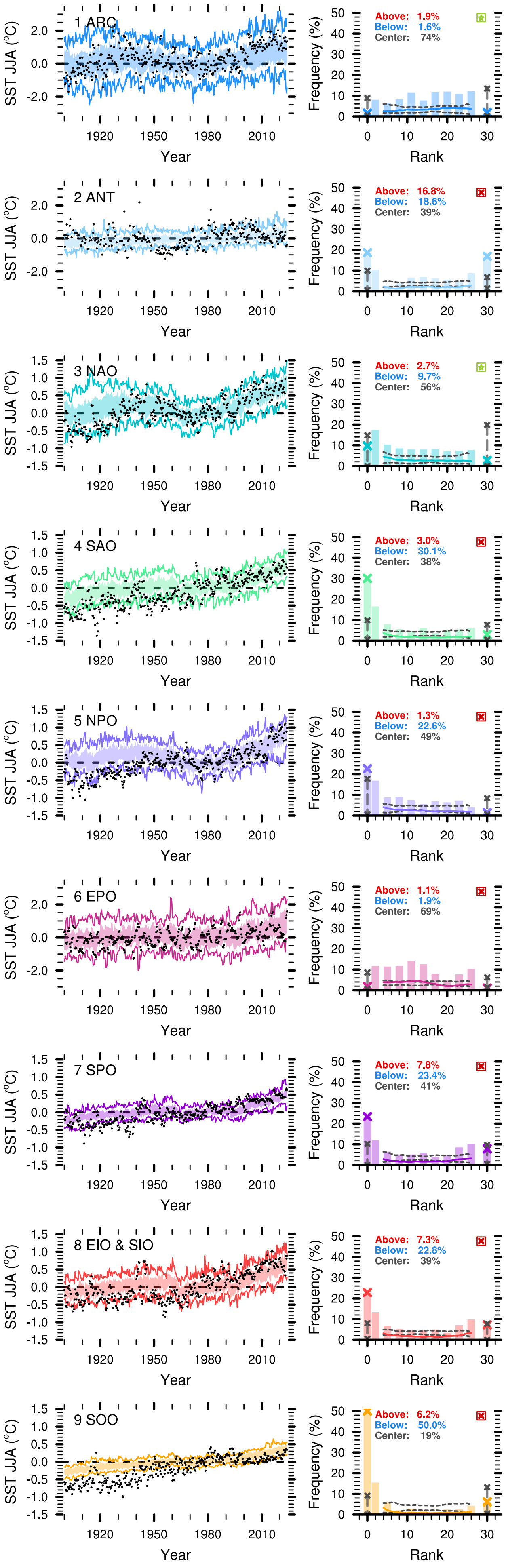

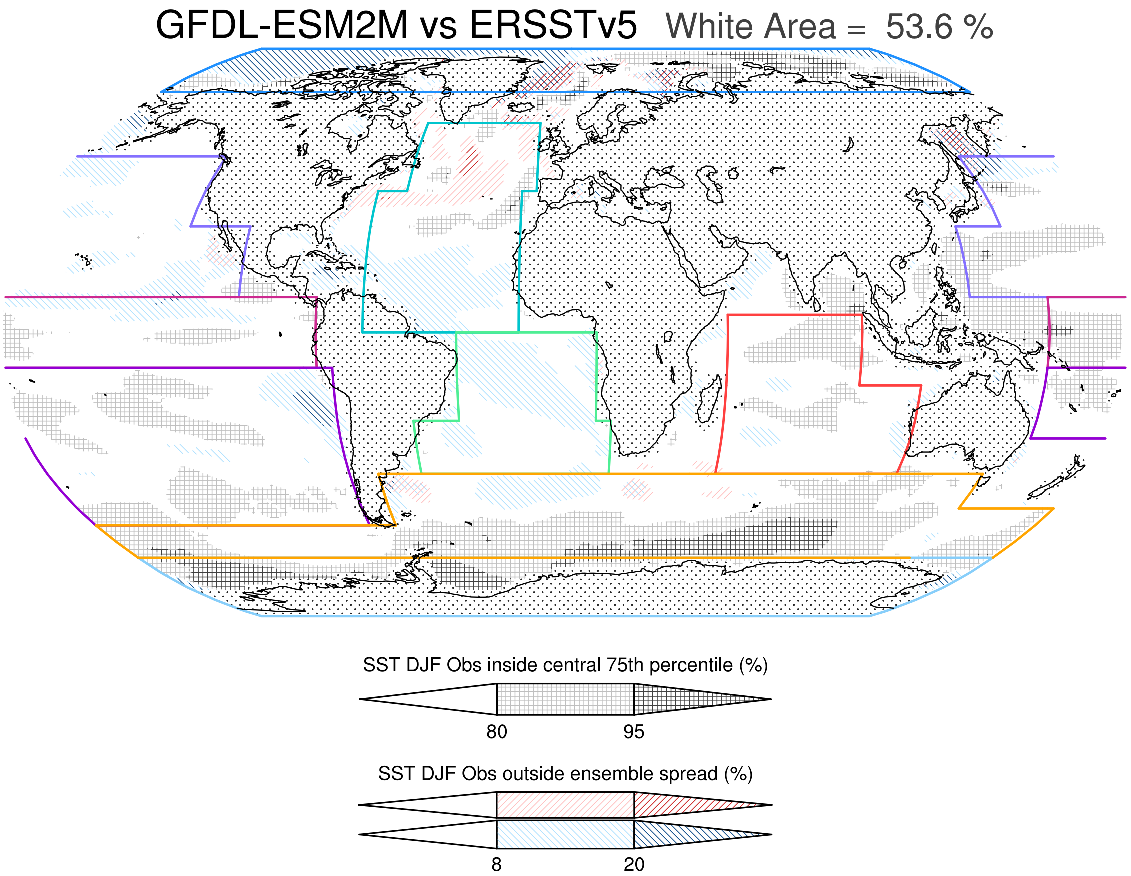
GFDL-ESM2M vs ERSSTv5 White Area = 53.6 %
SST DJF Obs inside central 75th percentile (%)
80
95
SST DJF Obs outside ensemble spread (%)
8
20

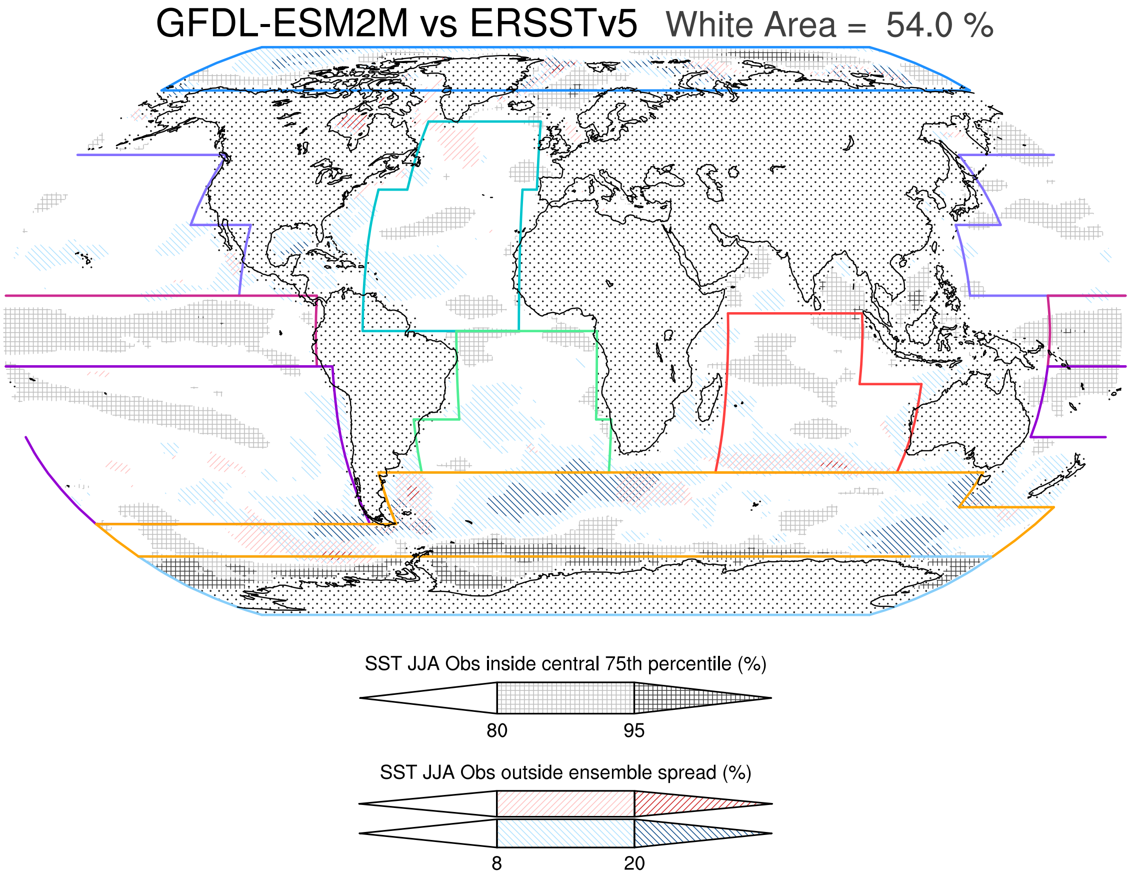
GFDL-ESM2M vs ERSSTv5 White Area = 54.0 %
SST JJA Obs inside central 75th percentile (%)
80
95
SST JJA Obs outside ensemble spread (%)
8
20

GFDL-ESM2M vs ERSSTv5 SST DJF   GFDL-ESM2M vs ERSSTv5 SST JJA

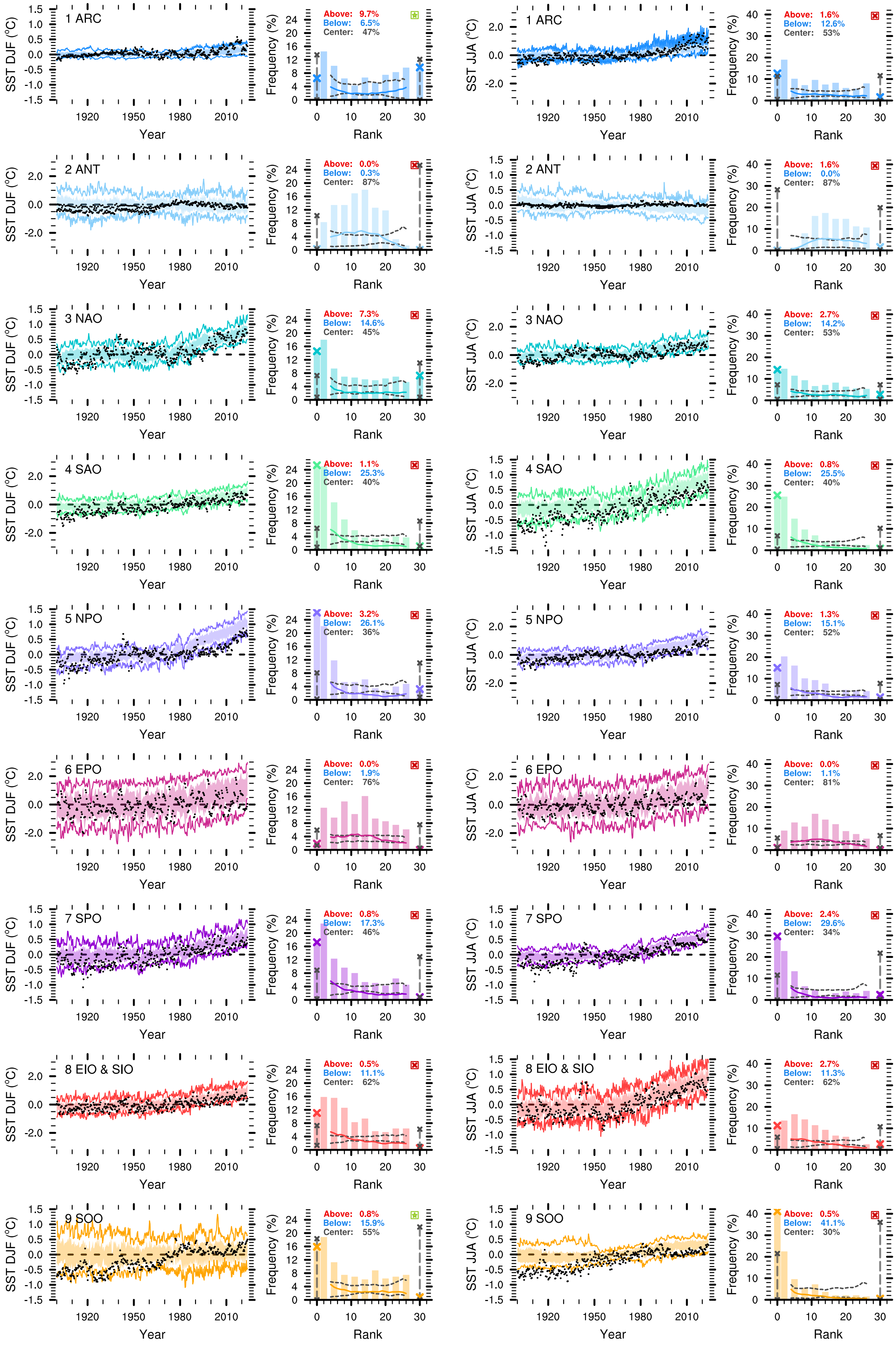

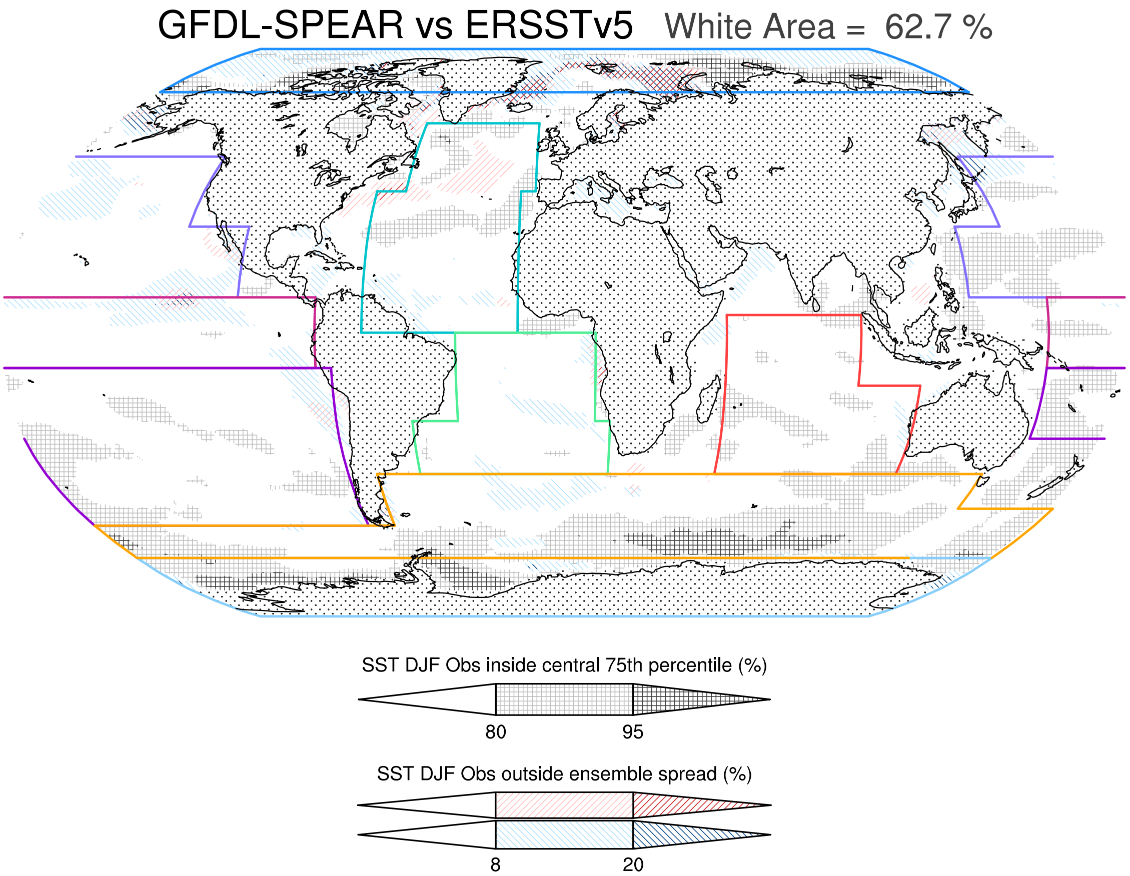
GFDL-SPEAR vs ERSSTv5 White Area = 62.7 %
SST DJF Obs inside central 75th percentile (%)
80
95
SST DJF Obs outside ensemble spread (%)
8
20

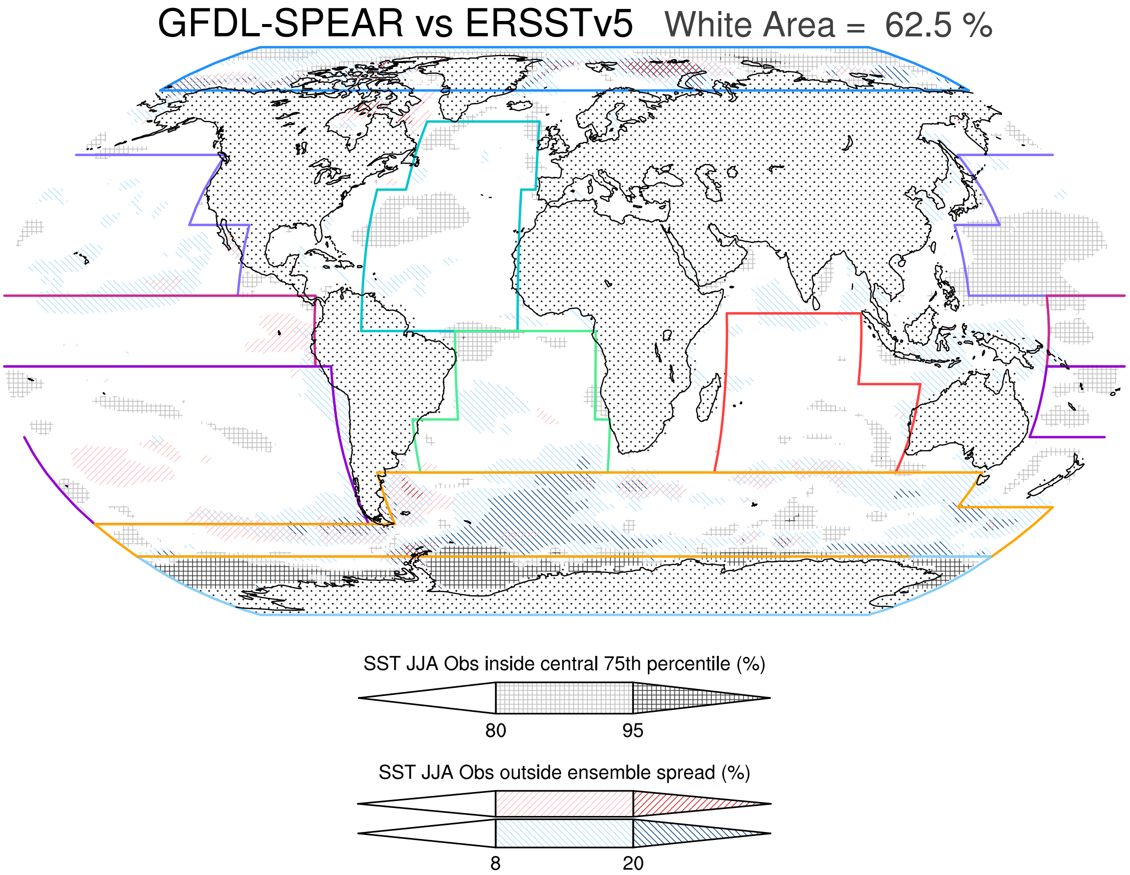
GFDL-SPEAR vs ERSSTv5 White Area = 62.5 %
SST JJA Obs inside central 75th percentile (%)
80
95
SST JJA Obs outside ensemble spread (%)
8
20

GFDL-SPEAR vs ERSSTv5 SST DJF   GFDL-SPEAR vs ERSSTv5 SST JJA

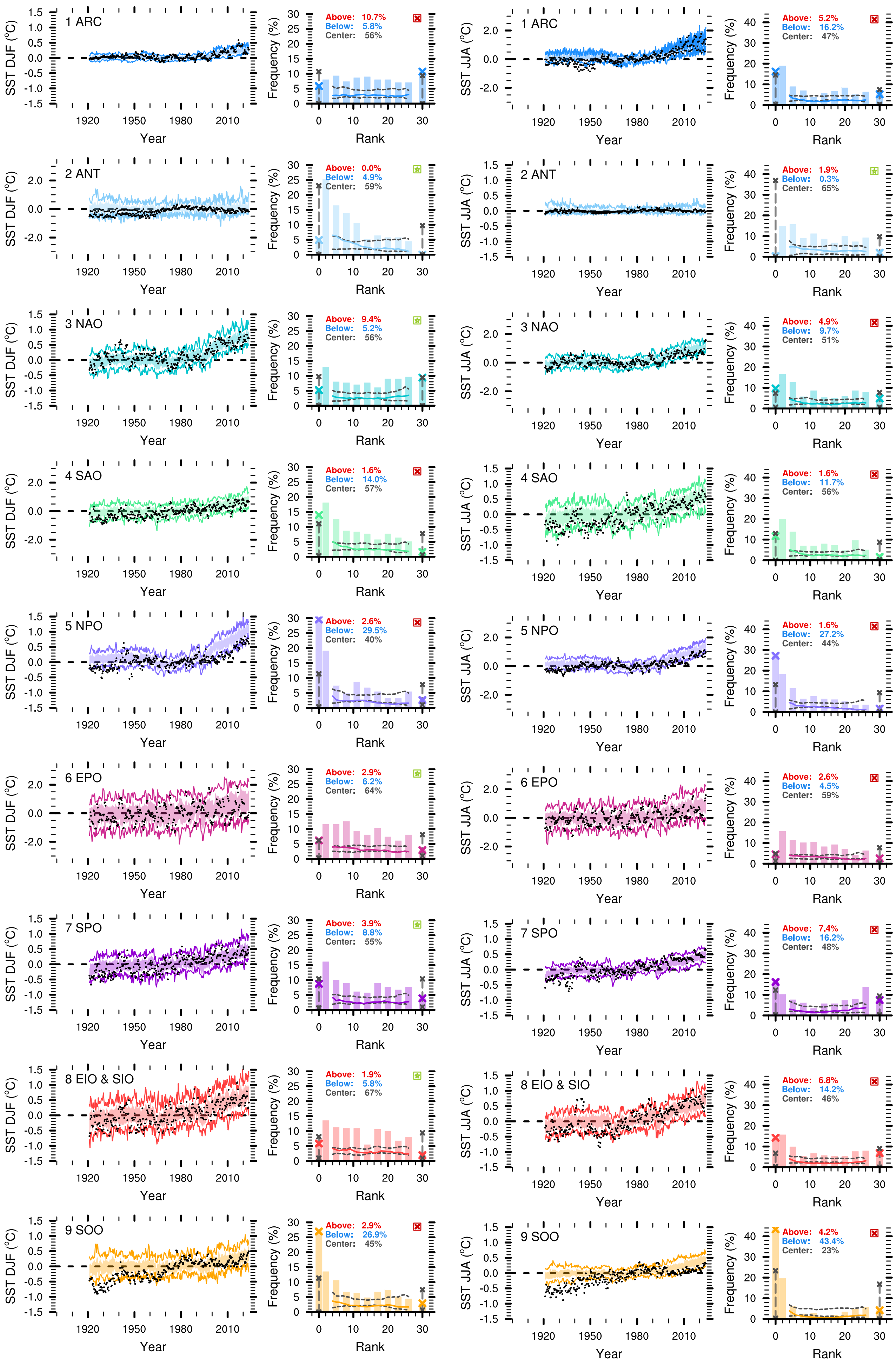

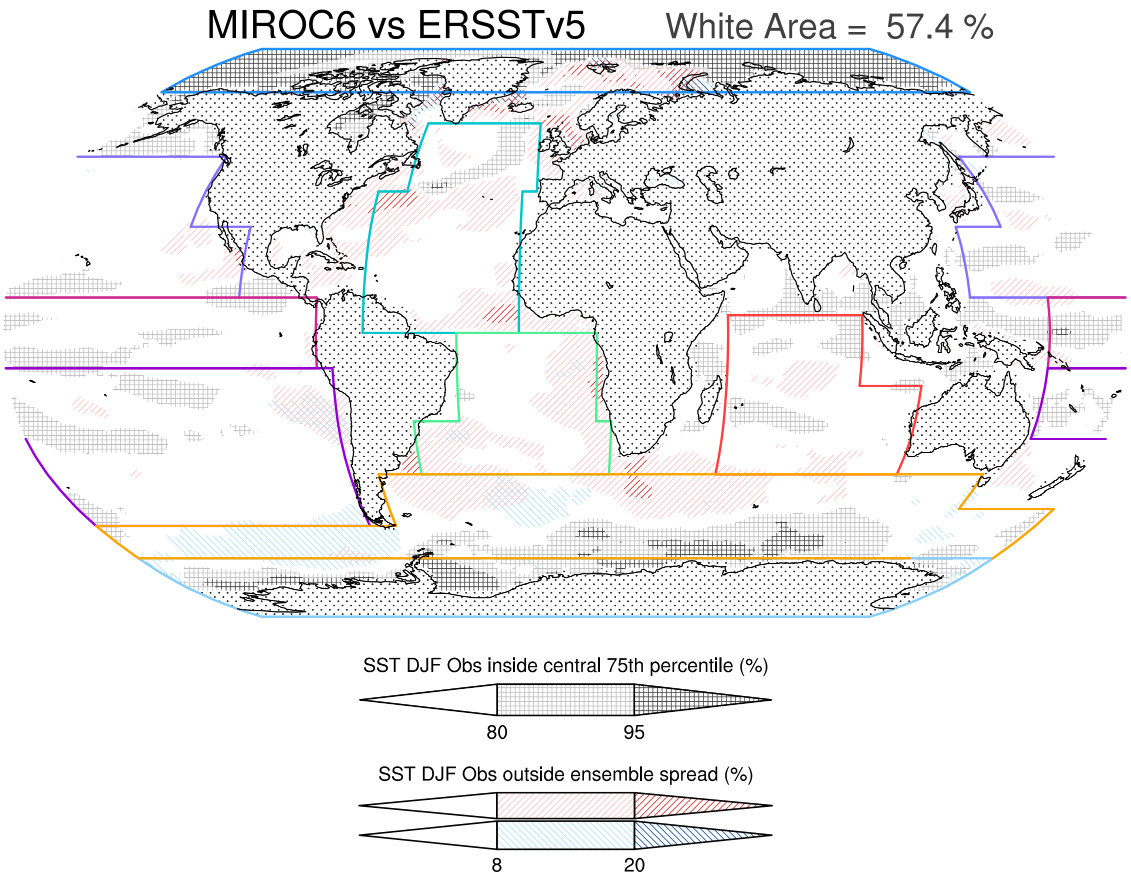
MIROC6 vs ERSSTv5
White Area = 57.4 %
SST DJF Obs inside central 75th percentile (%)
80
95
SST DJF Obs outside ensemble spread (%)
8
20

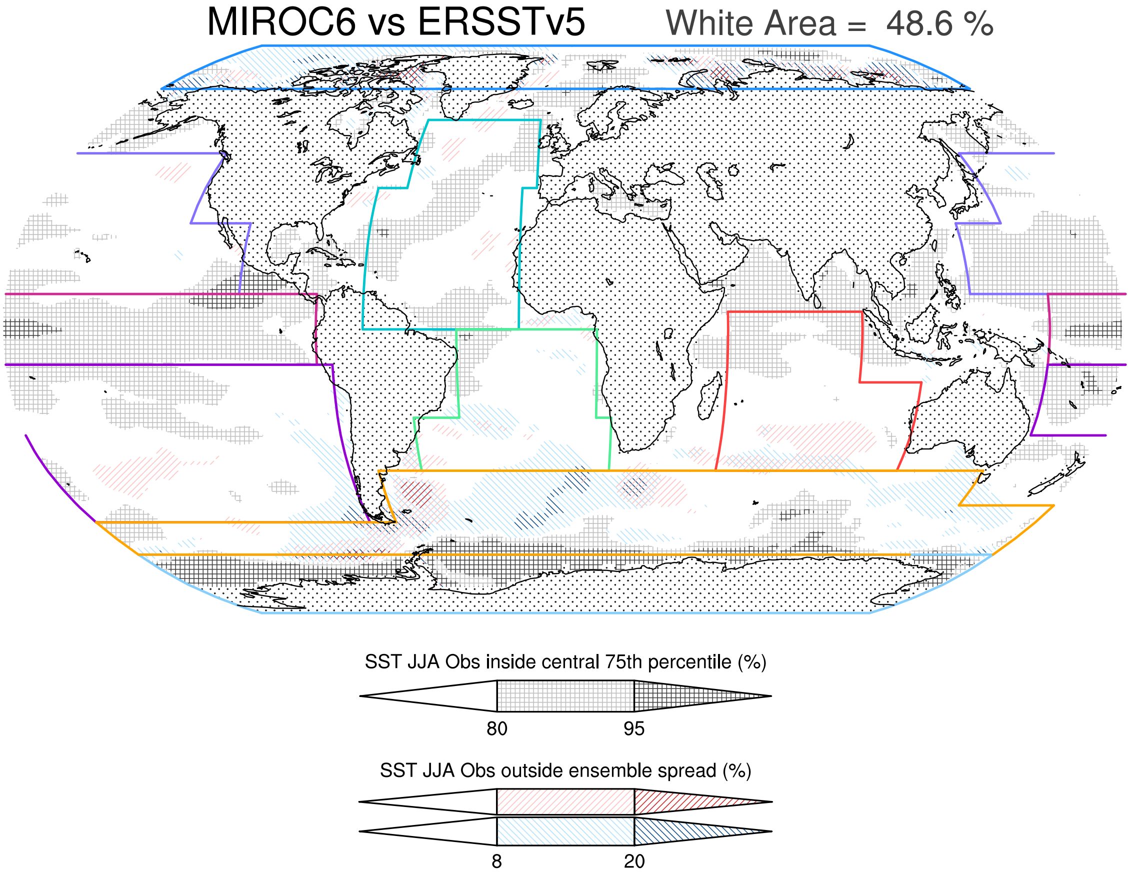
MIROC6 vs ERSSTv5
White Area = 48.6 %
SST JJA Obs inside central 75th percentile (%)
80
95
SST JJA Obs outside ensemble spread (%)
8
20

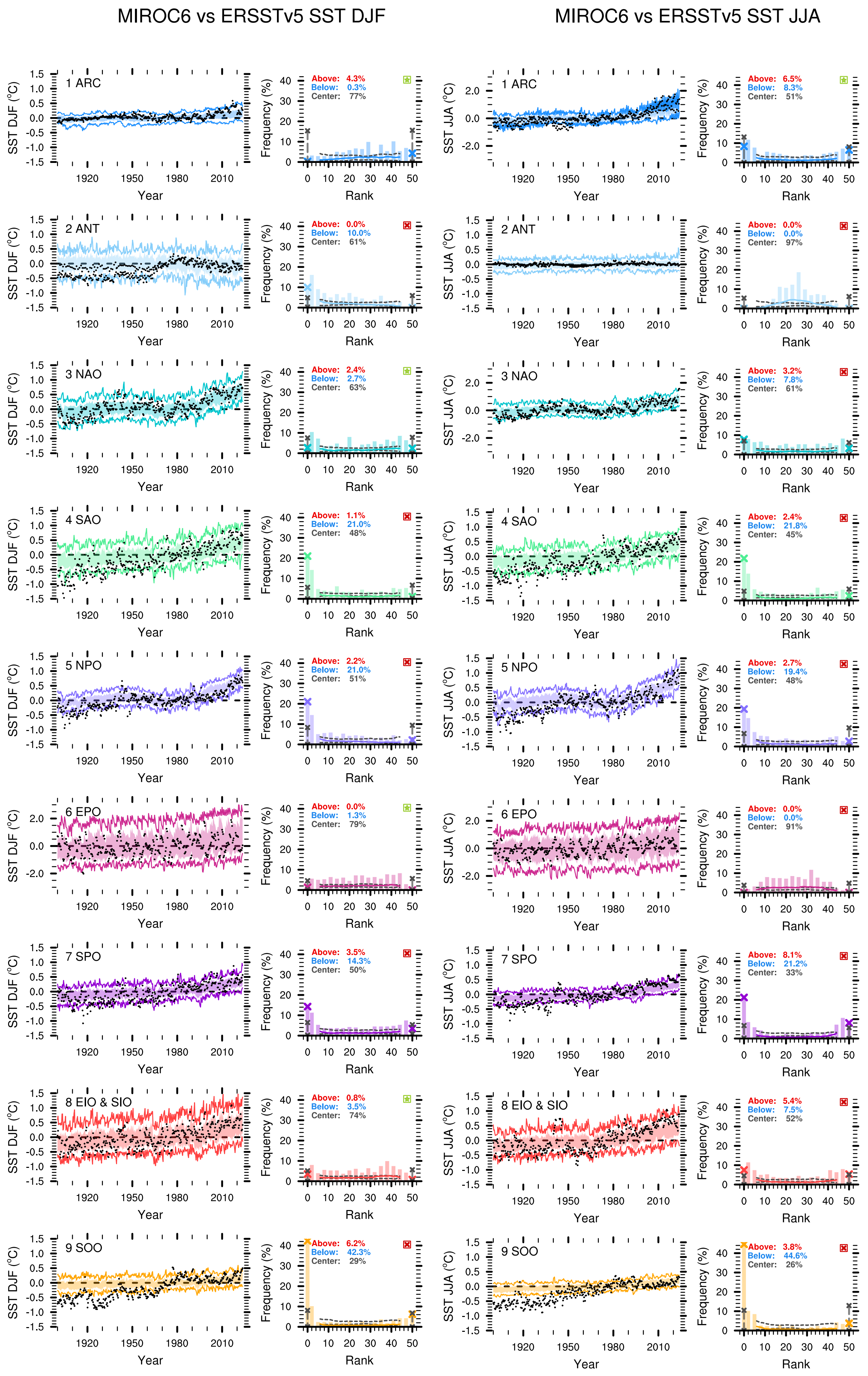

MIROC6 vs ERSSTv5 SST DJF
MIROC6 vs ERSSTv5 SST JJA
SST DJF (°C)
SST JJA (°C)
Year
Rank
Frequency (%)
1 ARC
Above: 4.3%
Below: 0.3%
Center: 77%
Above: 6.5%
Below: 8.3%
Center: 51%
2 ANT
Above: 0.0%
Below: 10.0%
Center: 61%
Above: 0.0%
Below: 0.0%
Center: 97%
3 NAO
Above: 2.4%
Below: 2.7%
Center: 63%
Above: 3.2%
Below: 7.8%
Center: 61%
4 SAO
Above: 1.1%
Below: 21.0%
Center: 48%
Above: 2.4%
Below: 21.8%
Center: 45%
5 NPO
Above: 2.2%
Below: 21.0%
Center: 51%
Above: 2.7%
Below: 19.4%
Center: 48%
6 EPO
Above: 0.0%
Below: 1.3%
Center: 79%
Above: 0.0%
Below: 0.0%
Center: 91%
7 SPO
Above: 3.5%
Below: 14.3%
Center: 50%
Above: 8.1%
Below: 21.2%
Center: 33%
8 EIO & SIO
Above: 0.8%
Below: 3.5%
Center: 74%
Above: 5.4%
Below: 7.5%
Center: 52%
9 SOO
Above: 6.2%
Below: 42.3%
Center: 29%
Above: 3.8%
Below: 44.6%
Center: 26%

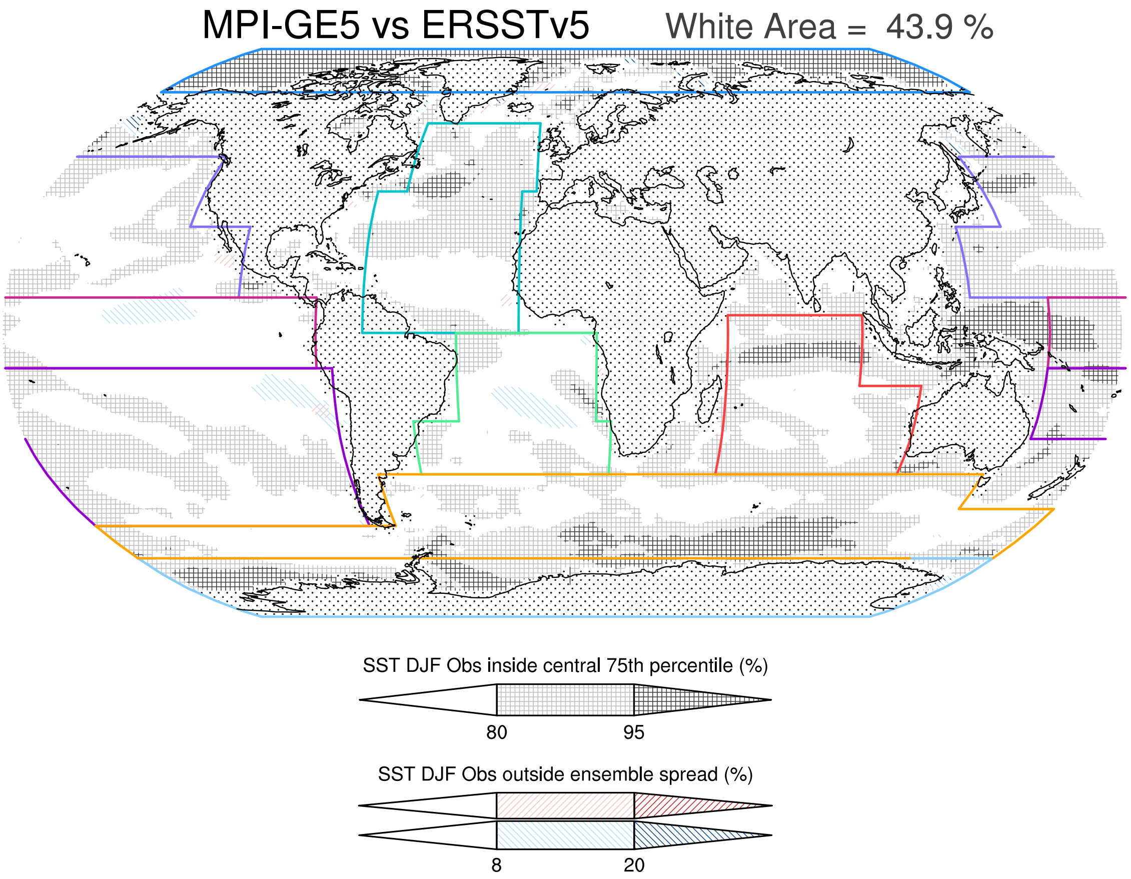
MPI-GE5 vs ERSSTv5
White Area = 43.9 %
SST DJF Obs inside central 75th percentile (%)
80
95
SST DJF Obs outside ensemble spread (%)
8
20

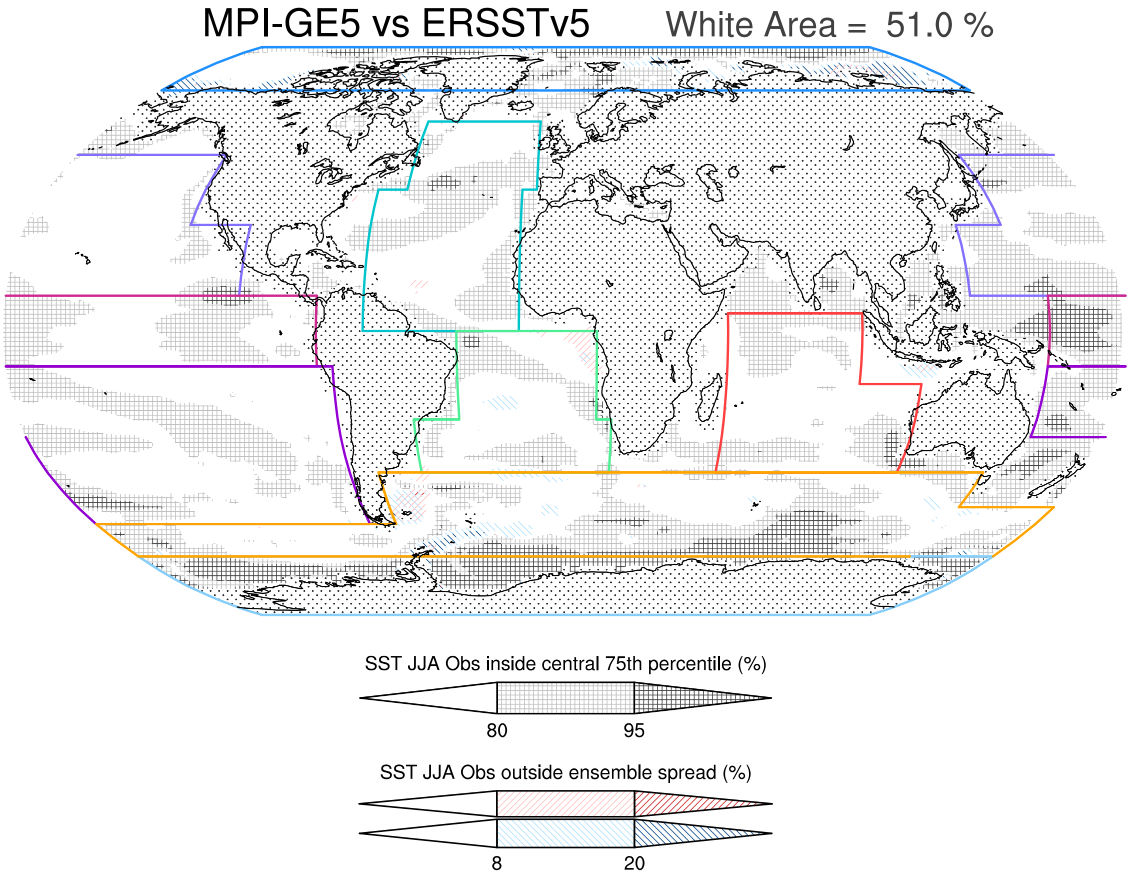
MPI-GE5 vs ERSSTv5
White Area = 51.0 %
SST JJA Obs inside central 75th percentile (%)
80
95
SST JJA Obs outside ensemble spread (%)
8
20

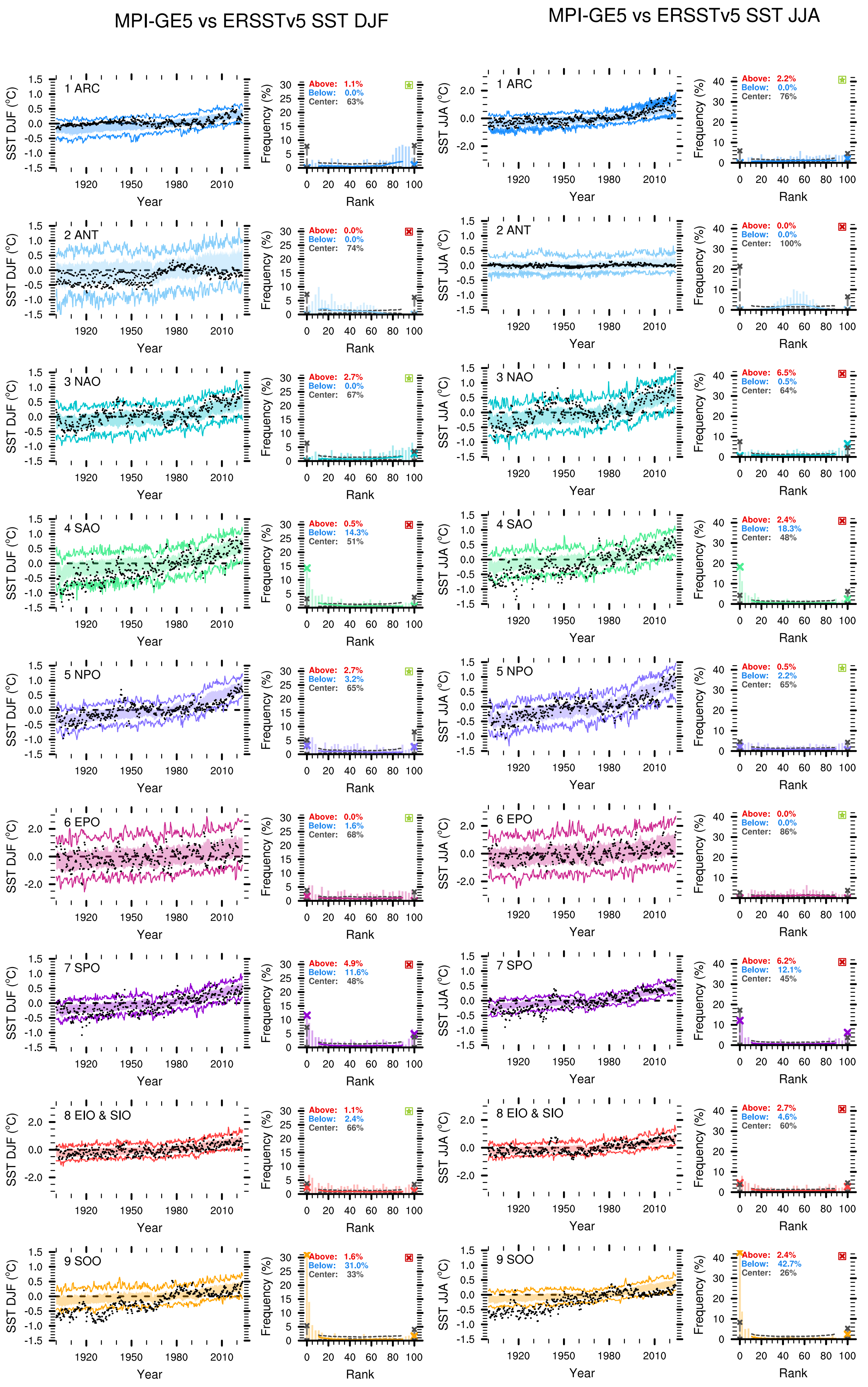
MPI-GE5 vs ERSSTv5 SST DJF
MPI-GE5 vs ERSSTv5 SST JJA
SST DJF (°C)
SST JJA (°C)
Year
Frequency (%)
Rank
1 ARC
Above: 1.1% Below: 0.0% Center: 63%
Above: 2.2% Below: 0.0% Center: 76%
2 ANT
Above: 0.0% Below: 0.0% Center: 74%
Above: 0.0% Below: 0.0% Center: 100%
3 NAO
Above: 2.7% Below: 0.0% Center: 67%
Above: 6.5% Below: 0.5% Center: 64%
4 SAO
Above: 0.5% Below: 14.3% Center: 51%
Above: 2.4% Below: 18.3% Center: 48%
5 NPO
Above: 2.7% Below: 3.2% Center: 65%
Above: 0.5% Below: 2.2% Center: 65%
6 EPO
Above: 0.0% Below: 1.6% Center: 68%
Above: 0.0% Below: 0.0% Center: 86%
7 SPO
Above: 4.9% Below: 11.6% Center: 48%
Above: 6.2% Below: 12.1% Center: 45%
8 EIO & SIO
Above: 1.1% Below: 2.4% Center: 66%
Above: 2.7% Below: 4.6% Center: 60%
9 SOO
Above: 1.6% Below: 31.0% Center: 33%
Above: 2.4% Below: 42.7% Center: 26%

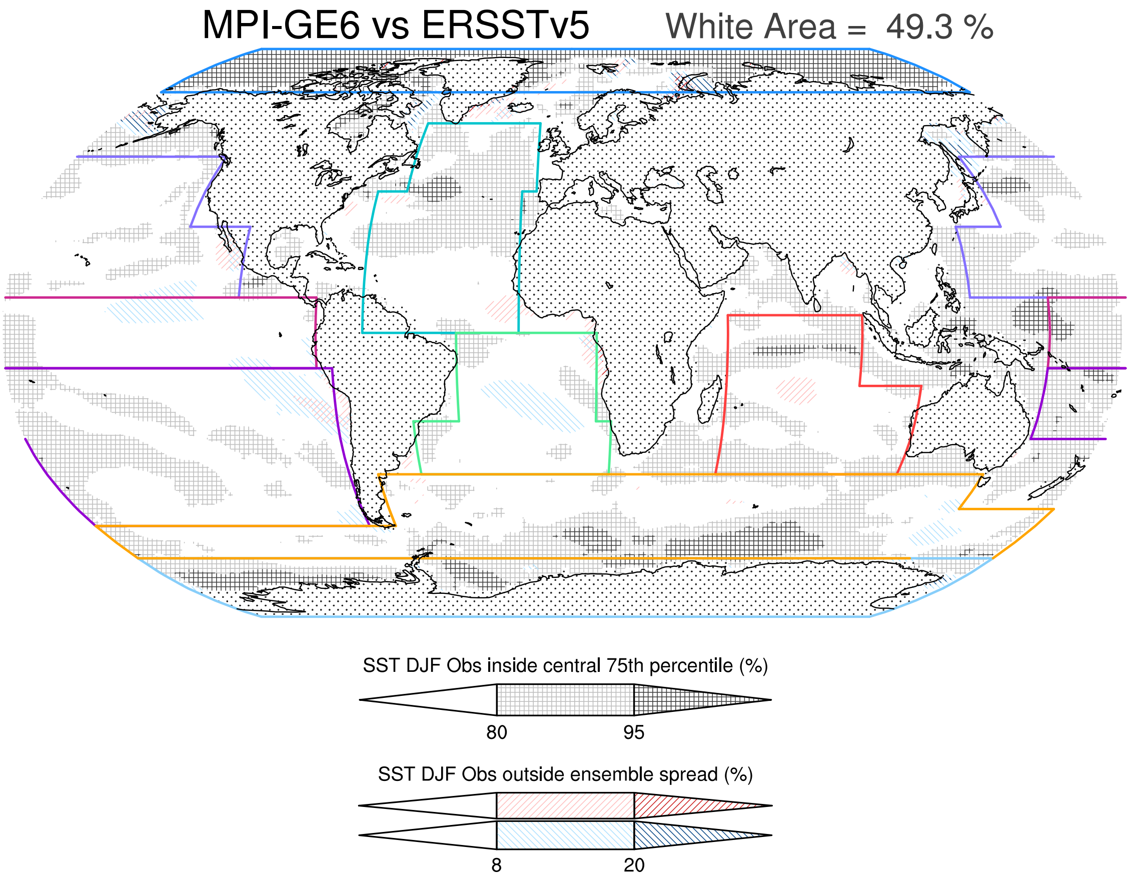
MPI-GE6 vs ERSSTv5
White Area = 49.3 %
SST DJF Obs inside central 75th percentile (%)
80
95
SST DJF Obs outside ensemble spread (%)
8
20

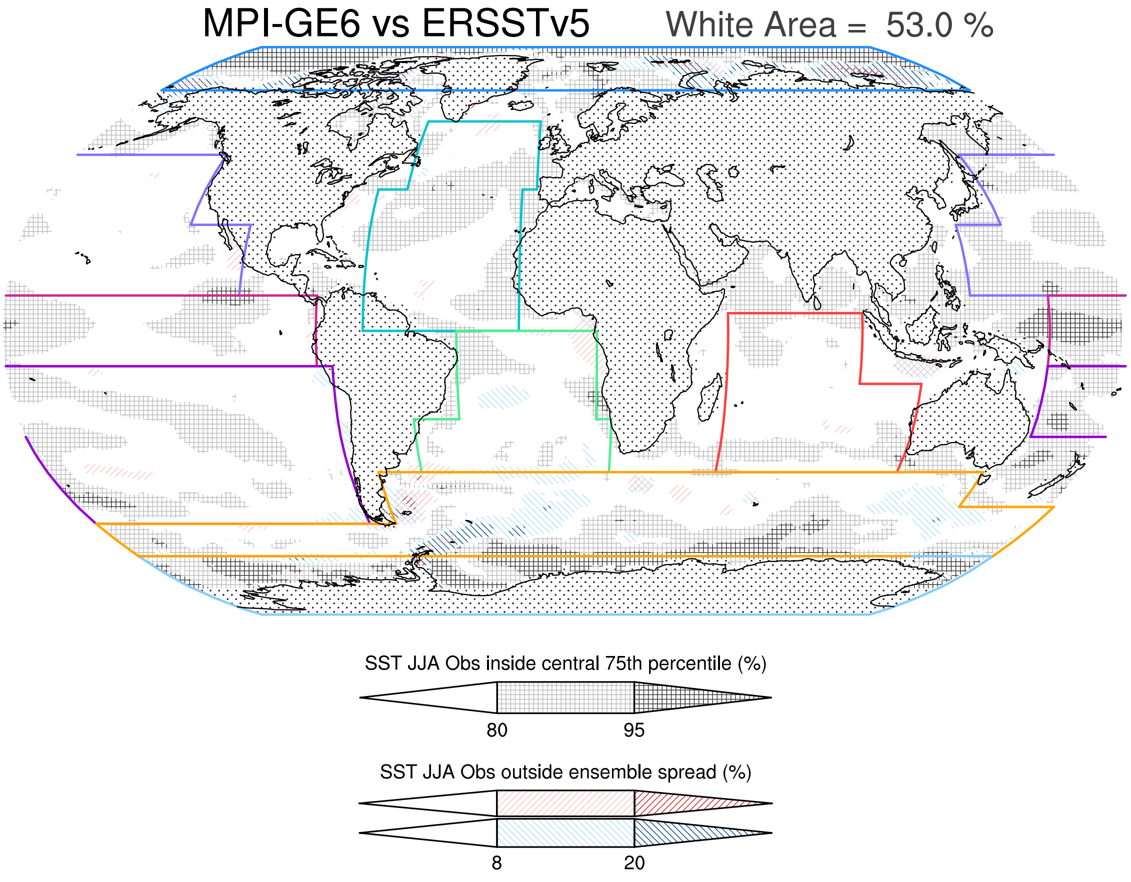
MPI-GE6 vs ERSSTv5
White Area = 53.0 %
SST JJA Obs inside central 75th percentile (%)
80
95
SST JJA Obs outside ensemble spread (%)
8
20

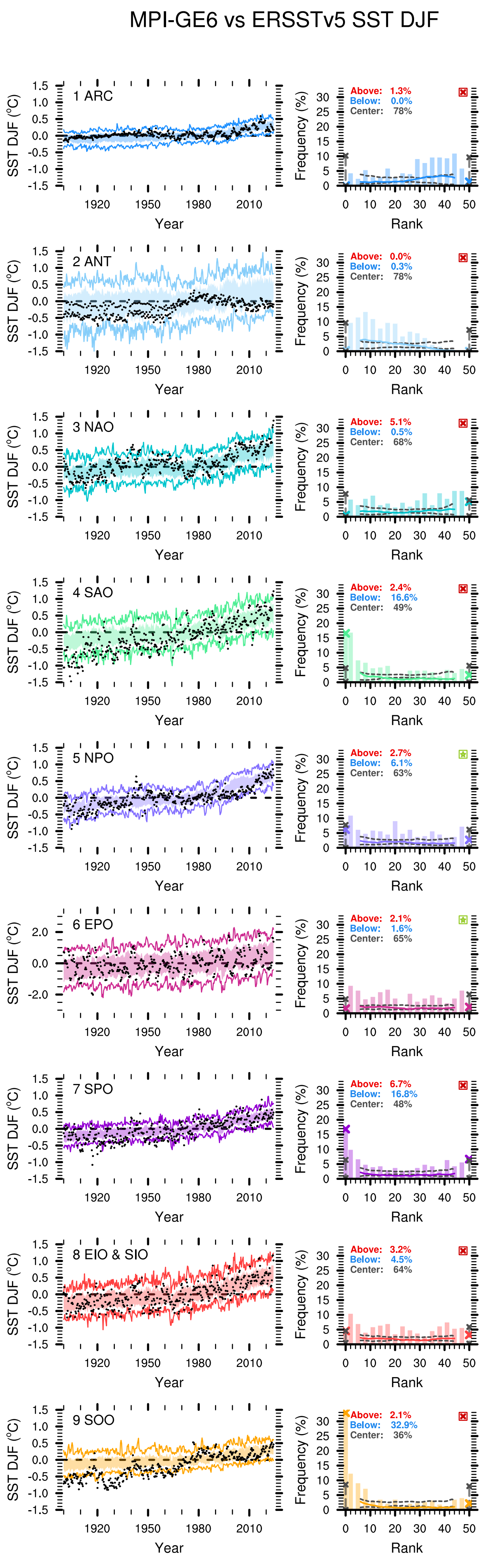
MPI-GE6 vs ERSSTv5 SST DJF
SST DJF (°C)
Year
Frequency (%)
Rank
1 ARC
Above: 1.3%
Below: 0.0%
Center: 78%
2 ANT
Above: 0.0%
Below: 0.3%
Center: 78%
3 NAO
Above: 5.1%
Below: 0.5%
Center: 68%
4 SAO
Above: 2.4%
Below: 16.6%
Center: 49%
5 NPO
Above: 2.7%
Below: 6.1%
Center: 63%
6 EPO
Above: 2.1%
Below: 1.6%
Center: 65%
7 SPO
Above: 6.7%
Below: 16.8%
Center: 48%
8 EIO & SIO
Above: 3.2%
Below: 4.5%
Center: 64%
9 SOO
Above: 2.1%
Below: 32.9%
Center: 36%

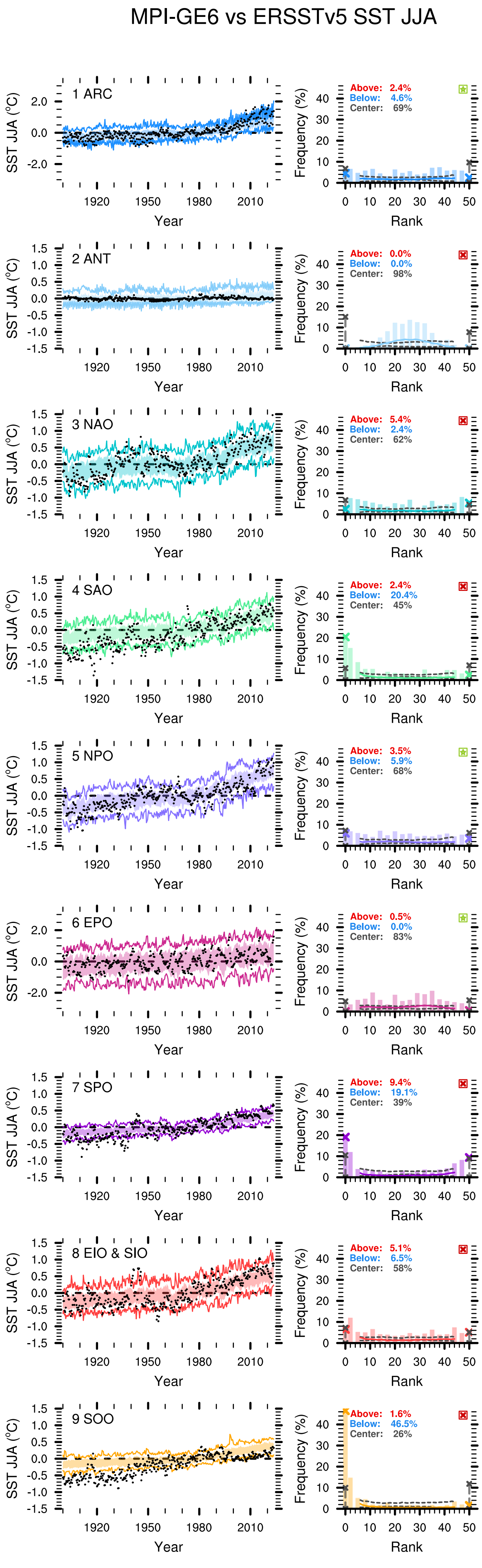
MPI-GE6 vs ERSSTv5 SST JJA
SST JJA (°C)
Year
Frequency (%)
Rank
1 ARC
Above: 2.4%
Below: 4.6%
Center: 69%
2 ANT
Above: 0.0%
Below: 0.0%
Center: 98%
3 NAO
Above: 5.4%
Below: 2.4%
Center: 62%
4 SAO
Above: 2.4%
Below: 20.4%
Center: 45%
5 NPO
Above: 3.5%
Below: 5.9%
Center: 68%
6 EPO
Above: 0.5%
Below: 0.0%
Center: 83%
7 SPO
Above: 9.4%
Below: 19.1%
Center: 39%
8 EIO & SIO
Above: 5.1%
Below: 6.5%
Center: 58%
9 SOO
Above: 1.6%
Below: 46.5%
Center: 26%

2. Global Warming Levels

This section encloses the information of the specific years in which each SMILE reaches the five global mean warming levels used in this study (Table 1). Warming levels are defined relative to a baseline period of 1850-1899 in each SMILE. Each warming level is computed as the year when the global mean surface temperature (annual) from the SMILE ensemble mean crosses an individual warming level.

SMILE	1°C	1.5°C	2°C	3°C	4°C
**ACCESS**	2017	2028	2041	2061	2080
**CanESM2**	2002	2015	2028	2049	2067
**CanESM5**	1999	2010	2020	2039	2054
**CESM-LE**	2017	2031	2042	2060	2079
**CESM2-LE**	2011	2030	2044	2068	2088
**CSIROMK3.6**	2018	2033	2045	2066	2084
**GFDL-ESM2M**	2008	2035	2051	2081	n/a
**GFDL-SPEAR-MED**	2013	2029	2041	2061	2081
**MIROC6**	2022	2038	2052	2075	2095
**MPI-GE5**	2008	2029	2046	2071	2095
**MPI-GE6**	2010	2034	2048	2071	2091

Supplementary Table 1: Warming levels for each single model initial-condition large ensembles (SMILE)

3. Performance Assessment of Constrained and Unconstrained Projections

To assess whether the constrained and unconstrained ensemble projections based on historical performance are more adequate to capture future changes is challenging, as we do not have observations of the future. To partially overcome this, we can compare the projections from the most realistic model over key poorly represented areas in the historical period, CESM2-LE, against projections from the full and constrained ensembles, excluding CESM2-LE (e.g., Main Fig. 8 and Supplementary Figs. 3.1 and 3.2)

CESM2-LE is one of the models closest to historical observations, particularly over the most poorly represented regions, including Australia and South-East Asia (L21-23), South America (L6-8), and Africa (L12-16; the other being CESM-LE), with 15 "good performances" out of 22 across both regions and seasons. Removing CESM2-LE, we recalculate the full and constrained ensemble for future projections and assess how closely it resembles the CESM2-LE future projections, distinguishing between the cases where CESM2-LE adequately captures observations from those where it does not. We explore this comparison of the full vs. constrained projections against the projections of the 'good performing model' CESM2 (Supplementary Figs. 3.1 and 3.2, which replicate Main Figs. 3 and 4, respectively, but now excluding CESM2.

These results highlight how the new constrained (excluding CESM2) and the full ensemble (also excluding CESM2) compare with CESM2. In some cases, the constrained ensemble of adequately performing models is closer to CESM2 when CESM2 is a good performer (e.g., L5 or O7 in Fig. 3.1). However, in other cases it is not (e.g., L3 or L13 in Fig. 3.2). This is likely caused by future variability being more closely linked to past variability performance in some cases. In contrast, in other cases climatic and environmental changes intrinsic to each model and region may affect the changes in the temperature variability distribution differently across different models, even for those that show a common and adequate historical representation. Disentangling the drivers of these diverging behaviours for future variability change across historically adequate performing models for key remaining uncertainty hotspots (e.g., the Amazon basin or the polar Oceans) remains a critical knowledge gap.

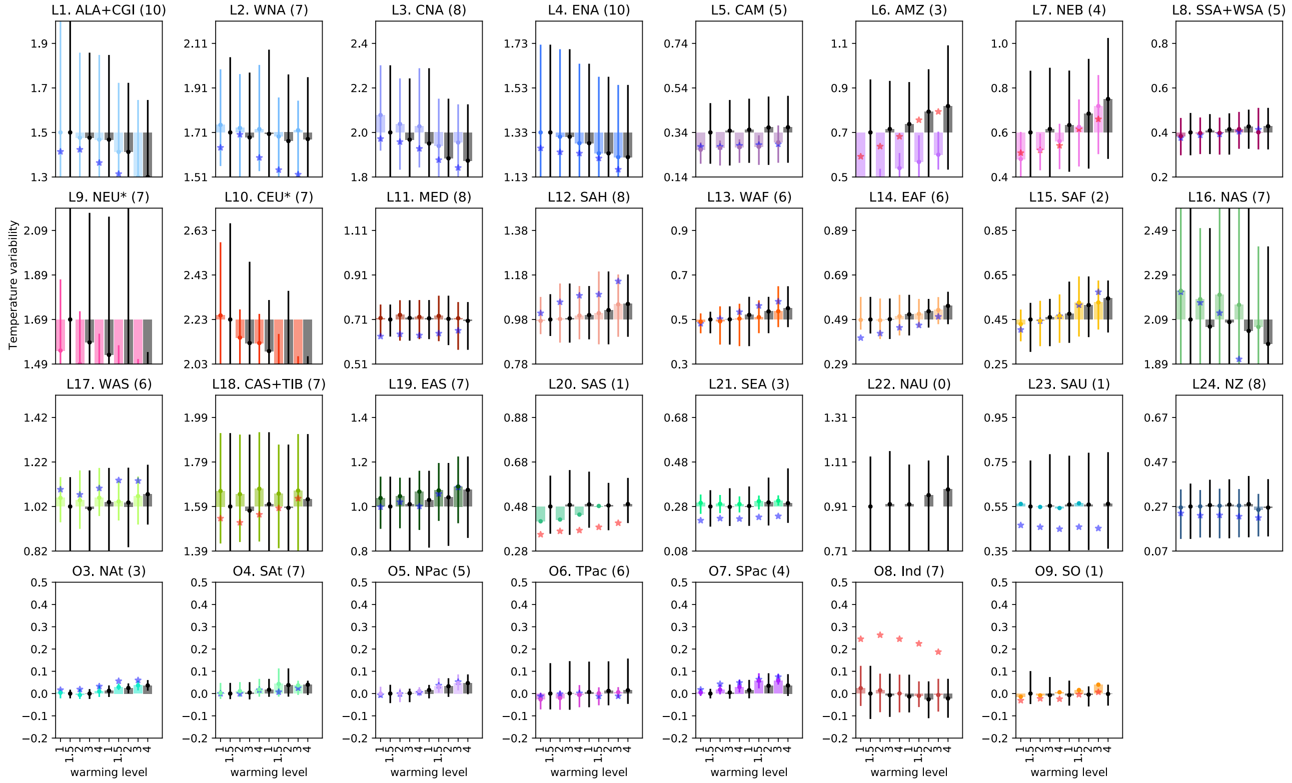

Supplementary Figure 3.1: Comparison of 'new' constrained ensemble without CESM2 against CESM2 projections for December, January, Feberuary (DJF). Blue stars mark regions for which CESM2 offers an adequate representation of historical variability, red stars represent where it does not, as shown in Main Fig. 2a. Multi-ensemble mean DJF temperature variability defined as the standard deviation over each region at each warming level for both the full ensemble (black) and the constrained ensemble (colour). Results are shown relative to the variability at 1 degree of warming in the full ensemble. Errorbars show the full model spread (i.e. the end members of each ensemble), with the fatter errorbars highlighting the 25th and 75th percentiles. The number of models that accurately represent observed variability are shown in brackets. We choose to exclude the polar oceans from these plots due to issues around the ice edges.

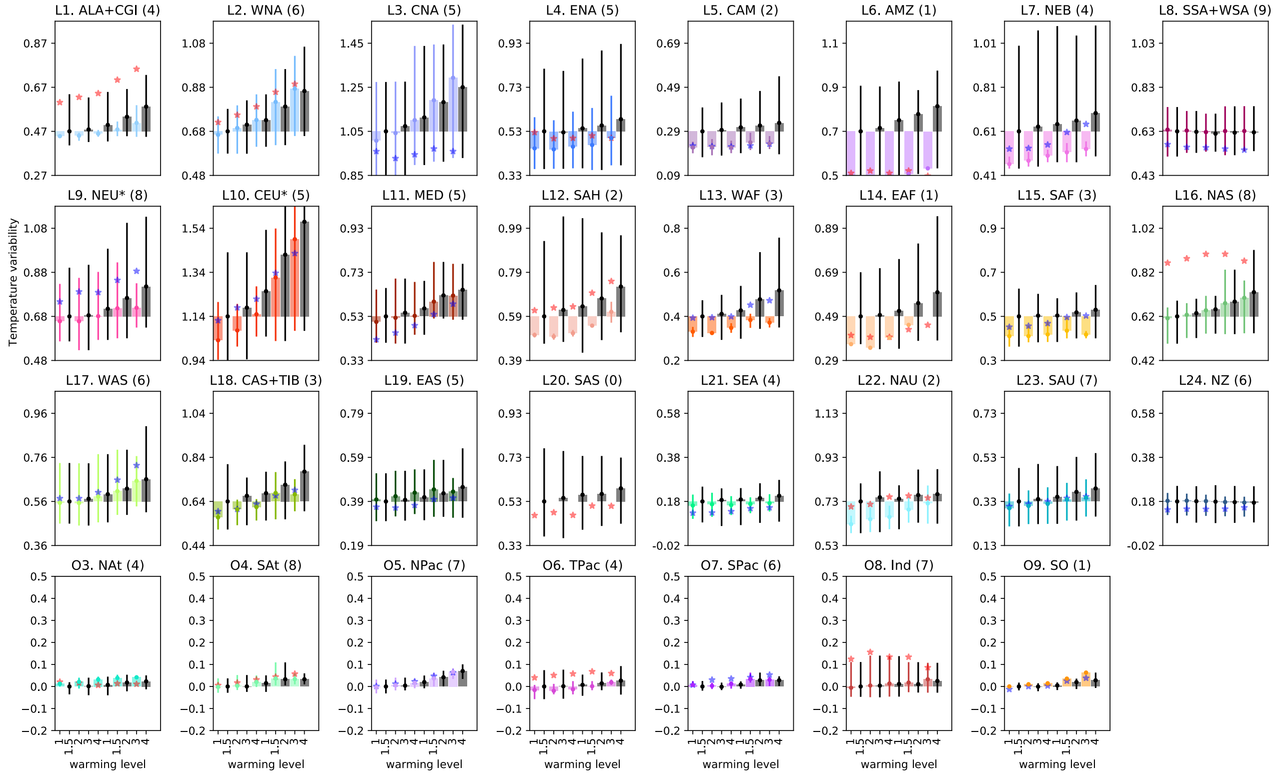

Supplementary Figure 3.2: Comparison of 'new' constrained ensemble without CESM2 against CESM2 projections for June, July, August (JJA). Same as Supplementary Fig. 3.1, but for JJA. Blue stars mark regions for which CESM2 offers an adequate representation of historical variability, red stars represent where it does not, as shown in Main Fig. 2a.
